# Novel molecules and target genes for vegetative heat tolerance in wheat

**DOI:** 10.1002/pei3.10096

**Published:** 2022-12-26

**Authors:** Teresa Rose, Mark Wilkinson, Claudia Lowe, Jiemeng Xu, David Hughes, Kirsty L. Hassall, Keywan Hassani‐Pak, Sandeep Amberkar, Clarice Noleto‐Dias, Jane Ward, Sigrid Heuer

**Affiliations:** ^1^ Rothamsted Research Harpenden UK; ^2^ Institute of Systems, Molecular and Integrative Biology University of Liverpool Liverpool UK; ^3^ National Institute of Agricultural Botany (NIAB) Cambridge UK

**Keywords:** antioxidants, climate resilient crops, heat stress, photosynthesis, propanediol, ROS, secondary metabolites, wheat

## Abstract

To prevent yield losses caused by climate change, it is important to identify naturally tolerant genotypes with traits and related pathways that can be targeted for crop improvement. Here we report on the characterization of contrasting vegetative heat tolerance in two UK bread wheat varieties. Under chronic heat stress, the heat‐tolerant cultivar Cadenza produced an excessive number of tillers which translated into more spikes and higher grain yield compared to heat‐sensitive Paragon. RNAseq and metabolomics analyses revealed that over 5000 genotype‐specific genes were differentially expressed, including photosynthesis‐related genes, which might explain the observed ability of Cadenza to maintain photosynthetic rate under heat stress. Around 400 genes showed a similar heat‐response in both genotypes. Only 71 genes showed a genotype × temperature interaction. As well as known heat‐responsive genes such as heat shock proteins (HSPs), several genes that have not been previously linked to the heat response, particularly in wheat, have been identified, including dehydrins, ankyrin‐repeat protein‐encoding genes, and lipases. Contrary to primary metabolites, secondary metabolites showed a highly differentiated heat response and genotypic differences. These included benzoxazinoid (DIBOA, DIMBOA), and phenylpropanoids and flavonoids with known radical scavenging capacity, which was assessed via the DPPH assay. The most highly heat‐induced metabolite was (glycosylated) propanediol, which is widely used in industry as an anti‐freeze. To our knowledge, this is the first report on its response to stress in plants. The identified metabolites and candidate genes provide novel targets for the development of heat‐tolerant wheat.

## Graphical abstract

Chronic heat stress induces distinct physiological, metabolomic, and transcriptomic changes in a heat‐tolerant *Triticum aestivum* cultivar, Cadenza, compared to a susceptible cultivar, Paragon. Previously uncharacterized metabolites and genes are differentially regulated in the heat‐tolerant cultivar, providing potential for future crop heat tolerance improvement.

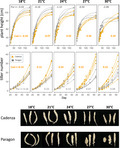

## 1 INTRODUCTION

The global average temperature has increased by 1.15°C above pre‐industrial levels in 2022 (WMO, 2022), and unprecedented measures would be needed globally to limit further warming to 1.5°C (Masson‐Delmotte et al., 2019). Climatic changes cause increasingly severe, erratic weather events, such as droughts, floods, and heat waves. Based on a recent comparative modeling study, it is estimated that each 1°C increase in average temperature will cause significant yield losses, most severely affecting maize (−7.4%) and wheat (−6.0%), followed by rice (−3.2%) and soybean (−3.1%; Zhao et al., 2017). The development of climate resilient crops is thus a matter of global food security.

High‐temperature stress affects every aspect of plant performance, including changes in phenology, growth and development, and yield. During the reproductive phase, high temperature causes pollen sterility and failure of fertilization and seed set (Erena et al., 2021; Jagadish et al., 2010; Xu et al., 2022), whilst post‐anthesis heat stress negatively effects grain size and quality in crops, such as rice (Yan et al., 2021) and wheat (Wang & Liu, 2021). The negative impact of high temperature has been shown for most economically important crops, including maize, barley, tomato, peanut, potato, rapeseed, grapes, citrus, and others (Janni et al., 2020).

The detrimental effect of vegetative heat stress on grain yield has been shown in a recent genetic diversity study in rice, with intolerant genotypes showing up to 80% yield reduction (Cheabu et al., 2018). However, the study also indicated that there are opportunities for tolerance breeding since yield reduction in some genotypes was only 30% An extensive comparative study in wheat using more than 100 genotypes adapted to rain‐fed conditions has shown a 30% reduction in tiller number (TN), and this was associated with a significant reduction in SPAD values, as a measure of chlorophyll content (Qaseem et al., 2019).

Most studies on heat stress are being conducted by exposing plants to very high temperatures (37–47°C for rice, 35–42°C for wheat), usually for a short period of time during the reproductive stage (Janni et al., 2020). However, studies on the effect of high night‐time temperatures (HNT) have made it clear that relatively mild heat stress can cause significant yield losses. For instance, a diversity field study in rice showed significant yield losses when night temperatures were raised by as little as 1‐3°C above ambient (Xu et al., 2021) and this was also observed in wheat (Prasad et al., 2008). Yield losses due to HNT in rice and wheat have been associated with enhanced night‐time respiration and decrease in grain starch (Impa et al., 2019, 2020; Xu et al., 2020).

The negative impact of heat stress on photosynthesis has been investigated in many studies (Hu et al., 2020) and the stay‐green phenotype has been associated with tolerance in wheat (Shirdelmoghanloo et al., 2016), as well as in sorghum and other crops (Kamal et al., 2019). Recent work in wheat has demonstrated the important role of Rubisco and Rubisco activase (Rca) under heat stress; the identification of *Rca* alleles with higher thermotolerance holds promise for the development of crops that maintain photosynthesis under heat stress (Degen et al., 2020; Perdomo et al., 2017; Scafaro et al., 2019). High temperature increases fluidity of membranes which, in chloroplasts, leads to dissociation of the photosystem II (PSII) light‐harvesting complex from thylakoid membranes, disrupting electron transfer and increasing formation of reactive oxygen species (ROS), such as reactive singlet oxygen (Dogra & Kim, 2019; Hu et al., 2020; Sun & Guo, 2016). Singlet oxygen, as well as other ROS, such as H_2_O_2_ or O_2_.^‐^and OH^.^ produced in the chloroplast, mitochondria, and other plant organelles, increase under stress. This causes damage to DNA and membranes due to lipid peroxidation, as well as oxidation of proteins, ultimately leading to cell death (Das & Roychoudhury, 2014). Hence, photoprotection and antioxidants have been recognized as key components for developing heat‐tolerant wheat (Cossani & Reynolds, 2015).

Plants have evolved a range of mechanisms to maintain ROS homeostasis, including synthesizing enzymes that scavenge and detoxify ROS, such as superoxide dismutase (SOD), catalase, ascorbate peroxidase (APX), or glutathione reductase (GR). In addition, chemical, non‐enzymatic ROS scavenging is facilitated by ascorbic acid and reduced glutathione, as well as tryptophane‐derived molecules generated within the flavonoid and phenylpropanoid pathways (Das & Roychoudhury, 2014; Hasanuzzaman et al., 2020). The latter represent a tremendously diverse group of phenolic molecules, many of which have been evaluated in medical research for ROS scavenging activity and beneficial effects on human cancer cell lines (Kopustinskiene et al., 2020). In addition to the antioxidant pathways, plants possess a suite of protective molecular chaperones, including large gene families of heat shock proteins (HSPs), such as HSP70 and HSP90, as well as small HSPs. In wheat, a total of 753 HSP genes were identified, including 169 sHSPs, 114 HSP70s, and 18 HSP90s (Kumar et al., 2020). The best characterized regulatory pathway responsible for upregulation of HSPs under heat stress is ROF1 (Meiri & Breiman, 2009), which enables nuclear import of the transcription factor HSFA2A, a positive regulator of sHSP gene expression, dependent on interaction with HSP90 and FKBP62 (a peptidylprolyl cis/trans isomerase).

To further enhance our understanding of plant heat adaptation during vegetative growth, we have conducted a chronic heat‐stress experiment using two UK spring wheat varieties that differ in their phenotypic response to high temperature stress. RNAseq and metabolomics analysis of vegetative tissue, collected pre‐dawn (AM) and in the afternoon (PM), revealed a set of interesting gene candidates and novel heat‐responsive metabolites, highlighting the importance of maintenance of photosynthesis and radical scavenging.

## 2 MATERIALS AND METHODS

### 2.1 Plant material and growth conditions

Two UK hexaploid spring wheat varieties with shared pedigree, Cadenza (http://wheatpedigree.net/sort/show/9638) and Paragon (http://wheatpedigree.net/sort/show/ 79390) released in 1992 and 1998, respectively, were used in this study. Both varieties were included in the wheat pangenome sequencing project (Walkowiak et al., 2020). Seeds were surface sterilized and pre‐germinated seeds transplanted into fully fertilized potting mix as described by (Oszvald et al., 2022). Plants were well‐watered throughout to avoid drought as a confounding variable.

#### 2.1.1 Pilot study

Plants were grown in a controlled environment glasshouse at 20°C with 16 h light for 1 week before transfer into Sanyo Gallenkamp growth cabinets (70% relative humidity, 13.5 h light, fluorescent light 400 μmol m^−2^ s^−1^). Lights and temperatures were ramped up over a period of 30 min. Plants were grown in a randomized complete block design with four replications under five different maximum day temperatures (*T*_max_) and corresponding 6°C cooler night temperatures: 18°C/12°C, 21°C/15°C, 24°C/18°C, 27°C/21°C and 30°C/24°C day/night. *T*_max_ was maintained for 12 h. At the end of the flowering stage, plants were returned to the glasshouse and supplemental lighting of 250 μmol m^−2^  s^−1^ was provided as required until harvest.

To assess the effect of *T*_max_, plant height (PH; measured from the soil surface to the tip of the longest leaf) and TN were recorded at seven time‐points until 50 days after germination. At harvest, the final plant height (measured from the soil surface to tip of awnless spike) and final tiller and spike number were recorded. The aboveground material was oven‐dried at 80°C for 16 h to determine straw dry weight (DW). Spikes were threshed by hand to determine total seed weight, as well as other detailed seed parameters (see main text) using a MARViN seed analyzer for small seeds (MARViTECH GmbH Germany). Grain hardness, individual seed weight, moisture, and diameter were determined using the Perten Single Kernel Characterisation System (SKCS) 4100 following the manufacturer's procedure. One hundred grains for each plant from each replicate were used for each analysis (Perten Instruments, Calibre Control International Ltd).

### 2.2 Sampling for RNAseq and metabolomics analyses, and physiological measurements

For isolation of RNA and metabolites Cadenza and Paragon plants were grown at *T*_max_ 21°C/15°C and 27°C/21°C (day/night), as described above. An independent set of plants were grown under the same conditions for measurements of photosynthesis and enzyme activities (see below).

Samples for RNAseq and metabolomics analyses were collected pre‐dawn and in the afternoon after plants were exposed to *T*_max_ for 5–6 h. Pre‐dawn leaf samples were analyzed to assess the effect of high night‐time temperature, whereas afternoon samples were analyzed to assess response to acute heat stress.

Plants were removed from the cabinets individually and immediately processed. Targeting the causal factors of differential heat response, plants were sampled at three developmental stages over a period of 47 days, corresponding to 26 (TP1), 39 (TP2), and 47 (TP3) days after germination, before heat‐induced changes in plant development became apparent (Figure S1).

Individual plants were sampled, with four replicates for each treatment and genotype. At TP1 the entire seedling (4–5 leaf stage) was harvested, at TP2 the main tiller was harvested, and at TP3 the youngest fully expanded leaf of the main tiller was harvested. This corresponds to a total of 96 samples, that is, 48 samples for each of Cadenza and Paragon. The fresh weight (FW) was recorded, and the material was immediately frozen in liquid nitrogen and stored at −80°C until further use.

### 2.3 RNAseq analysis

For RNA extraction, the 96 samples were hand‐ground in liquid nitrogen and RNA was extracted from 100 mg aliquots using TRIzol™ according to the manufacturer's protocol (Invitrogen). The RNA samples were analyzed by Novogene (HK) Company Limited (Hong Kong) using an Illumina PE 150 (Q30 ≥ 80%) and was based on a Eukaryotic RNA‐seq (library preparation and sequencing with 250–300 bp inserts) according to the company's specifications (March 2019). The quality of the obtained raw sequences was assessed with FastQC (http://www.bioinformatics.babraham.ac.uk/projects/fastqc/ (2015); “FastQC,” https://qubeshub.org/resources/fastqc) with a mean Q30(%) = 94% and mean clean read ratio of 97.83%. Based on this, six samples were excluded from further analysis. The overall alignment rate for all samples was 91.3% using the HISAT2 aligner (D. Kim et al., 2015). The data are available at the European Nucleotide Archive (ENA) under accession number PRJEB36237 and unique name ena‐STUDY‐ROTHAMSTED RESEARCH‐15‐01‐2020‐14:13:41:981‐1984.

### 2.4 Nuclear magnetic resonance spectroscopy

An aliquot of the ground tissue samples used for RNA extraction was subjected to primary and secondary metabolomic analysis, by nuclear magnetic resonance (NMR) spectroscopy and liquid chromatography‐mass spectrometry (LC‐MS) respectively, at the Rothamsted Research metabolomics facility. Samples were randomized for the analyses to avoid batch effects.

For NMR, ^1^H‐NMR samples were prepared from milled, freeze‐dried leaf samples (15 mg) extracted in triplicate using 80:20 D2O:CD3OD containing 0.01% d4‐trimethylsilylpropionate (TSP) (1 ml) as internal standard. After agitation, samples were extracted at 50°C for 10 min. After centrifugation (5 min at 13,000 rpm), the supernatant was removed to a clean tube and heated to 90°C for 2 min to halt enzyme activity. After cooling and further centrifugation, the supernatant (650 μl) was transferred to a 5 mm NMR tube for analysis. ^1^H‐NMR spectra were acquired under automation at 300°K using an Avance Spectrometer (BrukerBiospin) operating at 600.0528 MHz and equipped with a cryoplatform and a 5 mm triple inverse cryoprobe. Spectra were collected using a water suppression pulse sequence with a 90° pulse and a relaxation delay of 5 s. Each spectrum was acquired using 128 scans of 64,000 data points with a spectral width of 7309.99 Hz. Spectra were automatically Fourier‐transformed using an exponential window with a line broadening value of 0.5 Hz. Phasing and baseline correction were carried out within the instrument software. ^1^H chemical shifts were referenced to d4‐TSP at δ0.00. ^1^H‐NMR spectra were automatically reduced, using Amix (Analysis of MIXtures software, BrukerBiospin), to ASCII files containing integrated regions or “buckets” of 0.01 ppm equal width. Spectral intensities were scaled to the d4‐TSP region (δ0.05 to –0.05). The ASCII file was imported into Microsoft Excel for the addition of sampling/treatment details. Signal intensities for characteristic spectral regions were extracted via comparison to library spectra of known standards run under identical conditions. Quantitation against a known concentration of d4‐TSP was carried out using the known number of hydrogens responsible for each characteristic peak of each metabolite.

### 2.5 Liquid chromatography–mass spectrometry

Leaf samples were prepared as described for NMR, except that the extraction solvent was 80:20 H_2_O:MeOH. UHPLC–MS were recorded with an Dionex UltiMate 3000 RS UHPLC system, equipped with a DAD‐3000 photodiode array detector, coupled to an LTQ‐Orbitrap Elite mass spectrometer (Thermo Fisher Scientific). UHPLC separation was carried out using a reversed‐phase Hypersil GOLD™ column (1.9 μm, 30 × 2.1 mm i.d. Thermo Fisher Scientific) which was maintained at 35°C. The solvent system consisted of water/0.1% formic acid (A) and acetonitrile/0.1% formic acid (B), both Optima™ grade (Thermo Fisher Scientific). Separation was carried out for 40 min under the following conditions: 0–5 min, 0% B; 5–27 min, 31.6% B; 27–34 min, 45% B; 34–37.5 min, 75% B. The flow rate was 0.3 ml/min, and the injection volume was 10 μl. Mass spectra were collected in negative ion mode using an LTQ‐Orbitrap Elite with a heated ESI source (Thermo Fisher Scientific). Spectra were acquired with a resolution of 120,000 over *m/z* 50–1500. The source voltage, sheath gas, auxiliary gas, sweep gas, and capillary temperature were set to 2.5 kV, 35 (arbitrary units), 10 (arbitrary units), 0.0 (arbitrary units), and 350°C, respectively. Default values were used for other acquisition parameters. Automatic MS–MS was performed on the four most abundant ions and an isolation width of *m/z* 2 was used. Ions were fragmented using high‐energy C‐trap dissociation with a normalized collision energy of 65 and an activation time of 0.1 ms. Data were inspected using Xcalibur v. 2.2 (Thermo Fisher Scientific). For comparison between samples, spectra were processed in Compound Discoverer software using the “Untargeted Metabolomics Workflow.” Annotations were made by comparison to known standards run under the same conditions where possible. Putative identifications were made via comparison to literature of known wheat metabolites via the use of Reaxys databases (https://library.udel.edu/databases/reaxys/).

### 2.6 Photosynthesis measurements

Gas exchange and leaf fluorescence were measured using LI‐6400XT portable photosynthesis systems (Li‐Cor Inc.) equipped with leaf chamber fluorometers (Li‐6400XT, Li‐Cor Inc.). Measurements were taken from plants grown under heat (27°C/21°C) and control (21°C/15°C) conditions, as described above, at 6 weeks after transplanting, and again 4 days later.

Measurements were taken during a 2‐h pre‐dawn period and a 2‐h period in the afternoon, after plants were exposed to maximum light intensity and temperature for about 6 h. A total of 16 plants (four plants of each genotype and treatment) were measured in parallel, using two LI‐6400XT systems alternately according to the blocks of the randomized design. Respiration and stomatal conductance were measured during the dark period with an air flow of 200 μmol s^−1^, reference CO_2_ 400 μmol mol^−1^, block temperature was 15°C for plants from the control cabinet, and 21°C for plants from the heat cabinet, photosynthetic active radiation (PAR) of 0 μmol m^−2^ s^−1^. Photosynthesis, stomatal conductance, PSII operating efficiency (ΦPSII), maximum efficiency of PSII (Fv′/Fm′), and VPD were measured during the light period with an air flow of 200 μmol s^−1^, reference CO_2_ 400 μmol mol^−1^, block temperature was 21°C for plants from the control cabinet and 27°C for plants from the heat cabinet, PAR of 1800 μmol m^−2^ s^−1^. Relative humidity in the leaf chamber fluorometers was maintained between 50% and 70% throughout all measurements.

### 2.7 DPPH assay

Cellular radical scavenging capacity using the DPPH (2,2‐diphenyl‐1‐picrylhydrazyl) method was determined for the same freeze‐dried samples (TP1 and TP2) used for the metabolomics analysis. Additional samples were collected from the plants grown for the photosynthesis measurements. For this, the youngest fully extended leaf was harvested from two tillers of four plants per genotype, per heat treatment during peak light intensity and temperature in the sixth hour of the day. Samples (*n* = 16) were flash frozen in liquid nitrogen and stored at −80°C before grinding and freeze‐drying for 24 h. The assay was conducted using 0.1 ml of 0.03 mM DPPH (Sigma Aldrich) added to 0.1 ml extract at different concentrations (1.25–20 mg of plant tissue/ml). After 30 min of incubation in the dark at room temperature, the absorbance at 517 nm was measured with a spectrophotometer. The antioxidant activity of the extracts is expressed as IC_50_, which is the concentration (mg/ml) of extract that inhibits the formation of DPPH radicals by 50%. This was calculated from a four‐parameter log‐logistic curve fitted in R (version 3.6.1) using the “drc” package.

### 2.8 Statistical analyses

#### 2.8.1 Phenotypic data

For the phenotypic data derived from the pilot study, a nonlinear three‐parameter exponential model was fitted to the plant height (PH) $y=a+\left(y_{0}-a\right)\exp\left(-\mathrm{rt}\right)$, where y is the response variable, t is the time, and $y_{0},a,r$ are the model parameters. Only the exponential growth parameter (*r*) was taken forward for downstream analysis. Data included the final PH at harvest (day 191). For the TN an alternative parameterization was used $y=a+b\;\exp\left(\mathrm{rt}\right)$. A high correlation is observed between the linear coefficient and exponential rate, thus in what the gradient of growth at time 0 ($\tilde{r}$ = *r* × *b*) is included in the downstream analysis. Nonlinear modeling was done in R version 3.6.1 using nonlinear least squares. The self‐starting asymptotic regression function was used for the plant height model. For multivariate analysis of harvest variables, partial least squares discriminant analysis (PLS‐DA) was applied to the set of vegetative traits and harvest variables, with a one‐way treatment structure consisting of all cultivar × treatment combinations. Variables were scaled to have unit variance before analysis. PLSDA was done in R version 3.6.1 using the mixOmics package (Rohart et al., 2017).

### 2.9 Metabolomics data statistical analyses

The identified differential primary and secondary metabolites (see above) were analyzed by univariate analysis using Genstat 20th edition (VSN International Ltd) using a nested treatment structure whereby a two‐way factorial structure investigating line and temperature effects was extracted separately for each sampling occasion:

$$\begin{array}{c} \left(\text{sampling}\_\text{time}‐\text{of}‐\mathrm{day}*\;\text{sampling}\;\mathrm{TP}\right)/\\ \left(\right.\text{genotypeTP}1\text{predawn}*\text{tempTP}1\text{predawn}\\ +\text{genotypeTP}1\mathrm{pm}*\text{tempTP}1\mathrm{pm}+\text{genotypeTP}2\text{prewdawn}*\\ \text{tempTP}2\text{predawn}+\text{genotypeTP}2\mathrm{pm}*\text{tempTP}2\mathrm{pm}\\ +\text{genotypeTP}3\text{predawn}*\text{tempTP}3\text{predawn}\\ +\text{genotypeTP}3\mathrm{pm}*\text{tempTP}3\mathrm{pm}\left.\right) \end{array}$$

To establish quantitative differences of metabolites between Cadenza and Paragon, the following nested treatment structure was used whereby a two‐way factorial structure investigating line and temporal effects was extracted separately for each temperature by time of day combination:

$$\begin{array}{c} \left(\text{sampling}\_\text{time}‐\text{of}‐\mathrm{day}*\;\text{temperature}\right)/\\ \left(\right.\text{genotype}2\text{1Cpredawn}*\text{time}2\text{1Cpredawn}\\ +\text{genotype}2\text{1Cpm}*\text{time}2\text{1Cpm}+\text{genotype}2\text{7Cpredawn}*\\ \text{time}2\text{7Cpredawn}+\text{genotype}2\text{7Cpm}*\text{time}2\text{7Cpm}\left.\right) \end{array}$$

Analyses were done by fitting a linear mixed model using REML. Random effects included a term accounting for the cabinet.block. Significance of individual treatment terms was done using the (Kenward & Roger, 1997) approximate *F*‐tests. Multiplicity corrections for the overall false discovery rate were done by applying a Benjamini–Hochberg correction to the one‐way test and applying this correction to the set of independent tests for each metabolite according to the rank, filter, model approach of Hassall & Mead (2018). This process was done independently for the NMR and LC–MS data. Data transformation was used to normalize the data and ensure that downstream statistical model assumptions were met. Significant differences are highlighted by conditional formatting of the respective cells (see Table S2).

The Heatmap was constructed in R version 4.0.3 and show the scaled (mean centered and divided by the standard deviation) of the log2 abundance of all metabolites. Metabolites have been ordered according to a hierarchical clustering with complete linkage of the scaled Euclidean distance.

### 2.10 RNAseq data statistical analyses

Based on a PCA analysis, 16 outliers were identified and removed from further analysis. The remaining 80 samples showed a clear separation between cultivars and sampling time (pre‐dawn AM vs PM) and a small separation between growth temperatures. The BioConductor R package DESeq2 was used to fit a generalized linear model to transcript abundances. This accounts for both the design of the experiment and the statistical distribution of the counts and uses replicates to calculate a fitted value for each gene. Only genes in which eight or more samples (averaged over replicates) had mean raw counts greater than 7 were retained. This gave a counts table for 72,490 genes. The following factorial model was applied to the experiment: Cultivar (P, C) × Temperature (T21, T27) × Time (am, pm) × Development stage (ds1, ds2, ds3). DESeq2 was used to fit the full model to the data and extract 24 fitted values for each gene.

## 3 RESULTS

### 3.1 Phenotypic effect of heat treatment

**FIGURE 1:**
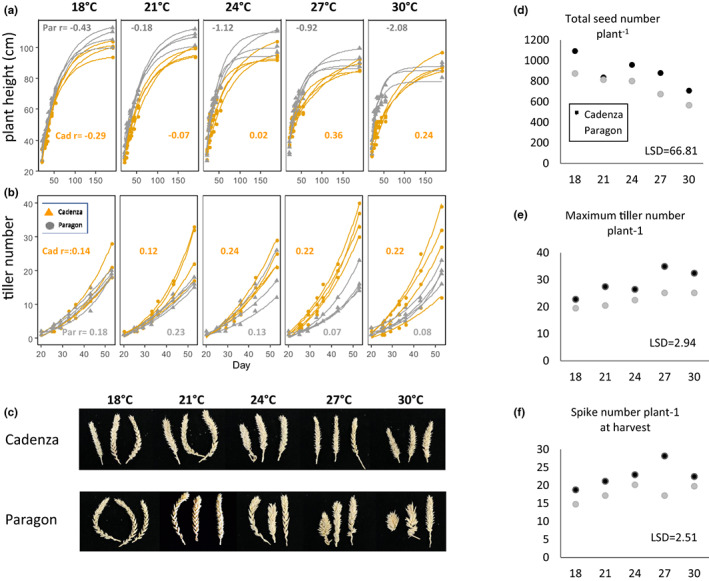
Phenotypic responses of two wheat varieties to different growth temperatures. Cadenza and Paragon plants were grown at the indicated five different Tmax. Models were fitted to describe changes over time in plant height (a, negative r = slower exponential growth rate) and tiller number (b, r = gradient of growth). Tiller number is shown in c. Spike number per plant at harvest is shown in d. Total seed number per plant is shown in e. Representative spikes at maturity are shown in f.

The two UK bread wheat genotypes included in this study both responded to high temperatures with a reduction in plant height (PH), with Paragon showing a significant change in growth rate at 24°C while this occurred at 27°C in Cadenza (Figure 1a). At plant maturity, PH at *T*_max_ 30°C compared to 18°C, was reduced by 24.3% in Paragon (from avg. 107.0 cm to 81.0 cm) and by 10.0% in Cadenza (from avg. 99.7 cm to 89.0 cm; Table S1). In contrast, TN significantly (*p* < .05) increased under heat stress in both genotypes (Figure 1b). This was most pronounced in Cadenza at *T*_max_ 27 and 30°C with an avg. TN of >32 compared to avg. 23 tillers at 18°C. This corresponds to 43%–54% heat‐induced increase in TN (Table S1). In Paragon, TN under heat increased by 30% (from avg. 19.5 at 18°C to 25.3 at 30°C).

In agreement with the observed increase in TN under heat stress, the number of fertile tillers and thus spikes remained higher, although both genotypes aborted some tillers (Cadenza avg. 3.5–10; Paragon 2.3–8.0; Figure 1c,d). In Cadenza, the highest spike number was found at *T*_max_ 27°C with an avg. of 28.3 spikes, a 50.5% increase over an avg. 18.8 spikes at 18°C. At *T*_max_ 30°C, spike number declined but was still higher by an avg. 3.7 spikes compared to 18°C (Figure 1d). Likewise, in Paragon spike number under heat was increased (by avg. 2.5 spikes at 27°C, 16.0%; avg. 5.9 at 30°C, 39.9%; Figure 1d; Table S1).

In contrast, spike length significantly decreased with increasing temperature (Table S1). Overall, this was less pronounced in Cadenza with a reduction of 34% (avg. 12.9 cm, 18°C to 8.52 cm, 30°C) compared to 46.8% reduction in Paragon (from avg. 15.6 to 8.3 cm). This corresponded to a significant reduction in spikelet number per spike which again was less severe in Cadenza (40% reduction) compared to Paragon (50%; Table S1). However, due to the higher number of spikes in Cadenza, the total spikelet number and seed number per plant was significantly higher across all temperatures, with an average of 315 spikelets and 708 seeds per plant at 30°C in Cadenza compared to avg. 280 spikelets and 492 seeds in Paragon (Figure 1; Figure S1). Compared to 18°C, this corresponds to a reduction in seed number per plant by 35.2% in Cadenza and 43.8% in Paragon. Accordingly, total seed weight was reduced by only 33.1% in Cadenza (from avg. 49.6 g to 33.2 g) but 44.5% in Paragon (from avg. 45.4 to 25.2 g; Table S1). Representative spike images are shown in Figure 1f. There was no significant effect of the temperature treatment on seed parameters, such as seed size, area and hardness in either genotype (Table S1).

**FIGURE 2:**
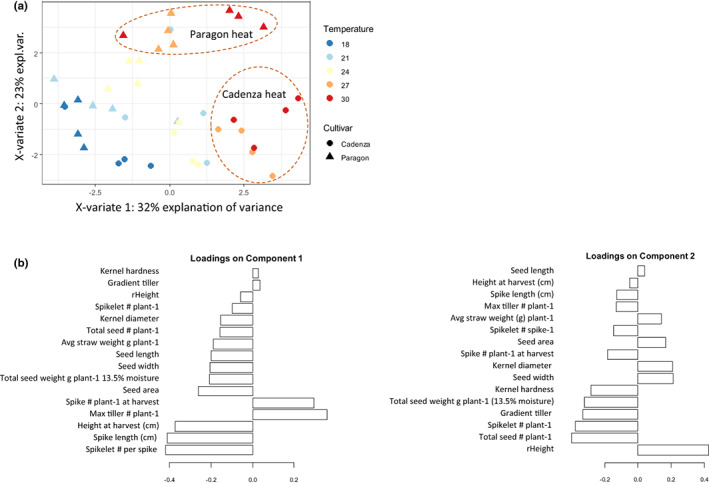
Principal component analysis of phenotypic responses of two wheat varieties grown at five different Tmax The principal component analysis separated Paragon and Cadenza at Tmax 27°C and 30°C (a). The loadings for components 1 and 2 are shown in (b). The actual data are provided in Suppl. Table 1.

The above data suggest a differential heat response, and this was confirmed by a PCA analysis (Figure 2a,b), which distinguished between the five different temperatures and clearly separated Paragon and Cadenza at *T*_max_ 27 and 30°C, based on the higher number of spikes and yield observed in Cadenza.

### 3.2 Metabolite analysis

Apart from tryptophane (see below), only the primary metabolites aspartate and octopamine showed a significant heat response at timepoints (TP) 1 and 2, both AM and PM (Table S2; see columns labeled “pTime.Date.temp”). Other metabolites (glutamate, glutamine, isoleucine, valine, trigonelline, guanosine, uridine) showed a significant heat response at AM and PM only at TP1. However, while these metabolites show might have a role to play in heat response, the absence of a temperature × genotype interaction (see Table S2; columns labeled “pTime.Date.genotype.temp”) suggest that they are not relevant for the higher tolerance observed in Cadenza. Proline, a small amino acid often reported to be upregulated under stress, showed a significant heat response only at TP1, pre‐dawn (Table S2).

**FIGURE 3:**
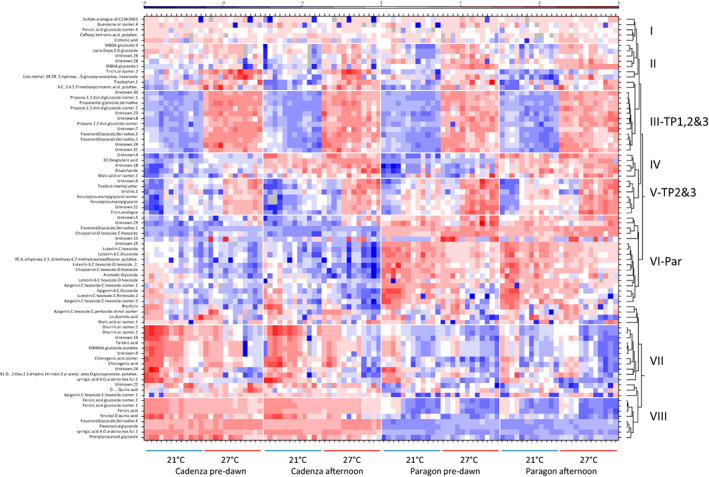
Heat map cluster analysis of metabolites. Leaf samples were collected pre‐dawn and in the afternoon from Cadenza and Paragon plants at Tmax 21°C and Tmax 27°C. For each Tmax, three developmental stages with four replicates each are included (not indicated; see main text for details). The clusters were manually re‐assessed and grouped into the eight groups indicated to the right. The actual data are provided in Suppl. Figure S2 and Suppl. Tables S2a‐d.

In contrast, several secondary metabolites showed a significant response to heat (Table S2). Based on a cluster analysis, eight distinct groups were identified (Figure 3), of which groups II and III contain metabolites that are heat responsive at pre‐dawn and in the afternoon at all three time points, whereas group V was heat responsive only at TP2 and TP3. Metabolites in group IV were not heat‐responsive and were PM specific. The remaining groups contained metabolites specific to Paragon (group VI) and Cadenza (VII, VIII), respectively, indicating constitutive differences in metabolic pathways between these two spring wheat genotypes. Large constitutive differences were also observed in the RNAseq analysis (see below).

**FIGURE 4:**
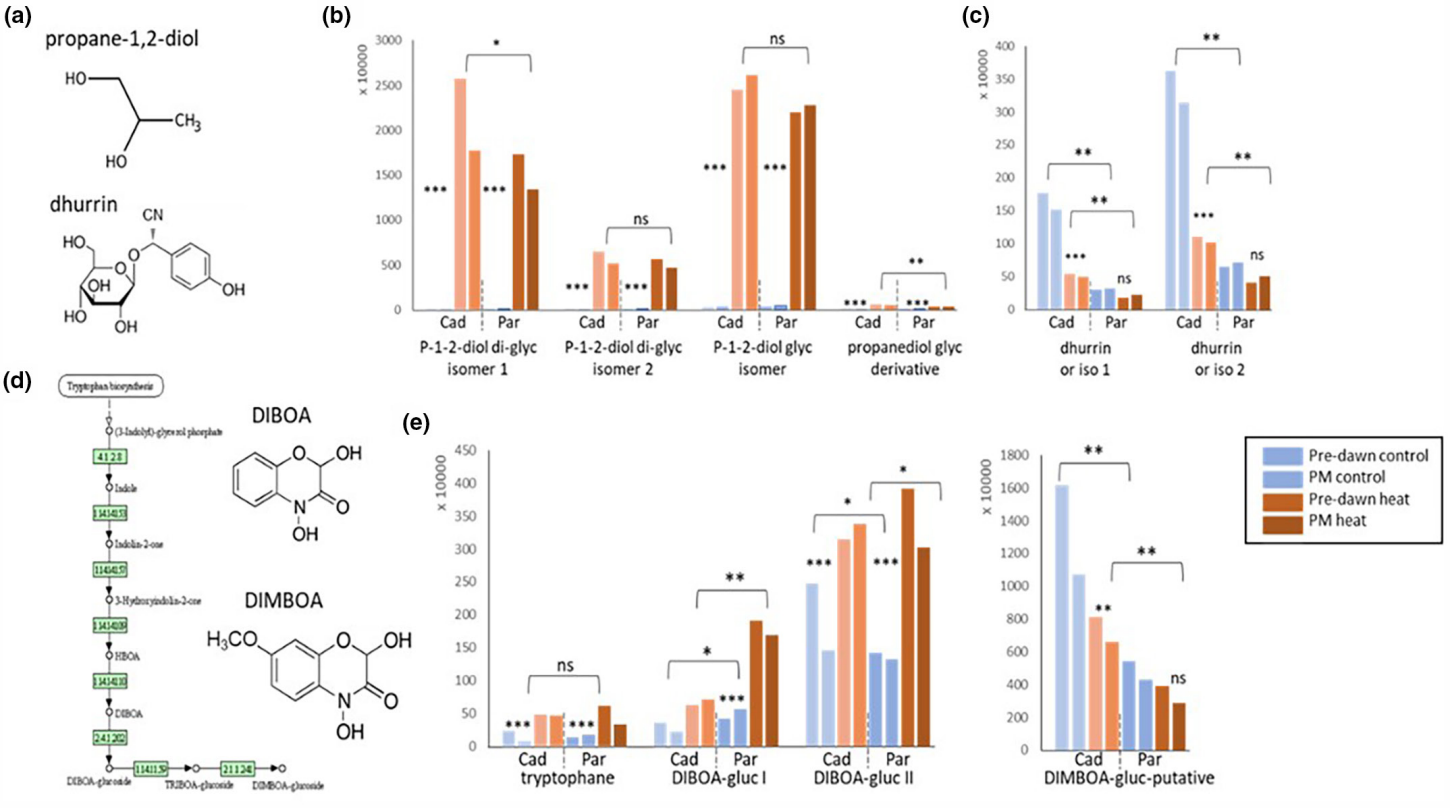
Heat‐responsive metabolites, propanediol, DIBOA, and dhurrin. The chemical structure (a) and abundance of propane‐1‐2‐diol (b) and dhurrin (c) for simplicity averaged across the analysed three developmental stages is shown. The actual data are provided in the Suppl. data Table S2a‐d. The blue bars indicate control growth conditions at 21°C pre‐dawn (left) and afternoon (right). The brown bars indicate growth at 27°C pre‐dawn (left) and PM (right). Two metabolites of the benzoxazinoid pathway, DIMBOA and DIBOA and their average abundance are shown in d and e. Note that the aglycons are shown here. Cad = Cadenza; Par = Paragon. Asterisks *, **, *** indicate significant difference at p < 0.05, 0.001 and 0.0001 (Suppl. Table S2). Ns = non‐significant.

**FIGURE 5:**
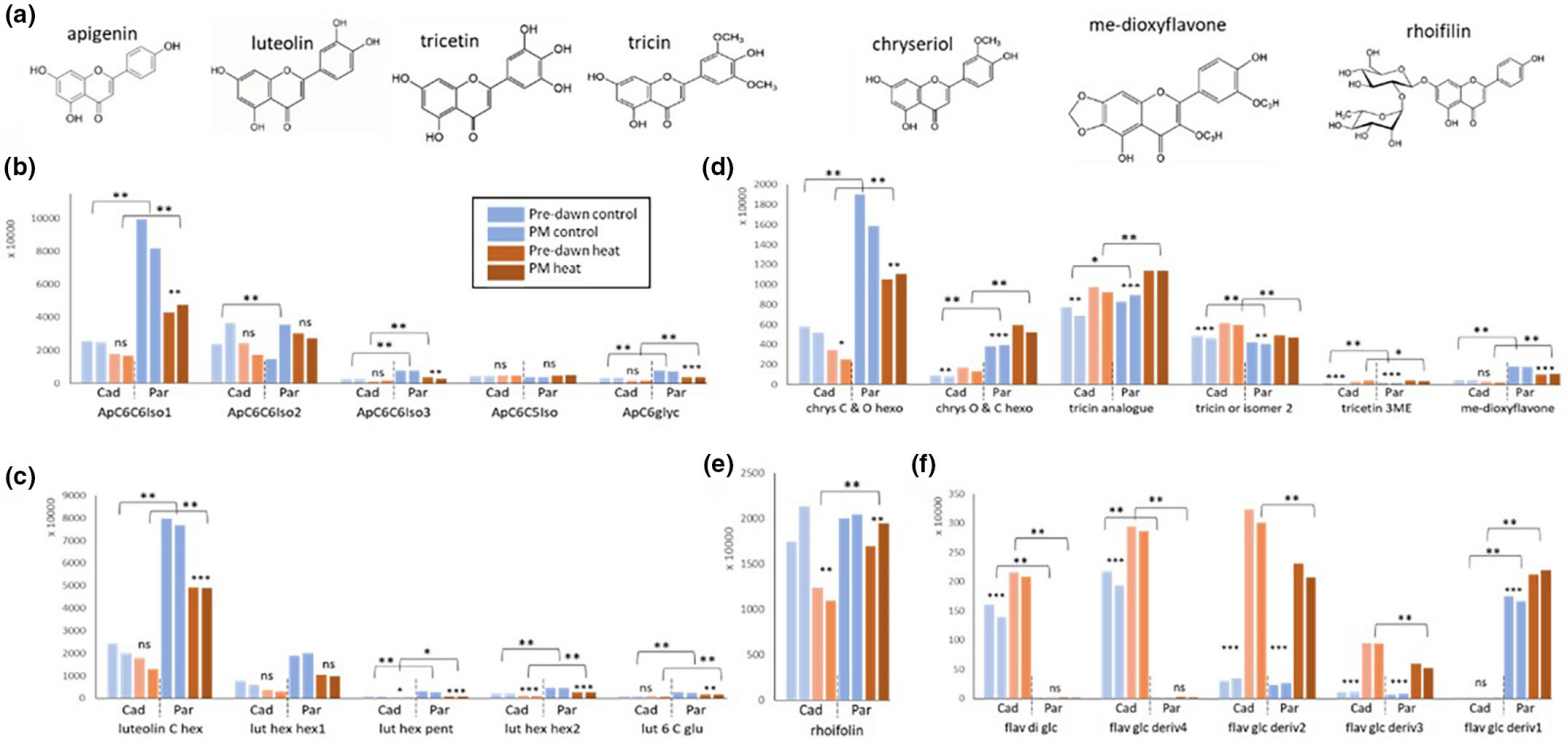
Heat responsive metabolites of the flavonoid pathway. The chemical structures (aglycons) of the identified flavonoid metabolites are shown in (a). Average values are shown as described in the legend of Figure 4 for apigenin‐derived molecules (b) and luteolin‐derivatives (c). Chryseriol derivatives and tricin derivatives, and a methoxy‐dioxyflavone is shown in (d) and rhoifolin in (e). A highly heat induced unspecified differentially glycosylated flavonoid is shown in (f). Cad= Cadenza; Par = Paragon. Asterisks *, **, *** indicate significant difference at p < 0.05, 0.001 and 0.0001 (Suppl. data Table S2). Ns= non‐significant.

**FIGURE 6:**
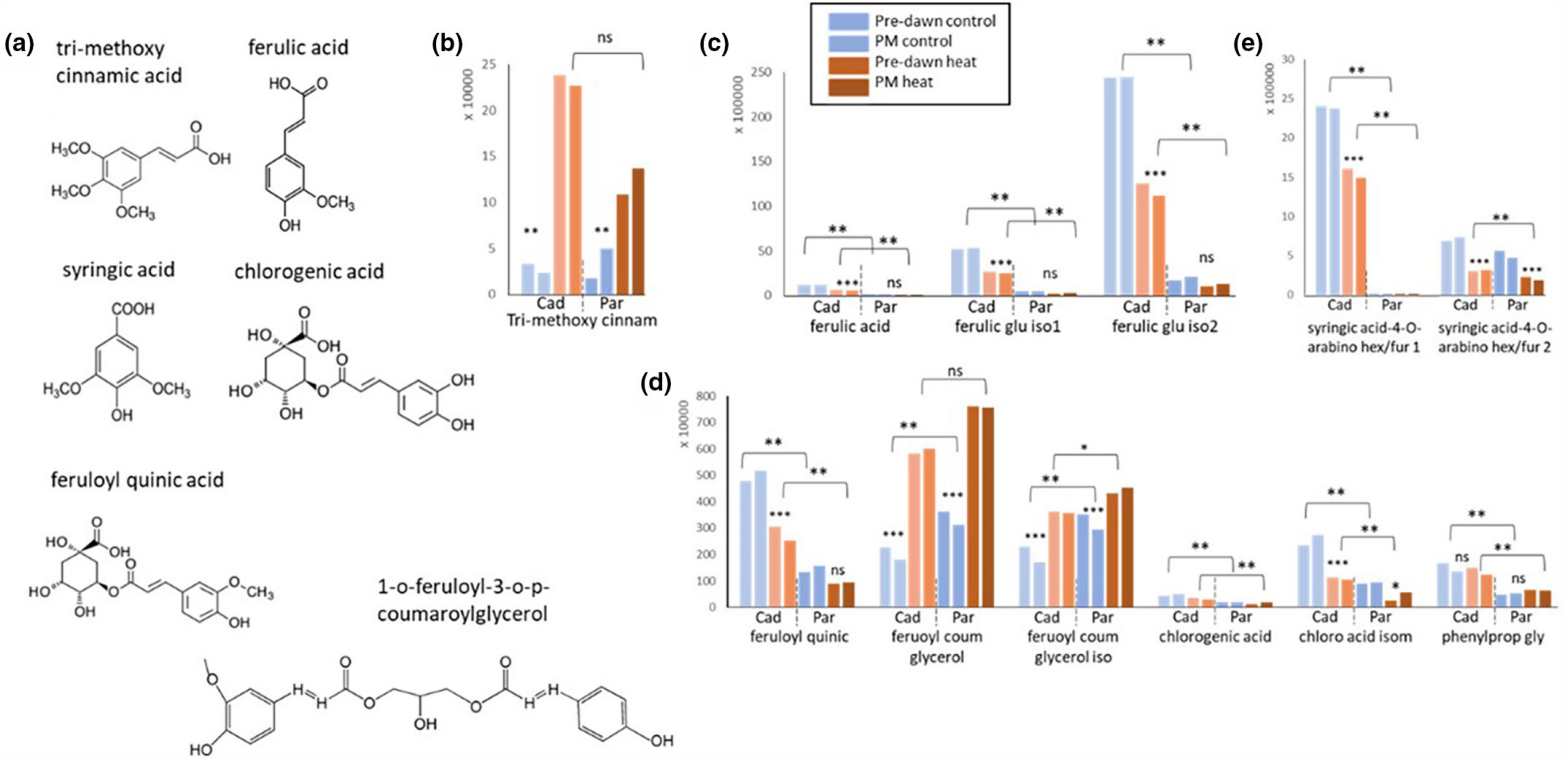
Heat‐responsive metabolites of the phenylpropanoid pathway. The chemical structures (aglycons) of the identified phenylpropanoid metabolites are shown in (a). Average values are shown as described in the legend of Figure 4 for Tri‐methoxy‐cinnamic acid (b), ferulic acid and its glucosylated derivatives (c). Derivatives of ferulic acid and chlorogenic acid are shown in (d) and derivatives of syringic acid are shown in (e). Cad= Cadenza; Par = Paragon. Asterisks *, **, *** indicate significant difference at p < 0.05, 0.001 and 0.0001 (Suppl. data Table S2). Ns= non‐significant.

For simplicity, average values across the three analyzed time points are presented in Figures 4, 5, 6. NMR and LC‐MS raw data and statistical analyses are provided in Table S2. Among the most highly heat‐induced molecules in both genotypes, and part of group III, was propane‐1‐2‐diol (P‐1‐2‐diol) and its glycosylated derivatives (Figure 4a,b). The highest increase was observed for P‐1,2‐diol diglucoside isomer 1, which increased by up to 390‐fold in Paragon and 480‐fold in Cadenza, when averaged across the three time points (Figure 4b). Similarly, the diglycoside isomer 2 increased up to 180‐fold in Cadenza and 190‐fold in Paragon, while a monoglycosylated P‐1,2‐diol isomer and a low‐abundant isomer increased to a lesser extent (85 and 44‐fold in Cadenza, 57 and 19‐fold in Paragon, respectively). Propanediol is a small molecule (Figure 4a) widely used as an anti‐freeze compound and, in the food and cosmetic industry, as an emulsifier to enhance the viscosity of liquids. Despite its industrial value, the role and biosynthesis pathway in plants are not established and we were unable to find any detailed information apart from that it might be synthesized from glycerone‐P, a glycolysis compound (KEGG pathway map00640; propanoate metabolism).

Another interesting molecule identified in this study is dhurrin (isomer 1 and 2; group VII), which, in contrast to propanediol, was downregulated under heat stress (Figure 4a,c, respectively). This was more pronounced in Cadenza (up to 71‐fold reduction) compared to Paragon (threefold reduction), but because both dhurrin isomers were more abundant in Cadenza under control conditions they remained significantly higher (about twofold) under heat stress in Cadenza compared with Paragon. Dhurrin is a cyanogenic aromatic glucoside due to the presence of a CN chemical group (Figure 4a) which can form highly toxic cyanides. However, recently dhurrin has been discussed as a possible N source (Bjarnholt et al., 2018; Rosati et al., 2019). Interestingly, putative cyanide‐detoxifying rhodanese‐domain containing genes (Hatzfeld & Saito, 2000) have been identified in the RNAseq data and showed expression specific to Paragon (TraesCS6A02G005800, TraesCS7D02G531600, TraesCS6A02G005800) or pre‐dawn specific up‐regulation (TraesCS6A02G106000) and down‐regulation (TraesCS5A02G315800, TraesCS5B02G316400), respectively.

Among the molecules in the heat‐responsive group II was glycosylated DIBOA (2,4‐Dihydroxy‐1,4‐benzoxazin‐3‐one) and its methoxylated derivative DIMBOA (putative; Figure 4d,e). These molecules represent aromatic benzoxazinoids derived from tryptophane and are well known for their importance in plant defense, acting as a natural pesticide (see Discussion). In both Cadenza and Paragon, this pathway was differentially regulated with a heat‐induced two‐ to sixfold increase in tryptophane and a corresponding increase in DIBOA glycoside I and II (between 1.2‐ and 4.5‐fold). In contrast, DIMBOA glycoside, which is further downstream in the pathway (Figure 4d), was downregulated under heat stress but remained significantly (around twofold) higher under heat stress in Cadenza compared to Paragon, as was observed for dhurrin (Figure 4c). DIMBOA glucosylation appears to be finely regulated. Two genes encoding DIMBOA glucosidases (TraesCS5B02G294600 and TraesCS5A02G295200) increased significantly under heat in both genotypes. Additional DIMBOA glucosyltransferase genes showed genotype‐specific expression, with higher expression in Paragon (TraesCS2D02G522600, only pre‐dawn) or Cadenza (TraesCS7A02G389200, TraesCS2B02G599800; see below).

Apart from the above, different molecules of two well‐known plant pathways were identified, namely the phenylpropanoid (KEGG map00940) and flavonoid (KEGG map00941) pathways. Overall, the flavonoid pathway appeared more active in Paragon. Specific glycosylated isoforms of apigenin, luteolin and the tricin precursor chrysoeriol (*C*‐hexoside *O*‐hexoside) were highly abundant and about threefold higher in Paragon compared to Cadenza (Figure 5a–d). Due to this, although generally downregulated under heat stress, they remained significantly higher in Paragon (up to 3.8‐fold). This was also the case for less abundant luteolin and apigenin isoforms (Figure 5b,c). Another highly abundant molecule was rhoifilin, a glycosylated apigenin derivative, which also showed lower abundance under heat stress but remained significantly higher in Paragon (Figure 5a,e). Other metabolites of the flavonoid pathway were positively heat‐responsive in both genotypes. This was the case for tricin‐related molecules (tricin analogue, tricin or isomer 2) and tricetin, as well as a putative me‐dioxyflavone (Figure 5a,d). Chrysoeriol appears to be differentially glycosylated and contrary to the abovementioned C‐O glycosylated isoform, the *O*‐hexoside *C*‐hexoside isoform was significantly upregulated under heat stress in both genotypes and more abundant in Paragon (Figure 5d). Lastly, a group of unspecified glycosylated flavonoids was significantly more abundant under heat stress (up to 40% increase; Figure 5f). While some derivates were specific to Cadenza (flavonoid‐diglycoside, derivative 4) or Paragon (derivative 1), others were heat responsive in both genotypes but significantly more abundant in Cadenza (derivatives 2 and 3; Figure 5f).

As was observed for the flavonoids, metabolites of the phenylpropanoid pathway were mostly downregulated under heat stress and showed quantitative differences between the genotypes (Figure 6a–e). No genotypic difference or significant heat response was detected for phenylalanine, the pre‐cursor of the phenylpropanoid pathway (Table S2). Cinnamic acid is the first compound in this pathway, and we found a significant increase in tri‐methoxy cinnamic acid under heat stress to about the same level in both genotypes, though there was a higher fold change in Cadenza (7‐ to 9.6‐fold) compared with Paragon (3‐ to 6‐fold; Figure 6b). Ferulic acid, which is further downstream in this pathway and two more abundant glycosylated isoforms were downregulated under heat stress but remained significantly higher in Cadenza (by 8.5– 12‐fold; Figure 6c). Likewise, related feruloyl‐d‐quinic acid was less abundant under heat stress but remained about threefold higher in Cadenza (Figure 6d). Contrary to this, a positive heat response was observed for feruloyl‐coumaroylglycerol and an isomer, which significantly increased in both genotypes by up to twofold (Figure 6d). Other phenylpropanoid‐related molecules, chlorogenic acid and an unspecified glycosylated phenylpropanoid, were less abundant and further diminished under heat stress, but again remained higher in Cadenza (Figure 6d). This was also the case for two syringic acid‐related molecules of which syringic acid‐4‐*O*‐arabino hex/fur 1 was 12‐ to 14‐fold higher under heat stress in Cadenza compared to Paragon (Figure 6e).

Taken together the data showed that the flavonoid and phenylpropanoid pathways are highly responsive to heat stress, either negatively or positively, and display genotype‐specific differences in abundance and glycosylation pattern. Overall, the latter appeared more active in Cadenza whereas the flavonoid pathway appeared more responsive in Paragon. It is noteworthy, that phenylpropanoids with methoxy groups (‐OCH_3_) were overall more abundant in Cadenza (Figure 6a).

### 3.3 RNAseq analysis

**FIGURE 7:**
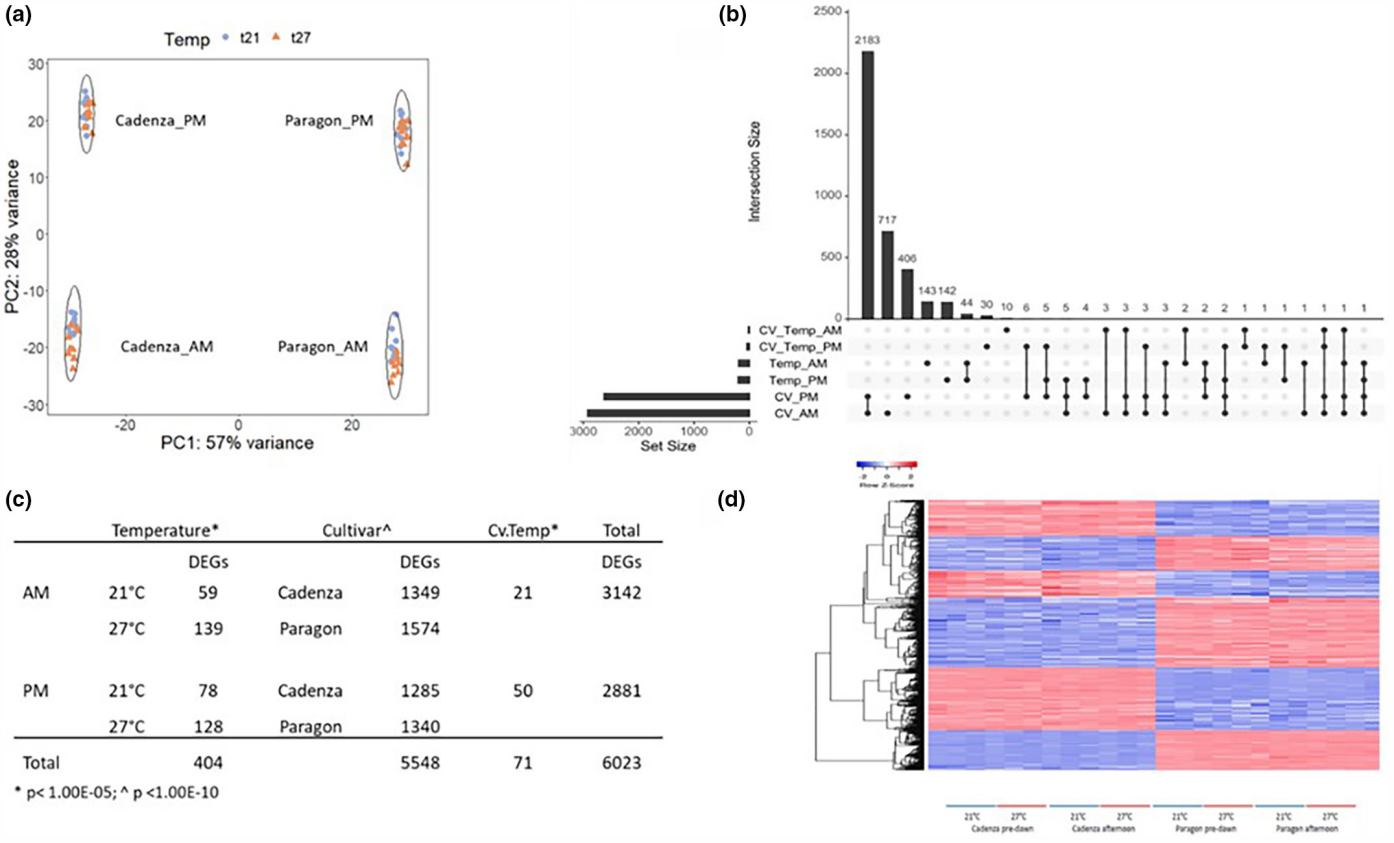
RNAseq data analysis. A principal component analysis of the differentially expressed genes (DEGs; a) clearly separated the two genotypes Cadenza and Paragon and the time‐of‐day of sampling (pre‐dawn and afternoon). Within that, a temperature effect is evident, though to a lesser extent. The number of temperature responsive DEGs, genotype specific DEGs and DEGs that showed an interaction is shown in the upside‐down plot (b) and corresponding table (c). The cluster analysis heat map shown in (d) illustrates the genotype‐specific genes . See legend of Figure 3 for details. Details on the DEGs are provided in Tables 1 and 2, and Suppl. data Tables S3 a‐f.

A PCA analysis of the DEG dataset showed a clear separation of the two genotypes (57% of variance) and time‐of‐day of sampling (AM, PM; 28% of variance), as well as a small effect of temperature, while the three analyzed developmental stages showed little separation (Figure 7a). In total, 6023 genes were differentially expressed, either due to heat treatment, cultivar, or an interaction between the two (Figure 7b,c; Table S3). There were more than 5500 genes that showed a differential expression between Paragon and Cadenza, independent of the treatment, suggesting substantial genotypic differences despite both being modern spring wheats (Figure 7d; Table S3).

A total of 404 genes were temperature‐responsive in both cultivars and 44 of these DEGs were differentially expressed pre‐dawn and PM (Table S3). This group includes a large number of HSPs and other known heat‐responsive genes (see below), which is indicative of the effectiveness of the heat treatment applied in this study.

Most of the genes in the genotype x temperature interaction group were differentially expressed in the afternoon, indicating a genotype‐specific response to exposure to acute heat stress, because *T*_max_ was reached during the day. There was very little overlap between the temperature and cultivar DEGs (Figure 7b), and only 71 genes (53 annotated genes; Figure 7c; Table S3) showed an interaction between the two factors at the thresholds applied. These genes are the most relevant for this study since they might help to explain the observed different levels of heat tolerance between the two genotypes.

### 3.4 Genotype × temperature interaction genes

Among the most heat‐responsive group of genes were those coding for protein chaperones (Table 1; Table S3). For example, two dehydrins, belonging to the late embryo abundant family of disordered chaperones, showed large (log2 8‐19) fold changes, and showed increased transcript abundance under heat in Cadenza while expression decreased in Paragon. Other differentially expressed chaperones include HSP70s (TraesCS6D02G049100, TraesCS6B02G058300, TraesCS6A02G042600), HSP100s, a HSP60 (TraesCS4A02G409100), and a small HSP (TraesCS7A02G232500). Expression of the HSP genes was found to increase more in Paragon than in Cadenza under heat suggesting that Paragon experienced a higher level of stress. However, expression of two HSP70s (TraesCS6B02G058300 and TraesCS1A02G295600) was overall higher in Cadenza under both heat and control conditions (Table S3). A cold shock protein (TraesCS6A02G350100; CS120), which decreased expression in Paragon under heat, remained stable in Cadenza.

**TABLE 1:** Annotated genes showing a cultivar‐specific heat response

Gene ID	DE time of day	Cadenza	Paragon	BaseMean	log2FoldChange	Padj	Annotation
TraesCS5B02G236800	AM	˄	˅	17.25	−3.29	0.04	LIPASE_GDSL DOMAIN‐CONTAINING PROTEIN (PTHR45648:SF4)
TraesCS5A02G238300	AM	˄	—	48.06	−2.81	0.05	LIPASE_GDSL DOMAIN‐CONTAINING PROTEIN (PTHR45648:SF4)
TraesCS4B02G393300	PM	˄	—	152.09	−2.97	0.00	NON‐SPECIFIC LIPID‐TRANSFER PROTEIN 2G (PTHR33214:SF34)
TraesCS7A02G232500	PM	—	˄	210.46	1.25	0.04	23.6 KDA HEAT SHOCK PROTEIN, MITOCHONDRIAL (PTHR46991:SF11)
TraesCS4A02G409100	PM	˅	˄	238.04	1.58	0.00	CHAPERONIN (PTHR45633:SF30)
TraesCS6D02G049100	PM	˅	˄	473.73	1.04	0.00	HEAT SHOCK 70 KDA PROTEIN BIP1‐RELATED (PTHR19375:SF492)
TraesCS6B02G058300	PM	˅	˄	1020.43	1.00	0.03	HEAT SHOCK 70 KDA PROTEIN BIP1‐RELATED (PTHR19375:SF492)
TraesCS6A02G042600	PM	˅	˄	2397.77	0.87	0.00	HEAT SHOCK 70 KDA PROTEIN BIP1‐RELATED (PTHR19375:SF492)
TraesCS1A02G295600	PM	˄	˄˄	123.32	1.95	0.01	70 KDA HEAT SHOCK PROTEIN (PTHR19375:SF493)
TraesCS1D02G284000	PM	˄	˄˄	1745.43	1.30	0.00	HEAT SHOCK COGNATE 71 KDA PROTEIN (PTHR19375:SF395)
TraesCS1A02G285000	PM	− (DS1)	˄ (DS1)	1221.36	1.03	0.05	HEAT SHOCK COGNATE 71 KDA PROTEIN (PTHR19375:SF395)
TraesCS5B02G426700*	AM	˄	˅	17.59	−17.76	0.00	DEHYDRIN RAB15 (PTHR33346:SF33)
TraesCS5B02G426700*	PM	˄	˅	41.68	−8.36	0.03	DEHYDRIN RAB15 (PTHR33346:SF33)
TraesCS6A02G350500	PM	˄	˅	17.89	−19.02	0.00	DEHYDRIN RAB16B (PTHR33346:SF37)
TraesCS6A02G350100	PM	—	˅	115.66	−2.35	0.00	COLD‐SHOCK PROTEIN CS120 (PTHR33346:SF14)
TraesCS4A02G290200*	AM	—	˄	266.54	1.93	0.00	ANK_REP_REGION DOMAIN‐CONTAINING PROTEIN (PTHR46224:SF19)
TraesCS4A02G290200*	PM	—	˄	389.36	2.24	0.00	ANK_REP_REGION DOMAIN‐CONTAINING PROTEIN (PTHR46224:SF19)
TraesCS2B02G587800	PM	—	˄	51.65	2.52	0.05	ANK_REP_REGION DOMAIN‐CONTAINING PROTEIN (PTHR46224:SF44)
TraesCS4A02G290100	PM	—	˄	61.22	1.33	0.04	ANK_REP_REGION DOMAIN‐CONTAINING PROTEIN (PTHR46224:SF19)
TraesCS3A02G045300	AM	—	˄	22.17	4.05	0.05	ALDO_KET_RED DOMAIN‐CONTAINING PROTEIN (PTHR11732:SF209)
TraesCS5B02G080800	AM	˄	—	169.51	−1.41	0.04	F‐BOX DOMAIN‐CONTAINING PROTEIN (PTHR32153:SF43)
TraesCS7B02G485300	AM	˄	˅	775.00	−1.76	0.01	CYSTEINE PROTEASE (PTHR12411:SF803)
TraesCS5D02G180000	PM	˄	—	254.16	−0.97	0.04	AA_TRANS DOMAIN‐CONTAINING PROTEIN (PTHR22950:SF645)
TraesCS5B02G188800	PM	˄ (DS1)	— (DS1)	106.64	−1.30	0.03	HYPERSENSITIVE‐INDUCED RESPONSE PROTEIN‐LIKE PROTEIN 2 (PTHR43327:SF17)
TraesCS6A02G324200	PM	— (DS1)	˅ (DS1)	539.72	−1.11	0.03	PEROXIDASE (PTHR31235:SF333)
TraesCS7B02G160000	PM	˅	˄	59.23	2.83	0.03	BIDIRECTIONAL SUGAR TRANSPORTER SWEET11 (PTHR10791:SF196)
TraesCS7D02G263100	PM	—	˄	73.32	2.37	0.04	BIDIRECTIONAL SUGAR TRANSPORTER SWEET11 (PTHR10791:SF196)
TraesCS2B02G594900	AM	˄	—	69.81	−2.42	0.01	FRUCTAN 6‐EXOHYDROLASE (PTHR31953:SF71)
TraesCS1D02G157000	PM	˅	˄	10.02	3.57	0.04	CYTOKININ DEHYDROGENASE 3 (PTHR13878:SF107)
TraesCS4D02G109500	PM	—	˅	5279.00	−0.83	0.04	ABSCISIC STRESS‐RIPENING PROTEIN 5 (PTHR33801:SF24)
TraesCS4B02G112000	PM	—	˅	5103.46	−1.09	0.03	ABSCISIC STRESS‐RIPENING PROTEIN 5 (PTHR33801:SF24)
TraesCS2B02G100800	AM	˅	˄	580.89	3.17	0.03	DIRIGENT PROTEIN (PTHR46506:SF1)
TraesCS7A02G427100	AM	—	˅	337.54	−1.72	0.01	XYLOGLUCAN ENDOTRANSGLUCOSYLASE/HYDROLASE (PTHR31062:SF239)
TraesCS7D02G419400	AM	—	˅	1397.68	−1.94	0.01	XYLOGLUCAN ENDOTRANSGLUCOSYLASE/HYDROLASE (PTHR31062:SF239)
TraesCS3B02G545900	PM	—	˅	58.30	−1.89	0.03	POLYSACC_SYNT_4 DOMAIN‐CONTAINING PROTEIN (PTHR31444:SF2)
TraesCS2A02G384600	AM	˅	˄	323.03	1.15	0.05	4‐HYDROXY‐7‐METHOXY‐3‐OXO‐3,4‐DIHYDRO‐2H‐1,4‐BENZOXAZIN‐2‐YL GLUCOSIDEBETA‐D‐GLUCOSIDASE (PTHR10353:SF191)
TraesCS7D02G107900	AM	—	˅	444.74	−1.35	0.00	N‐HYDROXYCINNAMOYL/BENZOYLTRANSFERASE, PUTATIVE‐RELATED (PTHR31147:SF33)
TraesCS6A02G239600	AM	˅	—	75.8	2.57	0	ALDEHYDE DEHYDROGENASE (PTHR43570:SF21)
TraesCS5B02G166300	AM	˅	˄	345.62	2.25	0.01	INDOLE‐3‐GLYCEROL‐PHOSPHATE SYNTHASE (PTHR22854:SF14)
TraesCS2D02G189900	PM	˅	˄	29.28	1.84	0.03	PROTEIN CHROMATIN REMODELING 35 (PTHR45821:SF1)
TraesCS6B02G277100	PM	˅	—	119.36	1.37	0.04	AAI DOMAIN‐CONTAINING PROTEIN (PTHR31731:SF24)
TraesCS3B02G299800	PM	˅	—	716.35	1.03	0.04	GLUTAMINE AMIDOTRANSFERASE TYPE‐2 DOMAIN‐CONTAINING PROTEIN (PTHR11938:SF133)
TraesCS6D02G090400	PM	—	˄	929.58	0.72	0.03	Chaperone (PTHR11073:SF44)
TraesCS1B02G320800	PM	˅	—	1066.24	0.68	0.03	TUBULIN BETA‐5 CHAIN (PTHR11588:SF327)
TraesCS4D02G279300	PM	—	˅	771.87	−0.58	0.04	POX (Plant Homeobox) DOMAIN‐CONTAINING PROTEIN (PTHR11850:SF139)
TraesCS2D02G027600	PM	˄	—	470.08	−0.84	0.01	DIOX_N DOMAIN‐CONTAINING PROTEIN (PTHR10209:SF718)
TraesCS3A02G113900	PM	˄	—	2385.82	−1.07	0.04	GALACTINOL—SUCROSE GALACTOSYLTRANSFERASE 2‐RELATED (PTHR31268:SF32)
TraesCS4B02G051000	AM	˄	—	125.5	−1.08	0.03	HYDROLASE_4 DOMAIN‐CONTAINING PROTEIN (PTHR11614:SF155)
TraesCS3B02G039700	PM	˄	—	136.19	−1.22	0.03	ASPERGILLUS NUCLEASE S(1) (PTHR33146:SF21)
TraesCS5B02G518400	PM	˄	—	95.04	−2.21	0.05	BHLH DOMAIN‐CONTAINING PROTEIN (PTHR31945:SF47)
TraesCS1B02G281100	PM	—	˅	49.28	−2.23	0.03	PROTEIN KINASE DOMAIN‐CONTAINING PROTEIN (PTHR24343:SF431)
TraesCS3A02G420900	PM	˄	˅	18.95	−3.03	0.04	F21O3.6 PROTEIN (PTHR31579:SF1)

*Note*: Arrows (˄ and ˅) indicate direction of expression change. Only DEGs with BaseMean over 10 were included. Negative log2 fold change values indicate upregulation in Cadenza relative to Paragon under high temperature and vice versa.

Three genes related to lipid metabolism were differentially expressed, suggesting that heat treatment had an impact on membrane integrity. These genes included two homeologs of a lipase GDSL domain‐containing gene (TraesCS5A02G238300 and TraesCS5B02G236800) and nonspecific lipid‐transfer protein 2G (nsLTP protein 2G; TraesCS4B02G393300). Expression of all three genes were overall higher in Paragon than Cadenza, but Cadenza demonstrated a greater (approx. threefold log2) upregulation under heat (Table 1; Table S3). Three genes encoding Ankyrin repeat domain containing proteins (TraesCS4A02G290200, TraesCS2B02G587800, and TraesCS4A02G290100), which mediate protein–protein interactions, showed higher transcript abundance in Cadenza, and although they increased in Paragon in response to heat, expression levels were still lower in the heat sensitive cultivar (Table 1; Table S3f).

Other genes with a positive heat response in Cadenza included an F‐box domain‐containing protein (TraesCS5B02G080800), which showed overall higher expression in Paragon but no increase under heat. Likewise, an Aa_trans domain‐containing protein showed a Cadenza‐specific increased expression (AM samples). A cysteine protease (TraesCS7B02G485300) showed overall higher expression in Cadenza under both control and heat conditions and was positively heat responsive in TP1. Similarly, a hypersensitive‐induced response protein (TraesCS5B02G188800) showed an early (TP1) positive heat responsive only in Cadenza. Two other genes with differential heat response included an Aldo_ket_red domain‐containing protein (TraesCS3A02G045300) and a peroxidase gene (TraesCS6A02G324200). The former showed overall higher expression and positive heat response in Paragon. In contrast, expression of the peroxidase gene was reduced in Paragon under heat, in both AM and PM samples, while in TP1 in Cadenza, it was unchanged (AM) or showed increased expression (PM) under heat. However, in the other samples, expression under heat was reduced also in Cadenza.

DEGs with roles in carbon partitioning, cell wall and hormone regulation were among the genotype x temperature interaction genes. These included two homeologs of a bidirectional SWEET sugar transporter which decreased under heat in Cadenza (AM and PM), whereas in Paragon, expression remained unchanged in the AM but increased under heat in the PM. In contrast, a fructan‐6‐exohydrolase showed a Cadenza‐specific increase under heat stress in TP1 (AM and PM). Galactinol‐sucrose galactosyltransferase 2‐related also showed an increase in Cadenza under HT in the PM samples. Two homeologs of xyloglucan endotransglucosylase/hydrolase showed decreased expression in Paragon under heat (AM). A generally lower expression and sharp decline under heat stress in Paragon, particularly in TP3, was also observed for polysacc_synt_4 domain‐containing protein. In contrast, a dirigent gene showed a Paragon‐specific increase under heat stress (AM). Two homeologs of abscisic stress‐ripening protein 5 showed differential expression especially in TP1, with a reduced expression under heat stress in Paragon (AM and PM).

Further to this, there are a variety of genes with other functions, most of which are positively heat responsive in Cadenza (Table 1; Table S3) but had no obvious relationship with heat.

### 3.5 Common heat‐responsive genes and constitutive genotypic differences

The transcriptional heat response common to both cultivars was largely related to prevention of protein degradation and to detoxification. Among the positive heat‐responsive genes were several HSP70s, HSP80s, and HSP100s, as well as four peptidylprolyl isomerase genes, which are a component of the well‐studied ROF heat‐response pathway (Meiri & Breiman, 2009). Importantly, in both genotypes 13 genes annotated as glutathione transferases were upregulated, an essential component of non‐enzymatic ROS scavenging. In contrast, enzymes such as catalase, SOD or peroxidases were not induced by the heat stress applied and there was a similar number of genes specific to Cadenza and Paragon, respectively (Table S3), suggesting that both genotypes have a similar capacity for enzymatic ROS scavenging. Interestingly, a large number (11) of aldo‐keto‐reductase domain‐containing proteins showed higher transcript abundance under heat stress (AM and PM; Table S3). Aldo‐keto reductases constitute a superfamily in plants implicated with many processes, including detoxification of reactive aldehydes that form under stress due to lipid peroxidation. Also induced under heat stress were 15 glycosyltransferase genes and these genes are of interest in relation to the observed glycosylation of the phenolic metabolites described above.

The most significant GO terms for the large number of genes that showed a constitutive difference in expression between Cadenza and Paragon were related to phosphorus metabolic processes and kinase activity, as well as DNA packaging (Table S3). Interestingly, there were several hundred protein kinase genes that showed differential expression between the two genotypes, suggesting substantial differences in signaling and post‐translational regulation. This is very interesting and warrants further investigation.

Among the genes that showed high base mean expression (>500) and most significant differences between the two genotypes were several chloroplastic and photosynthesis‐related genes (Table 2). Of particular interest is the higher constitutive expression of a RubisCo small subunit (RbcS) gene in Cadenza (TraesCS2B02G078900; Degen et al. 2021). Another gene with constitutively higher expression in Cadenza is a PsbP domain containing protein (TraesCS4B02G003600), which might play a role in PSII assembly and repair and adaptation to changing light conditions (Che et al., 2020).

**TABLE 2:** Genes with constitutive differential genotypic expression

Higher in	Gene ID	Annotation (panther subfamily)	AM		PM	
			Base mean	log2 fold change	Base mean	log2 fold change
Cadenza	TraesCS2B02G078900	RIBULOSE BISPHOSPHATE CARBOXYLASE SMALL SUBUNIT 1B, CHLOROPLASTIC‐RELATED (PTHR31262:SF10)	2558.92	−4.71	3429.25	−5.33
	TraesCS4D02G309000	PHEOPHORBIDE A OXYGENASE, CHLOROPLASTIC (PTHR21266:SF24)	848.41	−2.23	—	—
	TraesCS4B02G003600	PsbP domain‐containing protein	801.47	−5.76	682.90	−6.16
	TraesCS6B02G063000	PROTEIN LOW PSII ACCUMULATION 3, CHLOROPLASTIC (PTHR34051)	585.03	−2.01	591.17	−2.22
	TraesCS7A02G137000	RIESKE DOMAIN‐CONTAINING PROTEIN (PTHR21266:SF45)	—	—	533.48	−2.53
	TraesCS4B02G003000	ASCORBATE TRANSPORTER, CHLOROPLASTIC (PTHR11662:SF255)	—	—	557.09	−1.81
	TraesCS4B02G009800	5‐AMINO‐6‐(5‐PHOSPHO‐D‐RIBITYLAMINO)URACIL PHOSPHATASE, CHLOROPLASTIC (PTHR47108:SF1)	519.92	−6.24	—	—
	TraesCS6B02G058300	HEAT SHOCK 70 KDA PROTEIN BIP1‐RELATED (PTHR19375:SF492)	870.05	−2.3	1020.43	−2.40
	TraesCS6A02G000700	HSC70‐INTERACTING PROTEIN (PTHR45883:SF2)	749.08	−1.49	—	—
	TraesCS3B02G041900	TRYPTOPHAN SYNTHASE (PTHR43406:SF8)	—	—	772.08	−1.67
	TraesCS2B02G204500	3BETA_HSD DOMAIN‐CONTAINING PROTEIN (PTHR10366:SF696)	—	—	515.79	−12.49
Paragon	TraesCS2D02G500600	GLUTAMINE SYNTHETASE (PTHR20852:SF57) note SH:that is GS2 and its in the chloroplast	8860.64	3.22	10158.82	2.90
	TraesCS2B02G133500	PHOTOSYSTEM I REACTION CENTER SUBUNIT N, CHLOROPLASTIC (PTHR36814:SF1)	5430.49	3.21	8040.33	2.98
	TraesCS1B02G317500	CHLOROPHYLL A‐B BINDING PROTEIN, CHLOROPLASTIC (PTHR21649:SF150)	—	—	6820.14	4.65
	TraesCS2A02G590600	PROTOCHLOROPHYLLIDE REDUCTASE A, CHLOROPLASTIC (PTHR44419:SF6)	—	—	6343.22	1.22
	TraesCS7D02G553300	PHYTOENE SYNTHASE, CHLOROPLASTIC (PTHR31480:SF2)	1348.03	9.37	1043.81	8.59
	TraesCS7B02G486500	PHOTOSYSTEM II STABILITY/ASSEMBLY FACTOR HCF136, CHLOROPLASTIC (PTHR47199:SF2)	1039.81	2.72	999.80	2.41
	TraesCS6D02G122800	NADPH‐DEPENDENT ALKENAL/ONE OXIDOREDUCTASE, CHLOROPLASTIC (PTHR44573:SF1)	1050.52	1.2	—	—
	TraesCS1B02G308500	PROTEIN MAINTENANCE OF PSII UNDER HIGH LIGHT 1 (PTHR35753:SF2)	918.57	1.36	—	—
	TraesCS1B02G237700	CHLOROPHYLL SYNTHASE, CHLOROPLASTIC (PTHR42723:SF1)	660.6	1.62	662.02	1.42
	TraesCS1B02G237701	CHLOROPHYLL SYNTHASE, CHLOROPLASTIC (PTHR42723:SF1)	661.6	1.63	662.02	1.42
	TraesCS3B02G490600	GLUTATHIONE S‐TRANSFERASE F13 (PTHR43900:SF72)	1569.3	1.26	2498.24	1.78
	TraesCS6A02G000300	SKP1‐LIKE PROTEIN 1 (PTHR11165:SF92)	1037.75	3.94	832.51	3.88
	TraesCS1D02G454400	HISTONE DEACETYLASE HDT2‐like (XP_044452191.1; NCBI)	514.59	5.55	518.65	5.77
	TraesCS2D02G491700	TLD‐DOMAIN CONTAINING NUCLEOLAR PROTEIN (PTHR23354:SF104)	523.47	1.17	322.26	1.14
	TraesCS3B02G612000	FLAVONE 3′‐O‐METHYLTRANSFERASE 1 (PTHR11746:SF199) (CL: Caffeic acid O‐methyltransferase?)	1279.95	1.22	—	—

In this context, it is noteworthy that, although there was no significant difference between the genotypes, three *rubisco activase 1* (*Rca1*) genes (TraesCS4A02G177600, TraesCS4D02G134900, TraesCS4B02G140200; Table S3; Degen et al., 2021), were significantly increased under heat stress.

The differences in these and other photosynthesis‐related genes might be directly relevant for the observed differences in photosynthetic capacity between Cadenza and Paragon (see below). Interestingly, one of the most highly expressed genes, which showed significantly higher expression in Paragon, was a glutamine synthetase 2 (*GS2*) gene (TraesCS2D02G500600). One of the main roles of chloroplastic GS2 is the reassimilation of photorespiratory ammonium.

### 3.6 Chemical ROS scavenging

**FIGURE 8:**
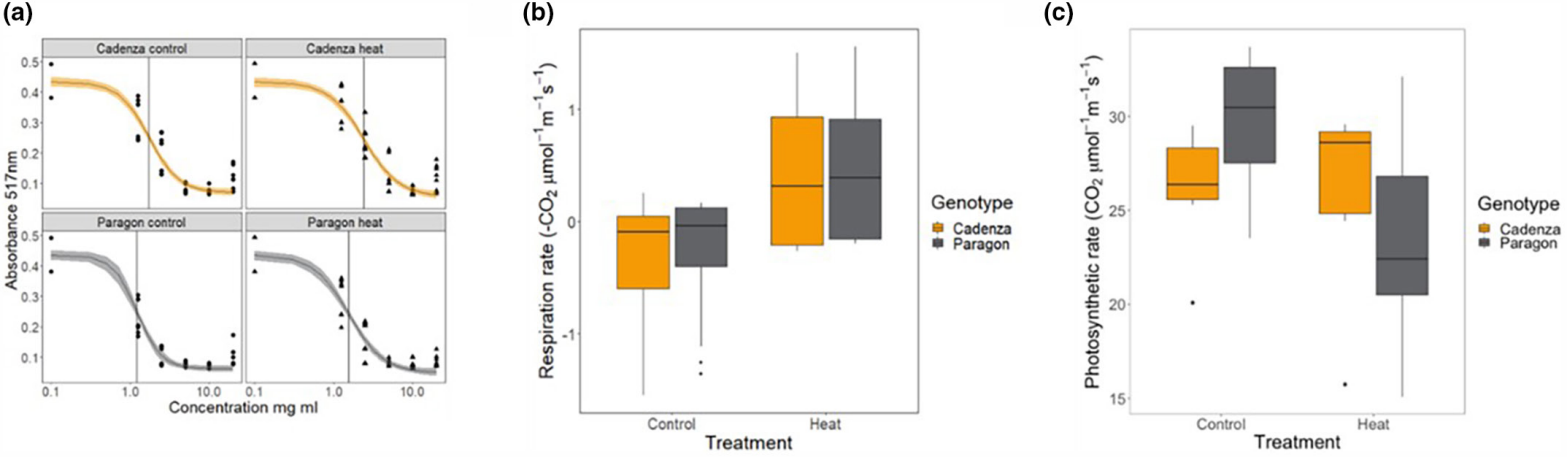
Chemical radical scavenging and photosynthesis under heat stress.Radical scavenging capacity was measured using the DPPH assay (a) showing a decrease under heat stress in both genotypes and an overall lower IC50 values in Paragon, indicative of a higher chemical scavenging capacity. Photosynthetic parameters were measured in Paragon and Cadenza plants grown under control and heat stress conditions using a Li‐Cor Li‐6400XT. Respiration rate increased under heat stress (b) and was overall similar in the two genotypes. The photosynthetic rate was higher in Paragon under control conditions but significantly lower under heat conditions compared with Cadenza (c; Suppl. Figure S3).

The capacity for chemical radical scavenging was assessed in control and heat‐stressed plants in the afternoon in two independent experiments with similar results using the standard DPPH assay. Data of the second experiment, for which an independent set of plants was grown under control and heat conditions, are shown in Figure 8a. Data are expressed as IC_50_ values, that is, the concentration of plant leaf extract required to reduce the activity of DPPH by 50%. Smaller IC_50_ values are therefore indicative of a greater free‐radical quenching capacity. The data show overall smaller IC_50_ values in Paragon and an increase in IC_50_ under heat stress in both genotypes, suggesting that the scavenging capacity under heat is compromised.

### 3.7 Photosynthesis measurement

The same plants sampled for the DPPH assay were used for Licor measurements pre‐dawn and in the afternoon using 6‐week‐old plants. There were no significant differences in ΦPSII, Fv'Fm' or dark respiration between the two genotypes under heat stress (Figure 8b; Figure S3). In contrast, the photosynthetic rate was significantly lower under heat stress compared with control conditions, based on a linear regression with photosynthetic rate predicted by genotype and treatment (Figure 8c; Figure S3). There was a significant interaction between genotype and treatment, with Paragon showing a significantly higher photosynthetic rate under control conditions. However, under heat stress the photosynthetic rate significantly dropped in Paragon while Cadenza was able to maintain its photosynthetic rate in agreement with its suggested heat tolerance.

Vapor pressure deficit (VPD) based on air temperature was significantly higher under high temperatures compared to control (Figure S3), although the true values were not so high as to suggest the plants were experiencing drought stress. There were no significant differences in VPD based on leaf temperature. Despite higher air VPD under heat treatment, no differences in stomatal conductance were observed (Figure S3), indicating that there were no changes in transpiration.

## 4 DISCUSSION

Comparative phenotypic assessment of two spring wheat varieties, Cadenza and Paragon, showed differences in plant height and TN at a *T*_max_ of 24°C, and a progressive reduction in yield with increasing temperature. Cadenza was more resilient overall and maintained a higher yield under stress compared to Paragon. This was attributed to excessive tillering and a higher final spike number under high temperatures. Subsequent metabolomic and RNAseq analyses conducted to identify causal factors revealed an interesting set of heat‐responsive metabolites and genes, as well as significant constitutive genotypic differences.

### 4.1 Anti‐freeze molecules and other unusual metabolites respond to heat stress in wheat

The metabolites that showed the most significant heat response in both genotypes were three P‐1‐2‐diol glycosides. The non‐glycosylated molecule P‐1‐2‐diol (or propylene glycol) has a broad range of industrial uses, for example, as an anti‐freeze compound, as an additive in cosmetics and medicines, as well as being an emulsifier in food, such as ice cream. To our knowledge, this is the first report of P‐1‐2‐diol in relation to heat stress and only one relevant plant‐based study has been published, suggesting that P‐1‐2‐diol, as a compound in an essential oil product, could promote root growth in lettuce (Nakajima et al., 2005). According to KEGG, P‐1‐2‐diol is a component of the propanoate metabolism pathway (map00640) and is synthesized from glycolysis‐derived glycerone‐P. Industrial uses suggest that it enhances viscosity of fluids, and it is interesting to speculate that high levels of P‐1‐2‐diol in the cytoplasm might reduce heat‐induced increase in Brownian motion, or otherwise stabilize cellular compounds and metabolic processes. It would be interesting to assess whether P‐1‐2‐diol levels also increase under cold stress.

Also significantly increased under heat stress are two DIBOA glycosides and the precursor tryptophane. In contrast, DIMBOA glycoside, the end‐product of the benzoxazinoid pathway, which carries an additional methoxy group, was less abundant under heat compared to controls. DIBOA has recently been shown to increase under severe drought in bread wheat (Itam et al., 2020). Benzoxazinoids are well known for their role in plant defense against insects and pathogenic microorganisms (Makowska et al., 2015; Zhou et al., 2018) and they also affect the microbiome (Cotton et al., 2019). Because benzoxazinoids are potentially autotoxic, activity is controlled via two‐component defense systems, that is, reactivity is reduced by chemical modification, such as glycosylation, while simultaneously a reactivating enzyme is provided, for example, a glycosidase (Niculaes et al., 2018). It has been shown that DIMBOA and DIBOA glycosides are inactive (Hashimoto & Shudot, 1996; Larsen & Christensen, 2000). In Cadenza, DIMBOA glycoside is constitutively higher and, although diminished, remains higher under heat stress compared to Paragon. Glucosylation of DIMBOA appears to be important in this context with several genes encoding glucosidases and glucosyltransferases differentially regulated (see above). It thus appears that the benzoxazinoid pathway in wheat is heat responsive and finely regulated and it will be interesting to establish its role in abiotic stress tolerance in more detail.

Another molecule identified in this study, which was significantly higher in Cadenza compared to Paragon and less abundant under heat stress, is dhurrin (or dhurrin isomer). Like DIBOA/DIMBOA, dhurrin is regulated by a two‐component defense system to prevent autotoxicity (Bjarnholt et al., 2018). Dhurrin is a well‐known cyanogenic glucoside from sorghum and functions as a chemical defense against herbivores and pathogens by releasing toxic hydrogen cyanide. It is developmentally regulated and can reach toxic levels in young plants, as well as under drought and under highly fertilized conditions (Gleadow et al., 2015; Rosati et al., 2019; Sohail et al., 2022). It has been suggested that cyanogenic glucosides might also serve as an alternative N source and it has recently been shown that dhurrin spontaneously builds conjugates with glutathione, which then undergo reductive cleavage by glutathione transferases, eventually leading to formation of free ammonia by nitrilases (Bjarnholt et al., 2018). Given the high concentration of dhurrin under control conditions in Cadenza, it will be of interest to investigate its function in wheat as a putative alternate N source.

### 4.2 Phenolic metabolite profiles suggest an essential role in radical scavenging

Phenylpropanoids and flavonoids are vastly heterogeneous groups consisting of thousands of phenylalanine‐derived secondary metabolites with important functions throughout plant growth and development, as well as tolerance to biotic and abiotic stresses, including heavy metals, drought, salinity, nutrients, cold, and heat (di Ferdinando et al., 2012; Dwivedi et al., 2017; Sharma et al., 2019). The protective function of these molecules is mainly attributed to their ability to scavenge excessive radicals that form under stress, thereby preventing lipid peroxidation, oxidation of macromolecules (e.g., DNA, proteins), as well as damage to PSII. However, as is the case for DIBOA and dhurrin, phenylpropanoids can be toxic and it has been shown that glycosylation by UDP‐glycosyltransferases reduces toxicity, and modifies solubility, compartmentalization, and stability, while reducing antioxidant capacity (le Roy et al., 2016; Shahidi et al., 2022). Likewise, multiple methoxy groups on one phenolic ring might reduce reactivity and antioxidant capacity and are thus important regulatory functional groups (Jeevitha et al., 2017; Teponno et al., 2016).

Tri‐methoxy cinnamic acid is the most significantly increased metabolite in the phenylpropanoid pathway under heat stress, in both Cadenza and Paragon. Cinnamic acid is the first product of the general phenylpropanoid pathway catalyzed by the enzyme PAL (phenylalanine ammonium lyase) and the precursor for derived ferulic and coumaric acid. The role of the three methoxy groups is not entirely clear. As shown in an animal study, tri‐methoxy cinnamic acid had a strong protective role against gastric lesions in humans (Lee et al., 2017); however, their effect on cancer cell lines, and DPPH radical scavenging properties, is dependent on the nature of functional groups and the number of methoxy groups, as mentioned above (Ruwizhi & Aderibigbe, 2020; Takahashi & Kakehi, 2010). Although ferulic acid was less abundant under heat, it remained significantly higher in Cadenza, compared to Paragon, due to the high concentration under control conditions. Ferulic acid is, in its covalently conjugated form, an important component of cell walls, while in its free form it is shown to act as an antioxidant (Kumar & Pruthi, 2014). Likewise, syringic acid derivatives remained higher in Cadenza despite being less abundant under heat. Syringic acid has been shown to have a free‐radical scavenging function, attributed to the methoxy groups on the aromatic ring at positions 3 and 5 (Srinivasulu et al., 2018). Constitutive genotypic differences have also been reported from a study on drought, showing constitutively higher levels of chlorogenic acid, ferulic acid and other shikimate‐derived metabolites in the intolerant genotype and general reduction under stress (Guo et al., 2018).

Contrary to free ferulic acid, feruloyl‐coumaroyl‐glycerol conjugates increased under heat in both genotypes to about the same level. Radical scavenging and antioxidant activity of the conjugate has been shown in extracts of *Tulipa systole*, a herbal medicine from Iraq (Ibrahim et al., 2017). An increase in this conjugate might therefore provide effective protection against ROS damage. This is in support of a study in rice, which identified 4‐hydroxycinnamic acid and ferulic acid as key metabolites related to the higher level of drought tolerance in a tolerant genotype (IAC1246), which also maintained a higher level of photosynthesis and antioxidant capacity (Ma et al., 2016). An increase in caffeic acid and ferulic acid has also been reported from a study in festuca and this was specific to the tolerant genotype in response to a short term (7 h) but not long‐term (21 h) heat stress (Wang et al., 2019). Similarly, differences in metabolic responses to cyclic versus prolonged drought stress has been shown in poplar, with the former mainly affecting primary metabolites, whereas the latter induced mainly secondary metabolites (populosides; Tschaplinski et al. 2019), which agrees with the response to chronic heat stress reported here.

In a detailed study in tomato, plants were exposed to heat and salt stress and a combination of the two stresses (Martinez et al., 2016). The study showed a differential accumulation of phenylpropanoids and flavonoids, with the latter specifically increased under heat stress and associated with greater protection from oxidative damage. Flavonoids are known to be powerful antioxidants, especially dihydroxy‐B‐ring substituted flavonoids, such as caffeic acid, tricetin, or luteolin (Agati et al., 2012). It was shown in maize that higher drought tolerance in a mutant (*doi*) was related to higher total flavonoid content and ROS scavenging capacity compared to the B73 wild‐type control (Li et al., 2021). Constitutive genotypic difference in flavonoids have also been shown in *Arabidopsis* and rice (Hu et al., 2014; Routaboul et al., 2012) and the role of flavonoids as antioxidants under drought stress have been shown in wheat (Ma et al., 2014) as well as by transgenic approaches in *Arabidopsis* (Nakabayashi et al., 2014; Rao et al., 2020) and apple (Geng et al., 2020).

In our study, we found a large increase of an unspecified, glycosylated flavonoid compound that was highly significantly upregulated under heat stress. Interestingly, while three derivatives of this compound were increased in both analyzed genotypes, other derivatives were highly specific to Cadenza and Paragon, respectively. Other compounds that increased under heat stress included molecules derived from tricin, tricitin, and chryseriol. As was the case for the phenylpropanoids, some flavonoid compounds were less abundant under heat stress but showed constitutive genotypic differences with an apparent overall higher abundance in Cadenza.

However, despite these marked genotypic differences, DPPH assay data from two independent experiments revealed an overall slightly higher ROS scavenging capacity in Paragon, as indicated by a lower IC_50_ value. This suggests that chemical radical scavenging is well developed in both genotypes and that tolerance in Cadenza might thus be related to other mechanisms.

### 4.3 Differential expression of stress‐response genes

The RNAseq analysis revealed a range of genes with known protective functions under stress, but there was little overlap with the heat‐responsive metabolic pathways described above. In Cadenza, candidate genes encode a peroxidase and an aldehyde dehydrogenase, which detoxify aldehydes originating from lipid hydroperoxides (Kotchoni et al., 2006; Sunkar et al., 2003). In addition, a large number of glutathione *S*‐transferases (GSTs) were upregulated in both genotypes. GSTs detoxify electrophilic compounds by catalyzing the nucleophilic conjugation of GSH (γ‐Glu‐Cys‐Gly) and have been implicated in many stress responses (Jain et al., 2010; Nutricati et al., 2006; Sappl et al., 2009; Sharma et al., 2014; Skopelitou et al., 2017). Surprisingly, thioredoxins, which also have been implicated with a range of protective functions under stress (Delaunay et al., 2002; Zhai et al., 2022), were downregulated under heat stress in both genotypes.

Cell membranes are particularly sensitive to high‐temperature stress because increased kinetic energy loosens chemical bonds leading to increased fluidity, and therefore permeability (Niu & Xiang, 2018). Membranes are also sensitive to ROS and lipid peroxidation is a key indicator of heat stress (Jiang & Huang, 2001). As a result of these effects, electrolytes can be lost, and the membrane is unable to perform its required function, which can ultimately lead to cell death (Narayanan et al., 2015, 2018; Narayanan, Prasad, et al., 2016; Narayanan, Tamura, et al., 2016). Our RNAseq analysis revealed differential expression and positive heat‐response in Cadenza of several genes encoding lipid‐related proteins, including a lipase GDSL domain‐containing protein and a non‐specific lipid‐transfer protein (nsLTP) 2G. Both GELPs and nsLTPs play crucial roles in plant growth and development (Ma et al., 2018; Watkins et al., 2019), and have demonstrated roles in biotic and abiotic stresses (Kim et al., 2013; Naranjo et al., 2006). There is evidence that both GELPs and nsLTPs play a role in male reproductive development, which is known to be particularly sensitive to heat stress (Huang et al., 2013; Wan et al., 2020; Zhao et al., 2020). Interestingly, nsLTPs also have a role in protecting thylakoid membranes during freezing (Hincha et al., 2001, 2002; Sror et al., 2003). This and the abovementioned identification of the anti‐freeze compound propanediol suggests commonalities between heat and cold stress responses.

Another major problem under heat stress is the degradation of proteins and, as a protective measure, plants upregulate HSPs to facilitate re‐folding and re‐solubilization of denatured proteins and protein aggregates (Bourgine & Guihur, 2021; Kumar et al., 2020). In the RNAseq study, six HSP70 genes were identified, with a general trend toward downregulation in Cadenza and upregulation in Paragon. However, two of these genes showed constitutively lower expression in Paragon, despite the heat‐induced upregulation. One of these encodes *BINDING IMMUNOGLOBULIN PROTEIN* (*BiP*), one of the major chaperones in the ER lumen (Pobre et al., 2019). In the ER, heat stress induces the so‐called unfolded protein response (UPR) (Angelos et al., 2017; Buchberger et al., 2010; Liu & Howell, 2010; Read & Schröder, 2021) which is a protective pathway increasing the ER's protein folding capacity. BiP‐encoding genes have been shown to be upregulated by the UPR and play a protective role during drought and osmotic stress (Alvim et al., 2001; Carvalho et al., 2014; Valente et al., 2009), possibly by preventing endogenous oxidative stress (Alvim et al., 2001). Constitutively higher expression of this gene in Cadenza could contribute to the observed tolerance to heat stress in this study.

HSP70s work in conjunction with small heat shock proteins (sHSPs) to protect their thermosensitive substrates (Bourgine & Guihur, 2021; Waters & Vierling, 2020). Expression of a sHSP was consistently higher in Cadenza but increased in Paragon under HT. The stress‐responsive and protective functions of these chaperones may contribute to the heat tolerance seen in Cadenza. Two HSP80s and four HSP100 genes were also upregulated in both cultivars under heat, as were several peptidylprolyl isomerase, which are known to interact with HSPs to regulate protein biosynthesis and refolding of proline‐containing proteins (Kaur et al., 2015; Kurek et al., 1999). Peptidylprolyl isomerases, specifically AtFKBP6/ROF1, are also part of a well‐studied heat response in which FKBP interacts with HSP90 and, in a heat‐dependent manner, with the heat shock transcription factor HSFA2A. Nuclear translocation of this complex then enables the HSFA2A‐dependent transcription of sHSP genes (Meiri & Breiman, 2009). An FKBP gene has previously also been identified as a candidate in a study on heat tolerance in rice (González‐Schain et al., 2016).

Another important chaperone family are dehydrins, which are shown to stabilize membranes (Eriksson et al., 2016) and prevent protein aggregation and/or inactivation under stress (Close, 1997; Park et al., 2006; Qin & Qin, 2016; Yu et al., 2018). Dehydrins also act as radical‐scavengers due to their high content of histidine, lysine, and glycine, which are targets for radical‐mediated oxidation (Drira et al., 2013; Hara et al., 2004, 2005; Yang et al., 2015). Two dehydrin genes, annotated as *RAB15* and *RAB16B*, showed increased expression under heat in Cadenza, and the dehydrin gene *CS120* remained stable in Cadenza while decreasing under heat in Paragon. CS120 has been implicated in membrane protection during drought and cold stress (Chu et al., 2021). Interestingly, one of the heat‐induced genes in Cadenza codes for a fructan exohydrolase (FEH). Fructans function as both short‐term storage polysaccharides and in stabilizing membranes during freezing and drought, acting as signaling molecules, and exerting an antioxidant effect (van den Ende, 2013).

Taken together, the nature of the identified DEGs suggest that Cadenza might have a greater capacity to prevent protein degradation, membrane damage and the ER UPR response.

Several other genes involved in effective protein synthesis, processing, ubiquitination, and degradation were among the differentially expressed genes. These include an F‐box domain containing protein, an aldo_ket_red domain‐containing protein, and an AA‐trans domain containing protein. This suggests a different response or capacity for coping with protein mis‐folding. A cysteine protease, known to be responsive to environmental cues (Morrell & Sadanandom, 2019) and play a part in oxidative stress‐induced programmed cell death (Solomon et al., 1999) showed constitutively higher expression in Cadenza. Also higher in Cadenza were ankyrin repeat (ANK) genes which have essential roles in plant development and have been shown to respond to heat and cold stress (Eun et al., 2007; Yang et al., 2008). Overexpression of *ANK genes* have been shown to mitigate the effects of drought (Yang et al., 2008) and oxidative stress (Seong et al., 2007) in *Artemesia desertorum* and capsicum, respectively. Despite evidence of the role of members of this protein family in response to stress, their roles in heat stress have not been well characterized, with no information available for their activity in wheat and they would therefore be novel targets for further investigation into heat stress tolerance in this crop.

Other relevant genes include Xyloglucan endotransglucosylase/hydrolase (XTHs) and Polysaccharide synthesis 4 (PS4) domain‐containing protein, both involved in cell wall biogenesis and remodeling. XTHs correlate with plant growth (Osato et al., 2006; van Sandt et al., 2007) and decrease in expression in response to heat, as observed in Paragon, has also been shown in other wheat cultivars (Iurlaro et al., 2016). Maintenance of expression of these genes under stress in Cadenza might thus be a factor contributing to its superior growth and tillering capacity under stress.

When the RNAseq data were mined for known tillering‐related genes, such as the *TIN* genes, *TaMOC1*, and *TaTB1* (Shang et al., 2021), no differential expression was found in response to heat treatment or genotype at any time of day or developmental stage. Some genes related to flowering showed genotype‐dependent expression. For example, several MADS‐box transcription factors were differentially expressed (one higher in Cadenza and two higher in Paragon) but none displayed a temperature response. Similarly, genes encoding *EARLY FLOWERING 3* and *FLOWERING‐PROMOTING FACTOR 1 PROTEIN 1‐LIKE* were both more highly expressed constitutively in Paragon than Cadenza but did not respond to high temperature. Other flowering‐related genes, such as *VRN1*, *VRN2*, *BM3*, *BM8*, and *FT1* (Shimada et al., 2009), did not appear in our gene lists. This could be due to the tissue sampled, however, as *TaMOC1*, for example, is expressed in the epidermal cells of leaf primordia and is subsequently expressed in axillary buds, SAM, and young leaves (Shang et al., 2021), but it is unclear whether expression would be expected in mature leaves, as sampled in this study.

Two hormone‐related genes with demonstrated roles in plant development, *ABSCISIC STRESS‐RIPENING PROTEIN* 5 (*ASR5*) and *CYTOKININ DEHYDROGENASE* 3, showed higher expression in Cadenza. ASR proteins have been implicated in heat and cold stress responses, and they enhance drought tolerance in *Arabidopsis* by upregulating ABA/stress‐regulated genes and acting as chaperone‐like proteins (Golan et al., 2014; Sah et al., 2016; Yacoubi et al., 2022). ABA has been shown to have a role in regulating tillering; daily application of ABA to young wheat plants leads to increased tiller and leaf numbers (Hall & Mcwha, 1981), while low ABA concentrations are associated with reduced assimilate transfer from vegetative organs to grain (Wang et al., 2016). Cytokinins have been similarly implicated in abiotic and biotic stress responses, as well as having a role in shoot and root growth, grain development, senescence, and mineral acquisition (Cortleven et al., 2019; Han et al., 2014). Higher cytokinin levels lead to development of more reproductive organs, and thus a higher yield (Yamburenko et al., 2017). Exogenous application of cytokinin has been found to enhance heat stress tolerance by slowing leaf senescence and inhibiting heat‐induced lipid peroxidation of cell membranes (Liu & Huang, 2002). Cytokinins are degraded by cytokinin dehydrogenases (Chen et al., 2020; Jameson & Song, 2016) and mutants expressing less cytokinin dehydrogenase develop larger panicles in rice (Li et al., 2011). It therefore appears that downregulation of a cytokinin dehydrogenase in Cadenza might enable maintenance of higher levels of cytokinins, which could confer protection against heat stress, ultimately leading to the higher final seed number we observed for this cultivar.

### 4.4 Cadenza maintains photosynthesis under heat stress

One of the key findings of this study was that Cadenza maintained photosynthetic rate under heat stress, while it was significantly reduced in Paragon. This has implications for the resources available for vegetative and reproductive growth and could explain differences in tillering and yield characteristics of the two cultivars under heat stress.

Maintenance of photosynthesis has previously been identified as a key component of heat tolerance (Sharma et al., 2015). Thylakoid membranes, electron carriers, and enzymes, particularly those of PSII are thermosensitive (Moore et al., 2021; Salvucci & Crafts‐Brandner, 2004a; Sharkey, 2005). High temperatures lead to an excess of chloroplastic reducing equivalent, accumulation of ROS, and photoinhibition (Hu et al., 2020; Wang et al., 2017). The above‐described differences in the chemical and non‐chemical radical scavenging capacity, as well as differential expression of genes encoding membrane‐protective proteins, between the genotypes might therefore be important factors protecting PSII.

Of the photosynthesis‐related heat‐responsive DEGs, a similar expression was observed in Cadenza and Paragon. While most of these genes were downregulated under heat, the three homeologs of *RuBisCo activase* were all upregulated. *Rubisco activase* is essential for the maintenance of the carboxylation reaction but is particularly sensitive to heat (Law & Crafts‐Brandner, 1999; Ristic et al., 2009; Salvucci & Crafts‐Brandner, 2004b). In agreement with our data, it has recently been shown that the *Rca1* splice variant *Rca1β* shows increased transcript abundance, as well as protein level under high temperature stress (Degen et al., 2021), suggesting an important role in heat protection. Interestingly, it was recently shown that a single amino acid substitution increased thermotolerance and activity under heat *in vitro* (Degen et al., 2020). Furthermore, it has been shown that overexpression of both *Rubisco* and *Rubisco activase* increases photosynthesis under heat stress in rice (Qu et al., 2021).

However, since *Rca1* was increased in both analyzed genotypes, this does not explain the observed genotypic differences in photosynthetic thermotolerance, which could instead be related to the photosynthesis‐associated genes that showed constitutively different expression. Of particular interest in this context are genes with higher expression in Cadenza, encoding for example, a PsbP domain‐containing protein, *PROTEIN LOW PSII ACCUMULATION 3* (*LPA3*), and an unspecified Rieske domain‐containing protein, as well as a RubisCo small subunit *RbcS* gene. PsbP‐like proteins are involved in the assembly of PSII, and it was shown in *Arabidopsis* that PsbPs optimize the water‐oxidizing reaction and are required for the efficient repair of photodamaged PSII (Che et al., 2020). Likewise, LPA3 has been implicated in PSII repair (Theis & Schroda, 2016). Rieske proteins are Fe‐S proteins, and as a subunit of Cytb6f, an essential component of PSII electron transport. Overexpression of the Brachipodium *PetC* gene in Setaria has recently been shown to enhance C4 photosynthesis (Ermakova et al., 2019).

Wheat *RbcS* genes constitute a gene family with at least 25 members, classified based on sequence similarity into groups 1‐3 (Degen et al., 2021). In agreement with our data, none of the groups is apparently heat responsive (Degen et al., 2021), suggesting that a Cadenza‐specific constitutive high expression of a group 2 *RbcS* gene, as well as the other genes mentioned above, rather than heat‐induced increase in expression, might be relevant for tolerance.

## 5 CONCLUSIONS

This study reveals genotypic differences in vegetative heat tolerance within UK spring wheat and highlights the importance of secondary metabolites for stress resilience, due to their protective role via chemical radical scavenging. The identification of propanediol as a novel, highly heat‐induced compound warrants further investigation and suggests communalities between heat and cold responses. The gene expression data confirmed the general role of heat‐induced chaperones and ROS scavenging pathways and further suggests that constitutive genotypic differences might be important for stress tolerance. Maintenance of photosynthesis in Cadenza under heat has been identified as a key component of tolerance and future work will establish whether this is related to the differentially expressed photosynthesis‐related genes or any other Cadenza‐specific heat‐responsive genes with putative protective functions.

## FUNDING INFORMATION

This project was supported by the RCUK project BB/P027970/1 “Transforming India's Green Revolution by Research and Empowerment for Sustainable food Supplies (TIGR2ESS) and the Designing Future Wheat” (DFW) Strategic Programme (BB/P016855/1). Further support was provided from the UKRI project “Safeguarding Sonora's wheat from climate change” (BB/S012885/1). SH is also supported by the National Institute of Agricultural Botany (NIAB), Cambridge, UK.

## CONFLICT OF INTEREST

The authors declare that there is no conflict of interest.

## Supporting information


Table S1.

Table S2.

Table S3.

Figure S1.

Figure S2.

Figure S3.

## Data Availability

The data that support the findings will be available in the European Nucleotide Archive (ENA) under accession number PRJEB36237 and unique name ena‐STUDY‐ROTHAMSTED RESEARCH‐15‐01‐2020‐14:13:41:981‐1984 following an embargo from the date of publication.
