# Supplementary figures and images for: Novel molecules and target genes for vegetative heat tolerance in wheat
Source: Plant Environ Interact. 2022 Dec 26;3(6):264–89. doi: 10.1002/pei3.10096 (PMC10168084; doi:10.1002/pei3.10096)

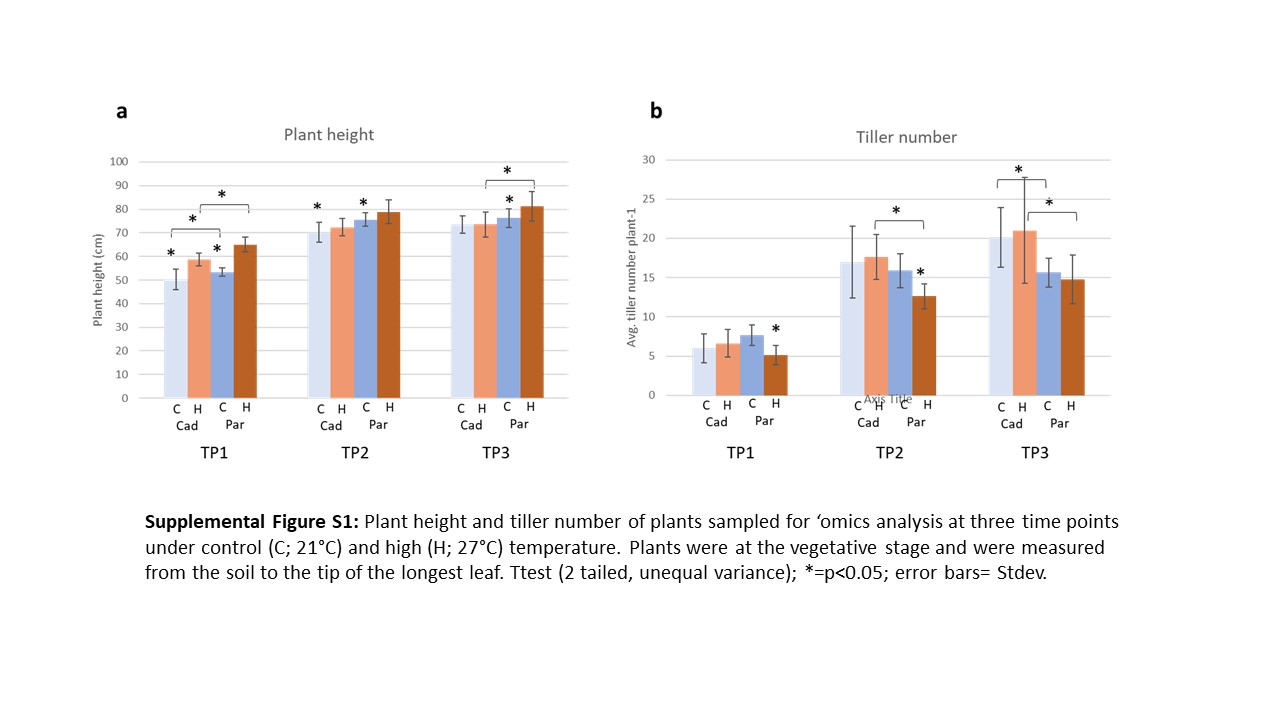

Supplement: Supplementary file 4 — Figure S1. [file PEI3-3-264-s006.jpg]
