# Supplementary material for: Novel molecules and target genes for vegetative heat tolerance in wheat
Source: Plant Environ Interact. 2022 Dec 26;3(6):264–89. doi: 10.1002/pei3.10096 (PMC10168084; doi:10.1002/pei3.10096)
Supplement: Supplementary file 5 — Figure S2. [file PEI3-3-264-s003.pdf]

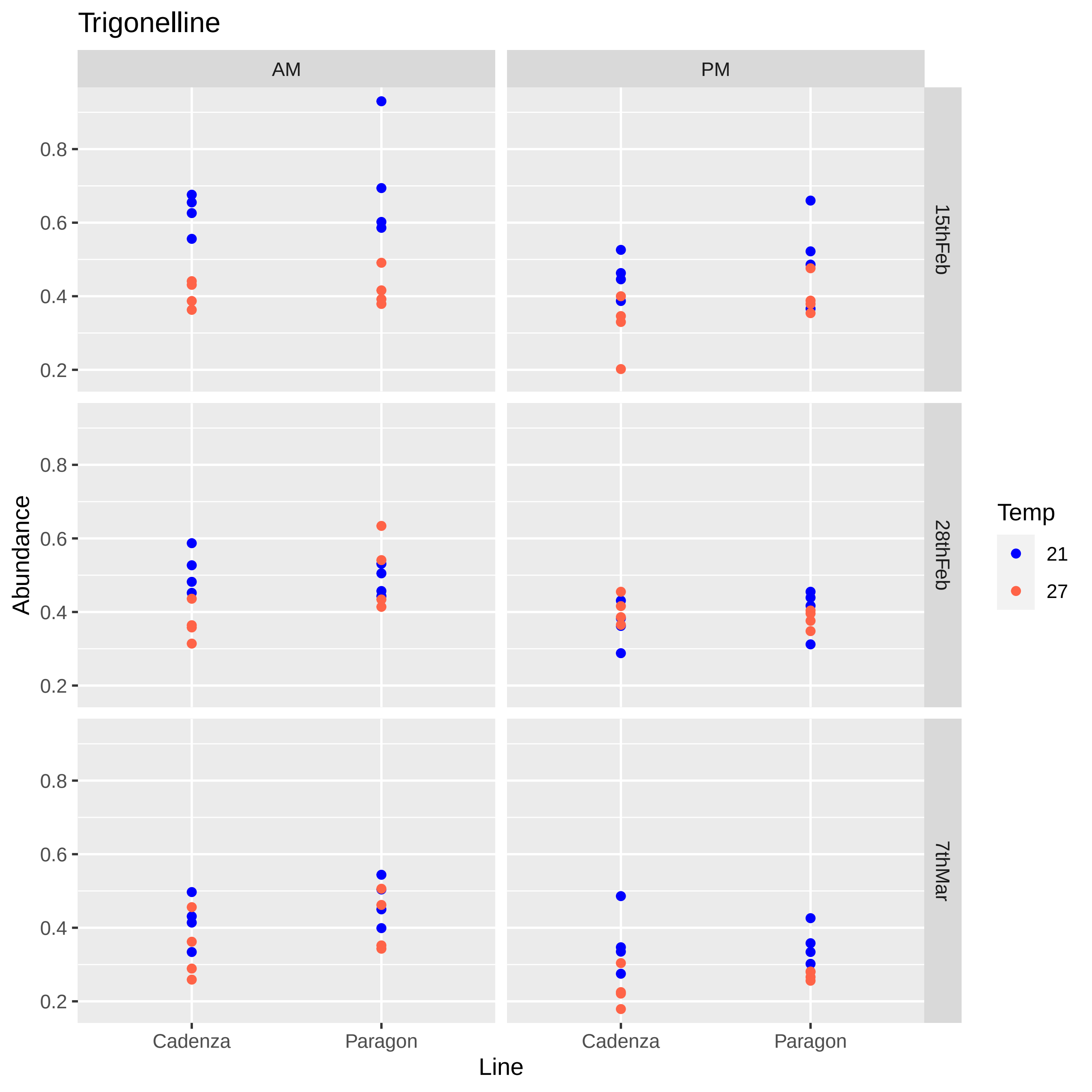

Trigonelline
AM
PM
15thFeb
28thFeb
7thMar
0.8
0.6
0.4
0.2
Abundance
Cadenza
Paragon
Line
Temp
21
27

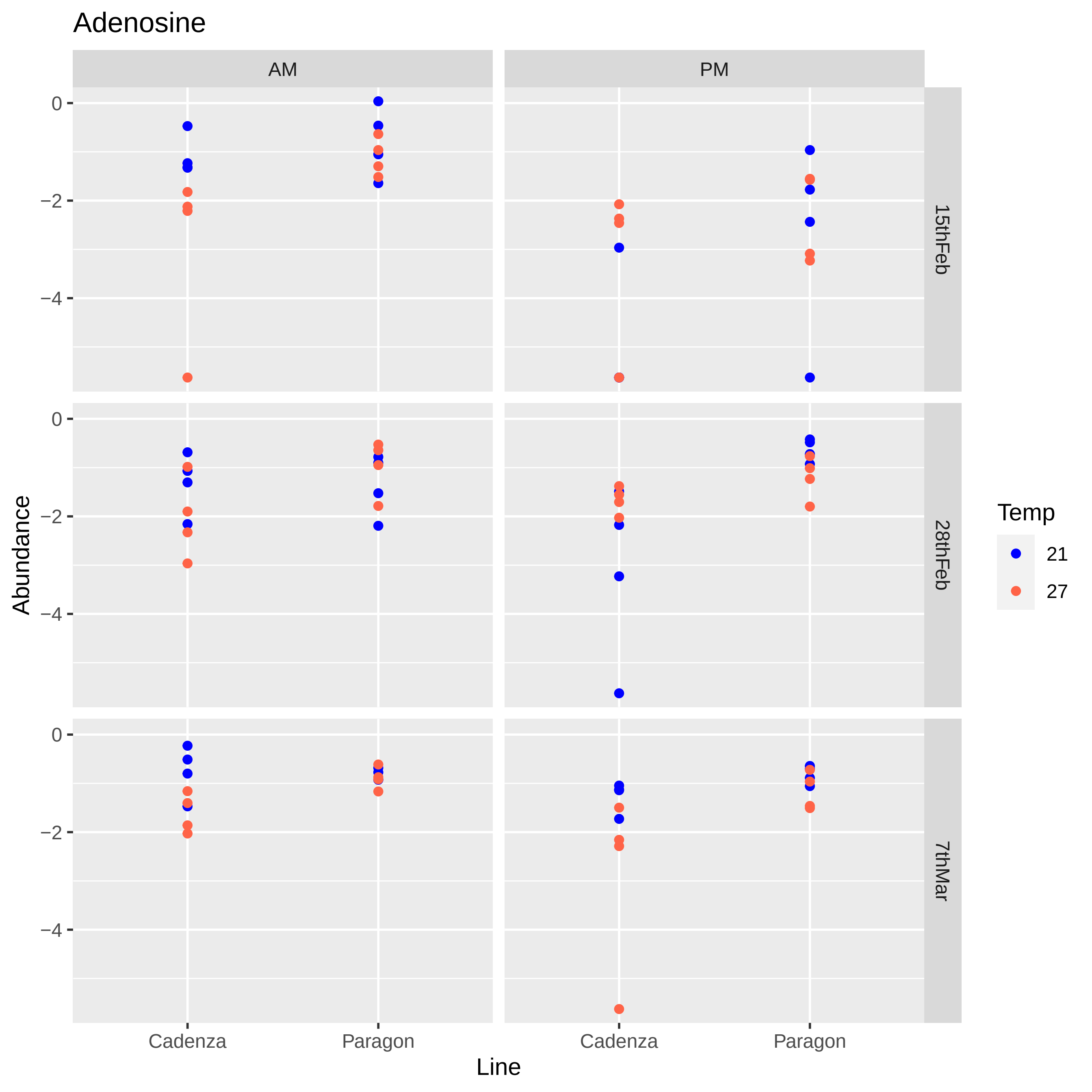

Adenosine
AM
PM
15thFeb
28thFeb
7thMar
Abundance
0
−2
−4
Cadenza
Paragon
Line
Temp
21
27

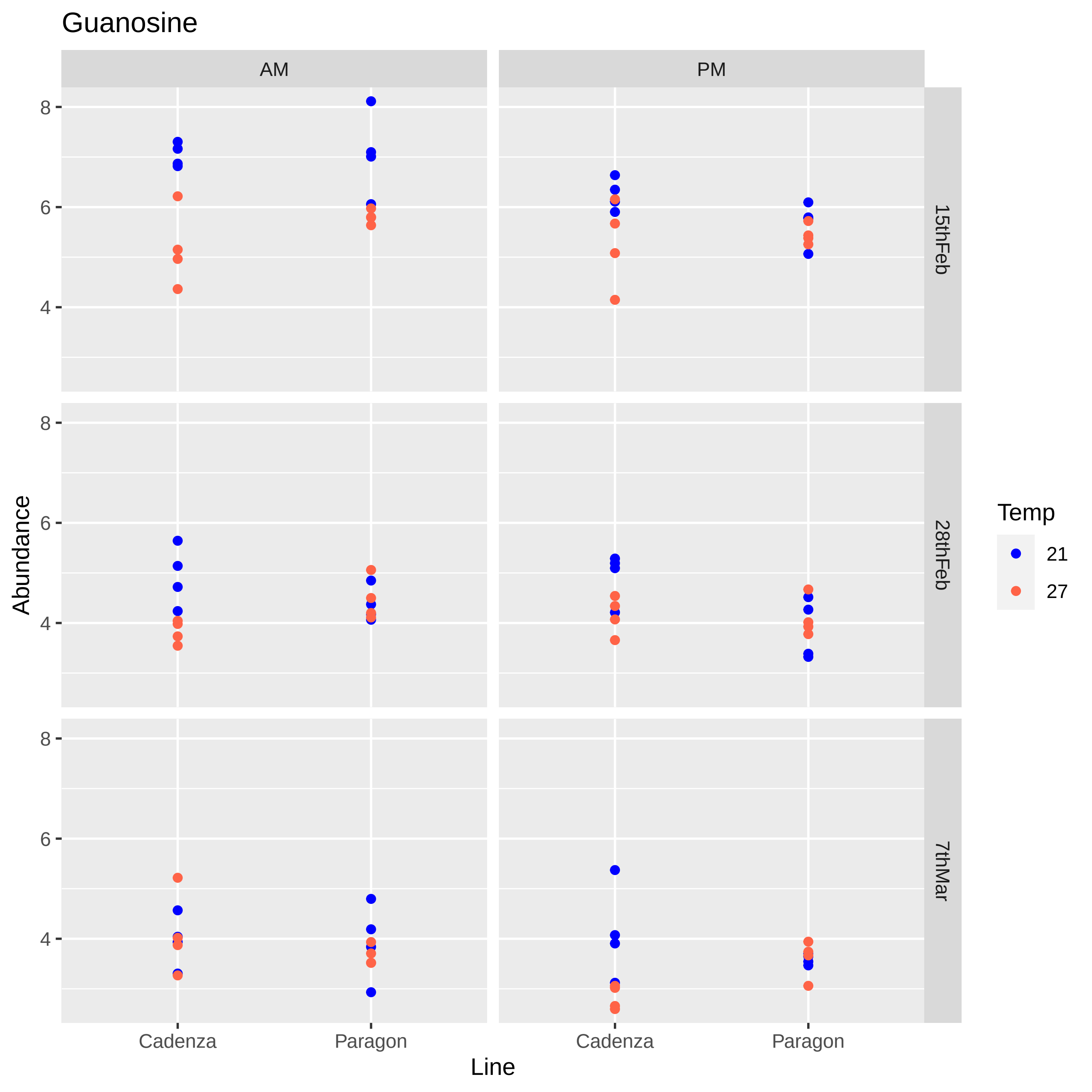

Guanosine
AM
PM
15thFeb
28thFeb
7thMar
Abundance
8
6
4
Cadenza
Paragon
Line
Temp
21
27

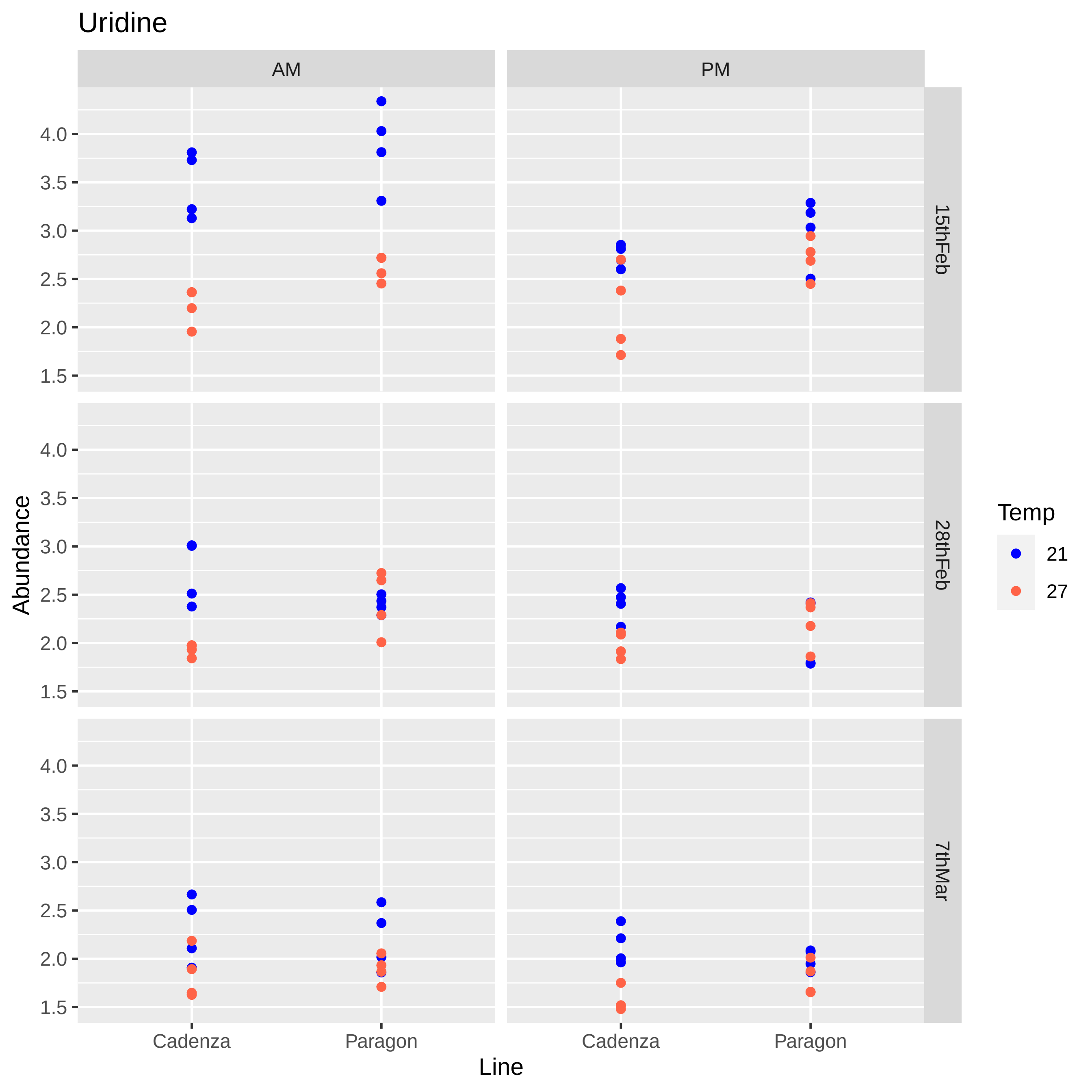

Uridine
AM
PM
15thFeb
28thFeb
7thMar
Abundance
4.0
3.5
3.0
2.5
2.0
1.5
Cadenza
Paragon
Line
Temp
21
27

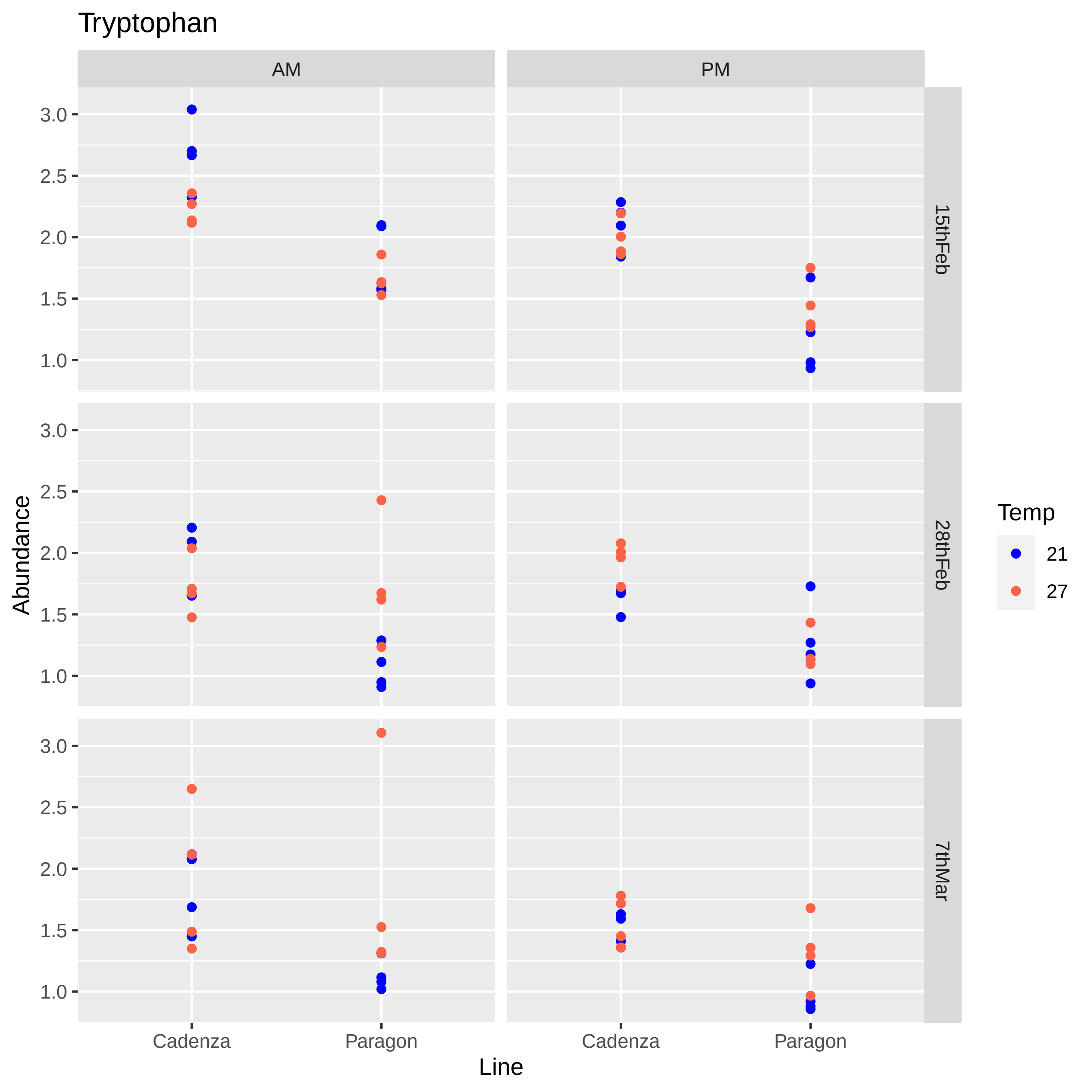
Tryptophan
AM
PM
15thFeb
28thFeb
7thMar
3.0
2.5
2.0
1.5
1.0
Abundance
Cadenza
Paragon
Line
Temp
21
27

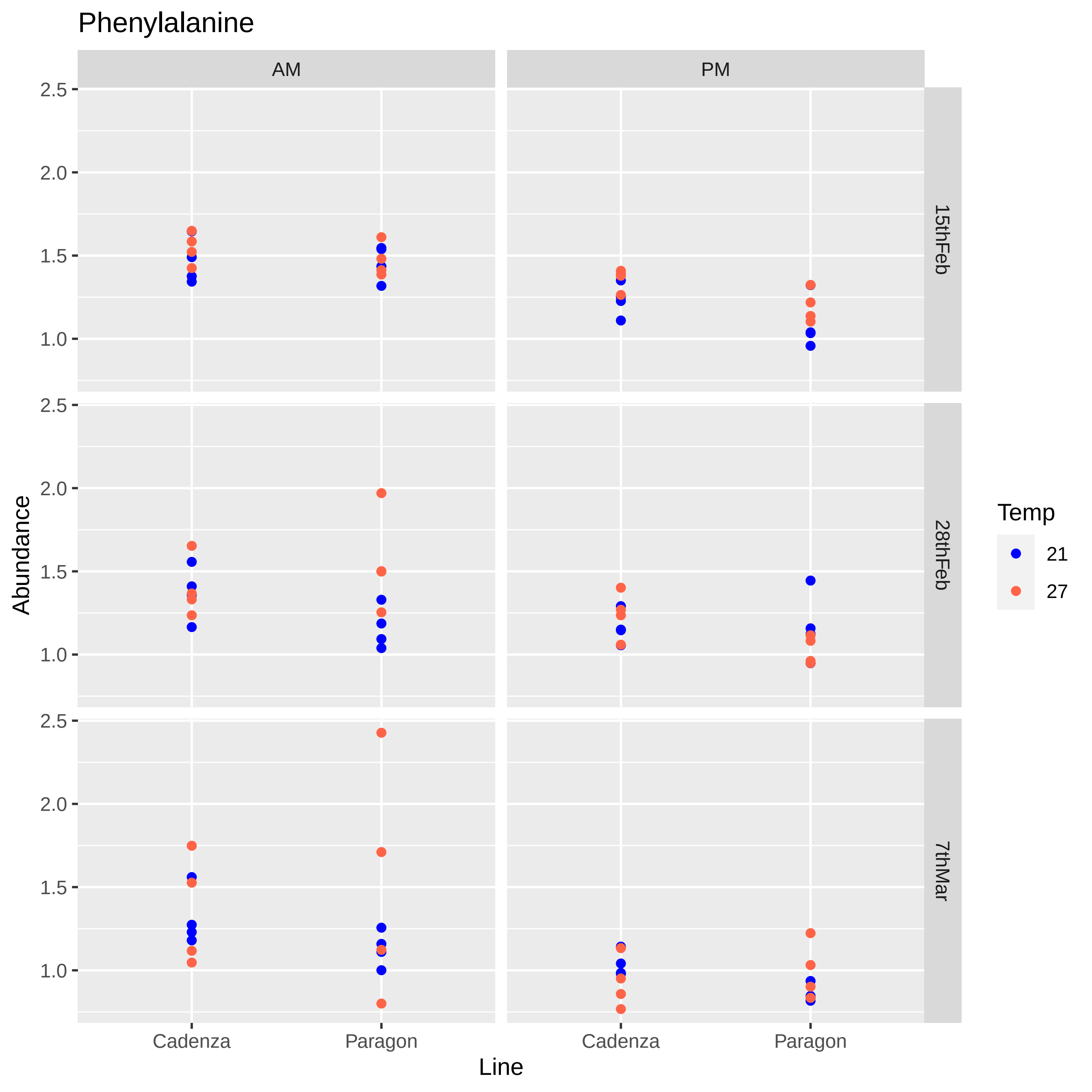
Phenylalanine
AM
PM
15thFeb
28thFeb
7thMar
Abundance
2.5
2.0
1.5
1.0
Cadenza
Paragon
Line
Temp
21
27

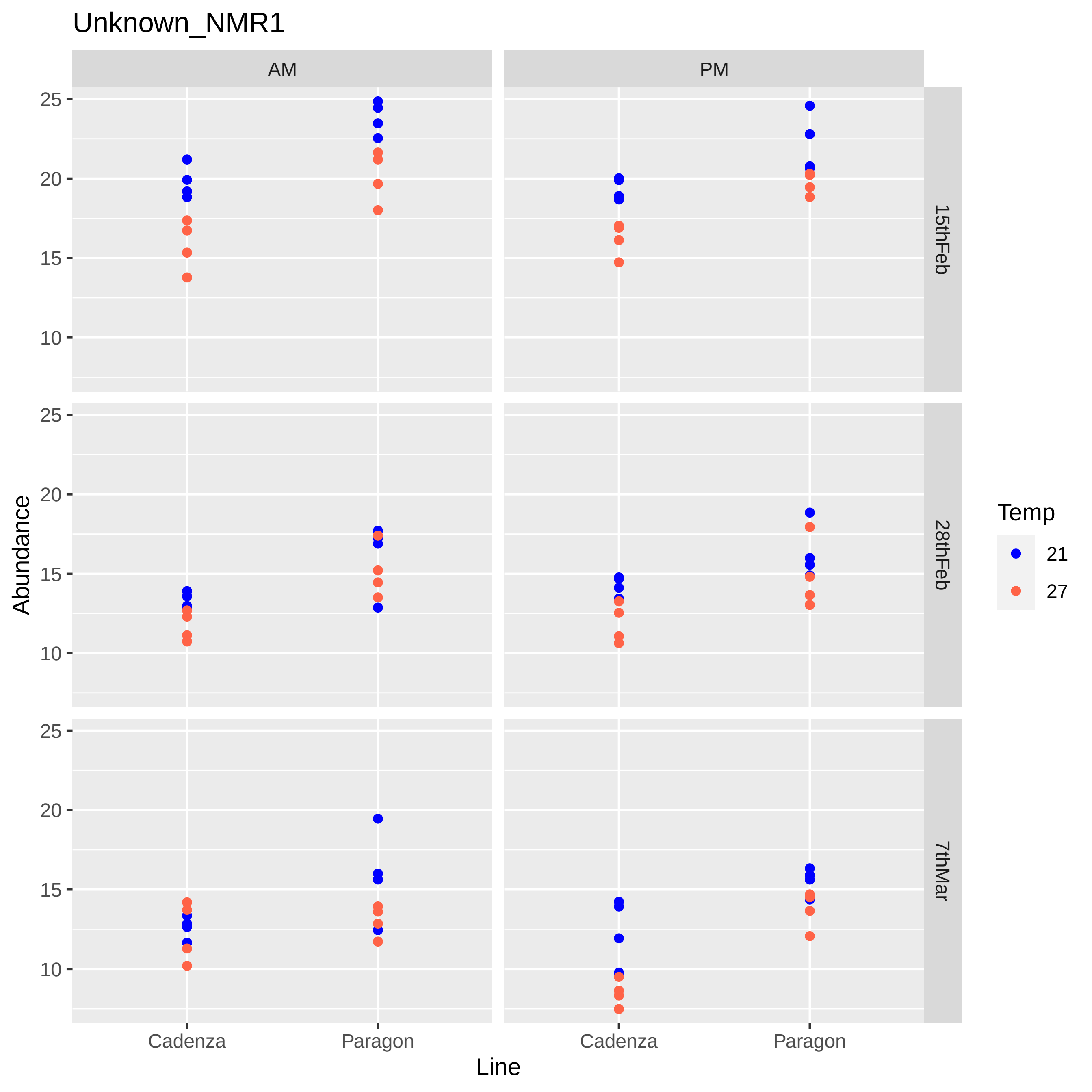

Unknown_NMR1
AM
PM
15thFeb
28thFeb
7thMar
Abundance
25
20
15
10
Cadenza
Paragon
Line
Temp
21
27

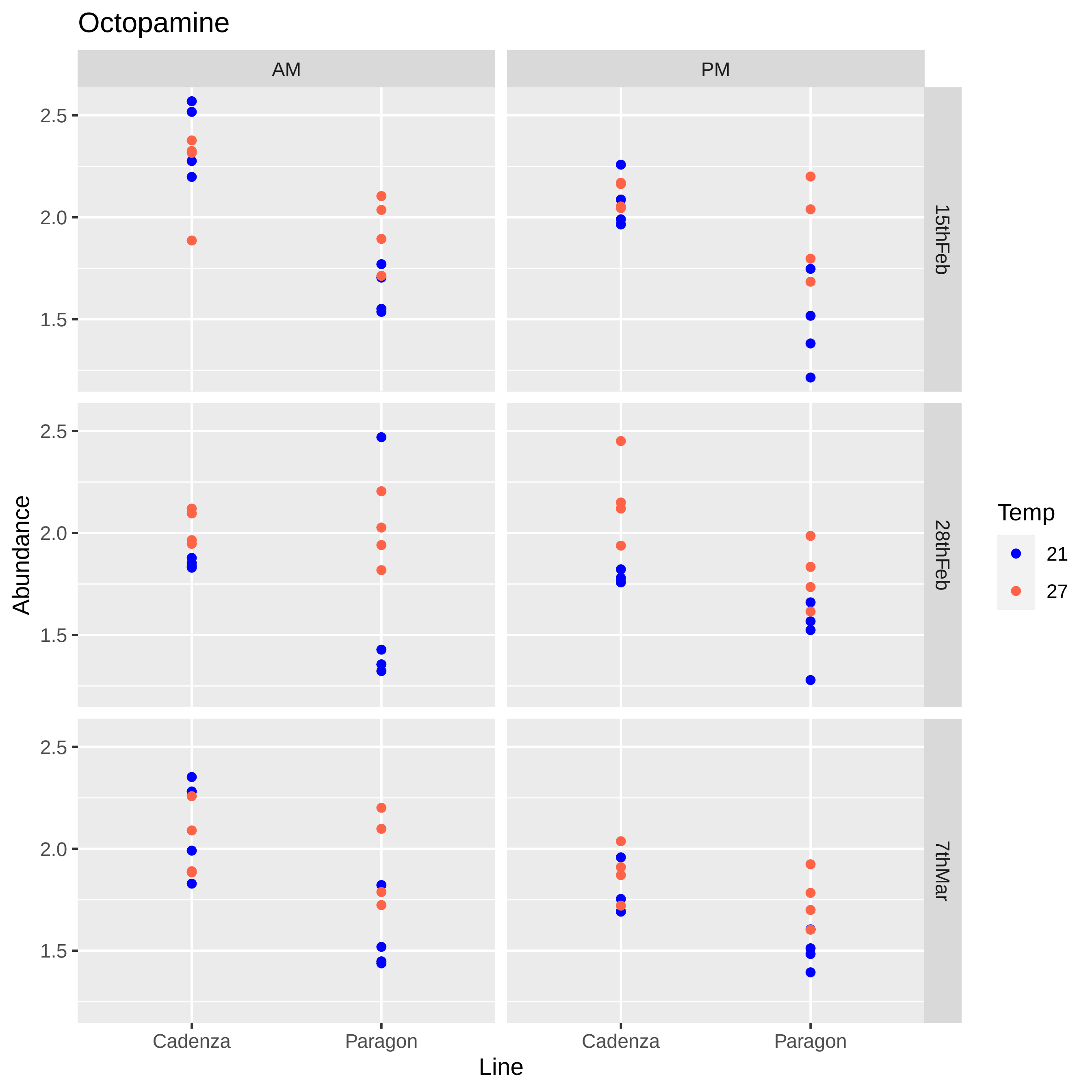
Octopamine
AM
PM
15thFeb
28thFeb
7thMar
Abundance
2.5
2.0
1.5
Cadenza
Paragon
Line
Temp
21
27

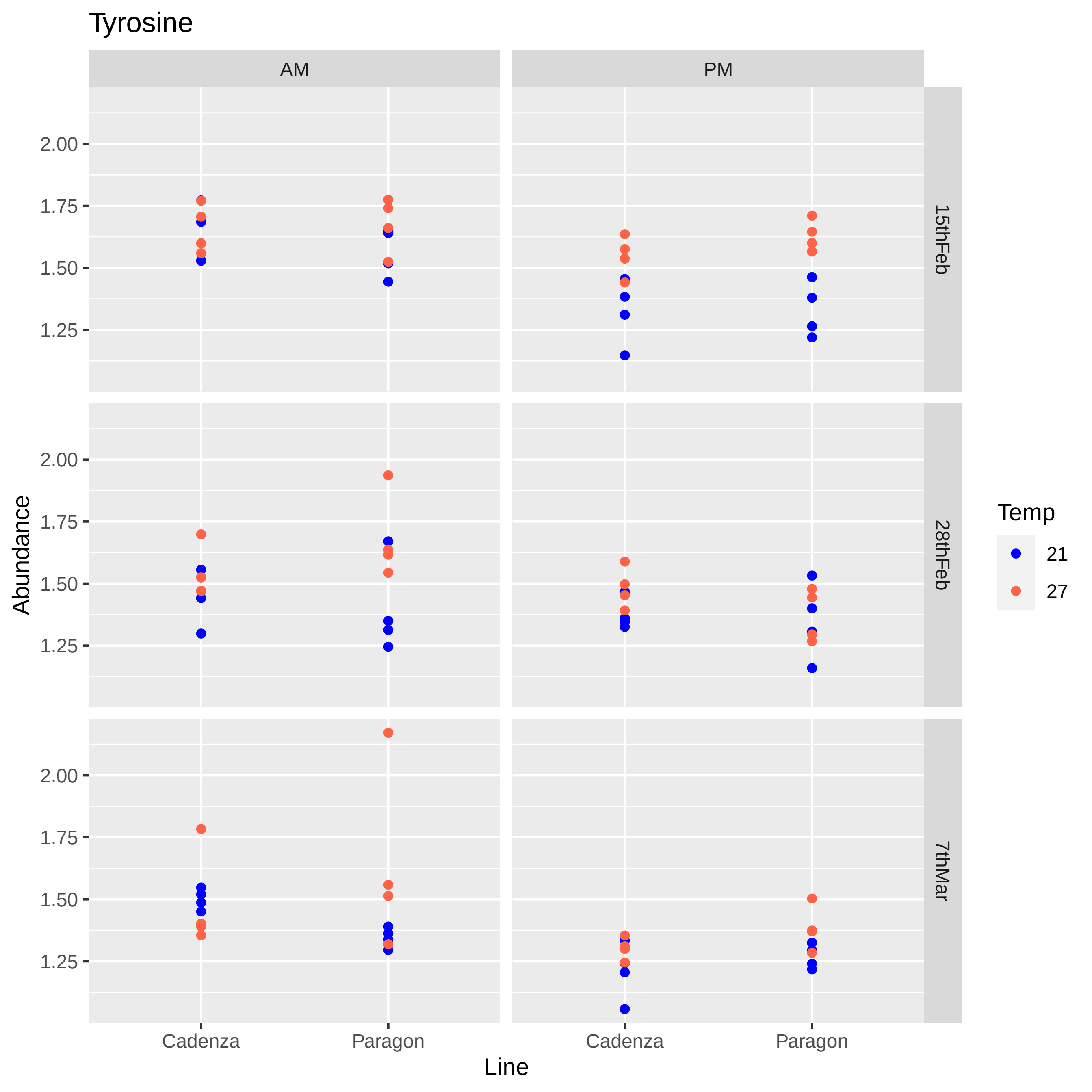

Tyrosine
AM
PM
15thFeb
28thFeb
7thMar
2.00
1.75
1.50
1.25
Abundance
Cadenza
Paragon
Line
Temp
21
27

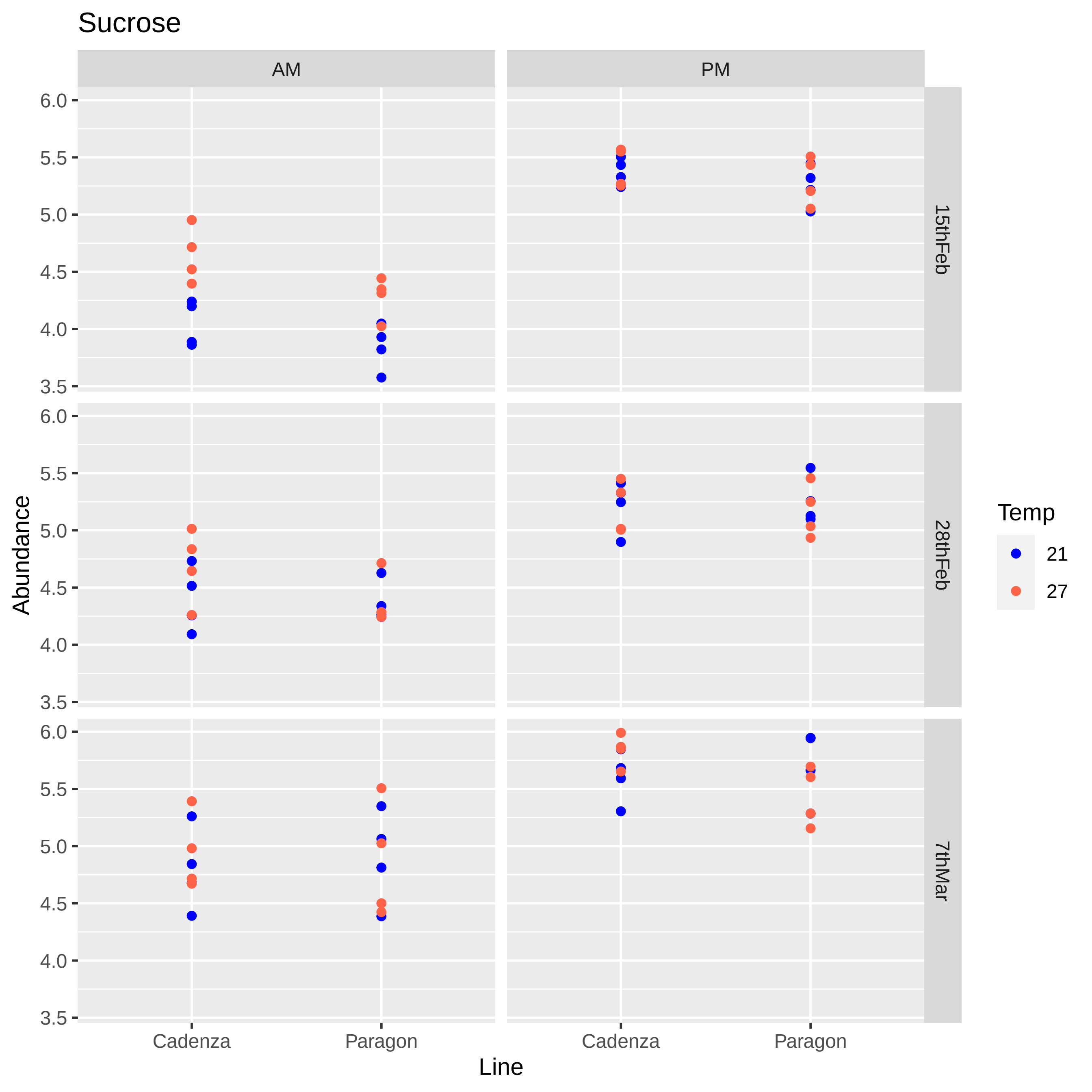
Sucrose
AM
PM
15thFeb
28thFeb
7thMar
Abundance
6.0
5.5
5.0
4.5
4.0
3.5
Cadenza
Paragon
Line
Temp
21
27

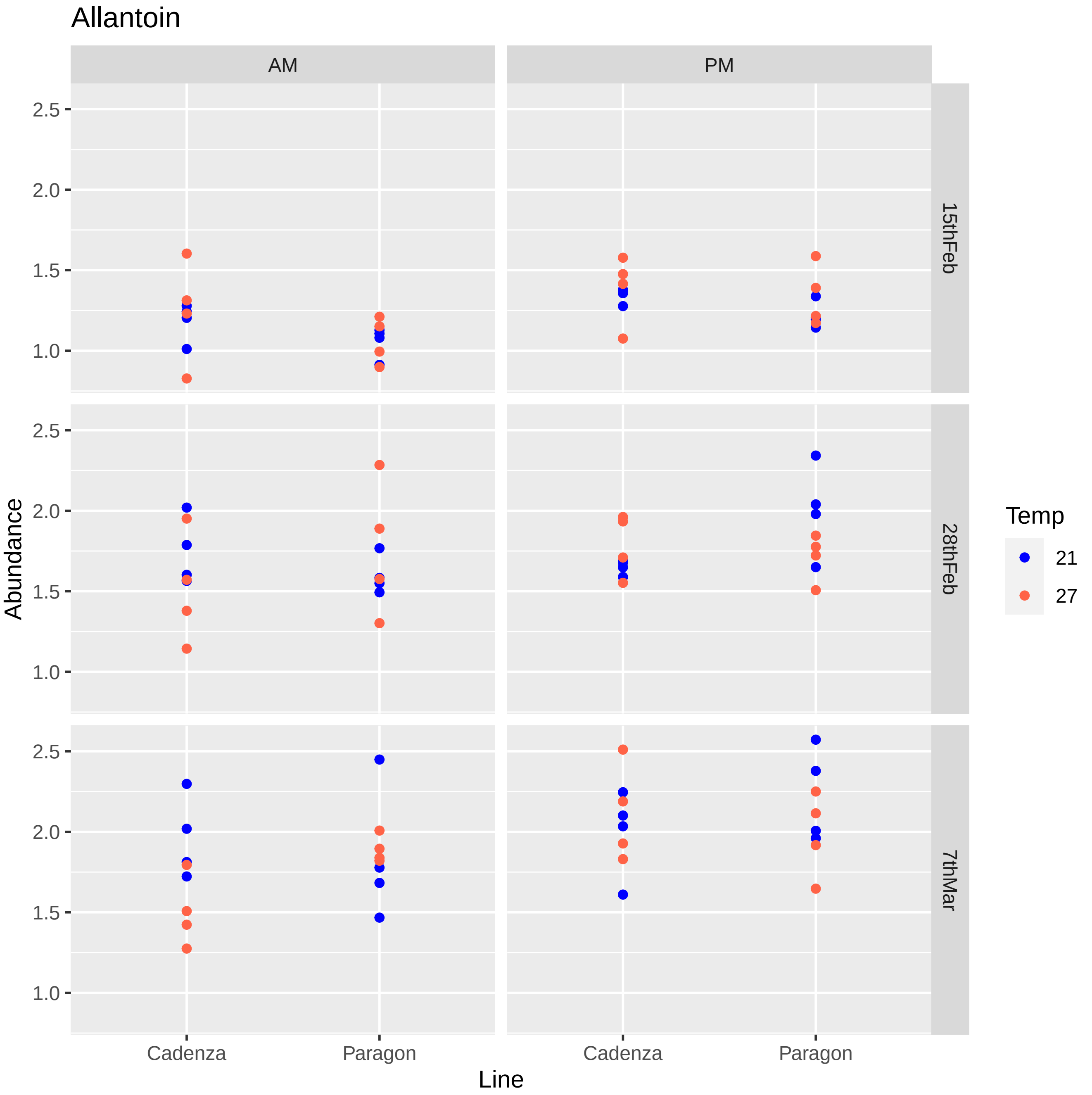

Allantoin
AM
PM
15thFeb
28thFeb
7thMar
Abundance
2.5
2.0
1.5
1.0
Cadenza
Paragon
Line
Temp
21
27

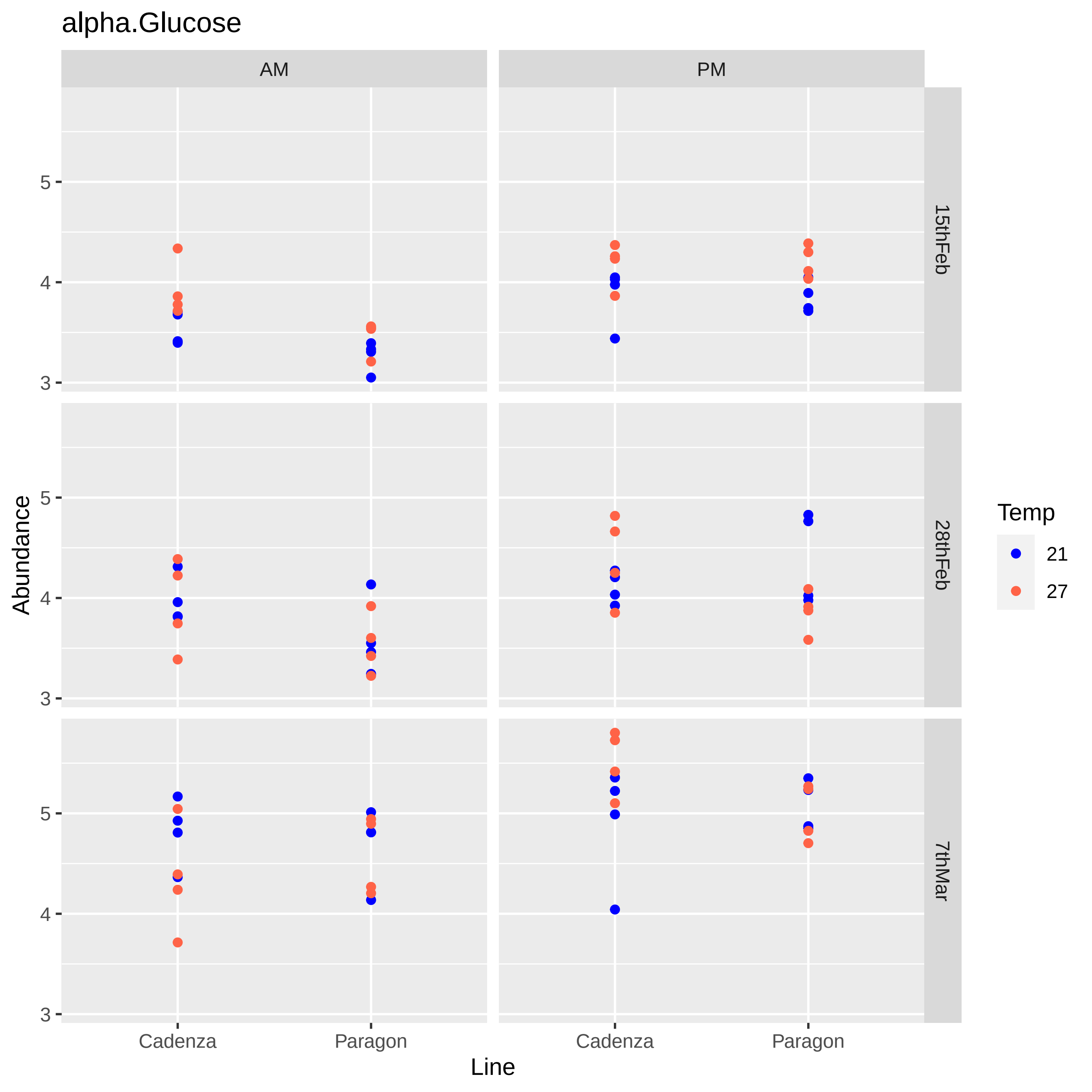

alpha.Glucose
AM
PM
15thFeb
28thFeb
7thMar
Abundance
5
4
3
Cadenza
Paragon
Line
Temp
21
27

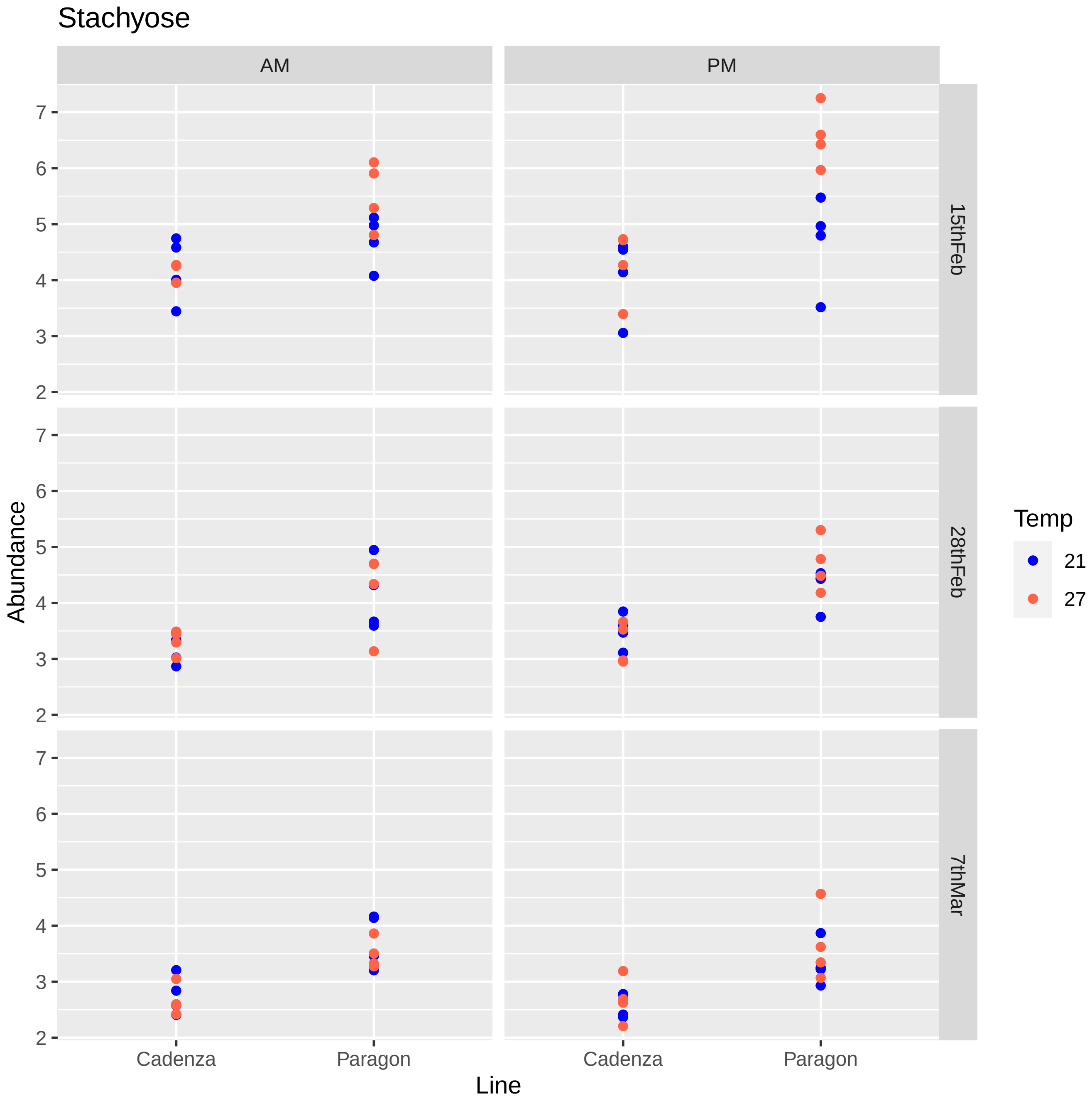
Stachyose
AM
PM
15thFeb
28thFeb
7thMar
Abundance
2
3
4
5
6
7
Cadenza
Paragon
Line
Temp
21
27

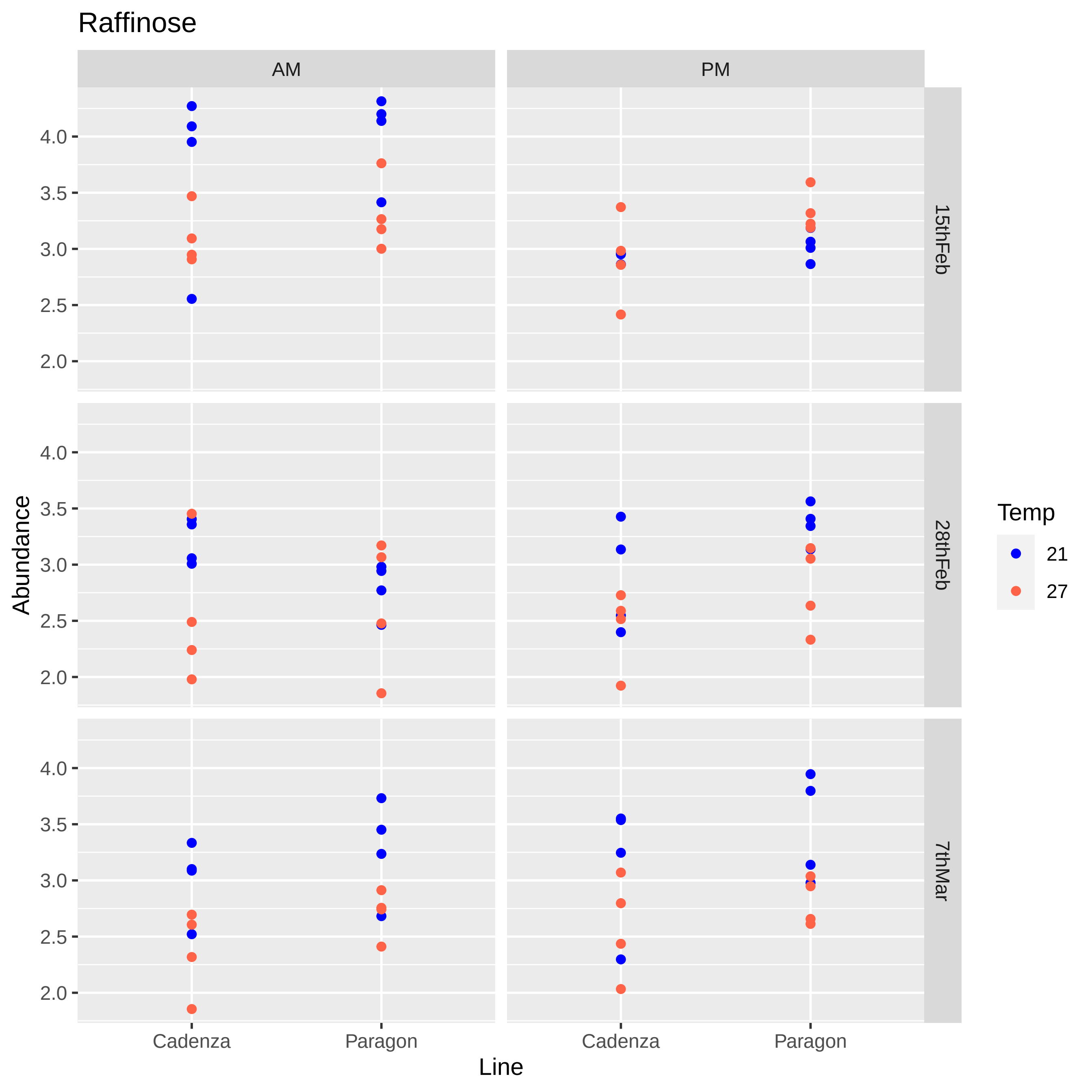

Raffinose
AM
PM
15thFeb
28thFeb
7thMar
Abundance
4.0
3.5
3.0
2.5
2.0
Cadenza
Paragon
Line
Temp
21
27

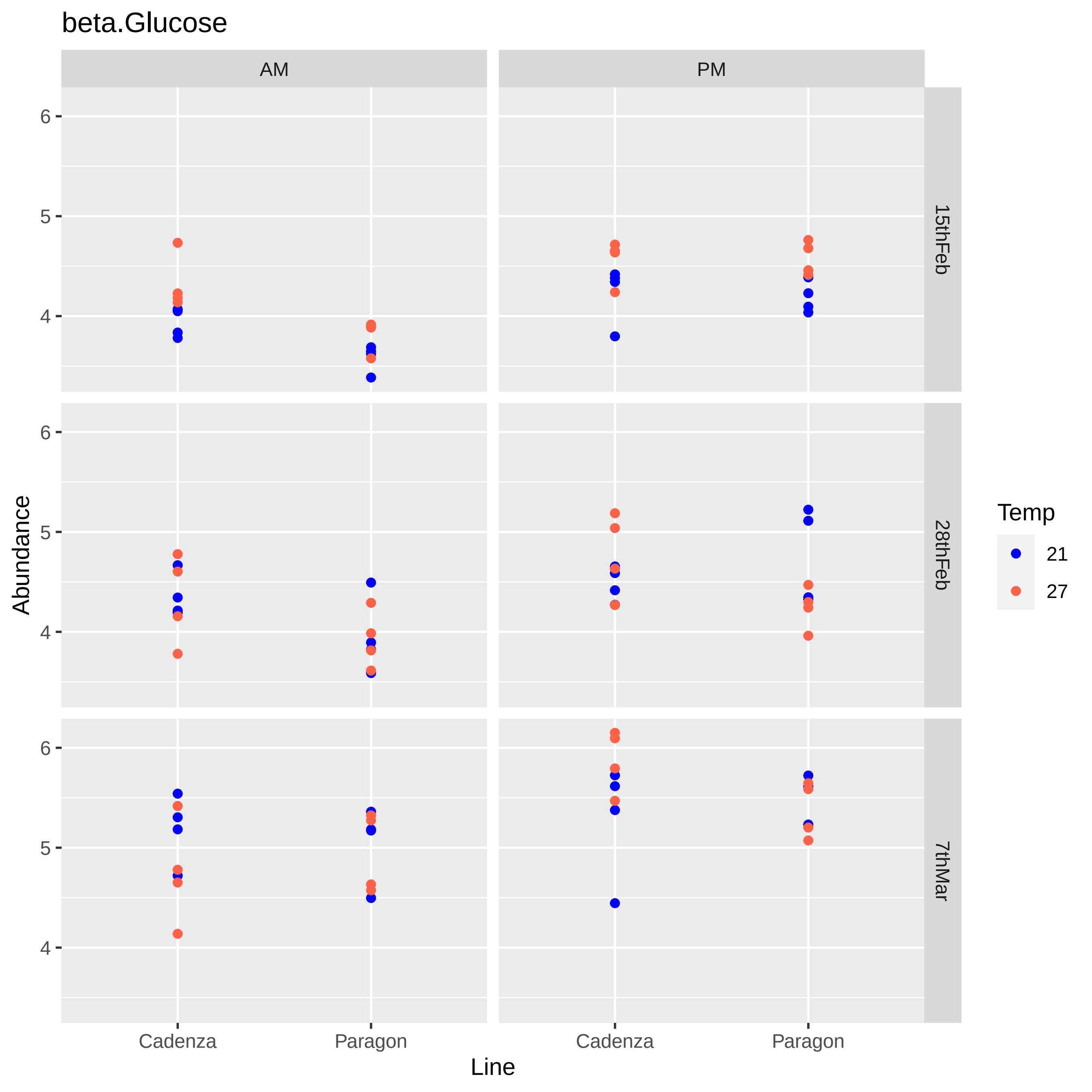

beta.Glucose
AM
PM
15thFeb
28thFeb
7thMar
Abundance
4
5
6
Cadenza
Paragon
Line
Temp
21
27

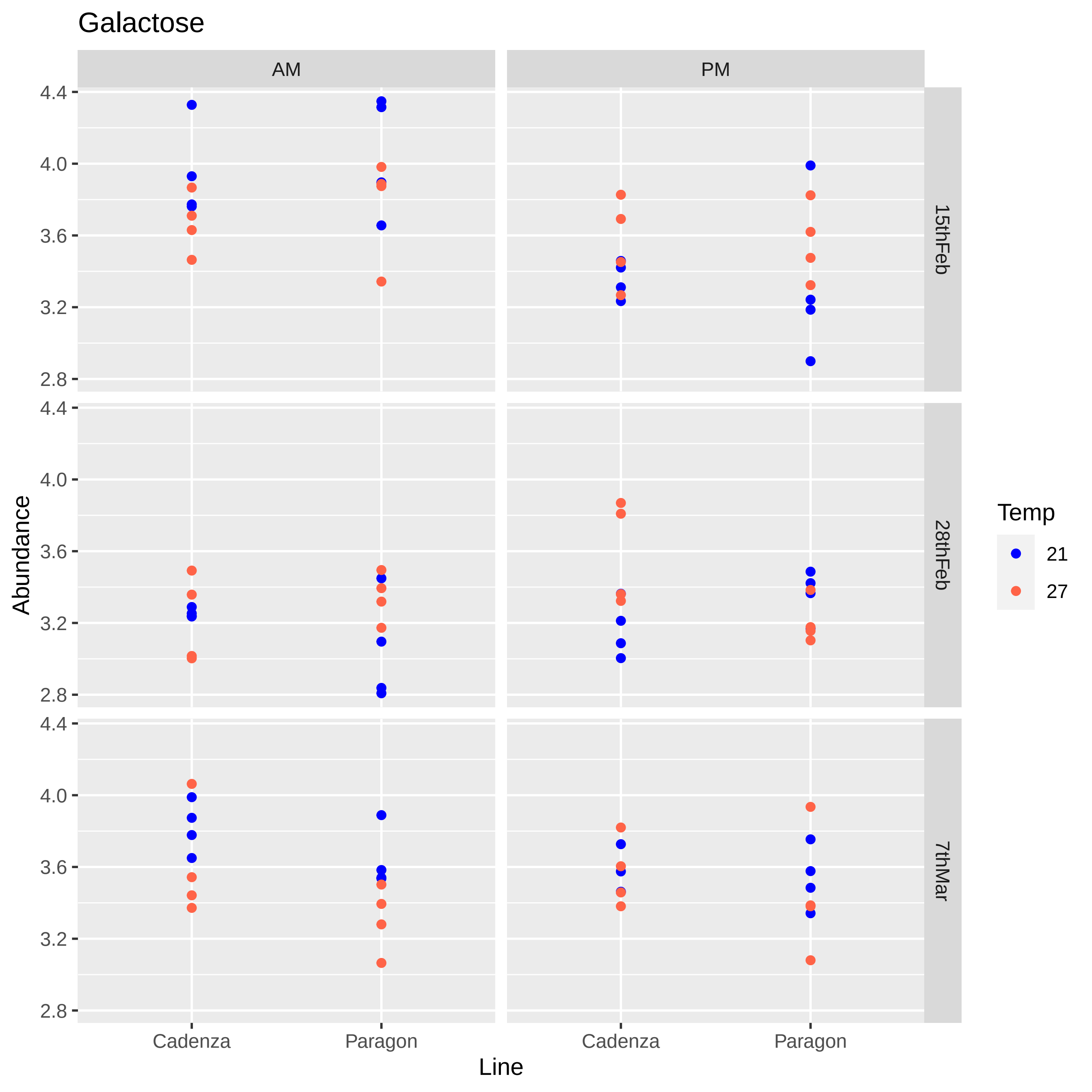
Galactose
AM
PM
15thFeb
28thFeb
7thMar
Abundance
4.4
4.0
3.6
3.2
2.8
Cadenza
Paragon
Line
Temp
21
27

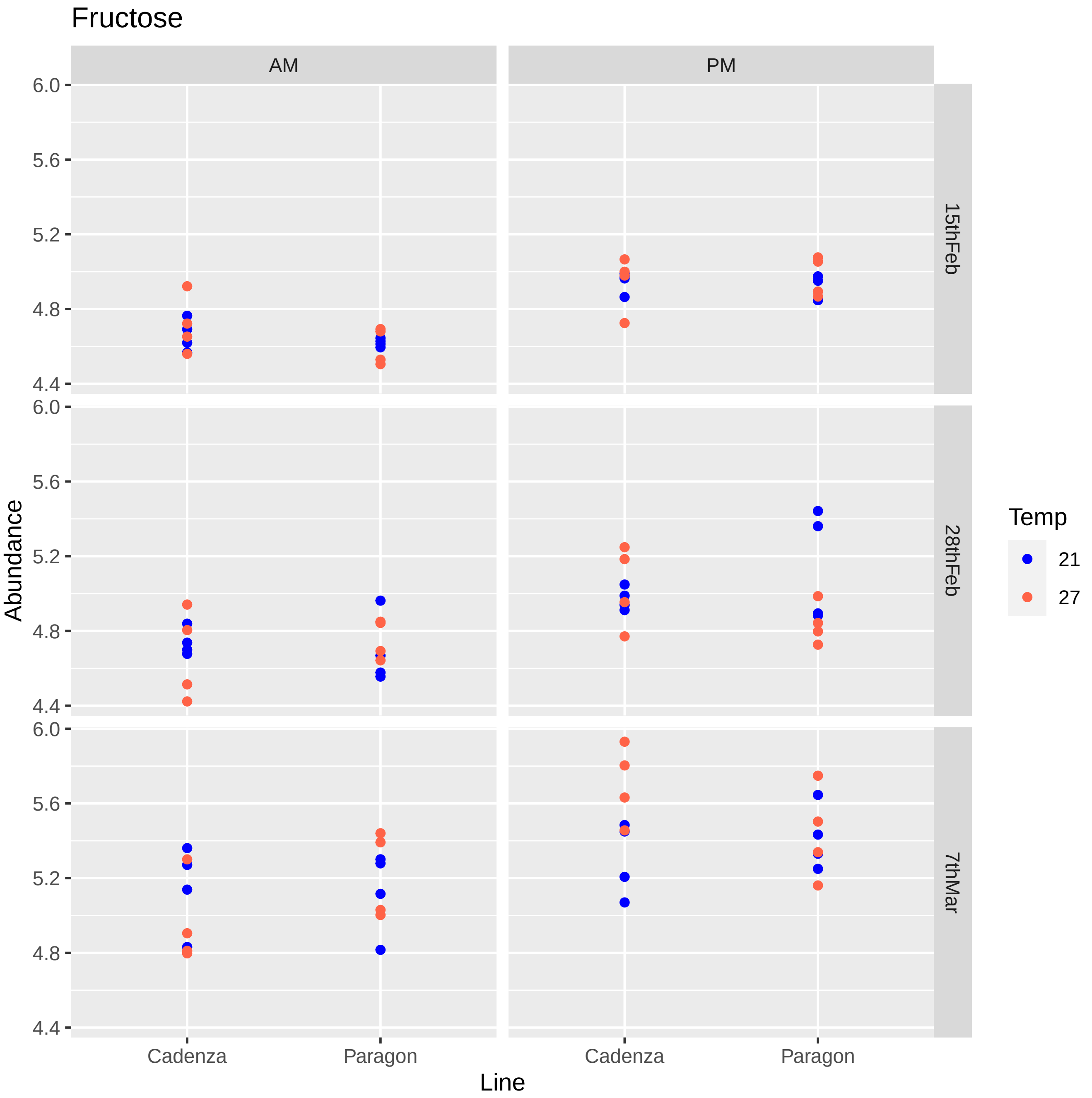

Fructose
AM
PM
15thFeb
28thFeb
7thMar
Abundance
Line
Cadenza
Paragon
Cadenza
Paragon
6.0
5.6
5.2
4.8
4.4
Temp
21
27

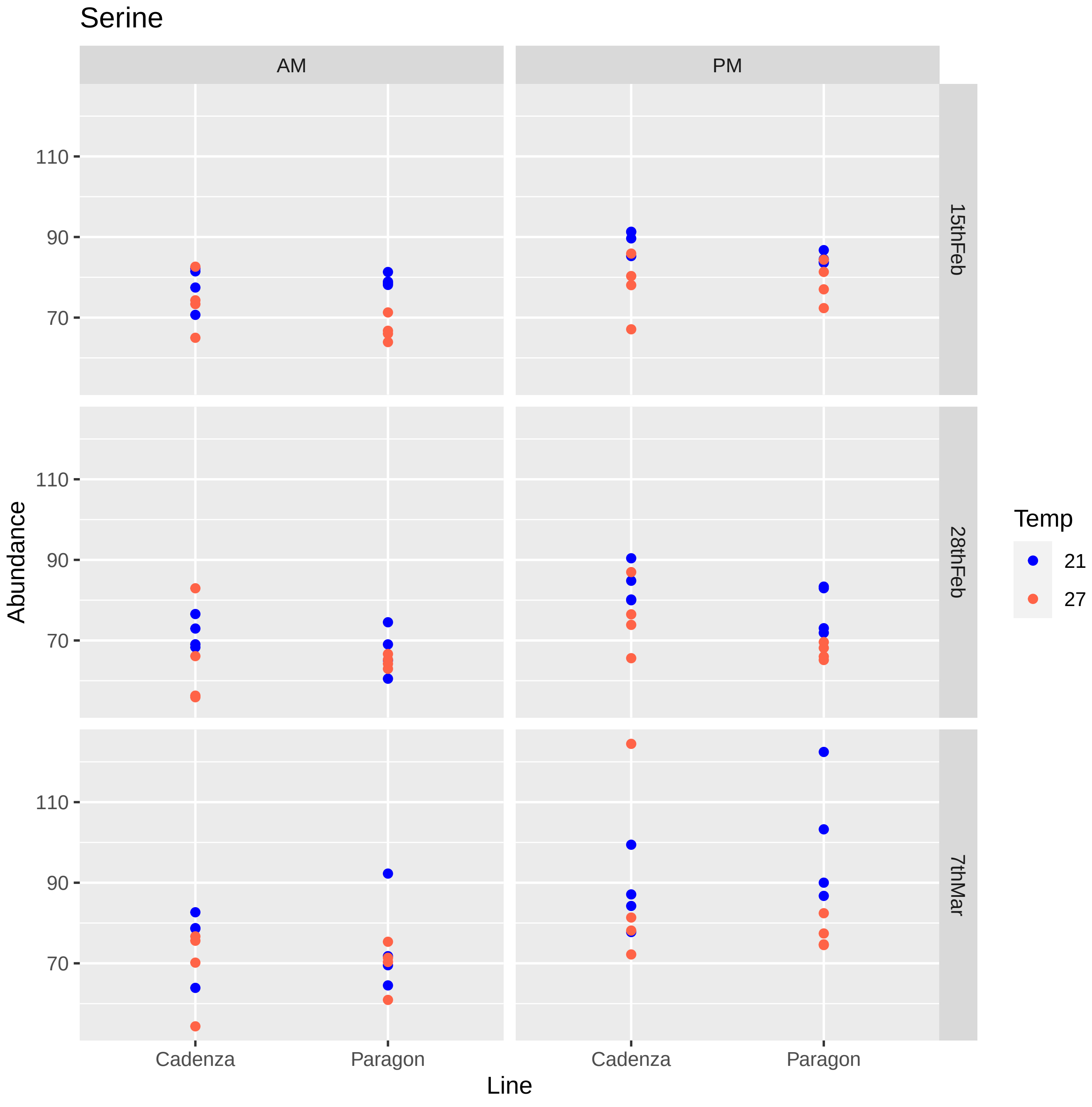

Serine
AM
PM
15thFeb
28thFeb
7thMar
110
90
70
Abundance
Cadenza
Paragon
Line
Temp
21
27

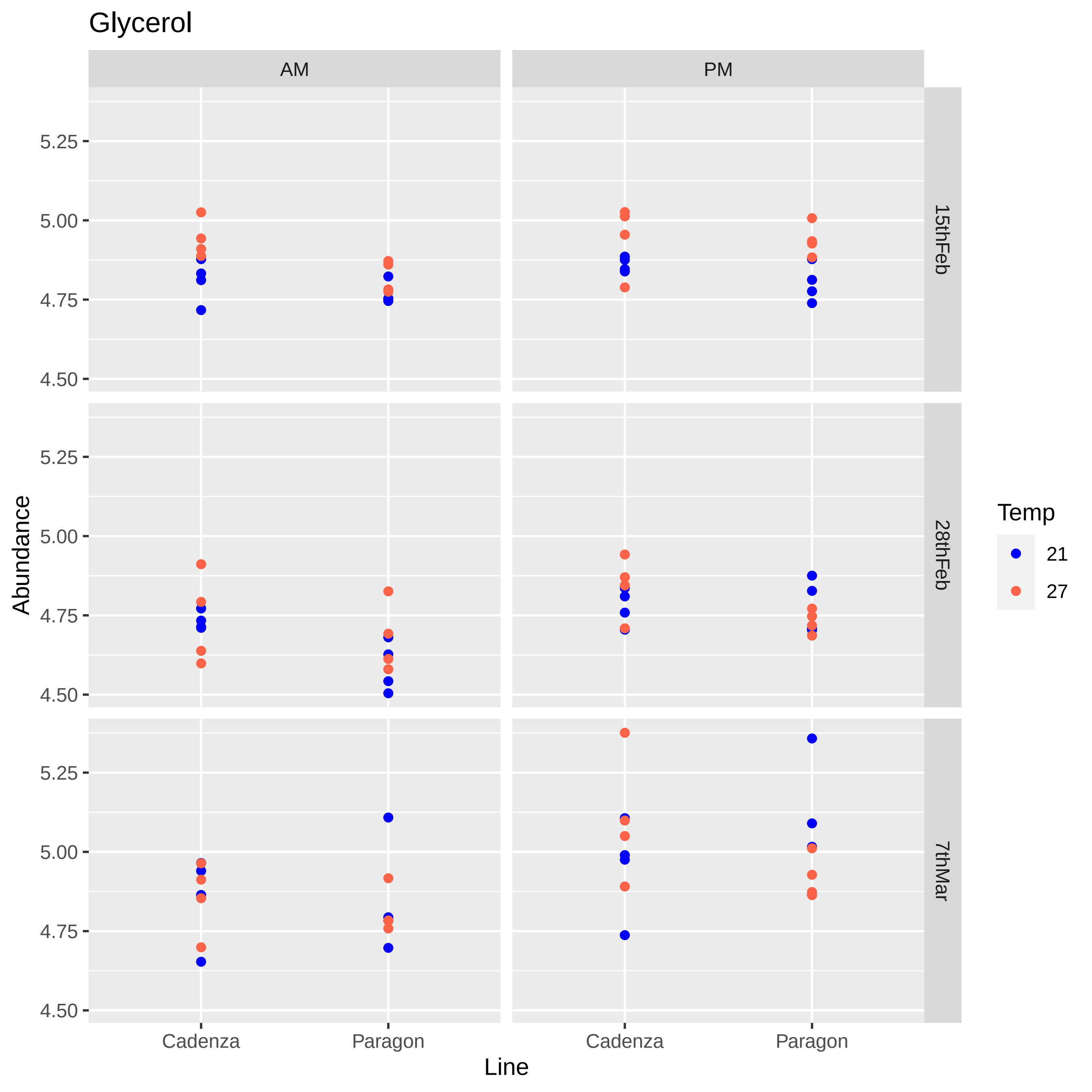
Glycerol
AM
PM
15thFeb
28thFeb
7thMar
5.25
5.00
4.75
4.50
Abundance
Cadenza
Paragon
Line
Temp
21
27

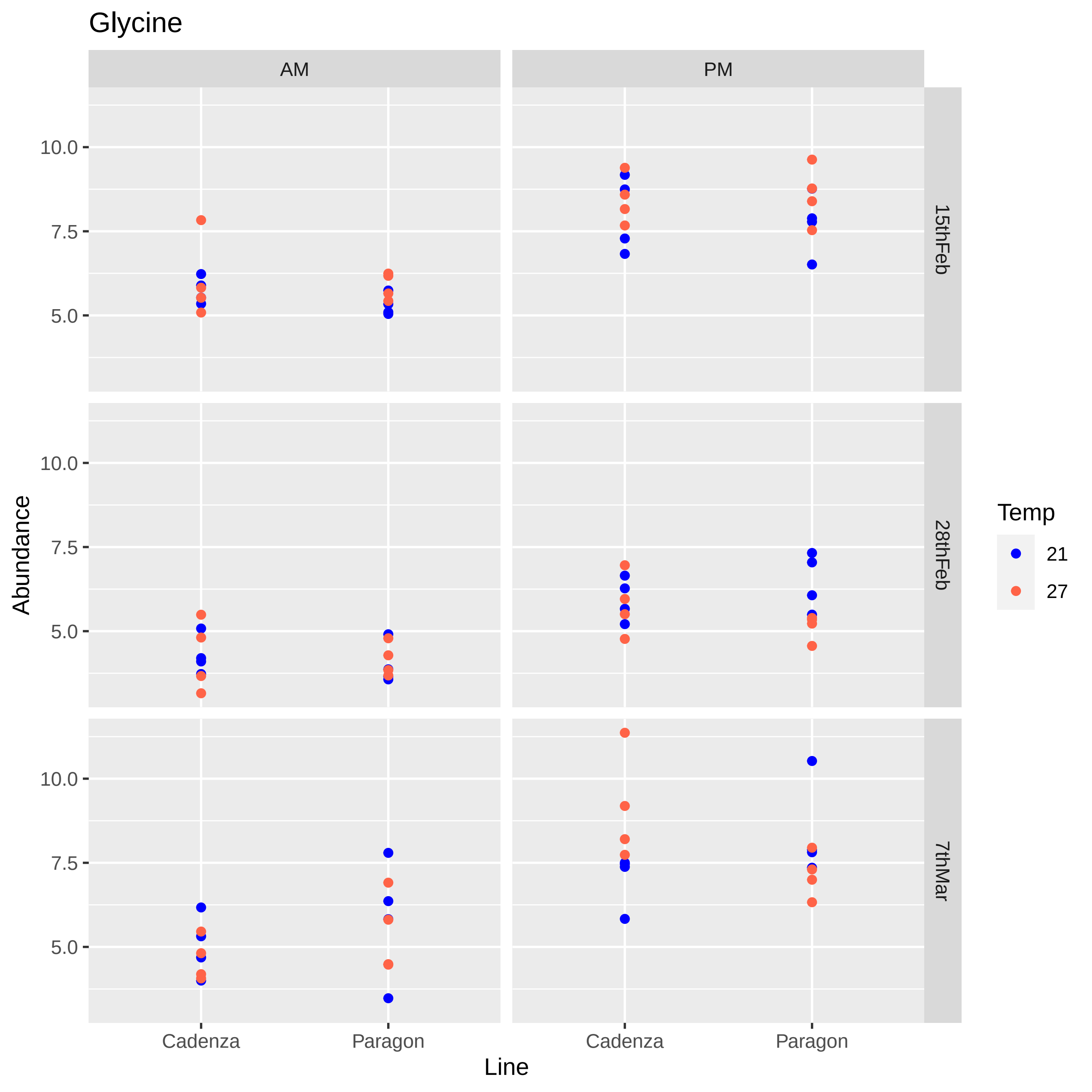

Glycine
AM
PM
15thFeb
28thFeb
7thMar
10.0
7.5
5.0
Abundance
Cadenza
Paragon
Line
Temp
21
27

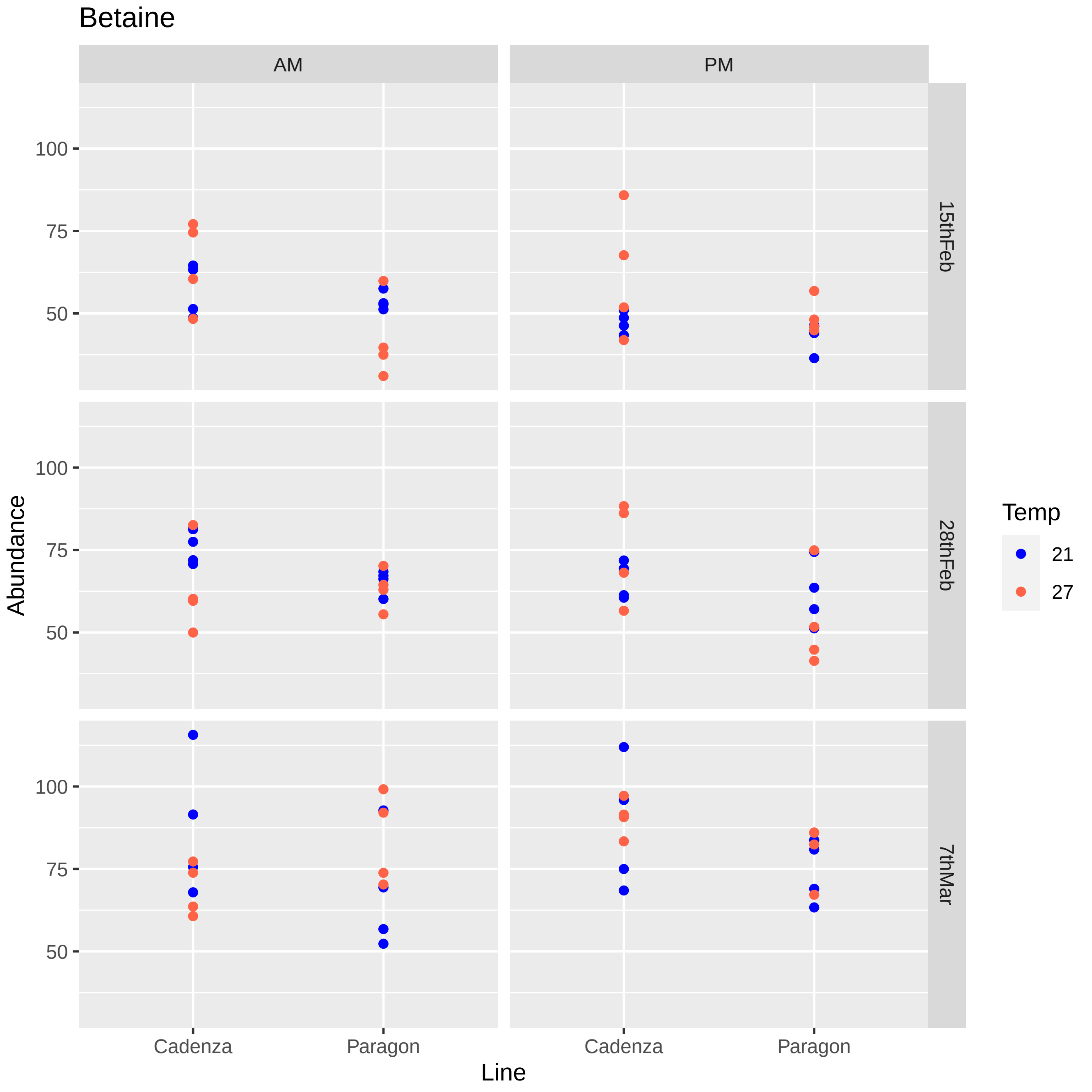

Betaine
AM
PM
15thFeb
28thFeb
7thMar
Abundance
100
75
50
Cadenza
Paragon
Line
Temp
21
27

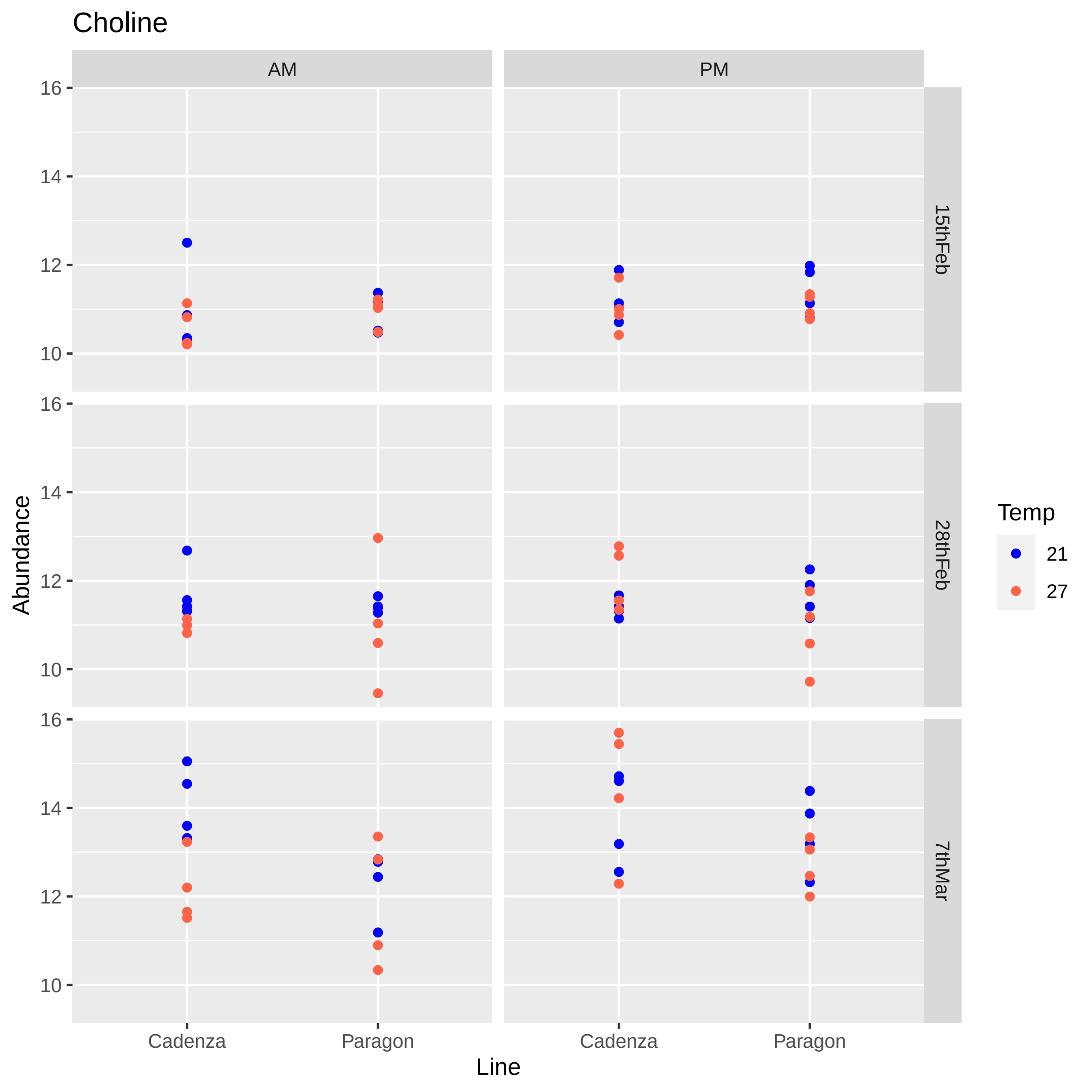
Choline
AM
PM
15thFeb
28thFeb
7thMar
Abundance
16
14
12
10
Cadenza
Paragon
Line
Temp
21
27

Asparagine

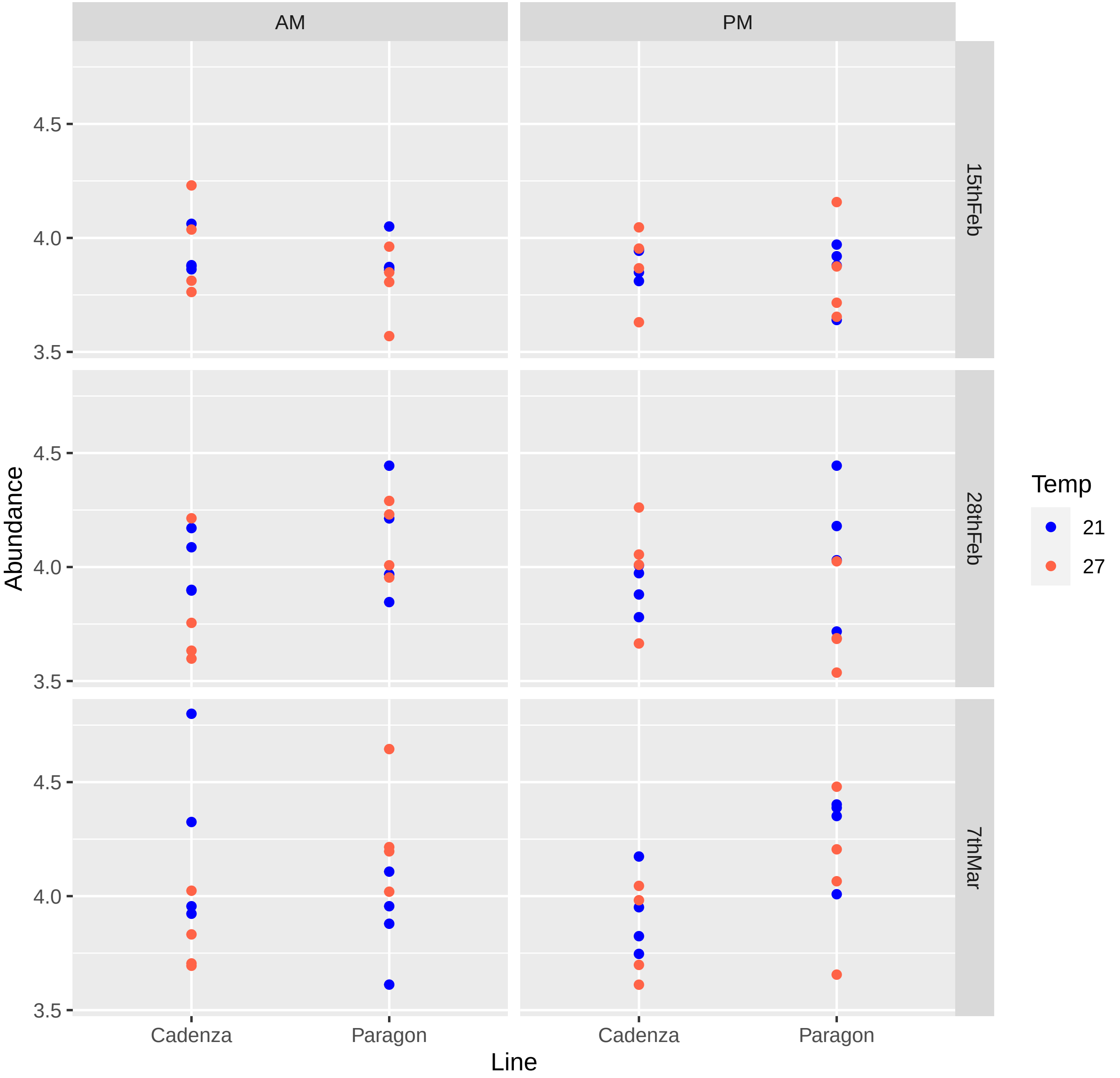

AM
PM
15thFeb
28thFeb
7thMar
4.5
4.0
3.5
Abundance
Cadenza
Paragon
Line
Temp
21
27

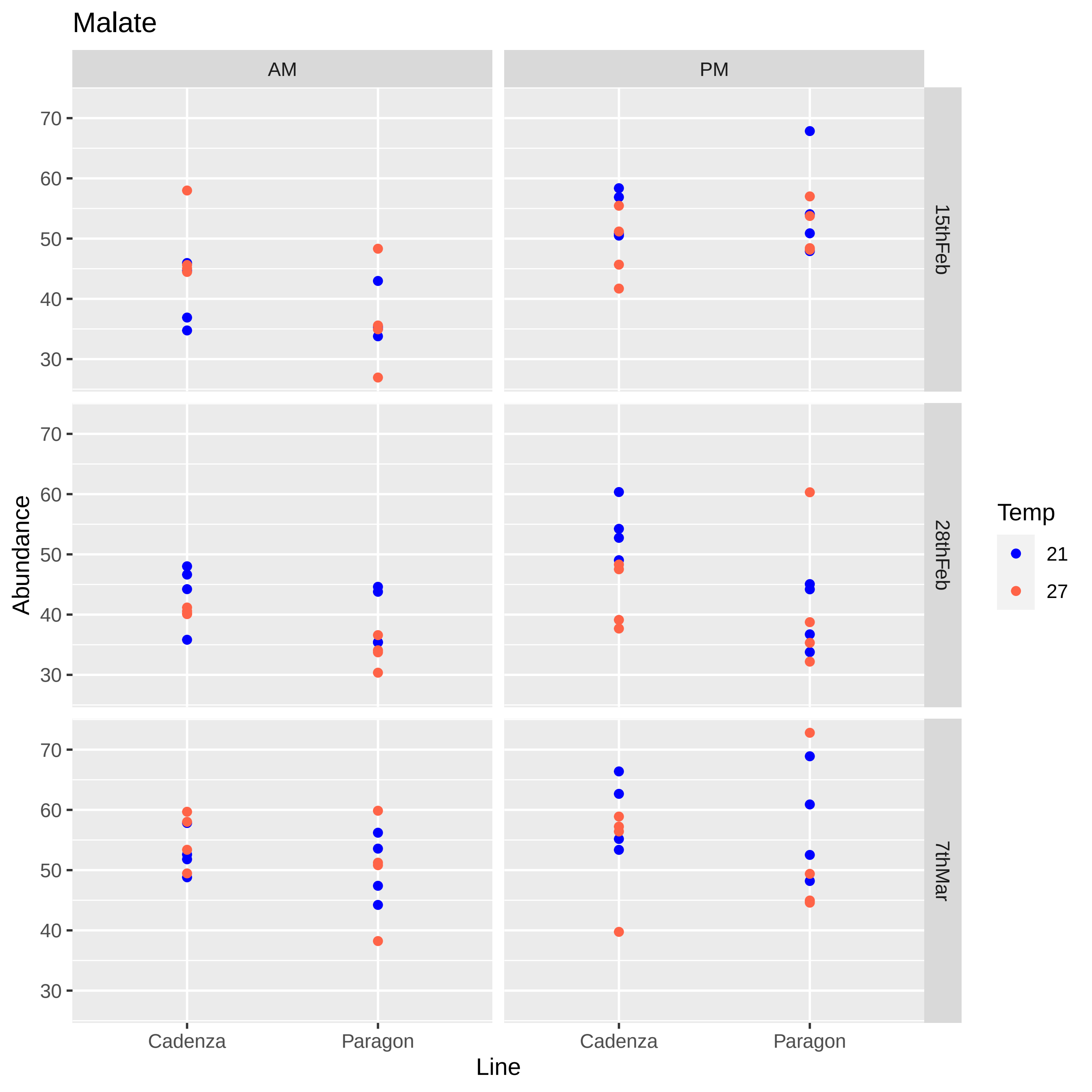

Malate
AM
PM
15thFeb
28thFeb
7thMar
70
60
50
40
30
Abundance
Cadenza
Paragon
Line
Temp
21
27

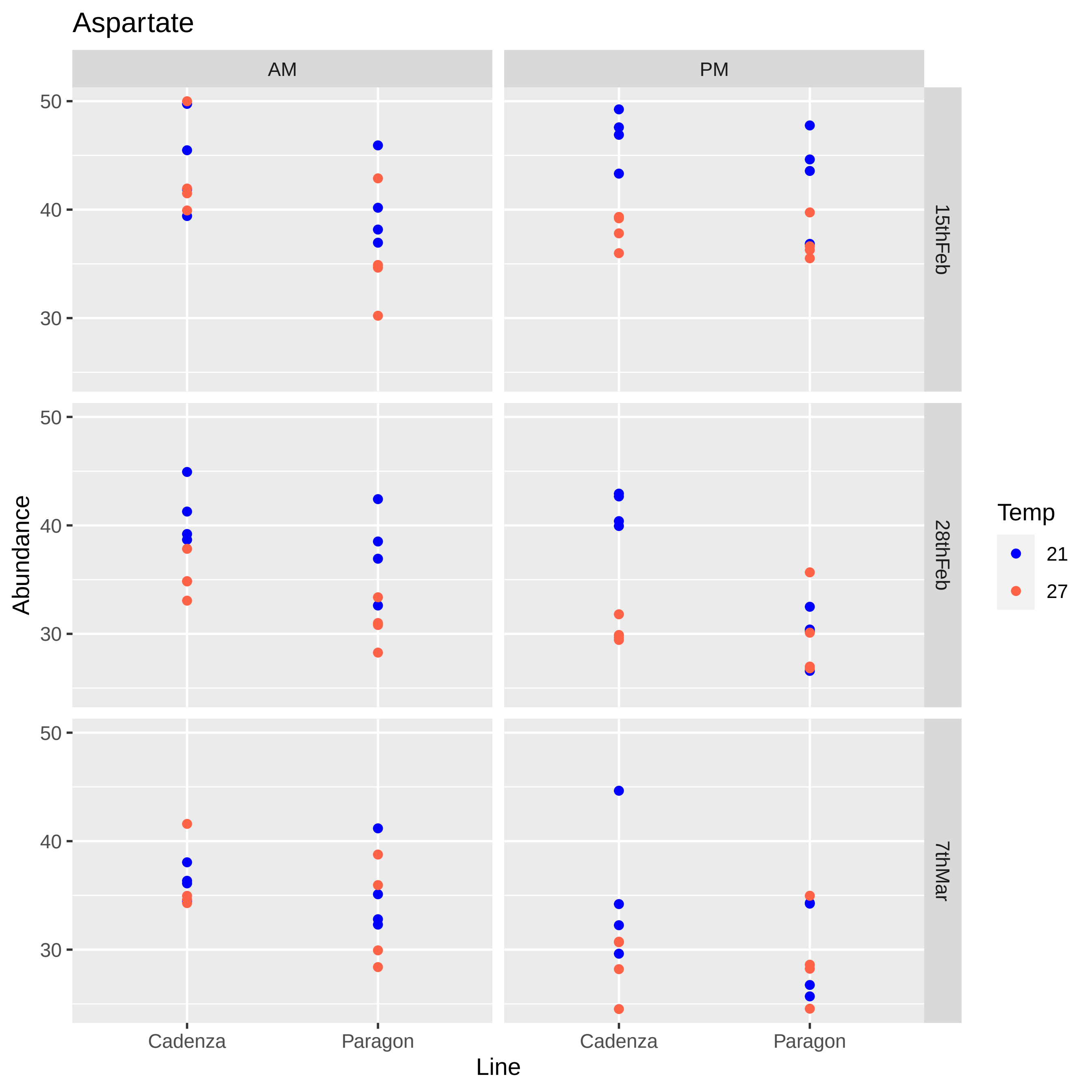

Aspartate
AM
PM
15thFeb
28thFeb
7thMar
50
40
30
Abundance
Cadenza
Paragon
Line
Temp
21
27

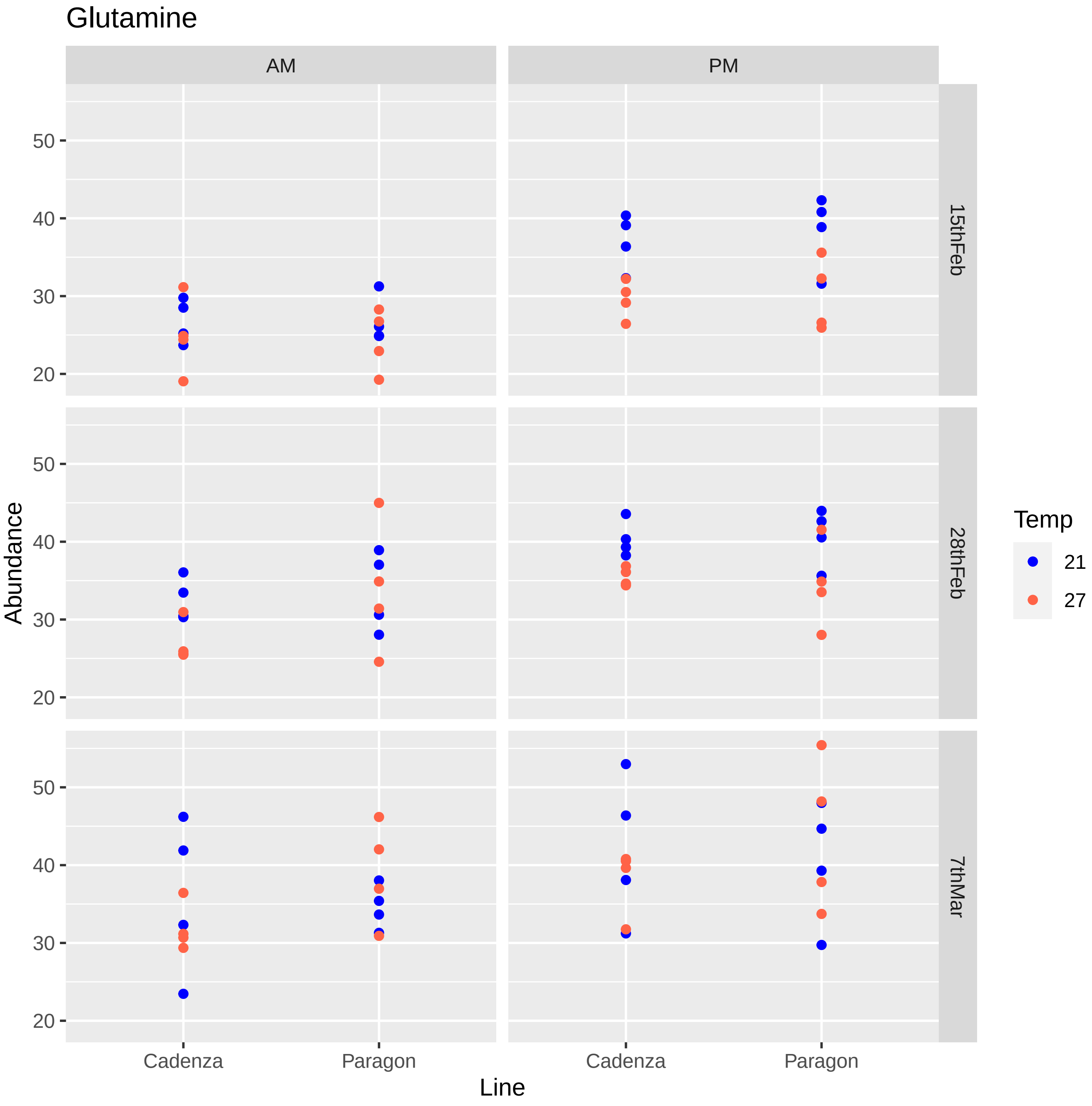

Glutamine
AM
PM
15thFeb
28thFeb
7thMar
Abundance
50
40
30
20
Cadenza
Paragon
Line
Temp
21
27

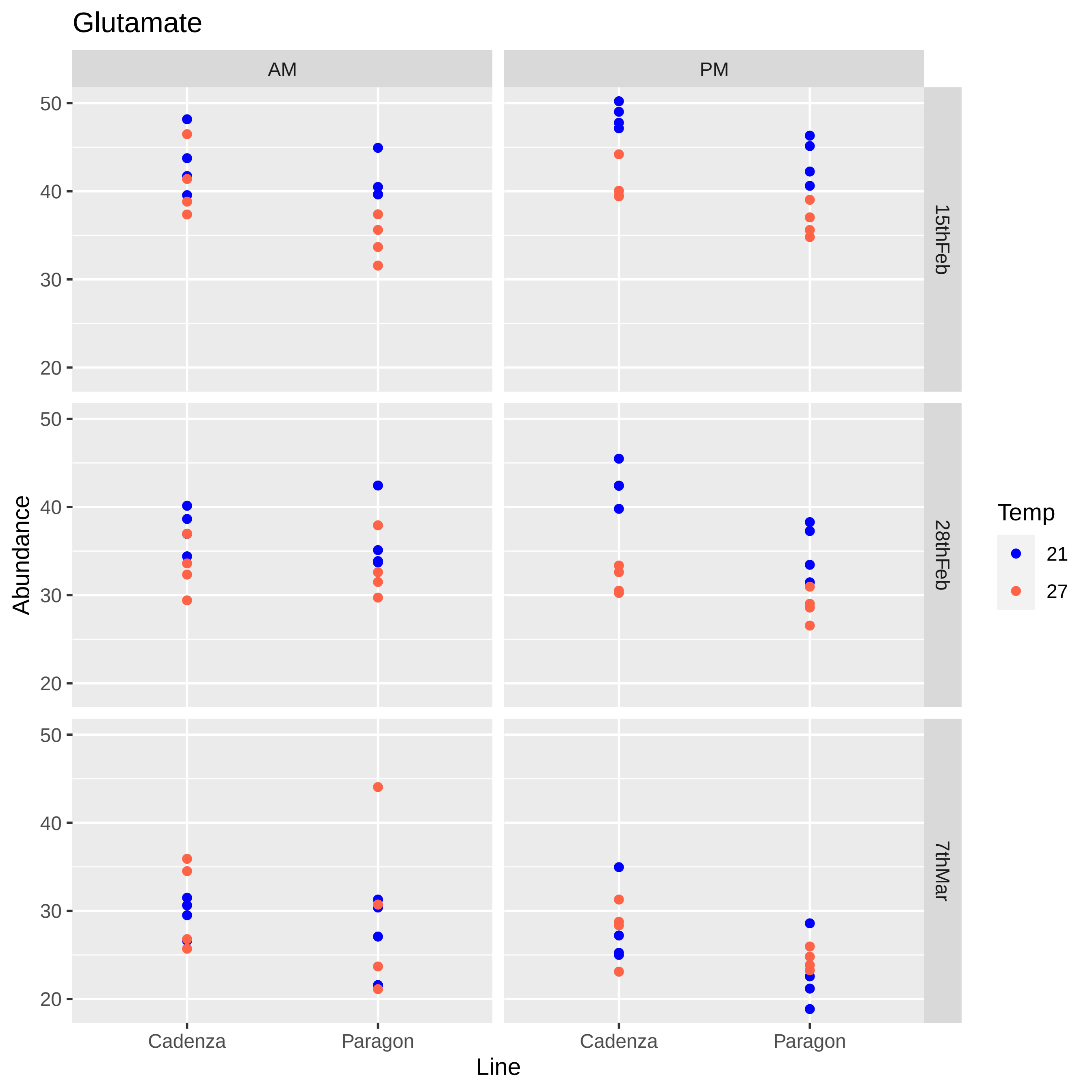
Glutamate
AM
PM
15thFeb
28thFeb
7thMar
50
40
30
20
Abundance
Cadenza
Paragon
Line
Temp
21
27

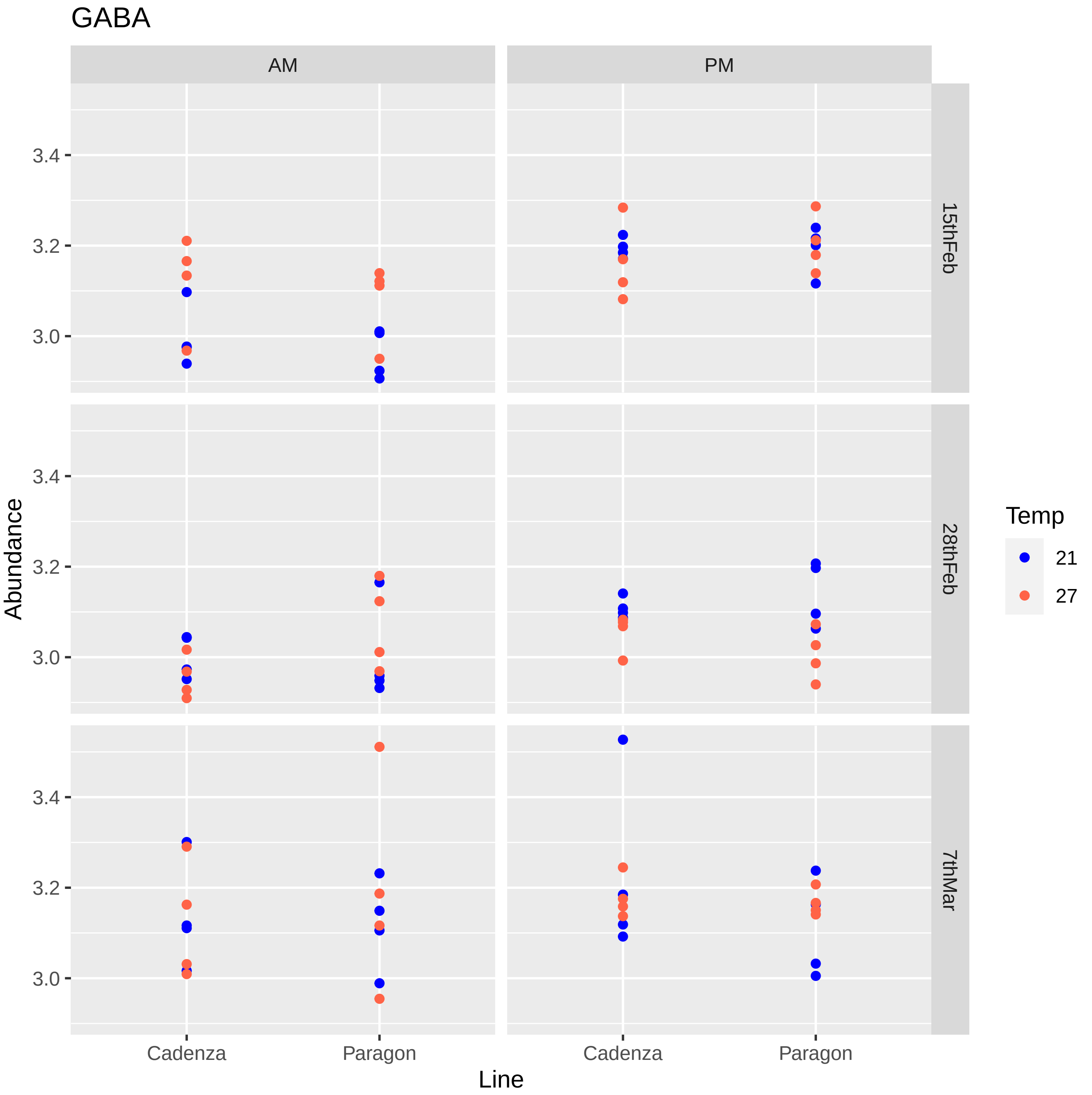

GABA
AM
PM
15thFeb
28thFeb
7thMar
3.4
3.2
3.0
Abundance
Cadenza
Paragon
Line
Temp
21
27

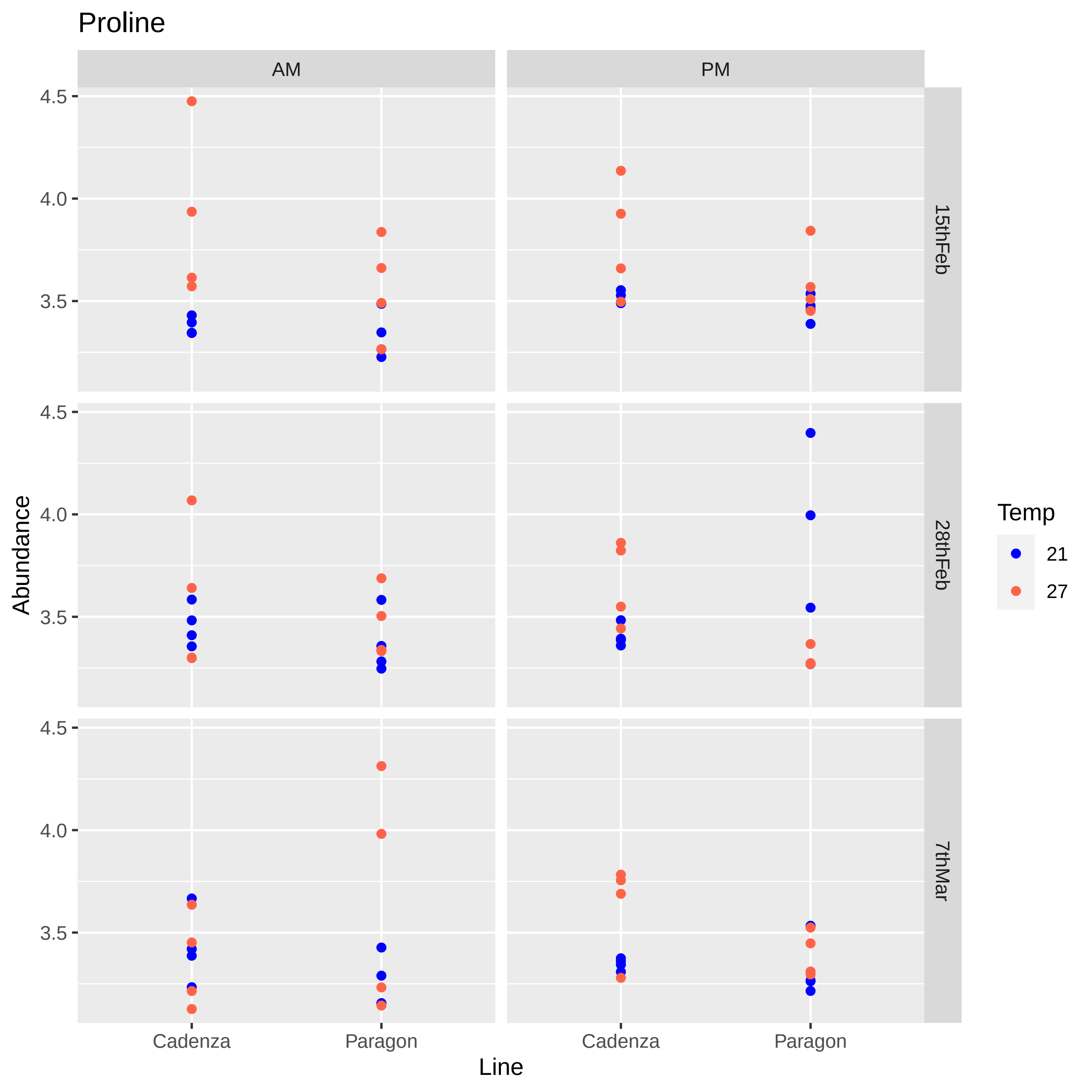

Proline
AM
PM
15thFeb
28thFeb
7thMar
Abundance
4.5
4.0
3.5
Cadenza
Paragon
Line
Temp
21
27

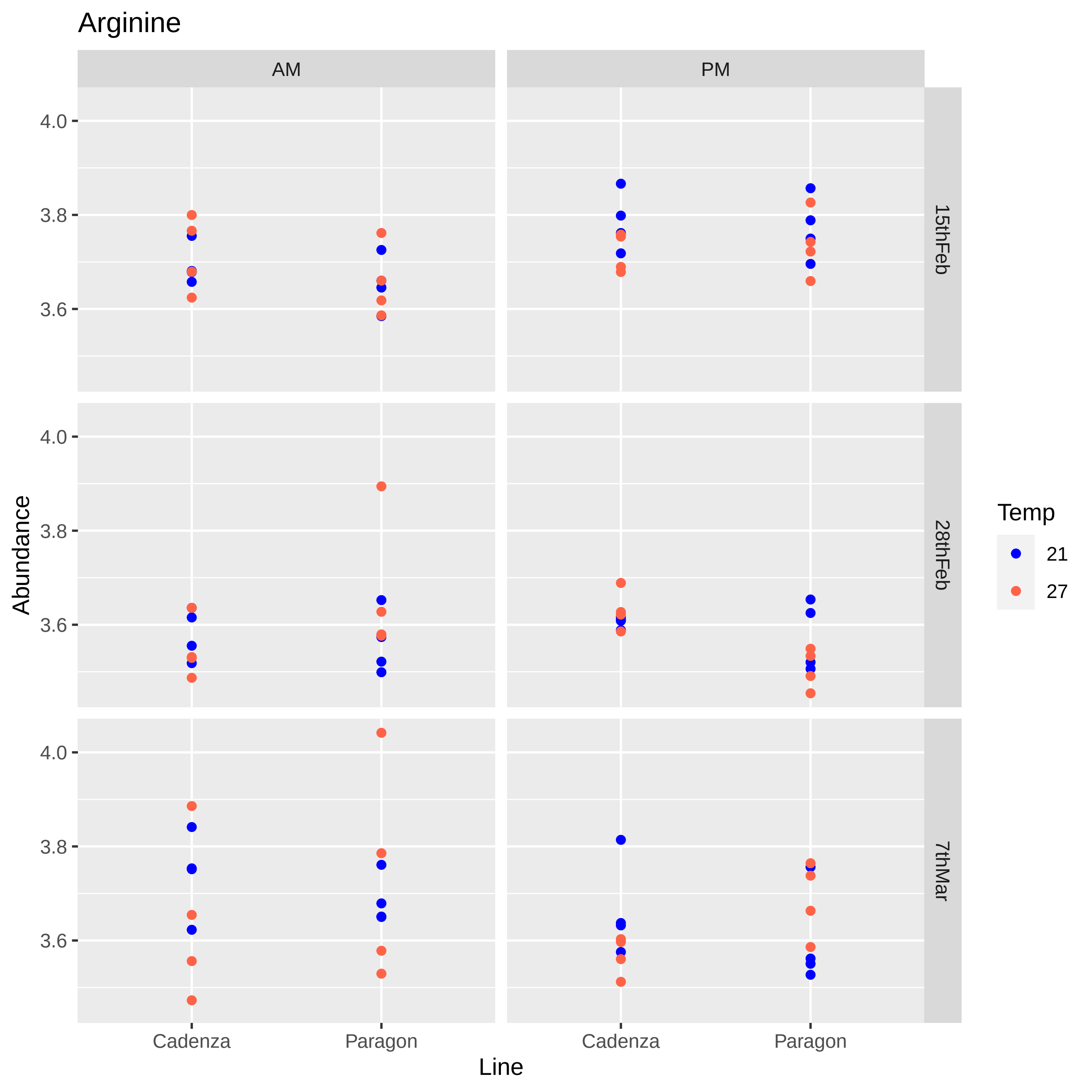
Arginine
AM
PM
15thFeb
28thFeb
7thMar
4.0
3.8
3.6
Abundance
Cadenza
Paragon
Line
Temp
21
27

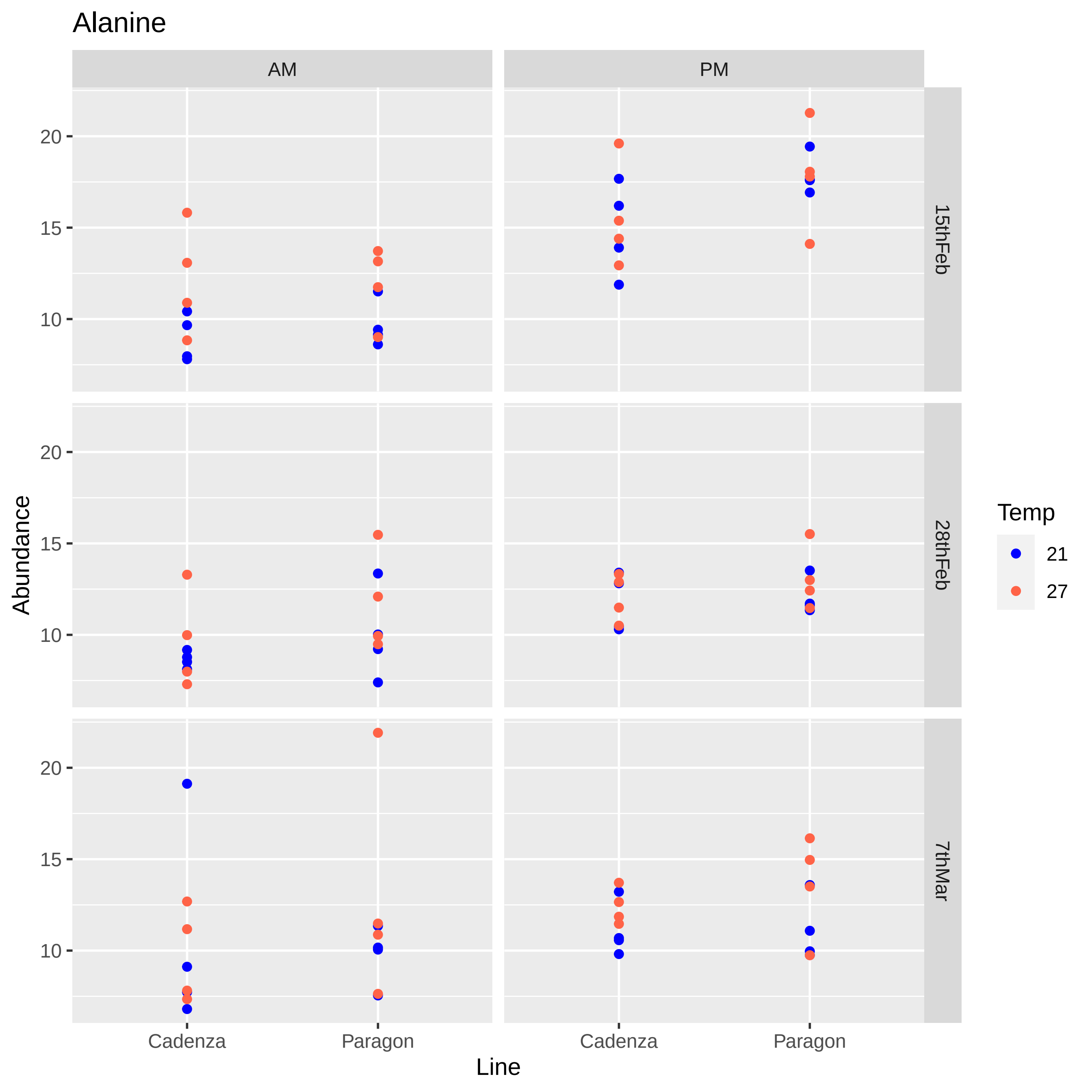
Alanine
AM
PM
15thFeb
28thFeb
7thMar
Abundance
20
15
10
Cadenza
Paragon
Line
Temp
21
27

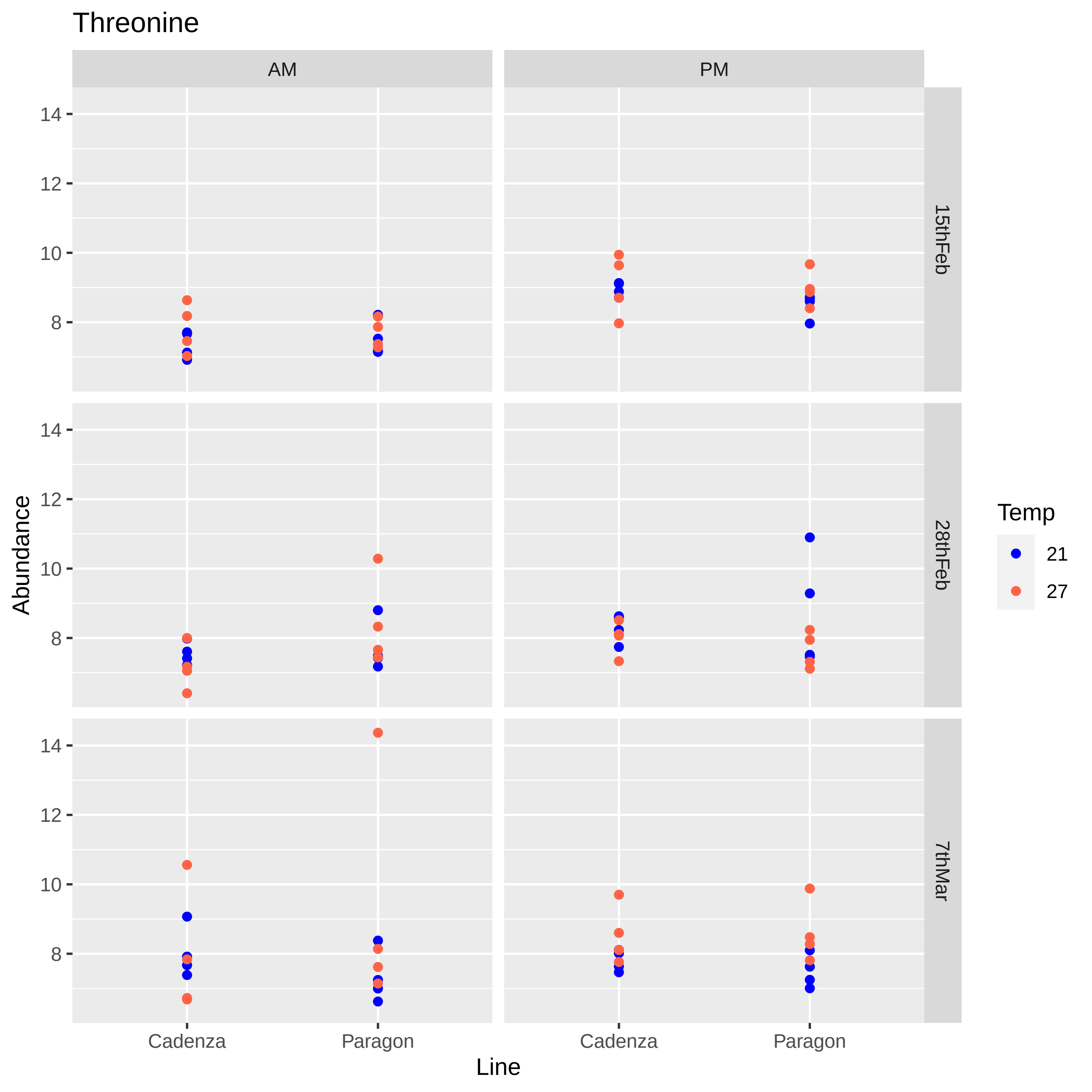

Threonine
AM
PM
15thFeb
28thFeb
7thMar
14
12
10
8
Abundance
Cadenza
Paragon
Line
Temp
21
27

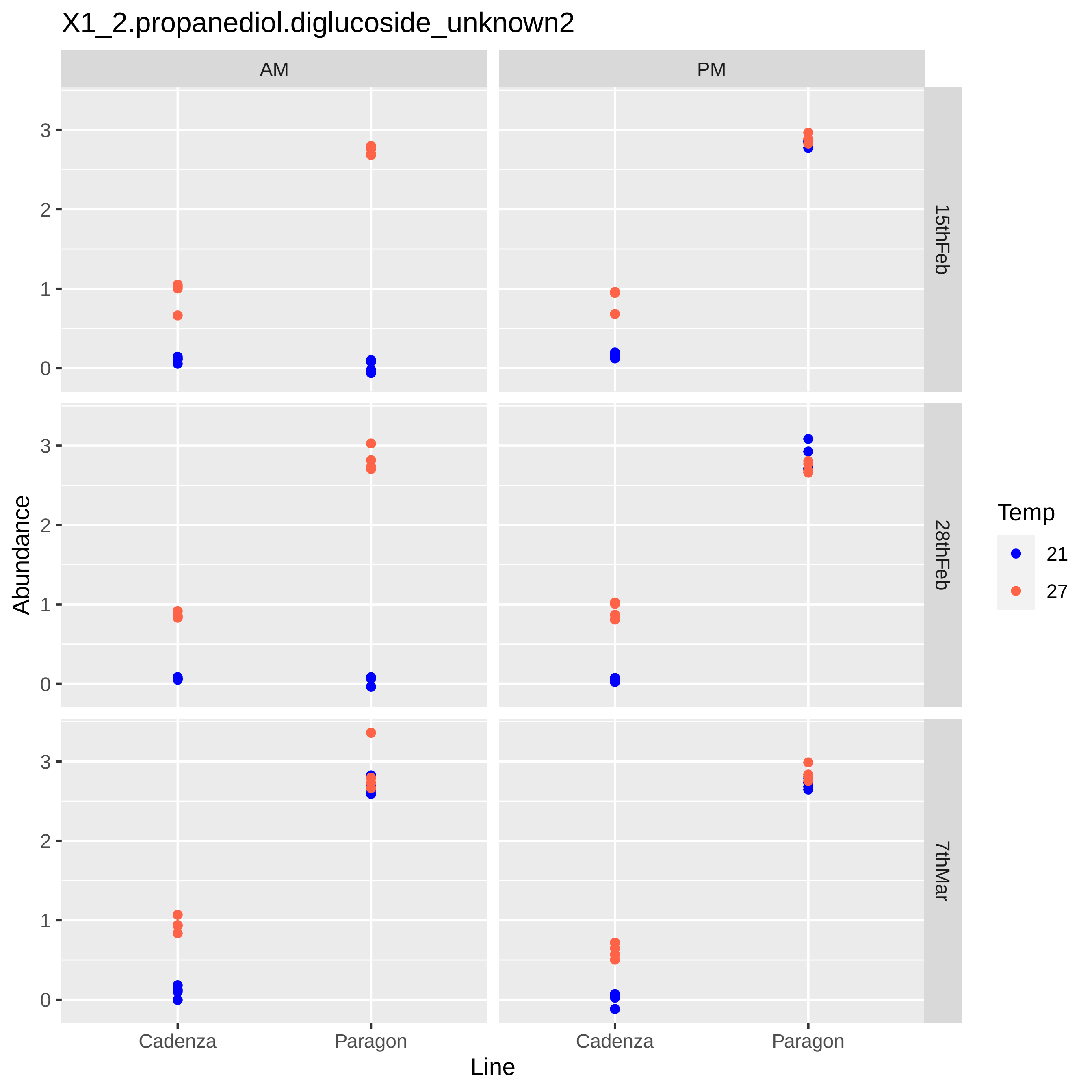

X1_2.propanediol.diglucoside_unknown2
AM
PM
15thFeb
28thFeb
7thMar
Abundance
0
1
2
3
Cadenza
Paragon
Line
Temp
21
27

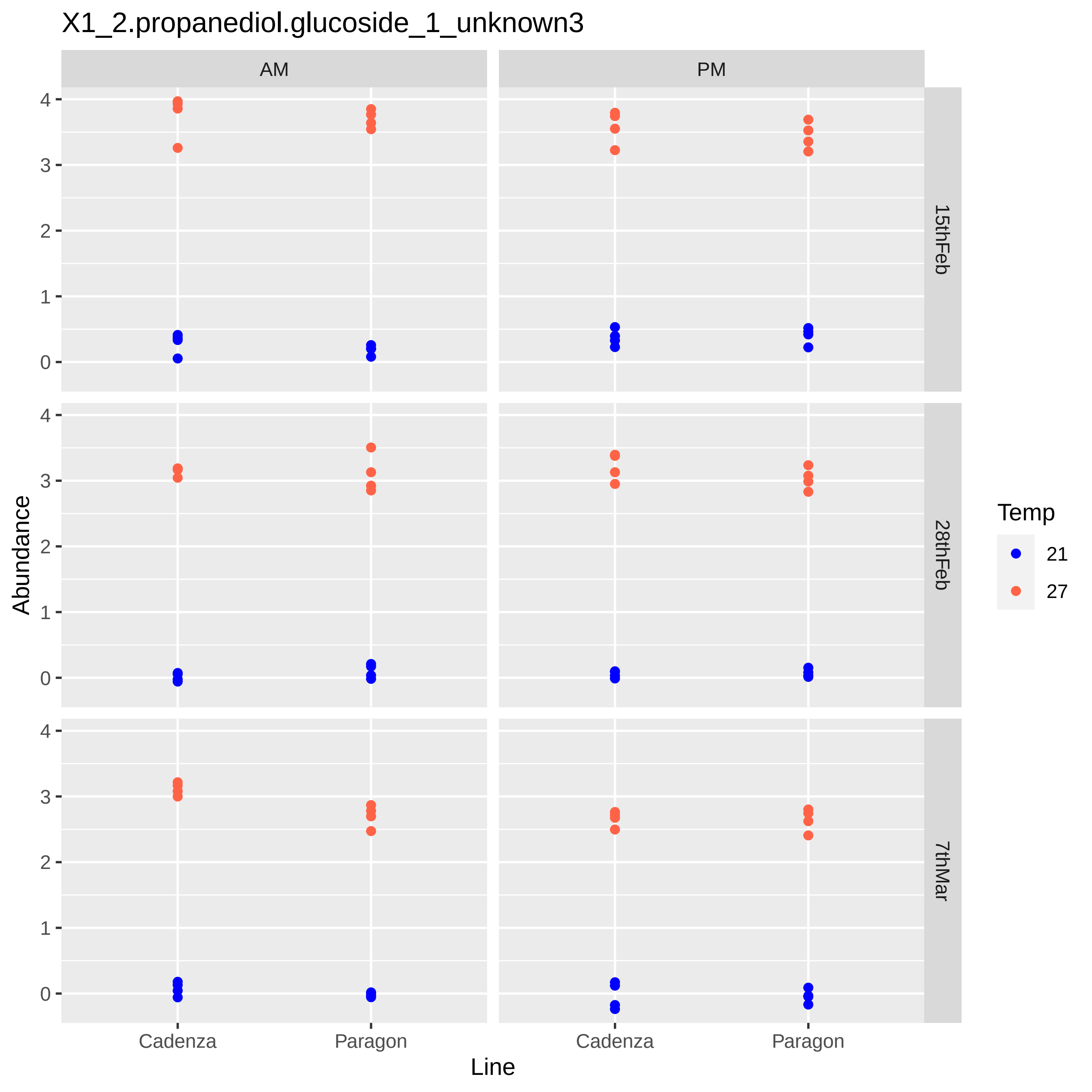

X1_2.propanediol.glucoside_1_unknown3
AM
PM
15thFeb
28thFeb
7thMar
Abundance
0
1
2
3
4
Cadenza
Paragon
Line
Temp
21
27

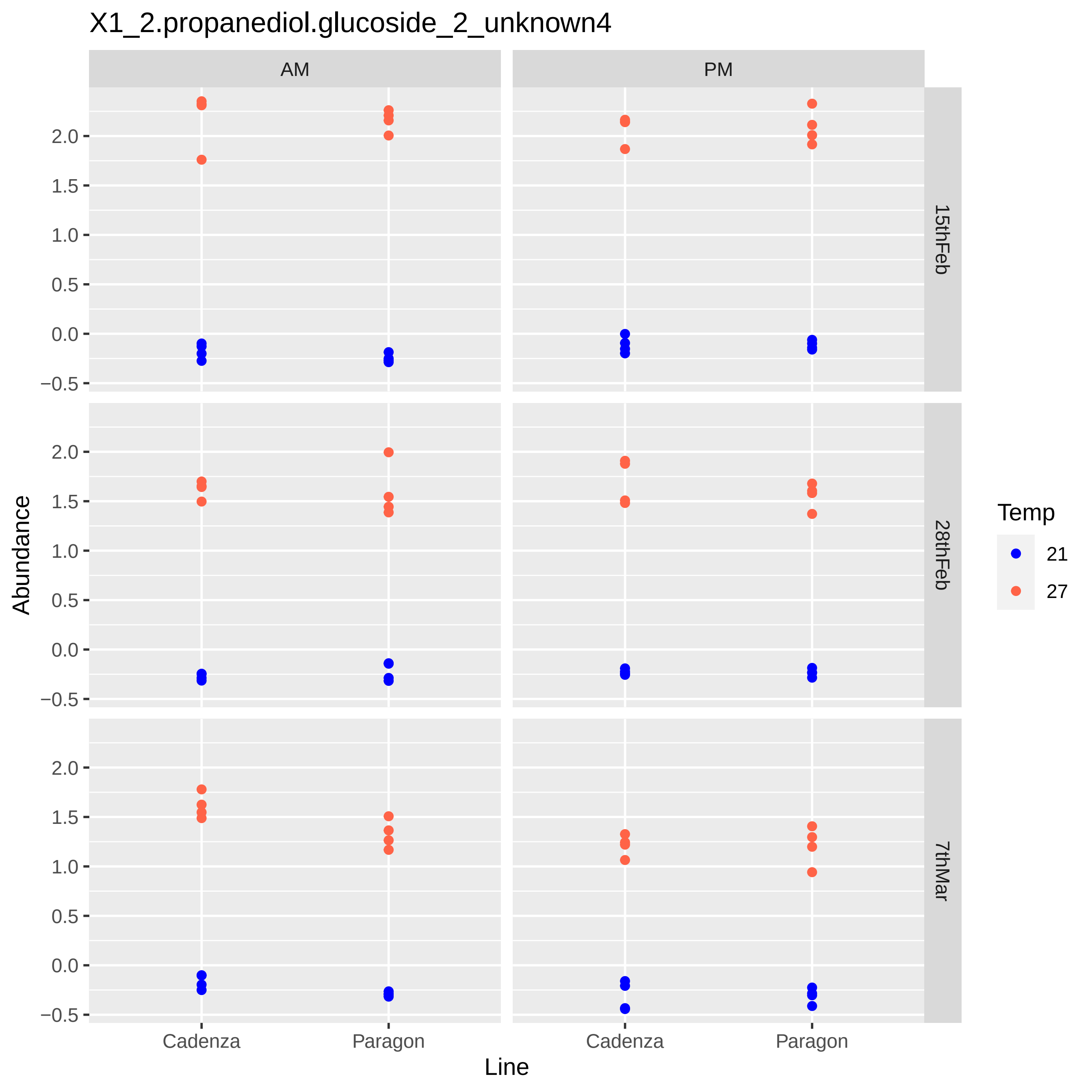

X1_2.propanediol.glucoside_2_unknown4
AM
PM
15thFeb
28thFeb
7thMar
2.0
1.5
1.0
0.5
0.0
−0.5
Abundance
Cadenza
Paragon
Line
Temp
21
27

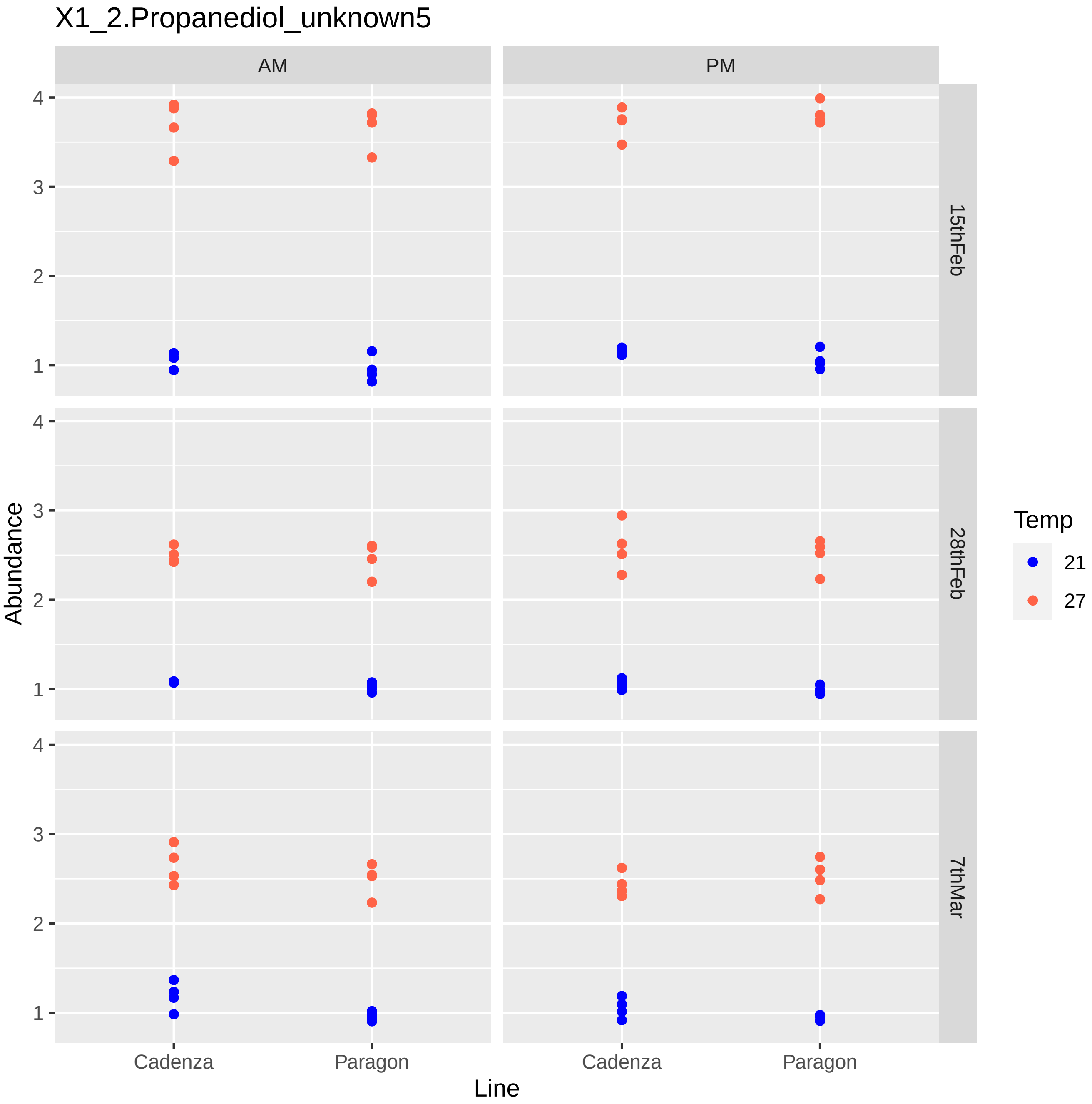

X1_2.Propanediol_unknown5
AM
PM
15thFeb
28thFeb
7thMar
Abundance
1
2
3
4
Cadenza
Paragon
Line
Temp
21
27

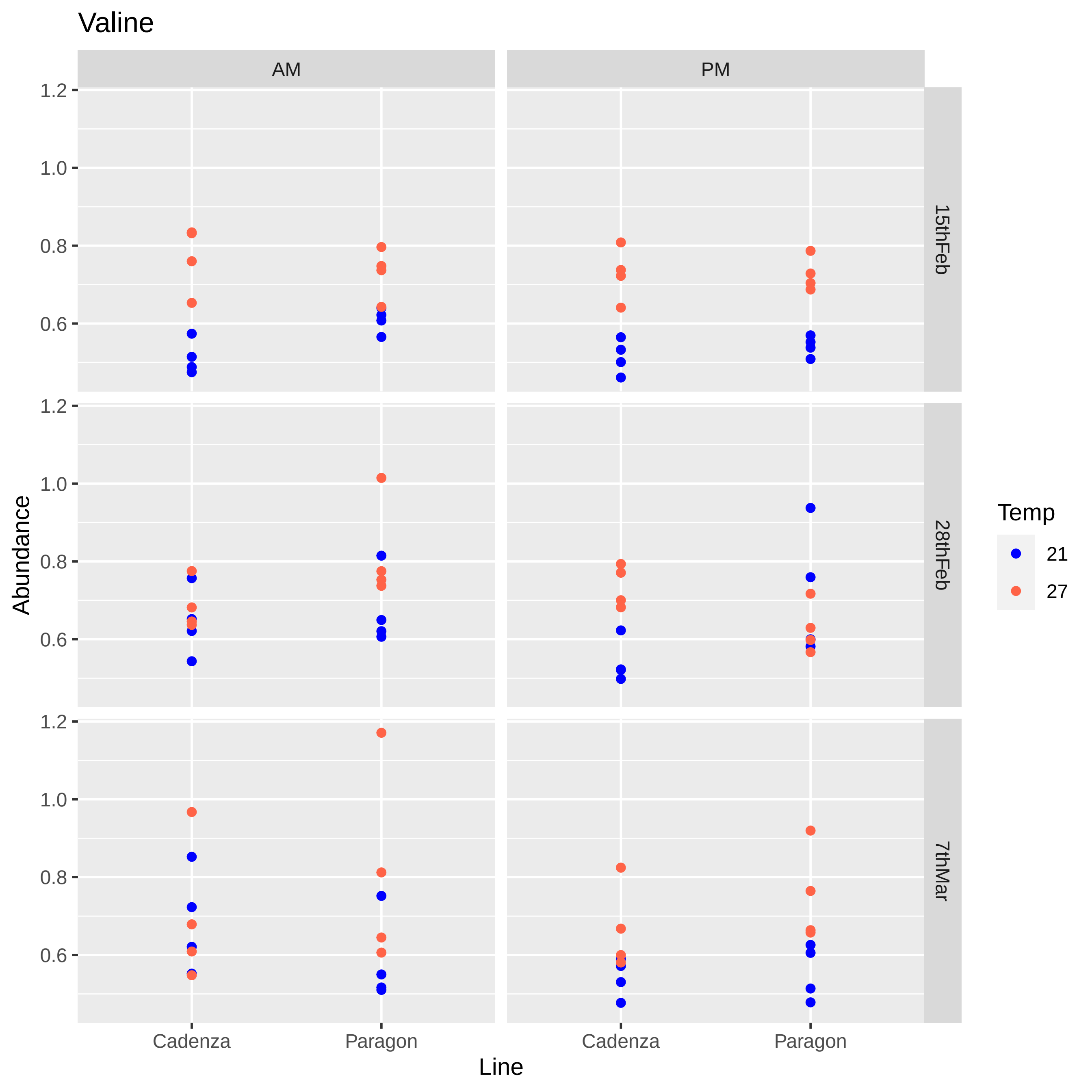

Valine
AM
PM
15thFeb
28thFeb
7thMar
Abundance
1.2
1.0
0.8
0.6
Cadenza
Paragon
Cadenza
Paragon
Line
Temp
21
27

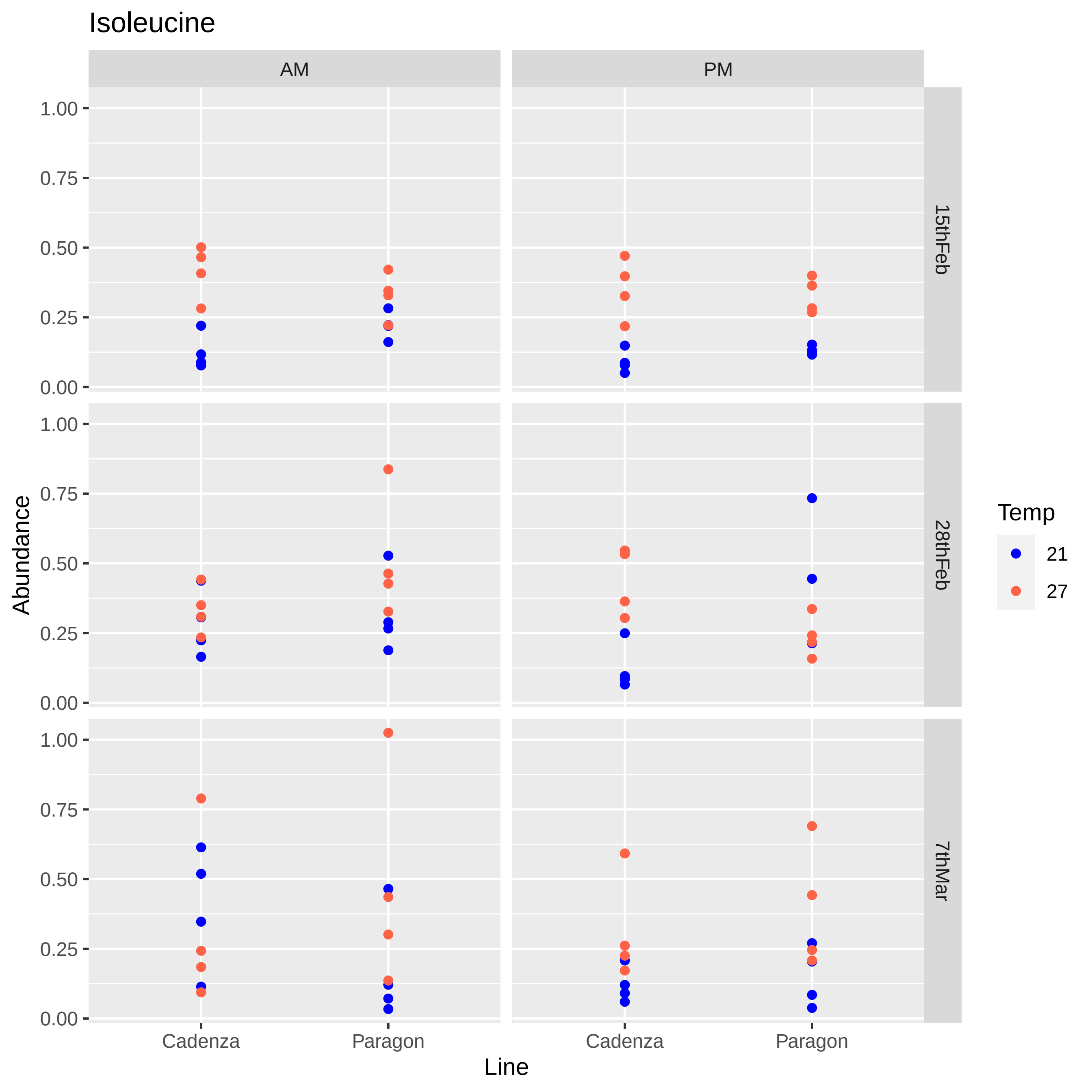

Isoleucine
AM
PM
15thFeb
28thFeb
7thMar
Abundance
0.00
0.25
0.50
0.75
1.00
Cadenza
Paragon
Line
Temp
21
27

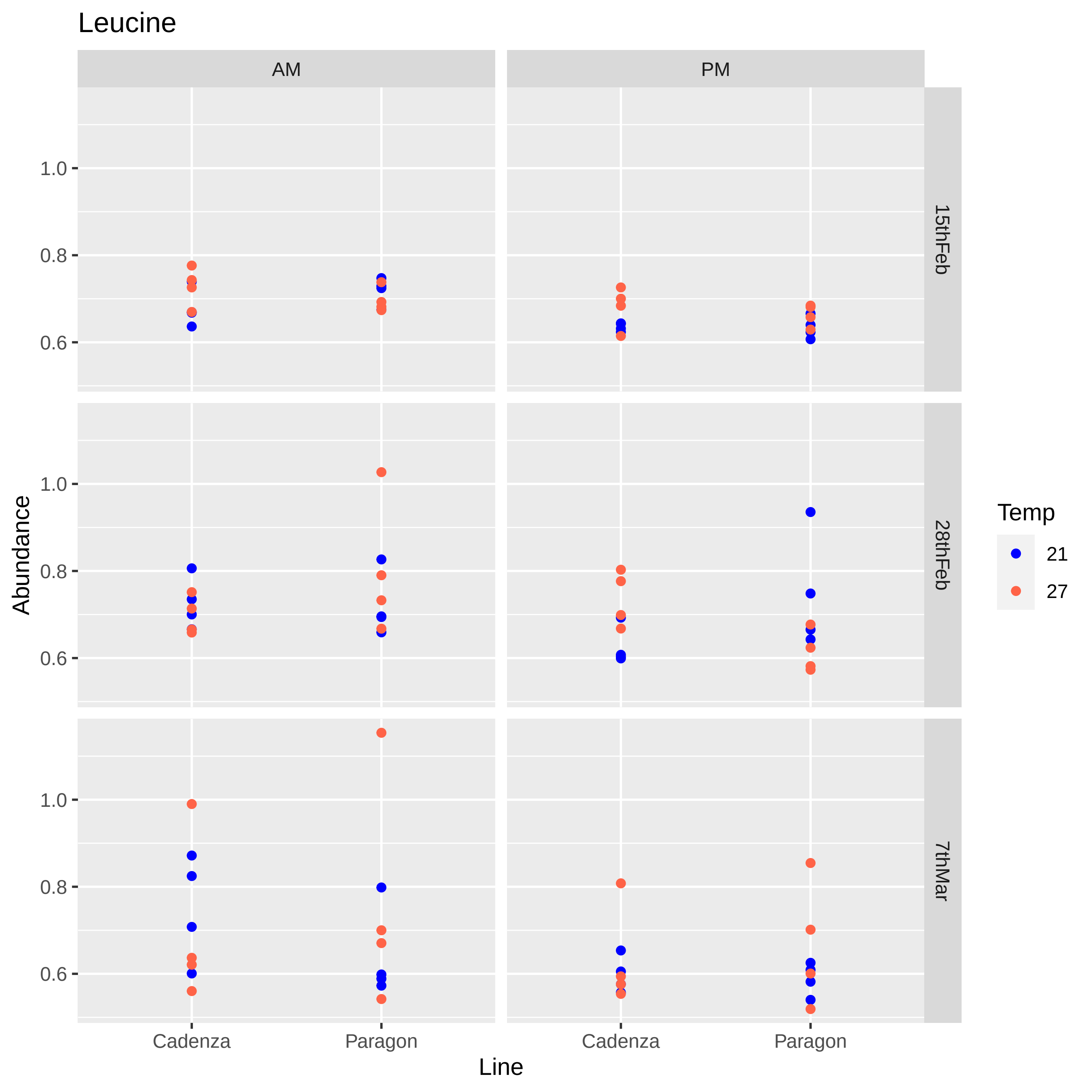
Leucine
AM
PM
15thFeb
28thFeb
7thMar
1.0
0.8
0.6
Abundance
Cadenza
Paragon
Cadenza
Paragon
Line
Temp
21
27

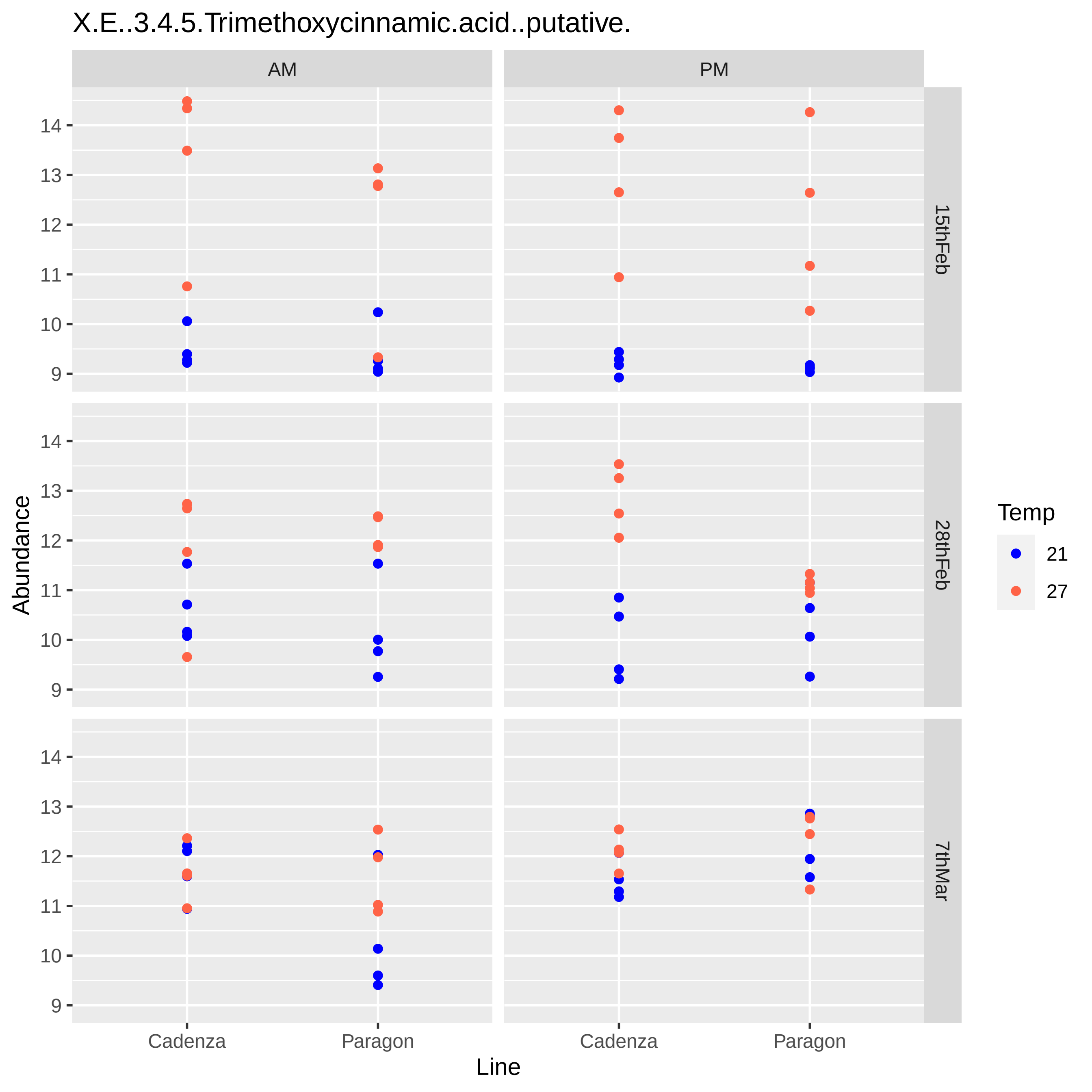
X.E..3.4.5.Trimethoxycinnamic.acid..putative.
AM
PM
15thFeb
28thFeb
7thMar
Abundance
14
13
12
11
10
9
Cadenza
Paragon
Line
Temp
21
27

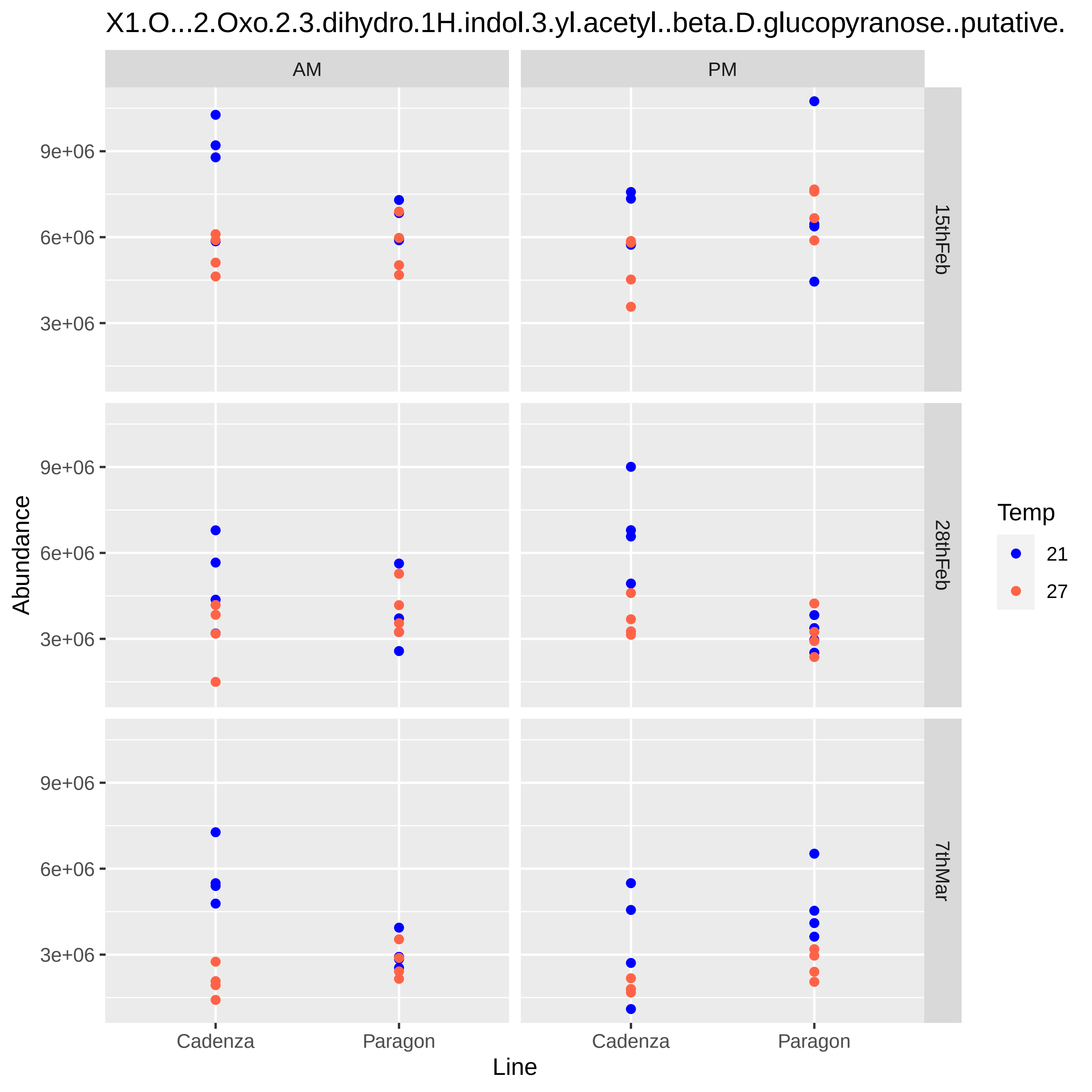
X1.O...2.Oxo.2.3.dihydro.1H.indol.3.yl.acetyl..beta.D.glucopyranose..putative.
AM
PM
15thFeb
28thFeb
7thMar
9e+06
6e+06
3e+06
Abundance
Cadenza
Paragon
Line
Temp
21
27

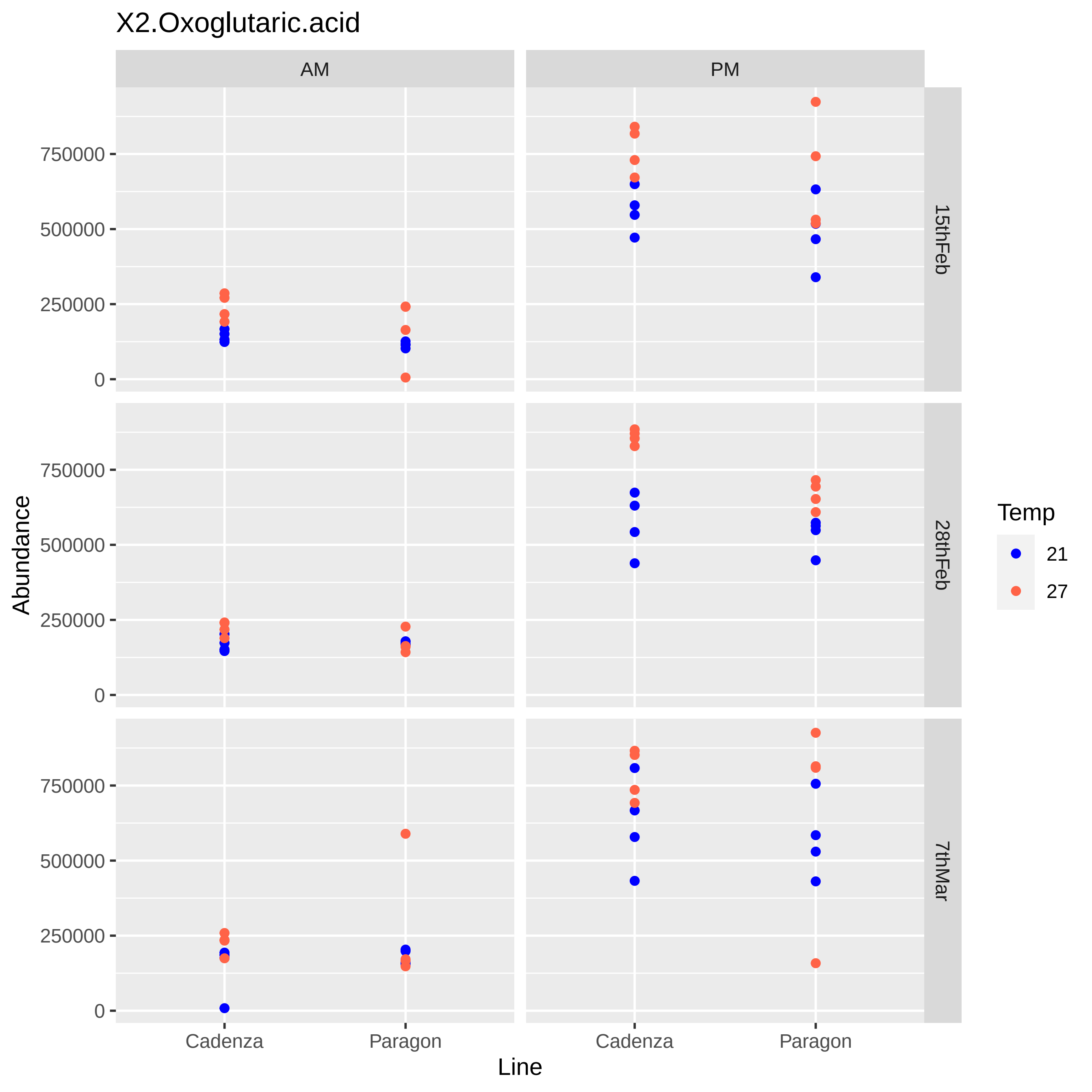
X2.Oxoglutaric.acid
AM
PM
15thFeb
28thFeb
7thMar
750000
500000
250000
0
Abundance
Cadenza
Paragon
Line
Temp
21
27

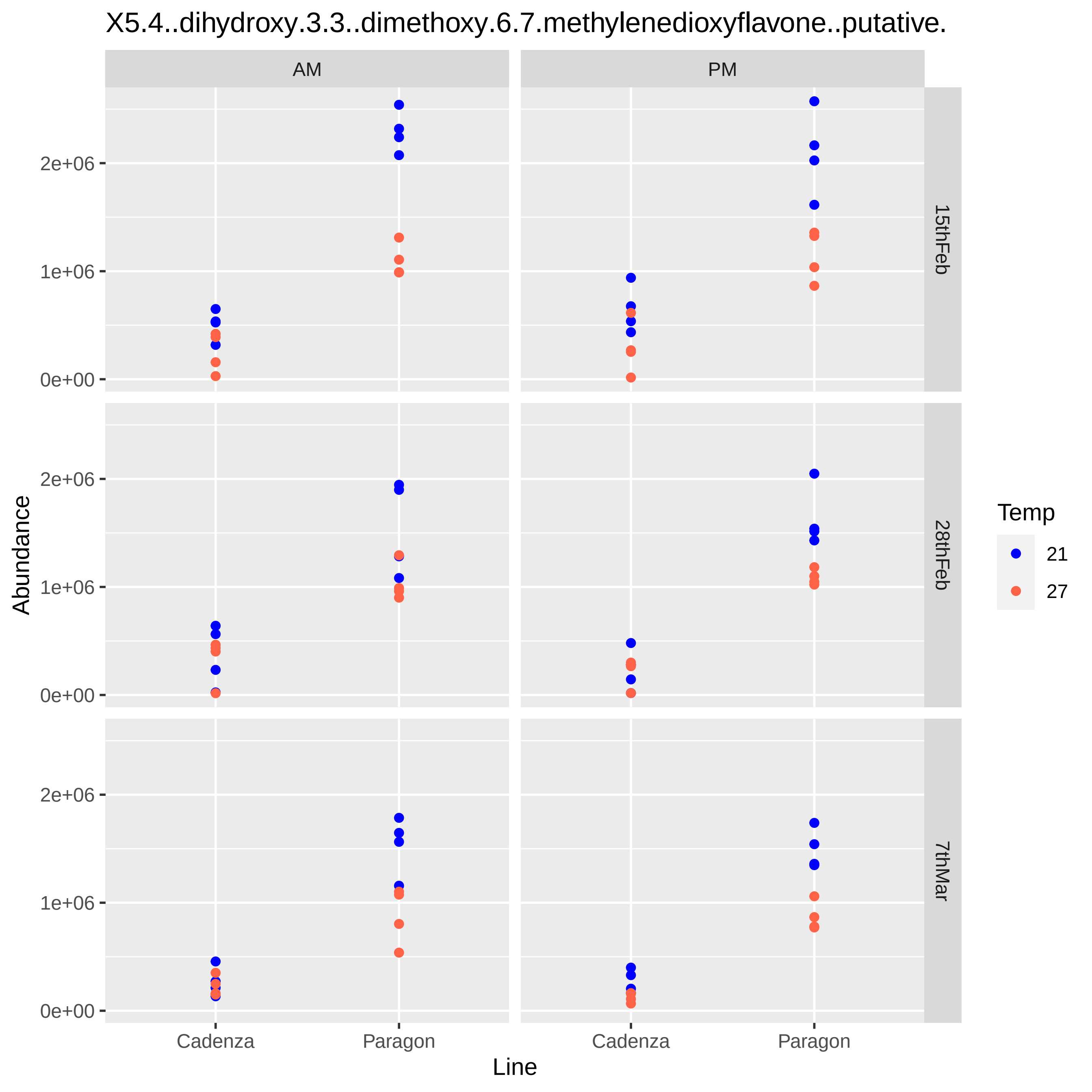
X5.4..dihydroxy.3.3..dimethoxy.6.7.methylenedioxyflavone..putative.
AM
PM
15thFeb
28thFeb
7thMar
Abundance
2e+06
1e+06
0e+00
Cadenza
Paragon
Line
Temp
21
27

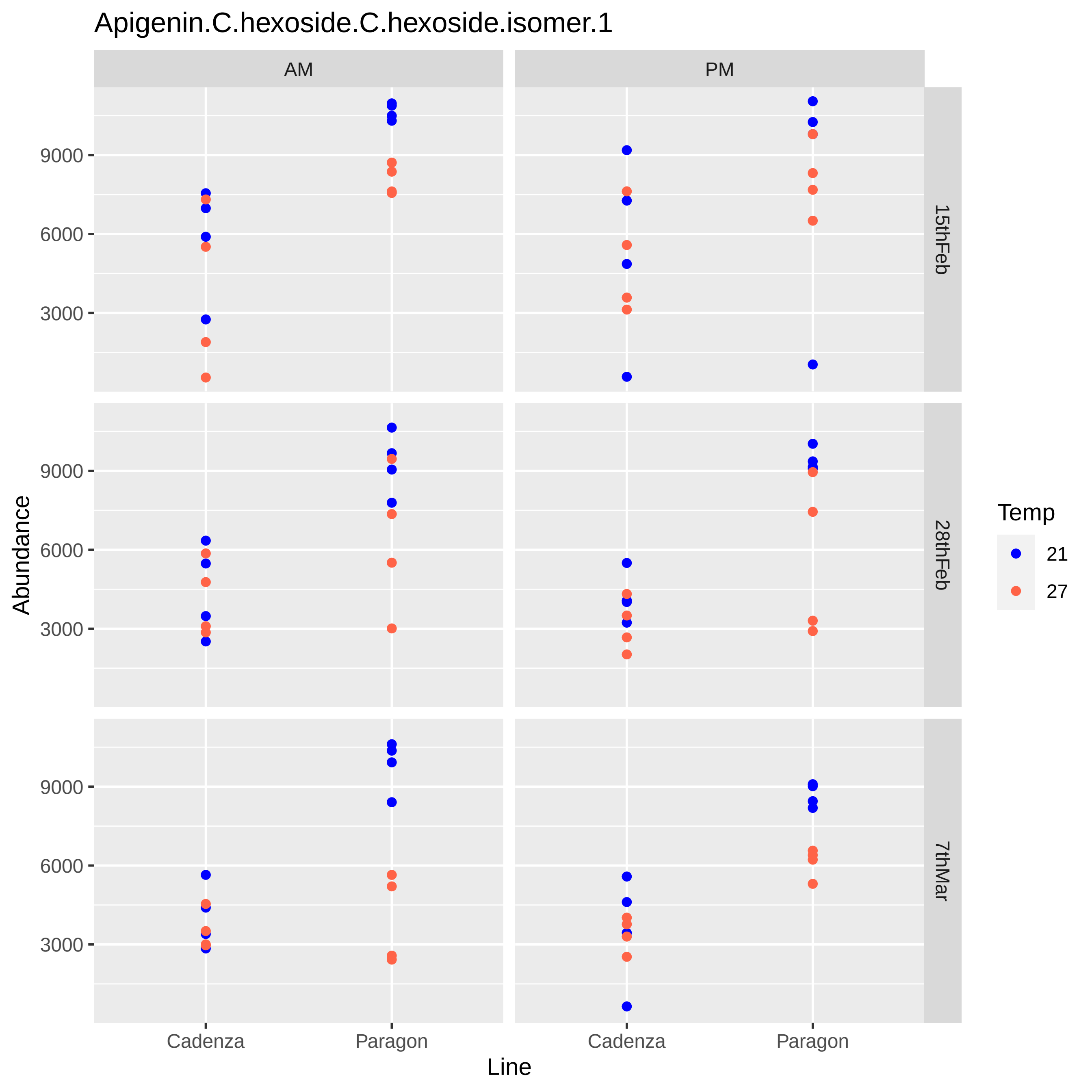

Apigenin.C.hexoside.C.hexoside.isomer.1
AM
PM
15thFeb
28thFeb
7thMar
9000
6000
3000
Abundance
Cadenza
Paragon
Line
Temp
21
27

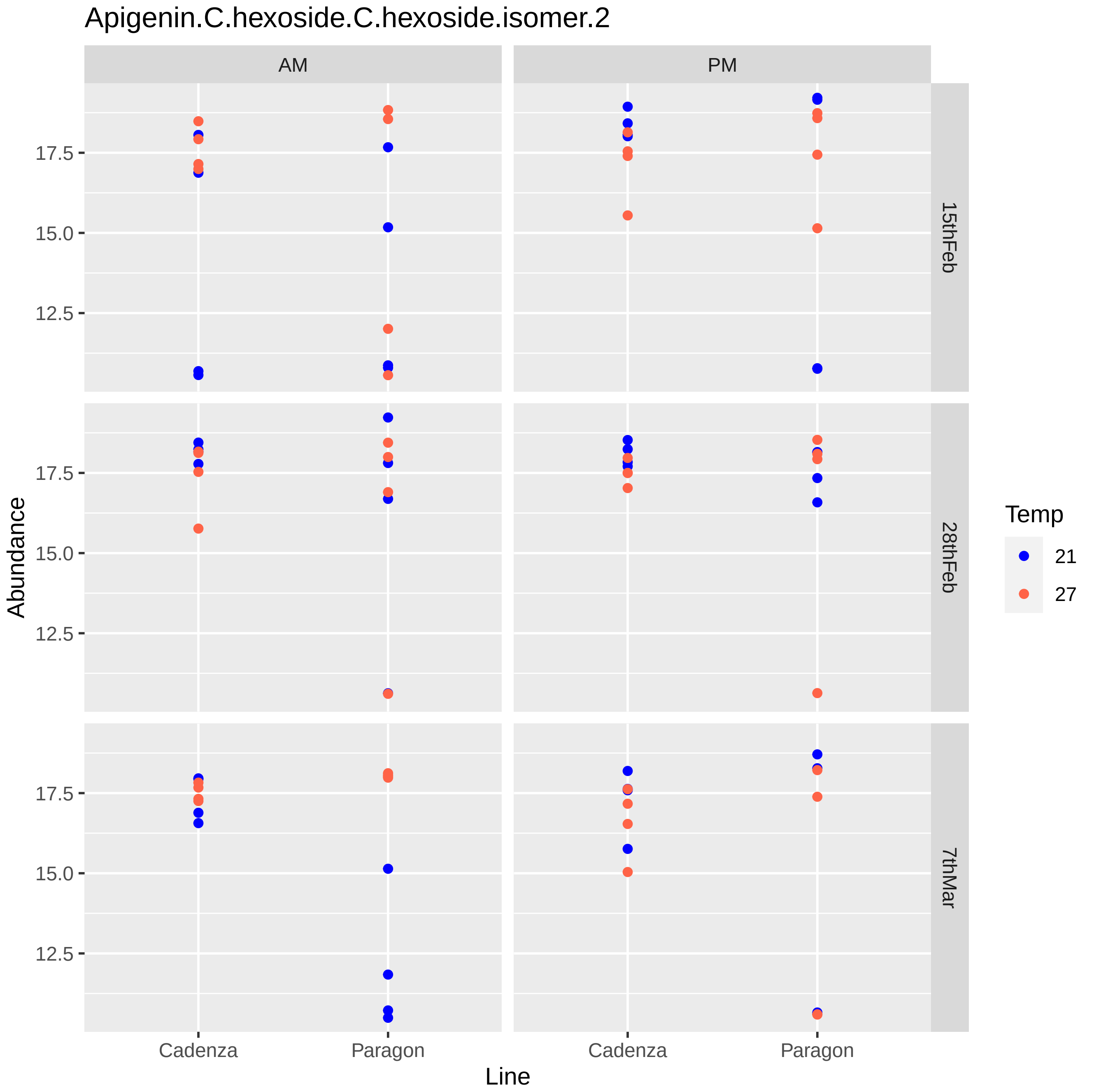
Apigenin.C.hexoside.C.hexoside.isomer.2
AM
PM
15thFeb
28thFeb
7thMar
Abundance
17.5
15.0
12.5
Cadenza
Paragon
Line
Temp
21
27

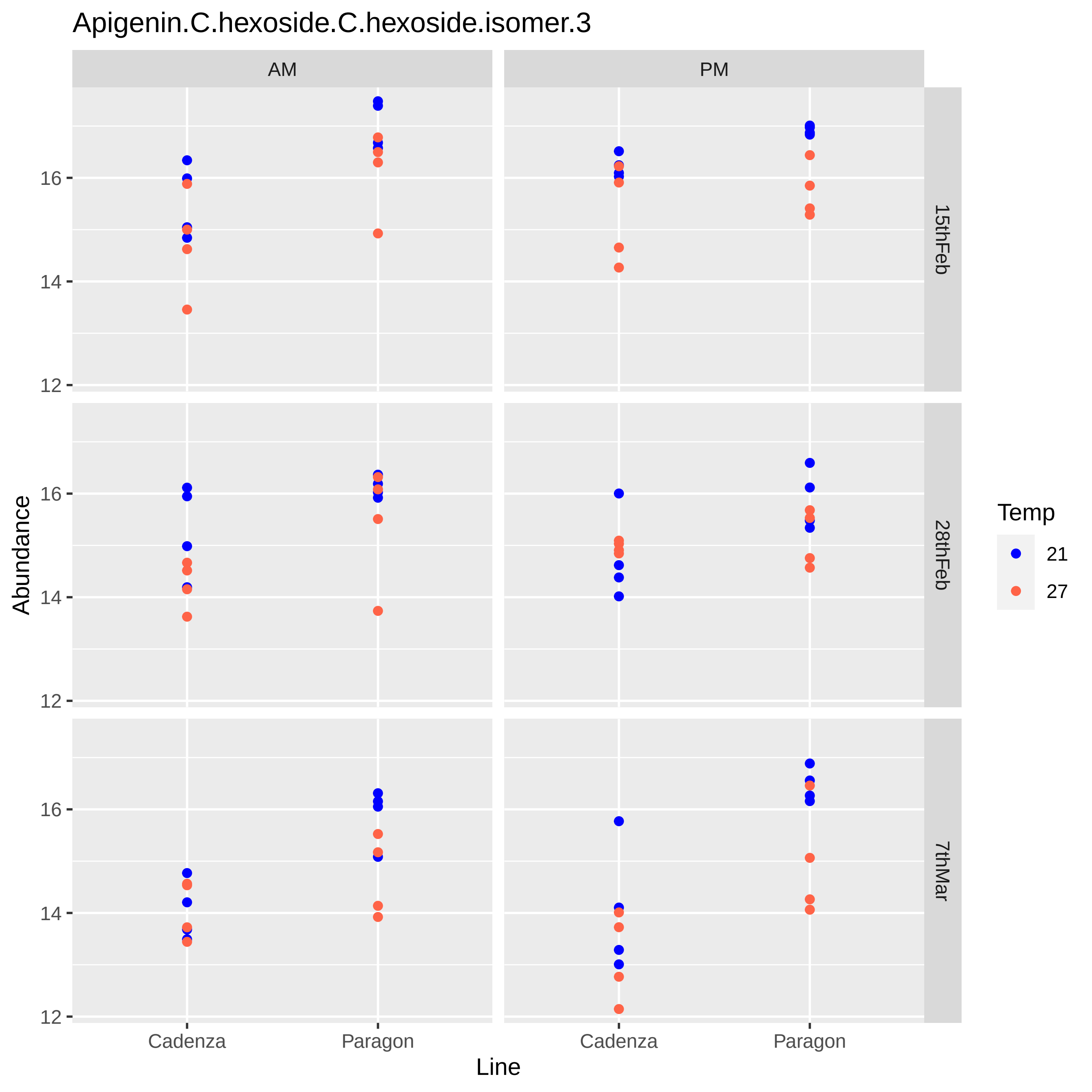
Apigenin.C.hexoside.C.hexoside.isomer.3
AM
PM
15thFeb
28thFeb
7thMar
Abundance
12
14
16
Cadenza
Paragon
Line
Temp
21
27

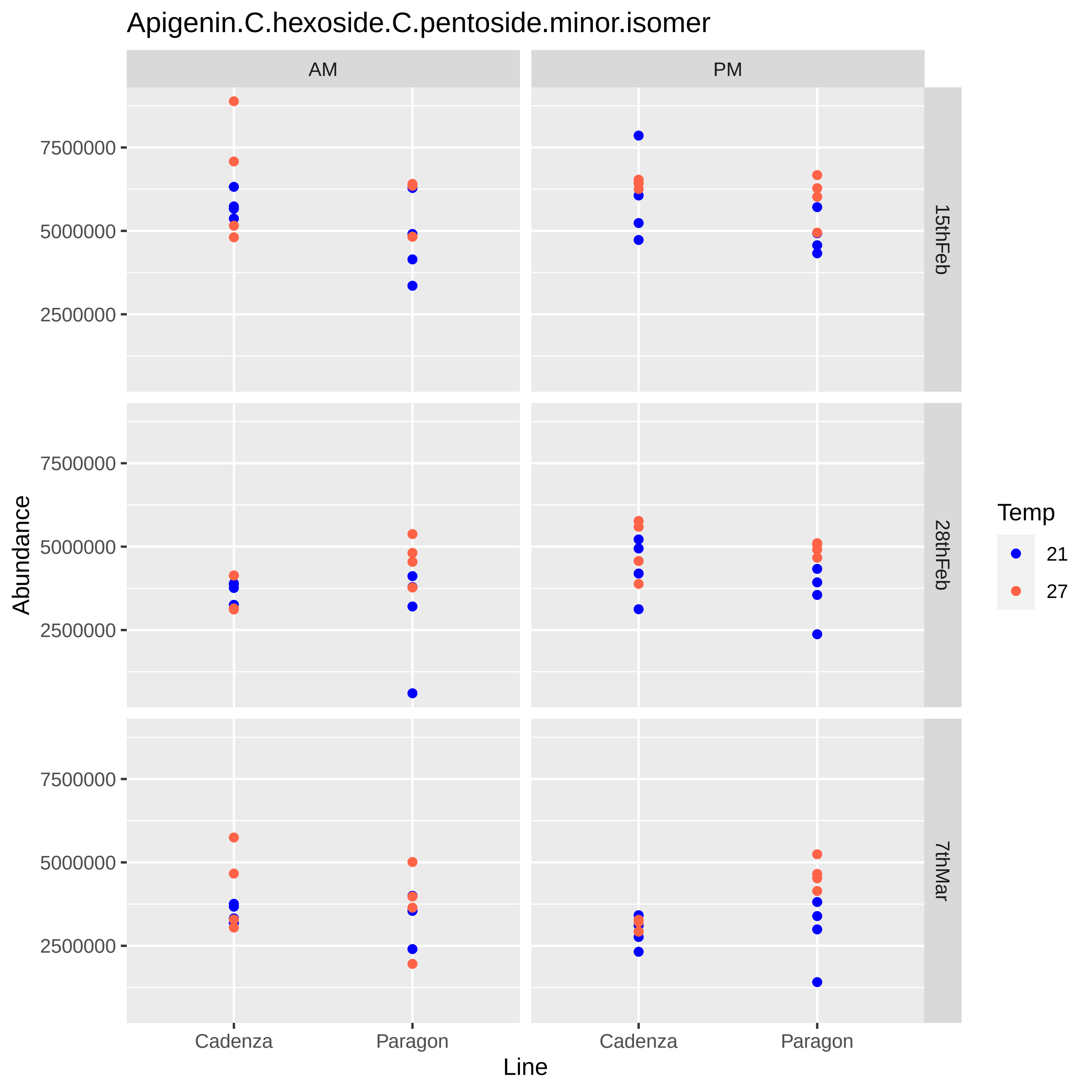

Apigenin.C.hexoside.C.pentoside.minor.isomer
AM
PM
15thFeb
28thFeb
7thMar
Abundance
7500000
5000000
2500000
Cadenza
Paragon
Line
Temp
21
27

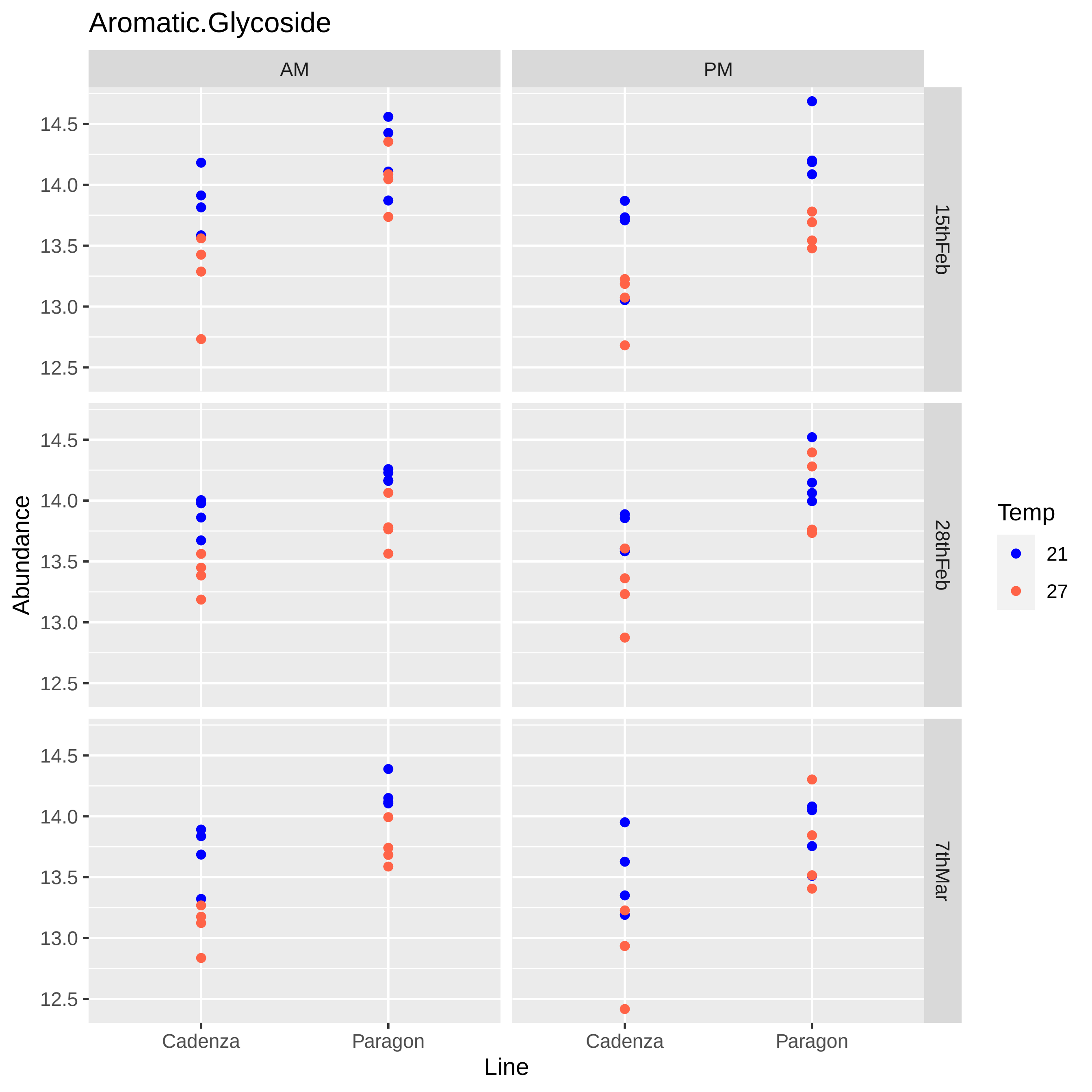

Aromatic.Glycoside
AM
PM
15thFeb
28thFeb
7thMar
Abundance
12.5
13.0
13.5
14.0
14.5
Cadenza
Paragon
Line
Temp
21
27

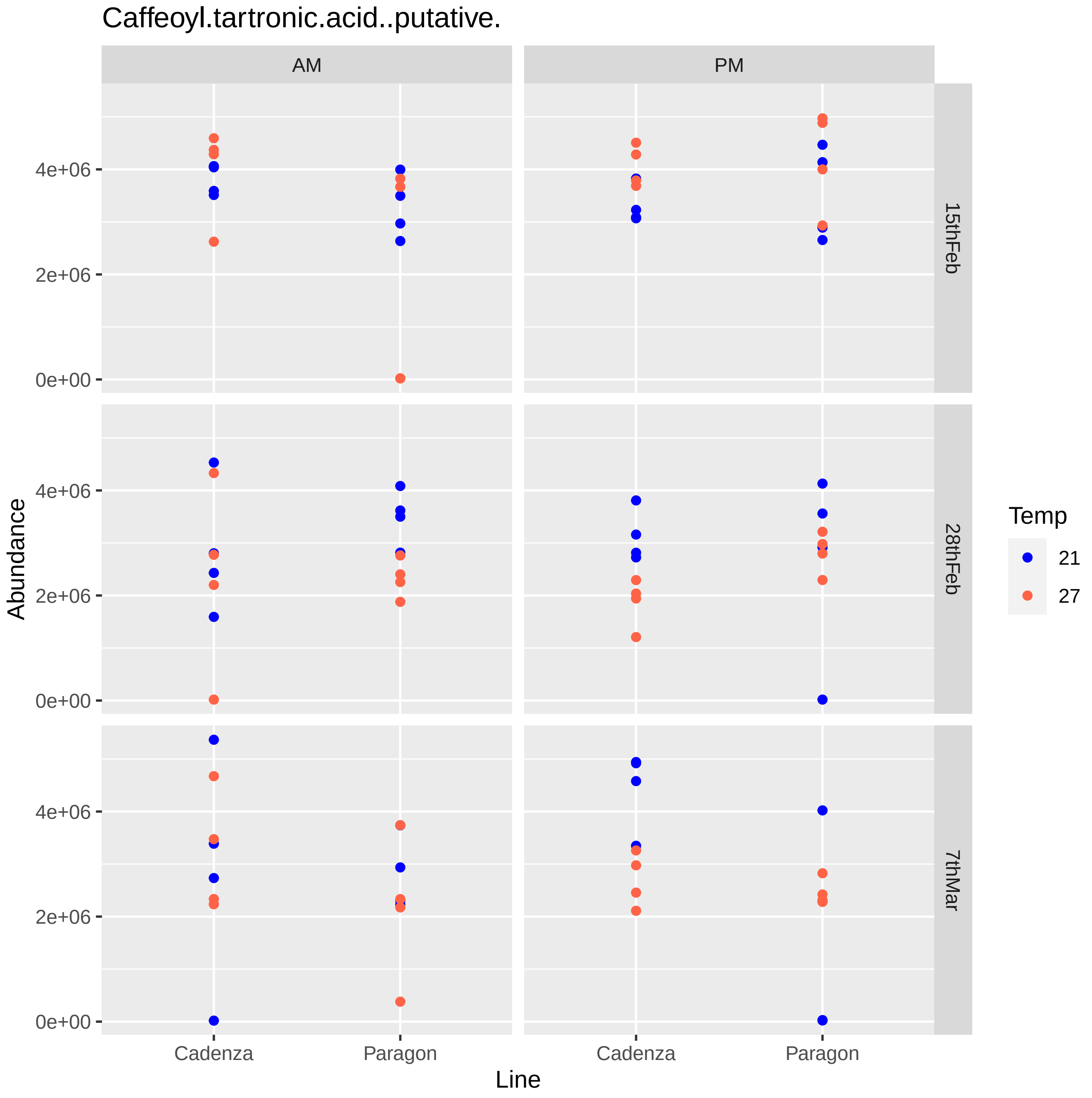

Caffeoyl.tartronic.acid..putative.
AM
PM
15thFeb
28thFeb
7thMar
Abundance
4e+06
2e+06
0e+00
Cadenza
Paragon
Line
Temp
21
27

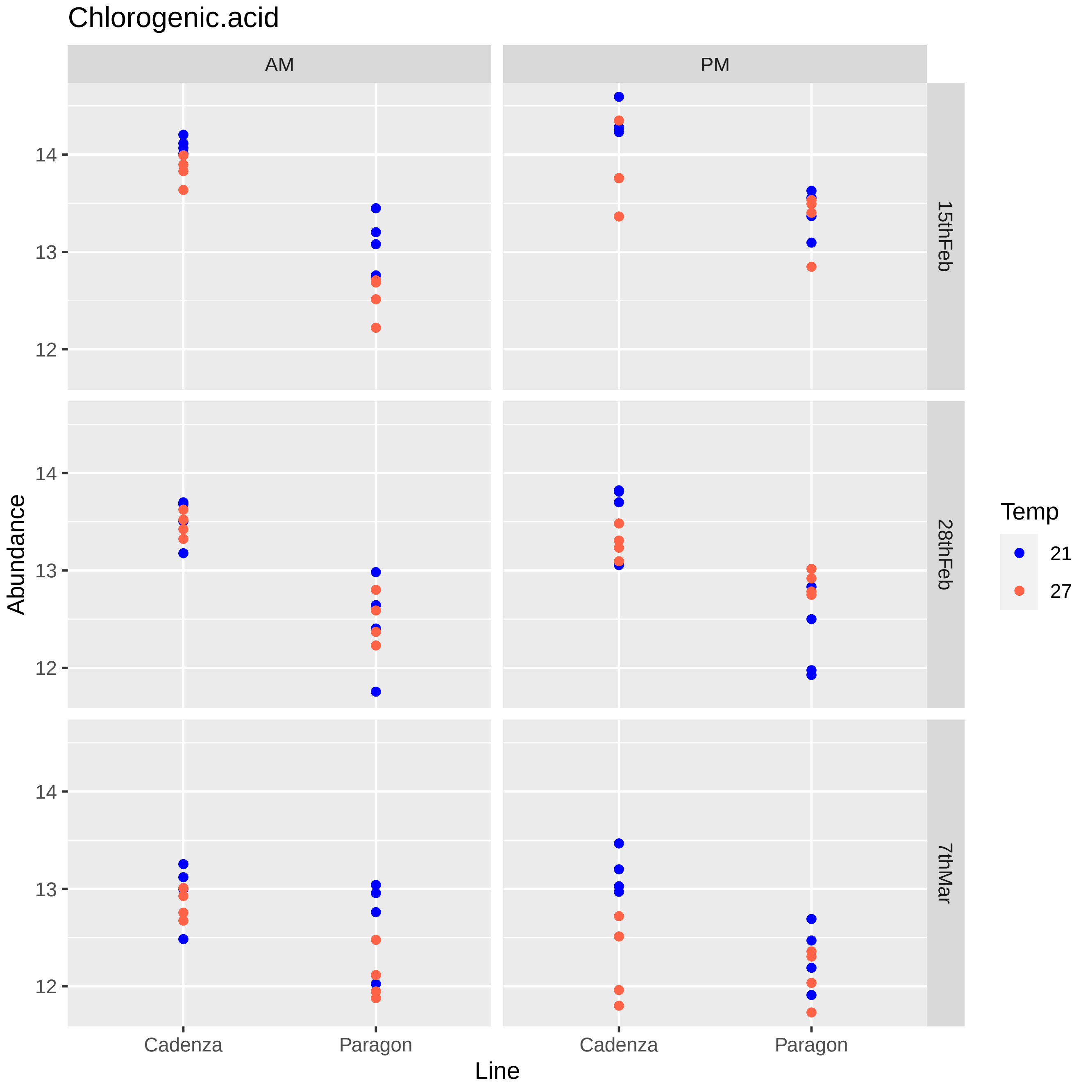
Chlorogenic.acid
AM
PM
15thFeb
28thFeb
7thMar
Abundance
14
13
12
Cadenza
Paragon
Line
Temp
21
27

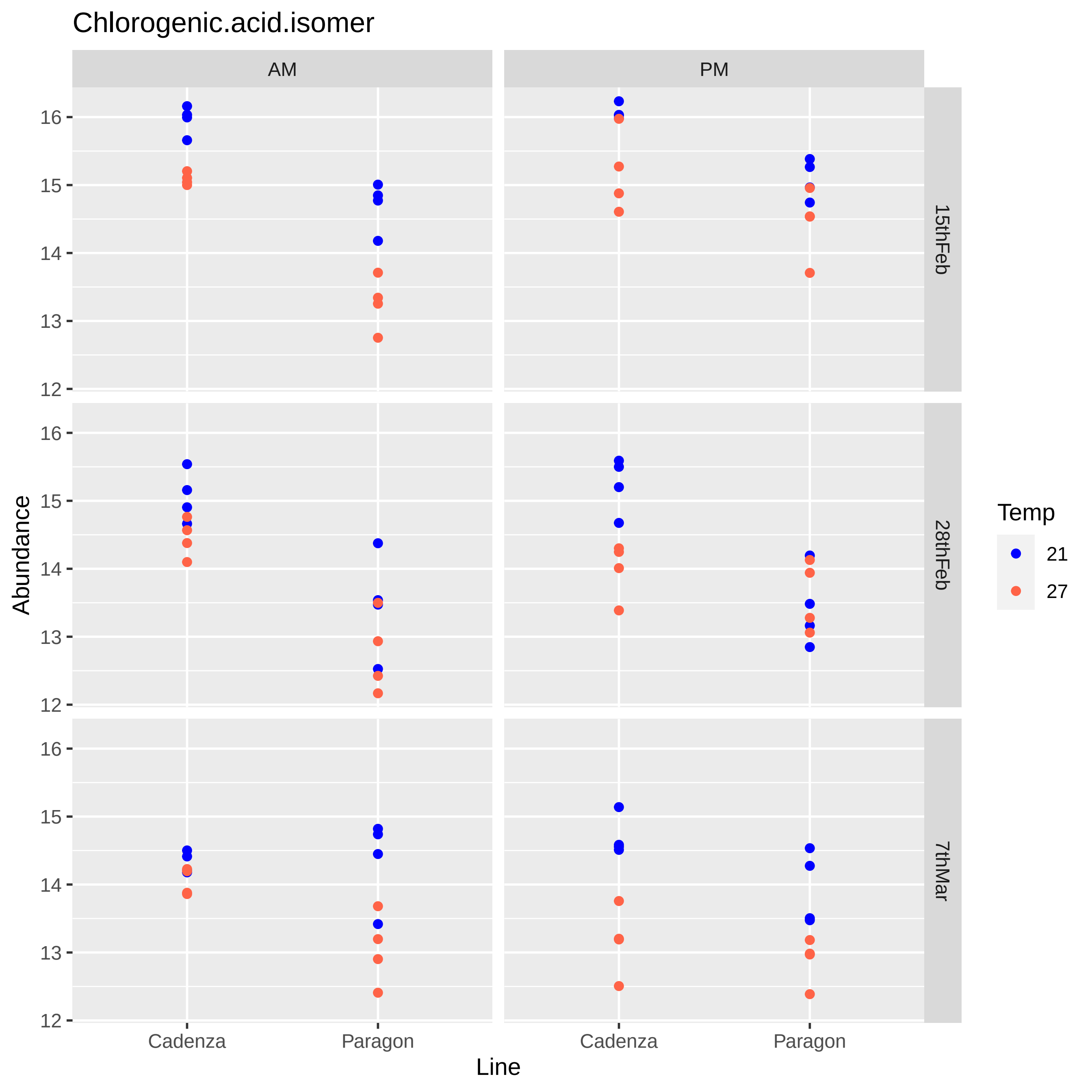

Chlorogenic.acid.isomer
AM
PM
15thFeb
28thFeb
7thMar
Abundance
Line
Cadenza
Paragon
12
13
14
15
16
Temp
21
27

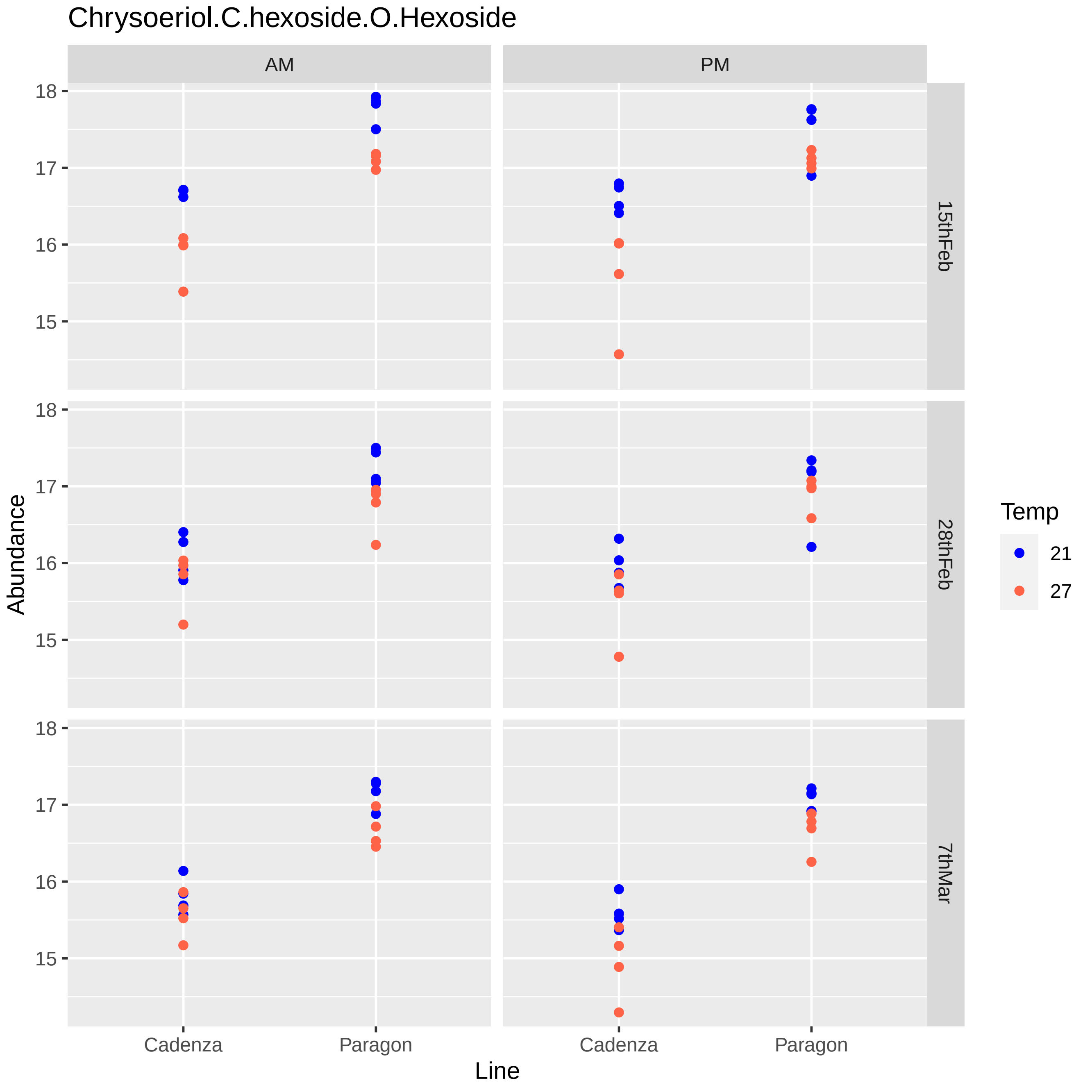

Chrysoeriol.C.hexoside.O.Hexoside
AM
PM
15thFeb
28thFeb
7thMar
Abundance
18
17
16
15
Cadenza
Paragon
Line
Temp
21
27

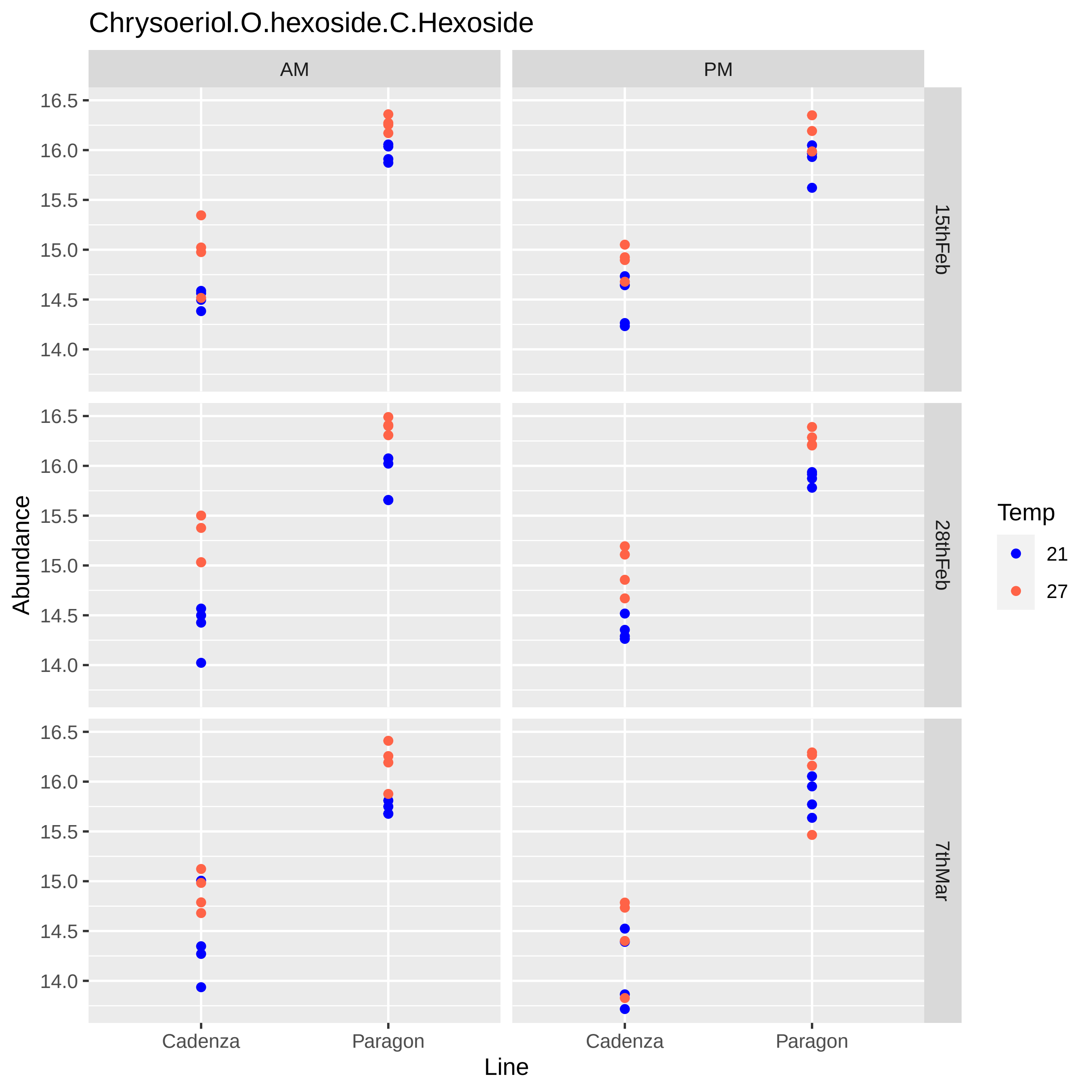

Chrysoeriol.O.hexoside.C.Hexoside
AM
PM
15thFeb
28thFeb
7thMar
16.5
16.0
15.5
15.0
14.5
14.0
Abundance
Cadenza
Paragon
Line
Temp
21
27

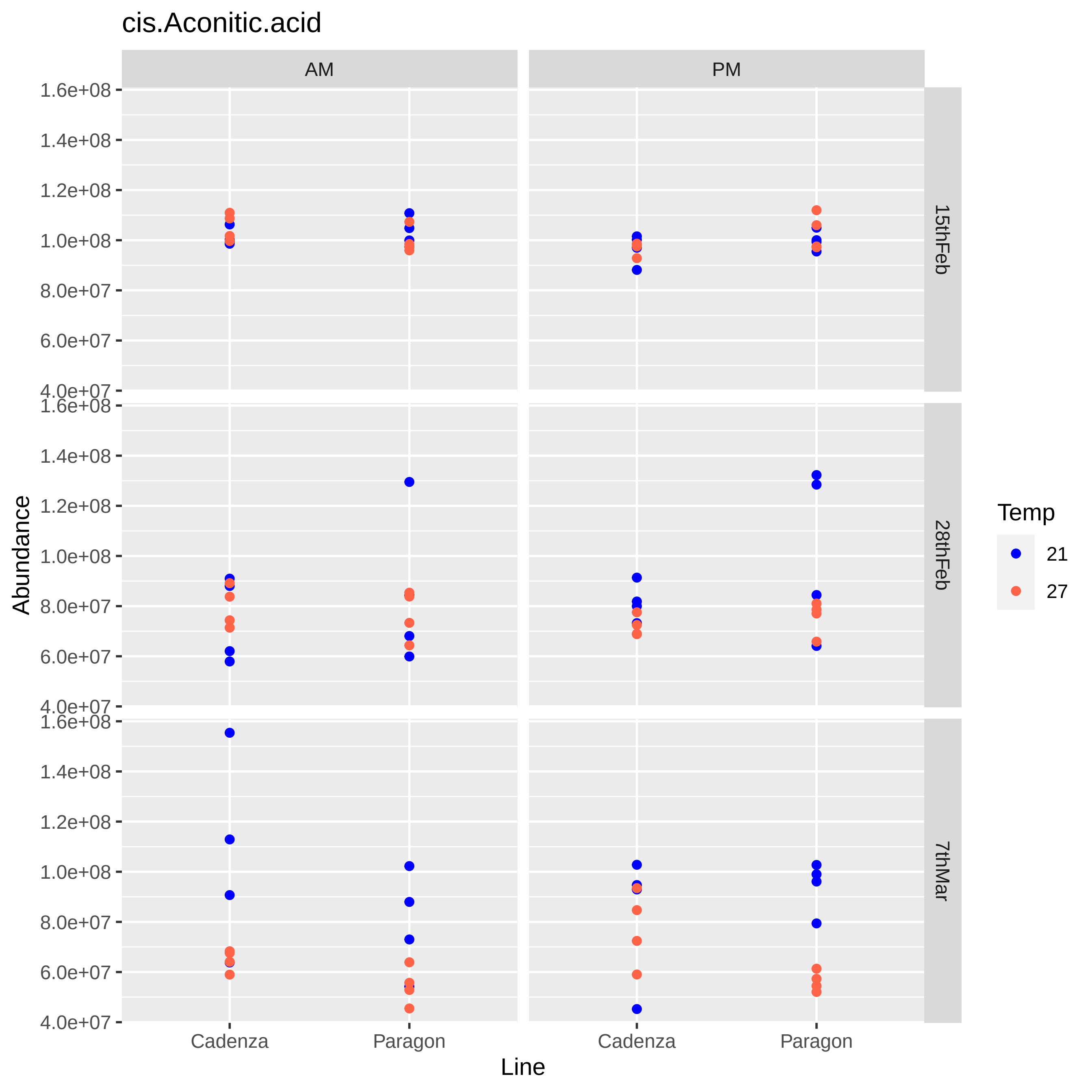
cis.Aconitic.acid
AM
PM
15thFeb
28thFeb
7thMar
Abundance
1.6e+08
1.4e+08
1.2e+08
1.0e+08
8.0e+07
6.0e+07
4.0e+07
Cadenza
Paragon
Line
Temp
21
27

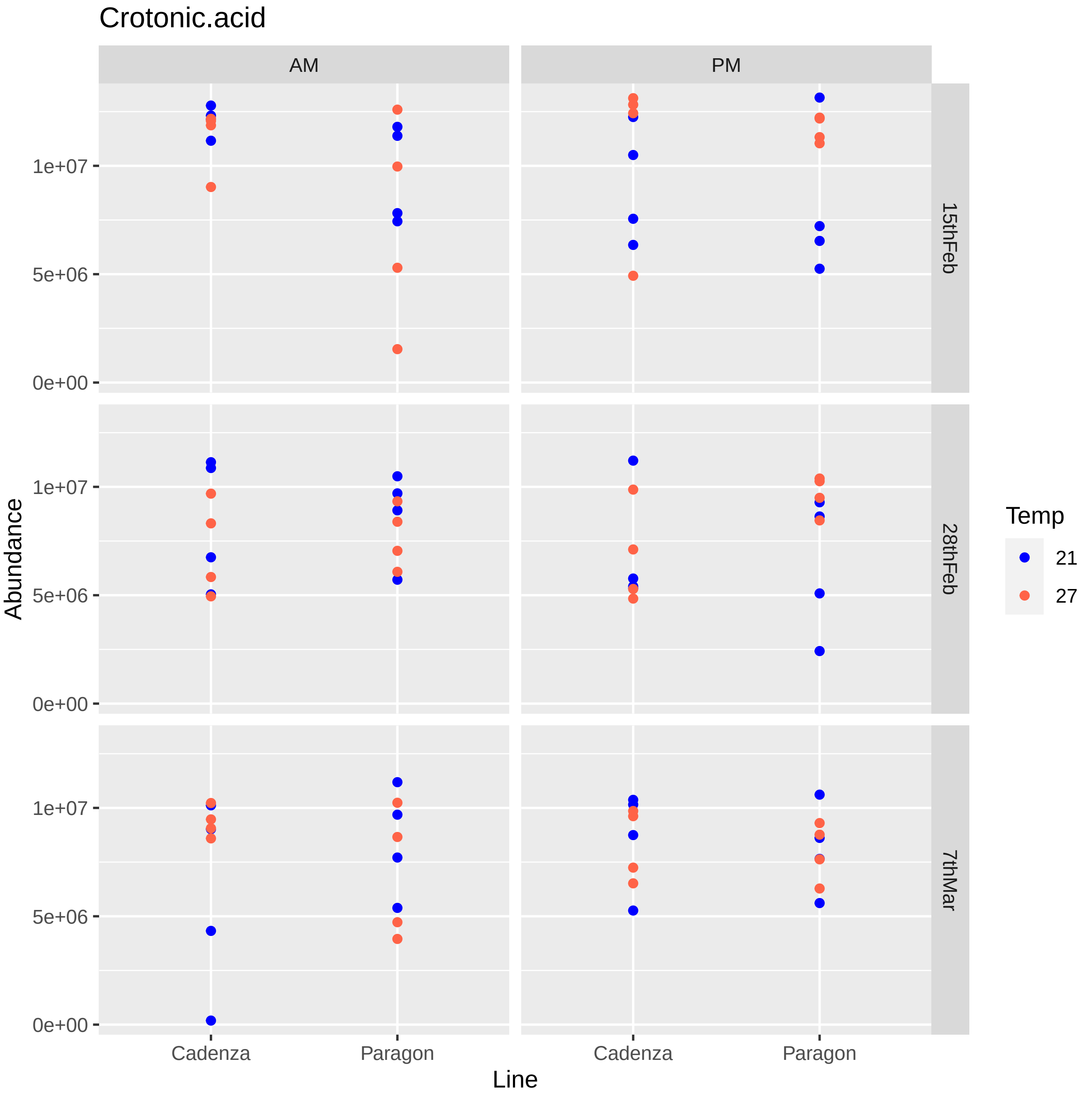

Crotonic.acid
AM
PM
15thFeb
28thFeb
7thMar
1e+07
5e+06
0e+00
Abundance
Cadenza
Paragon
Line
Temp
21
27

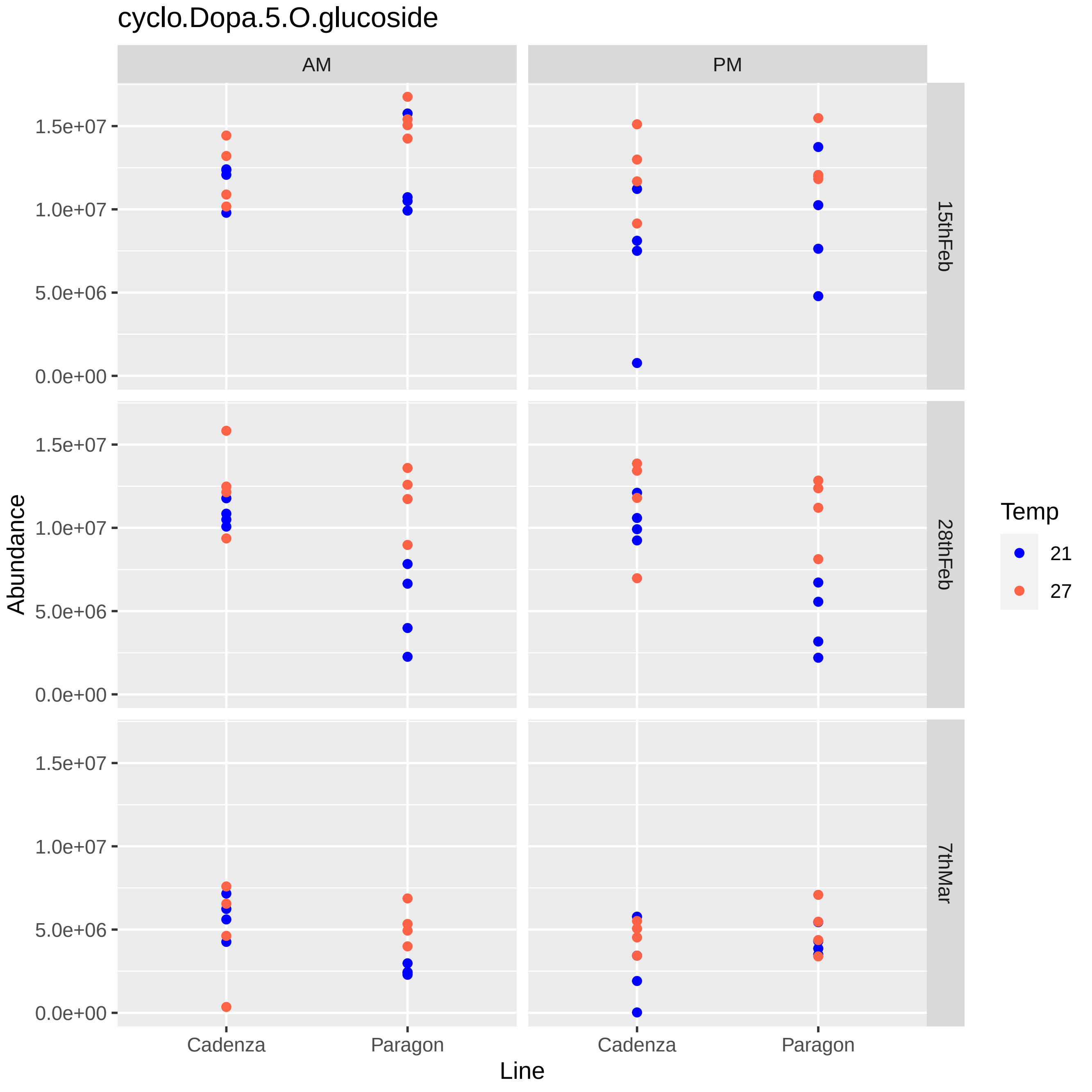
cyclo.Dopa.5.O.glucoside
AM
PM
15thFeb
28thFeb
7thMar
Abundance
0.0e+00
5.0e+06
1.0e+07
1.5e+07
Cadenza
Paragon
Line
Temp
21
27

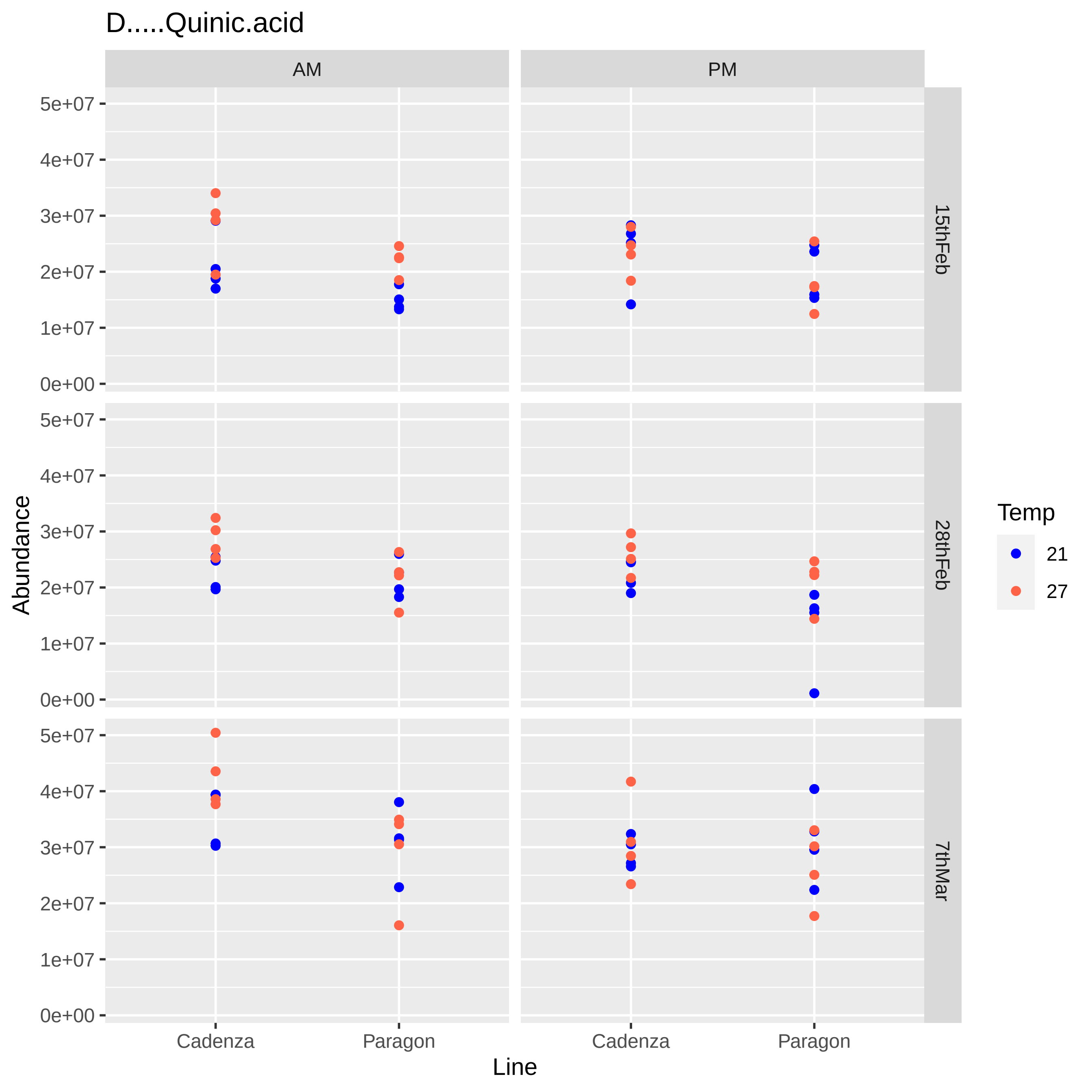

D.....Quinic.acid
AM
PM
15thFeb
28thFeb
7thMar
Abundance
5e+07
4e+07
3e+07
2e+07
1e+07
0e+00
Cadenza
Paragon
Line
Temp
21
27

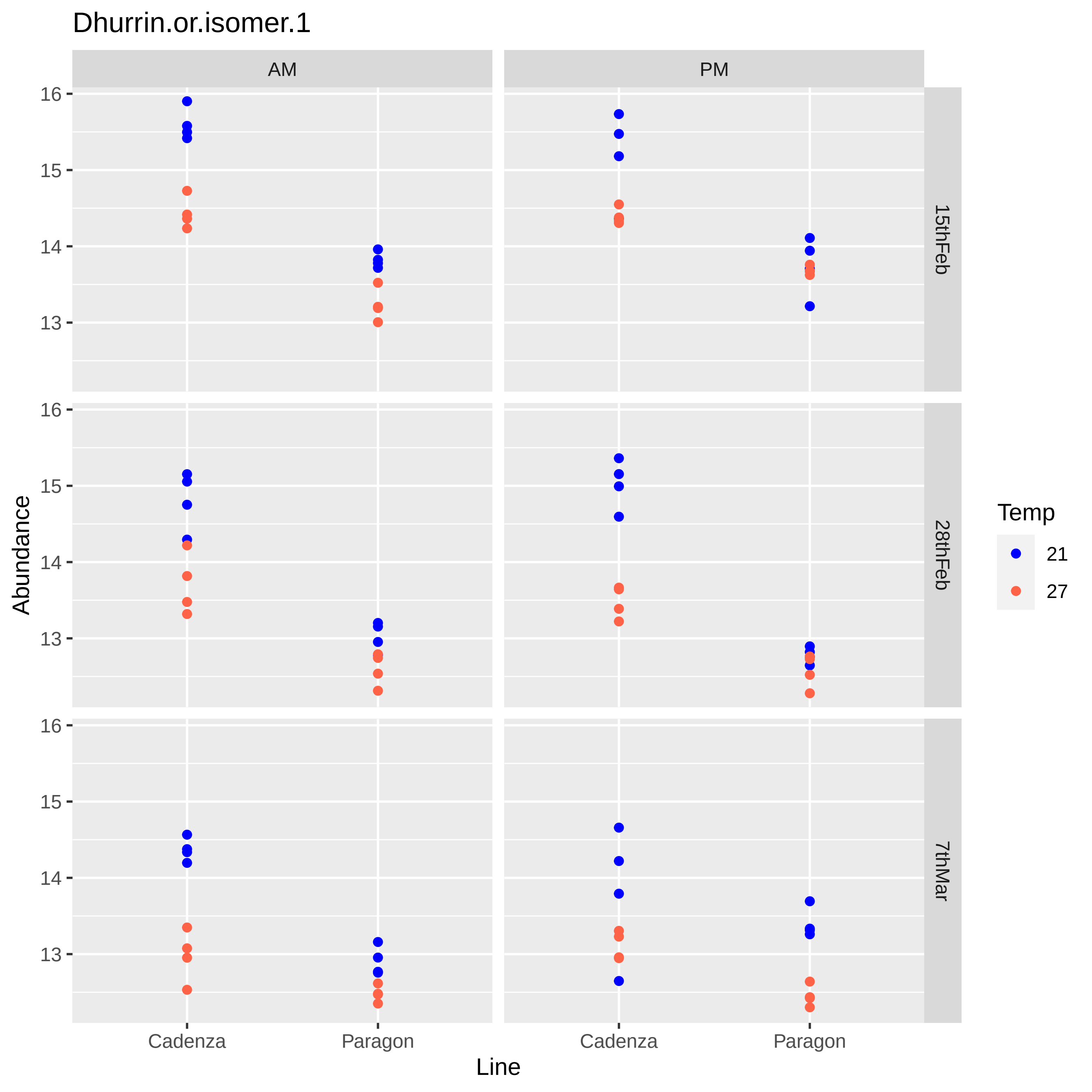

Dhurrin.or.isomer.1
AM
PM
15thFeb
28thFeb
7thMar
Abundance
16
15
14
13
Cadenza
Paragon
Cadenza
Paragon
Line
Temp
21
27

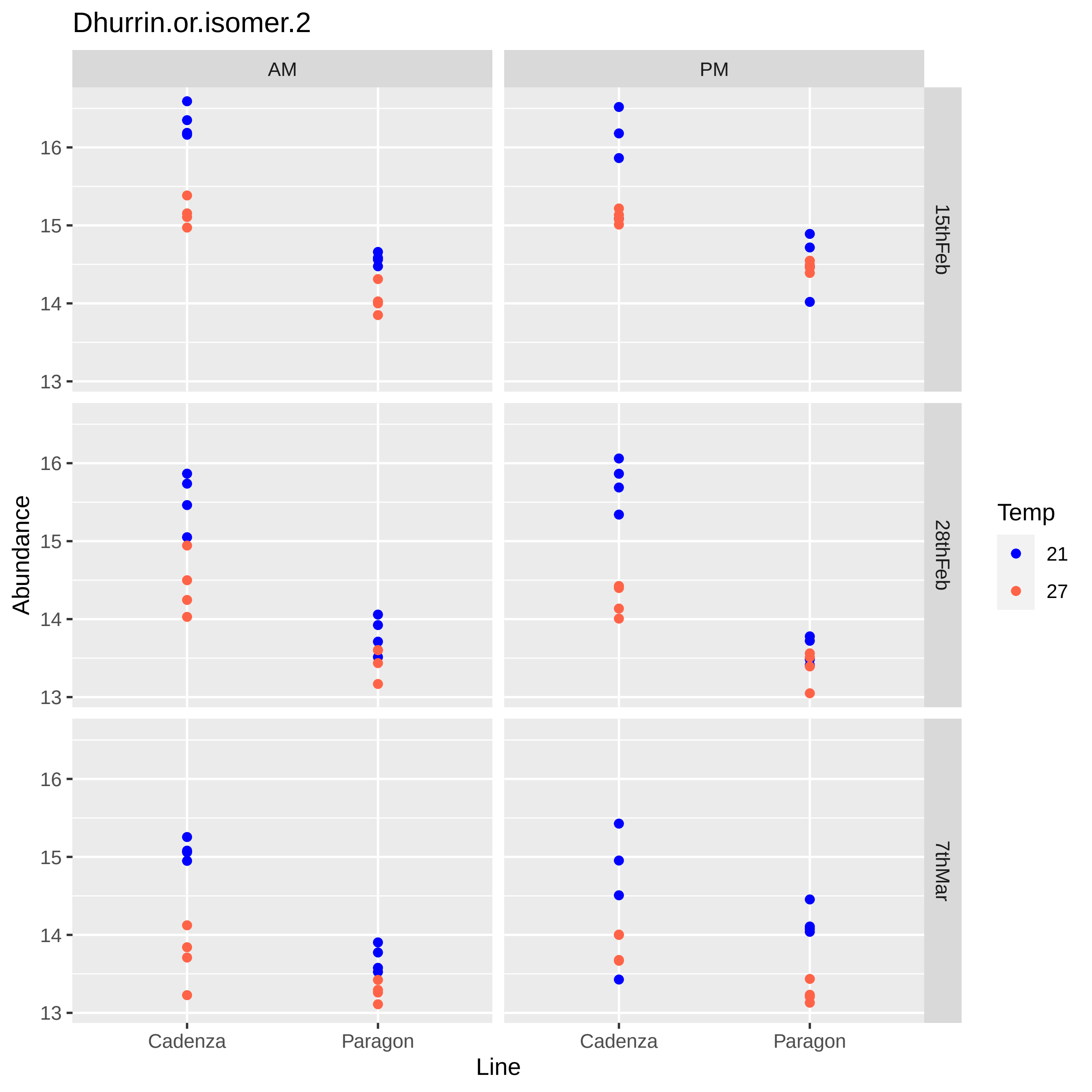

Dhurrin.or.isomer.2
AM
PM
15thFeb
28thFeb
7thMar
Abundance
16
15
14
13
Cadenza
Paragon
Line
Temp
21
27

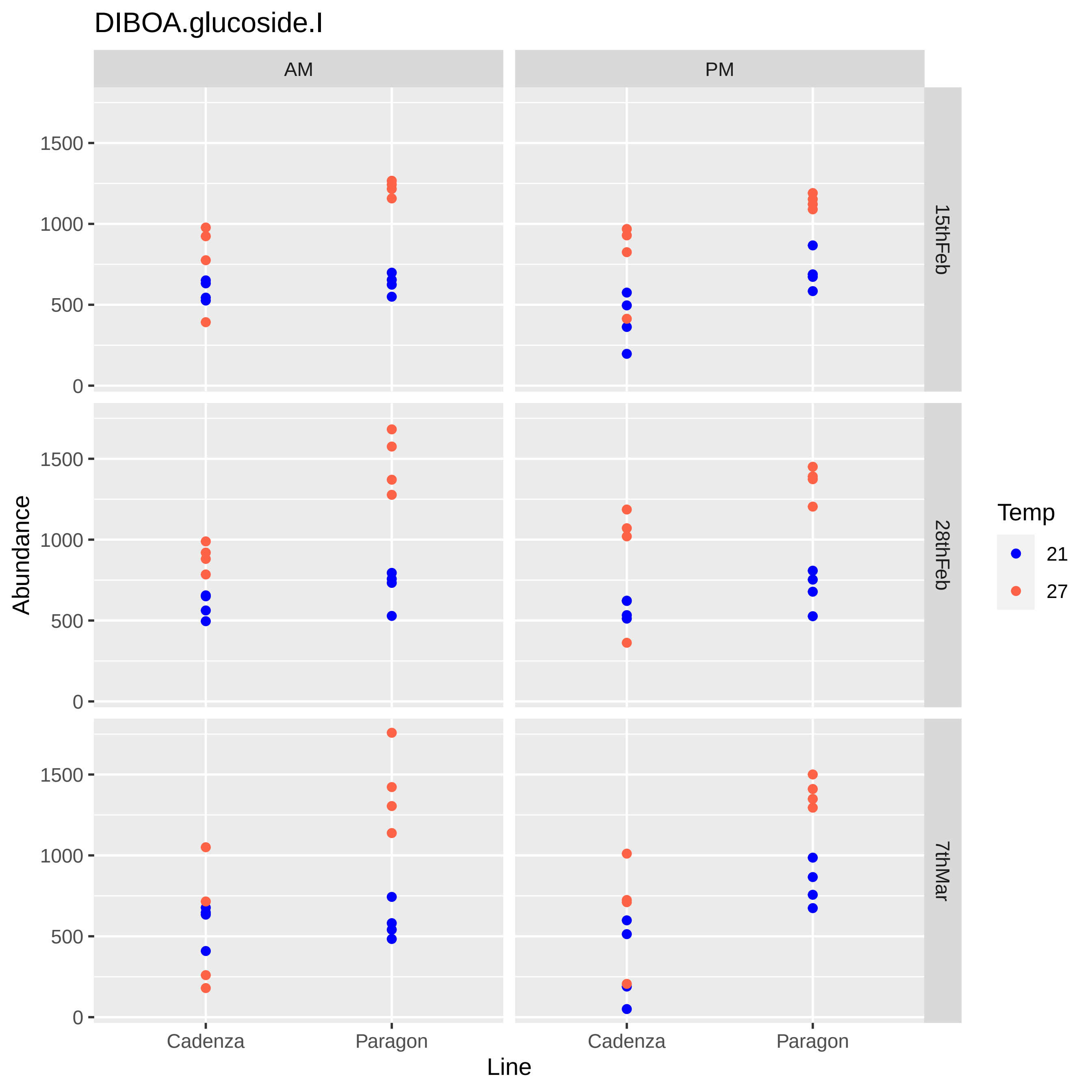

DIBOA.glucoside.I
AM
PM
15thFeb
28thFeb
7thMar
0
500
1000
1500
Abundance
Cadenza
Paragon
Line
Temp
21
27

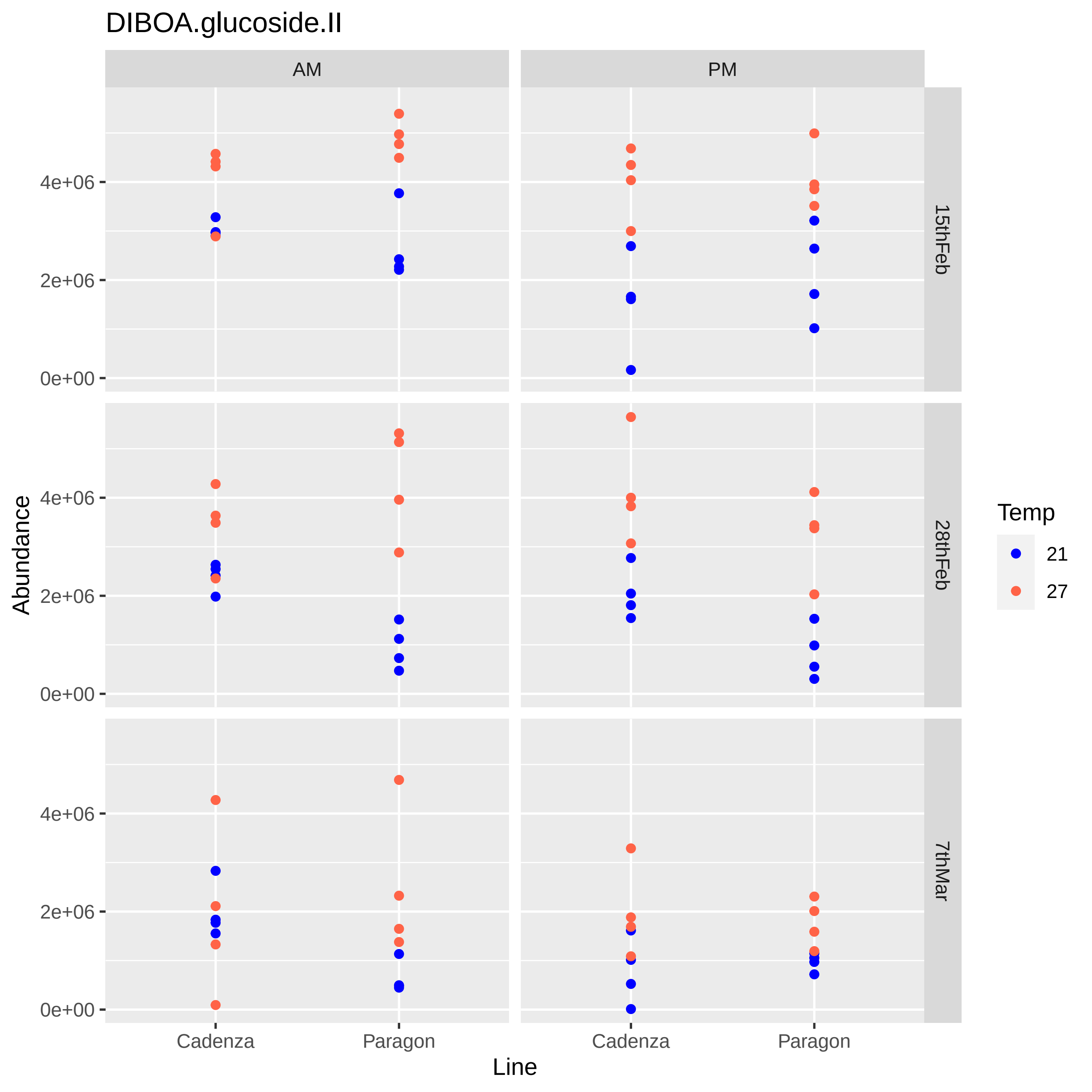

DIBOA.glucoside.II
AM
PM
15thFeb
28thFeb
7thMar
4e+06
2e+06
0e+00
Abundance
Cadenza
Paragon
Line
Temp
21
27

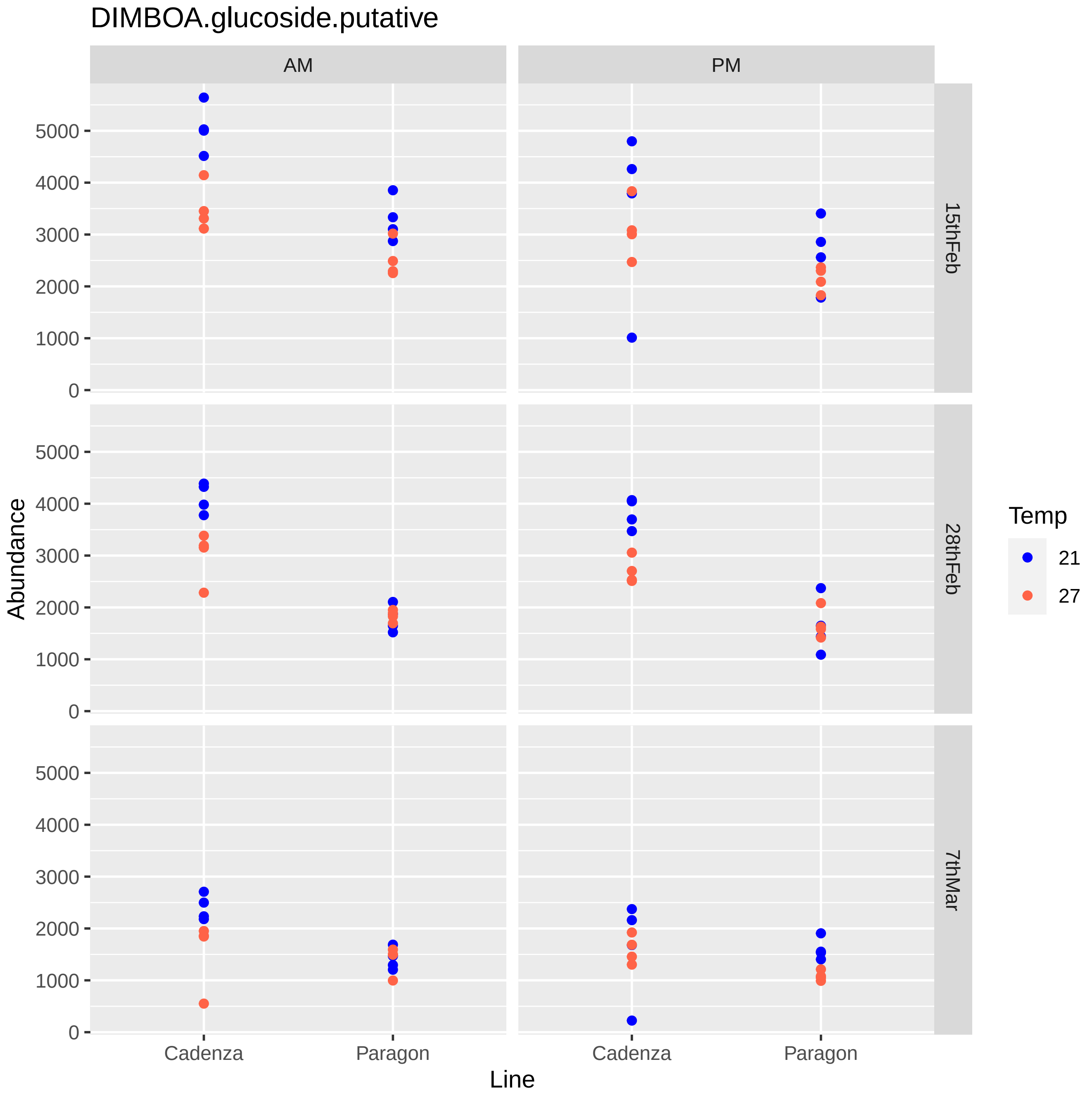
DIMBOA.glucoside.putative
AM
PM
15thFeb
28thFeb
7thMar
Abundance
0
1000
2000
3000
4000
5000
Cadenza
Paragon
Line
Temp
21
27

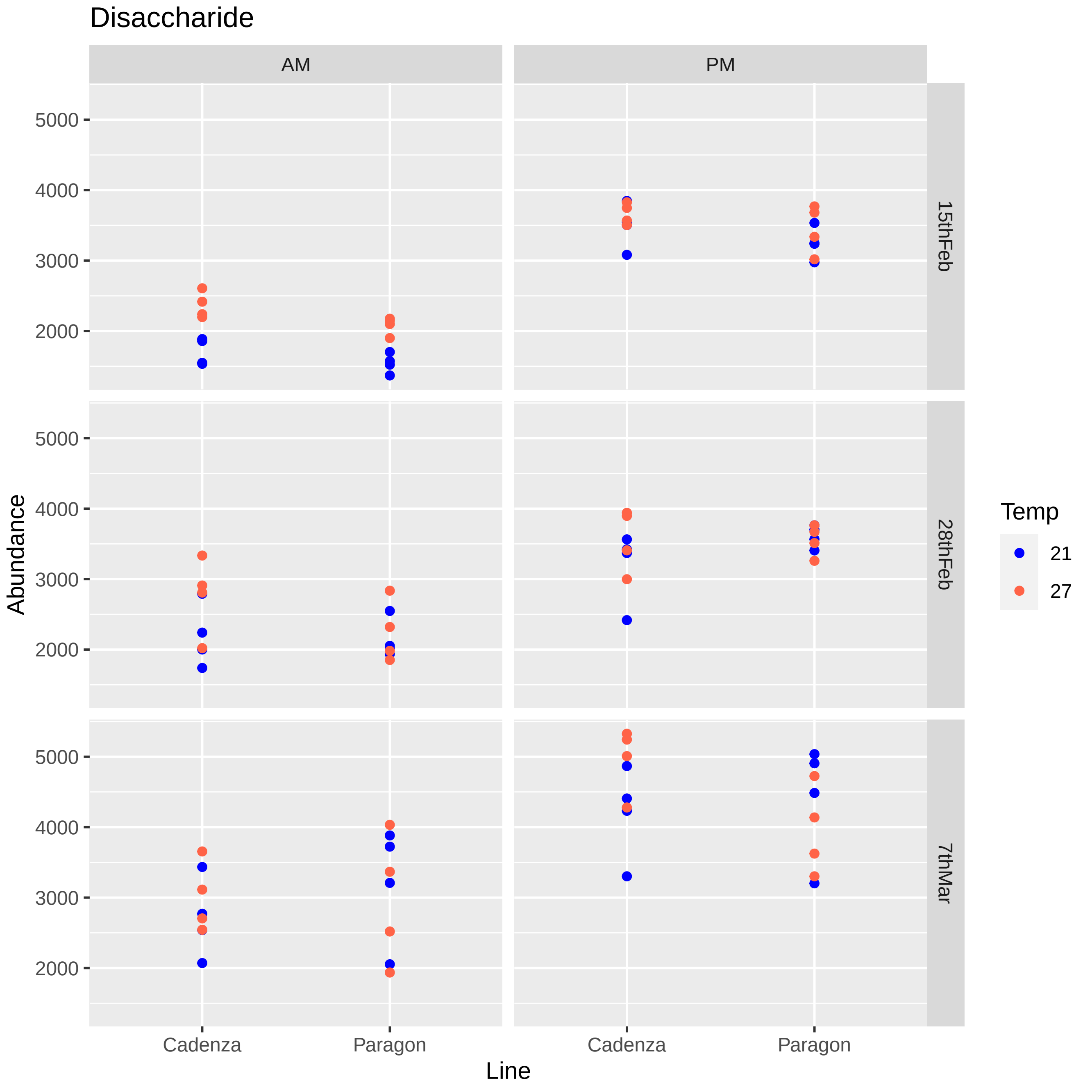
Disaccharide
AM
PM
15thFeb
28thFeb
7thMar
5000
4000
3000
2000
Abundance
Cadenza
Paragon
Line
Temp
21
27

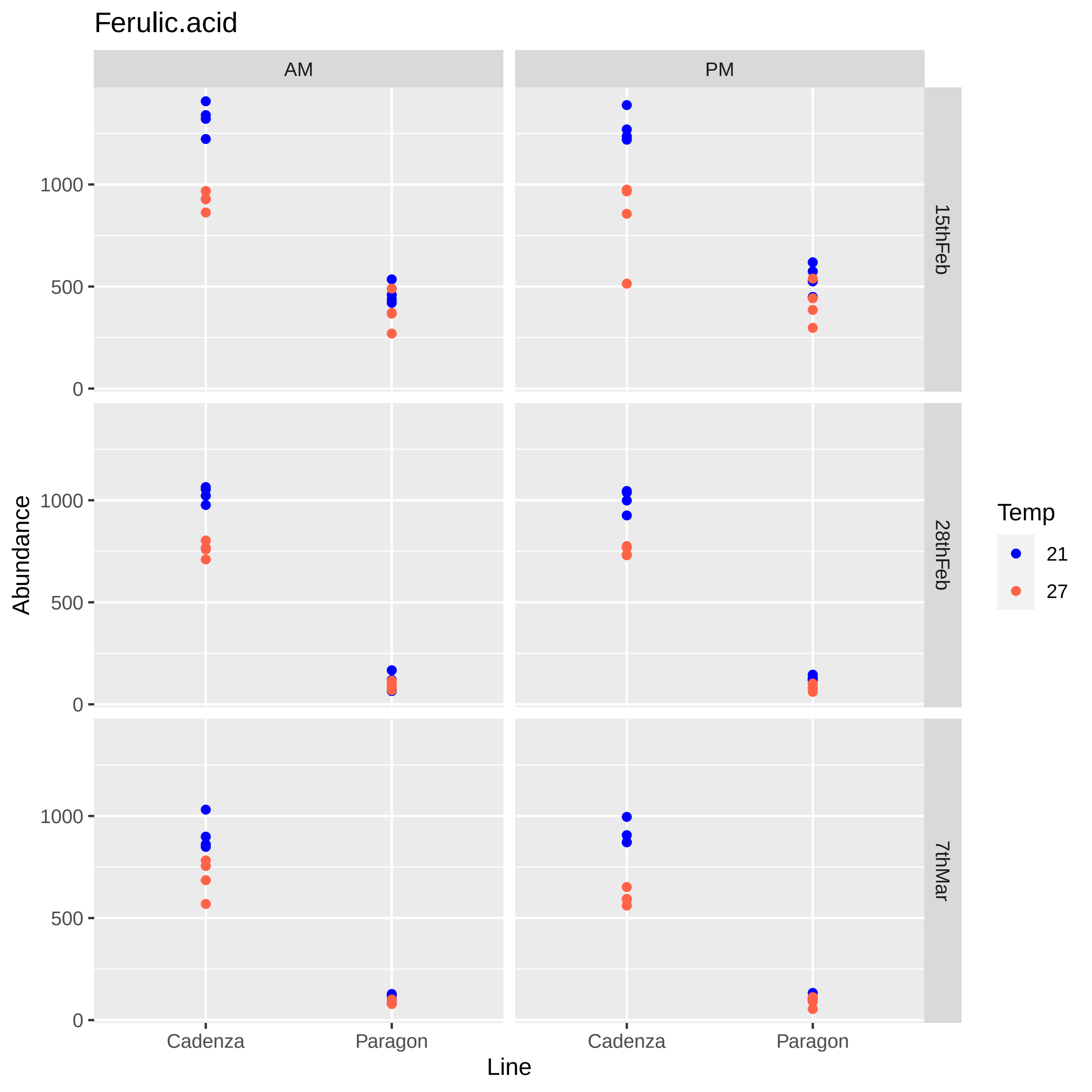
Ferulic.acid
AM
PM
15thFeb
28thFeb
7thMar
Abundance
0
500
1000
Cadenza
Paragon
Line
Temp
21
27

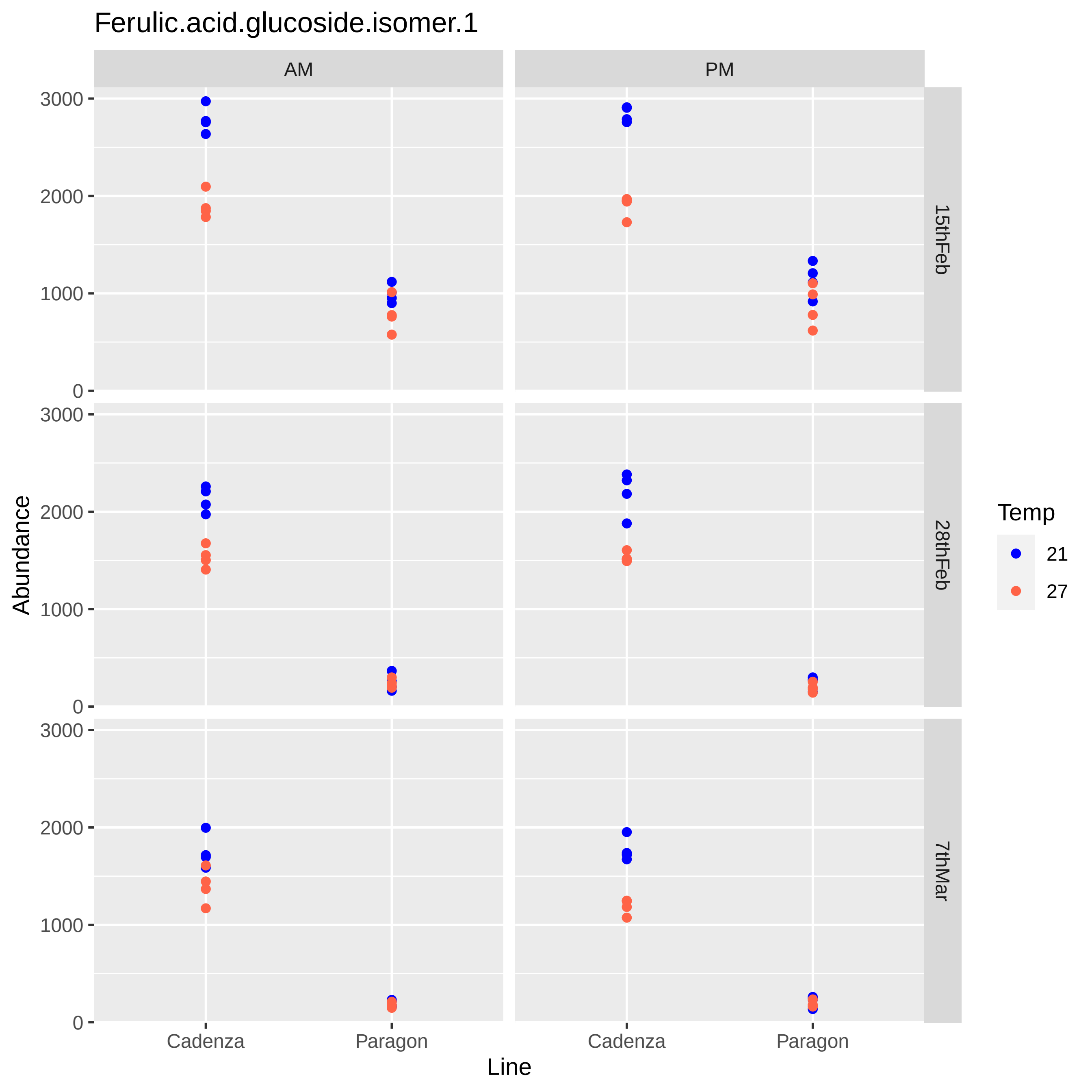

Ferulic.acid.glucoside.isomer.1
AM
PM
15thFeb
28thFeb
7thMar
Abundance
0
1000
2000
3000
Cadenza
Paragon
Line
Temp
21
27

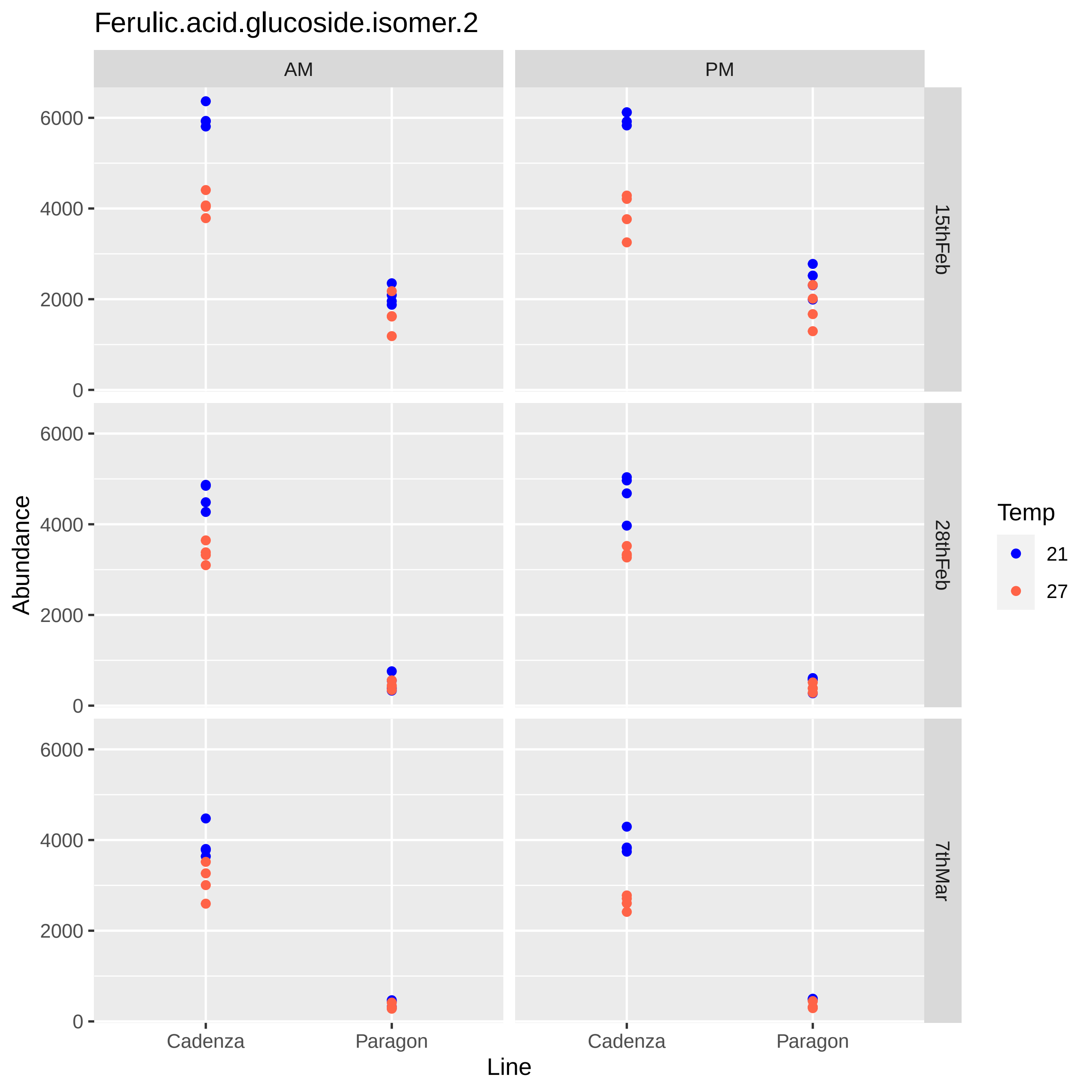

Ferulic.acid.glucoside.isomer.2
AM
PM
15thFeb
28thFeb
7thMar
Abundance
6000
4000
2000
0
Cadenza
Paragon
Line
Temp
21
27

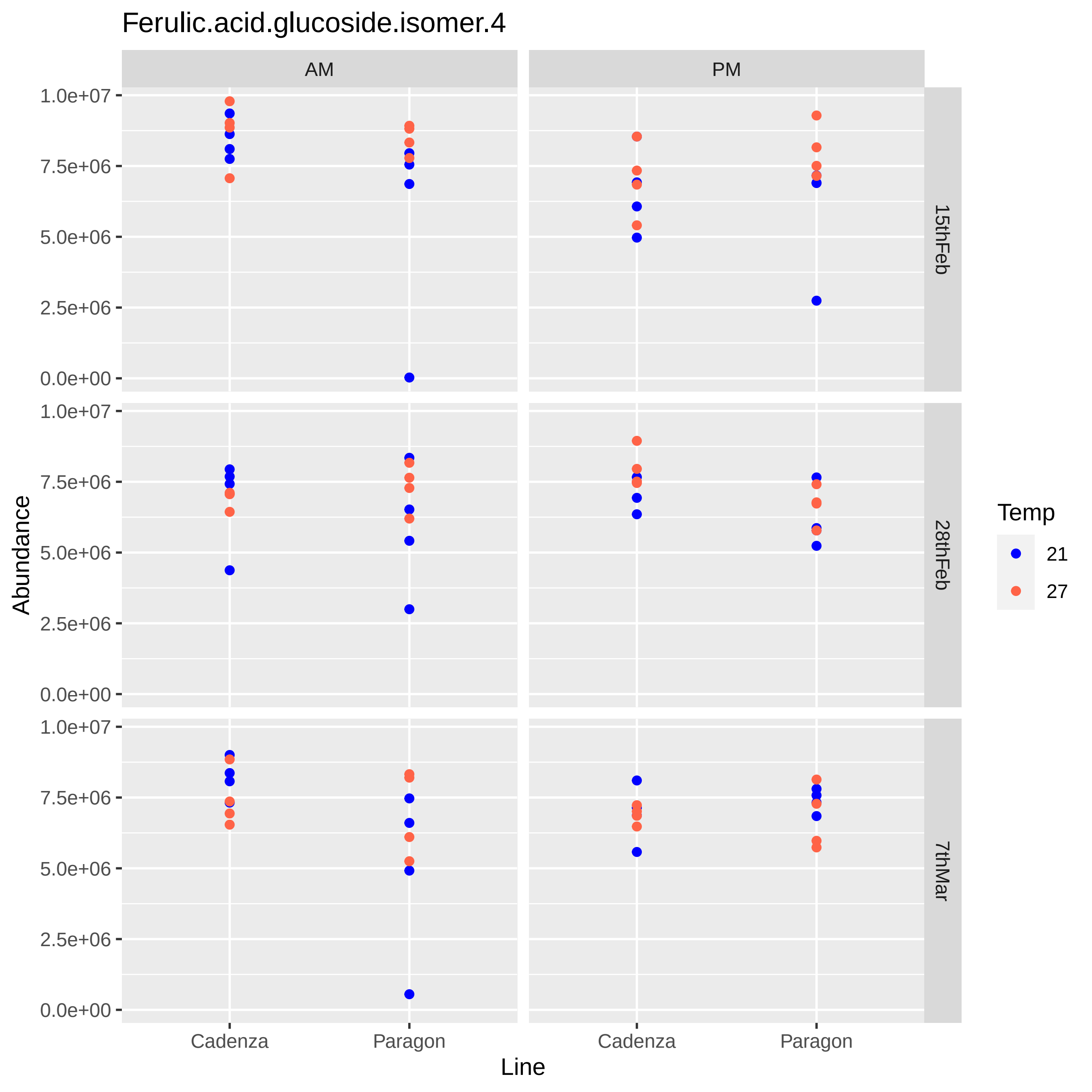
Ferulic.acid.glucoside.isomer.4
AM
PM
15thFeb
28thFeb
7thMar
1.0e+07
7.5e+06
5.0e+06
2.5e+06
0.0e+00
Abundance
Cadenza
Paragon
Line
Temp
21
27

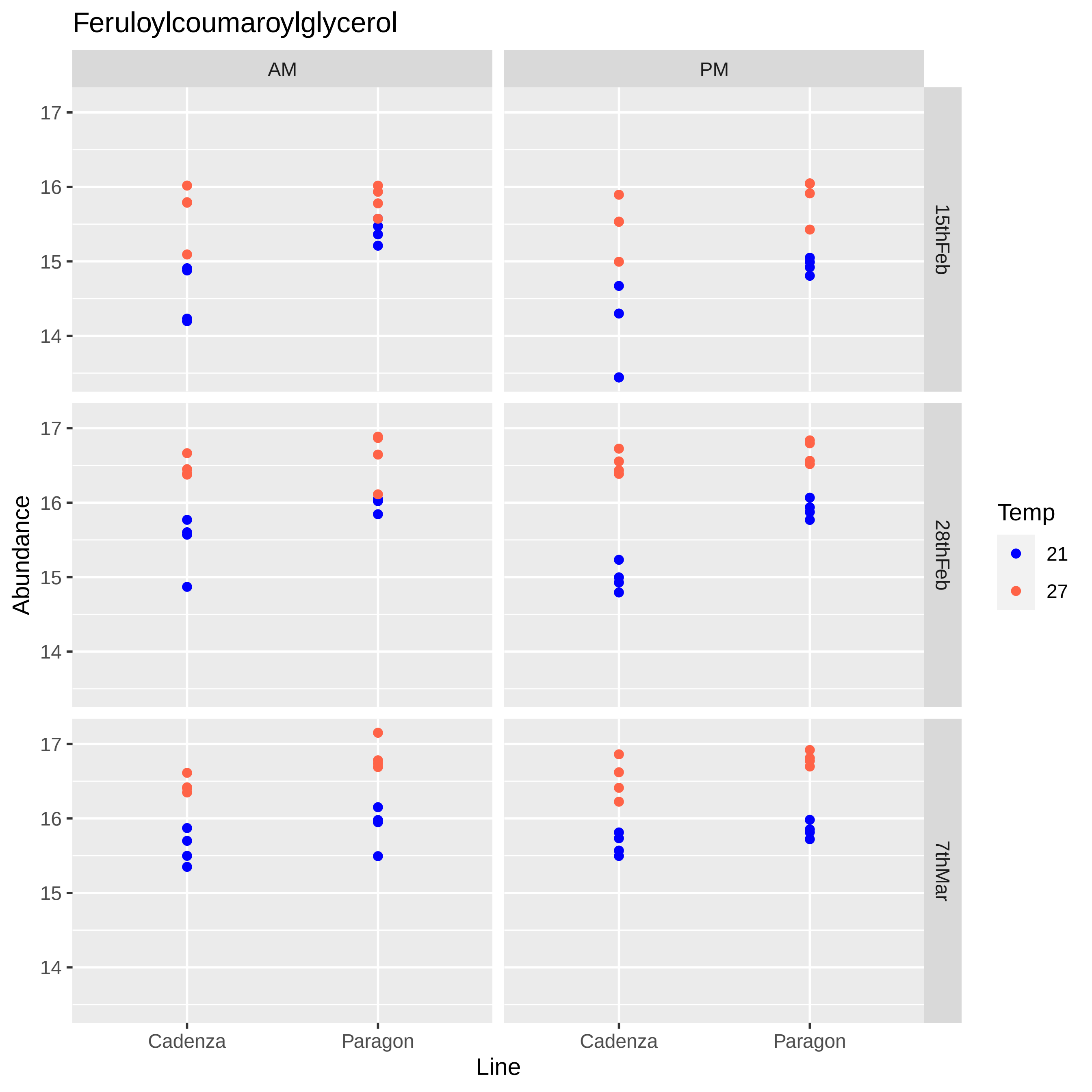

Feruloylcoumaroylglycerol
AM
PM
15thFeb
28thFeb
7thMar
17
16
15
14
Abundance
Cadenza
Paragon
Line
Temp
21
27

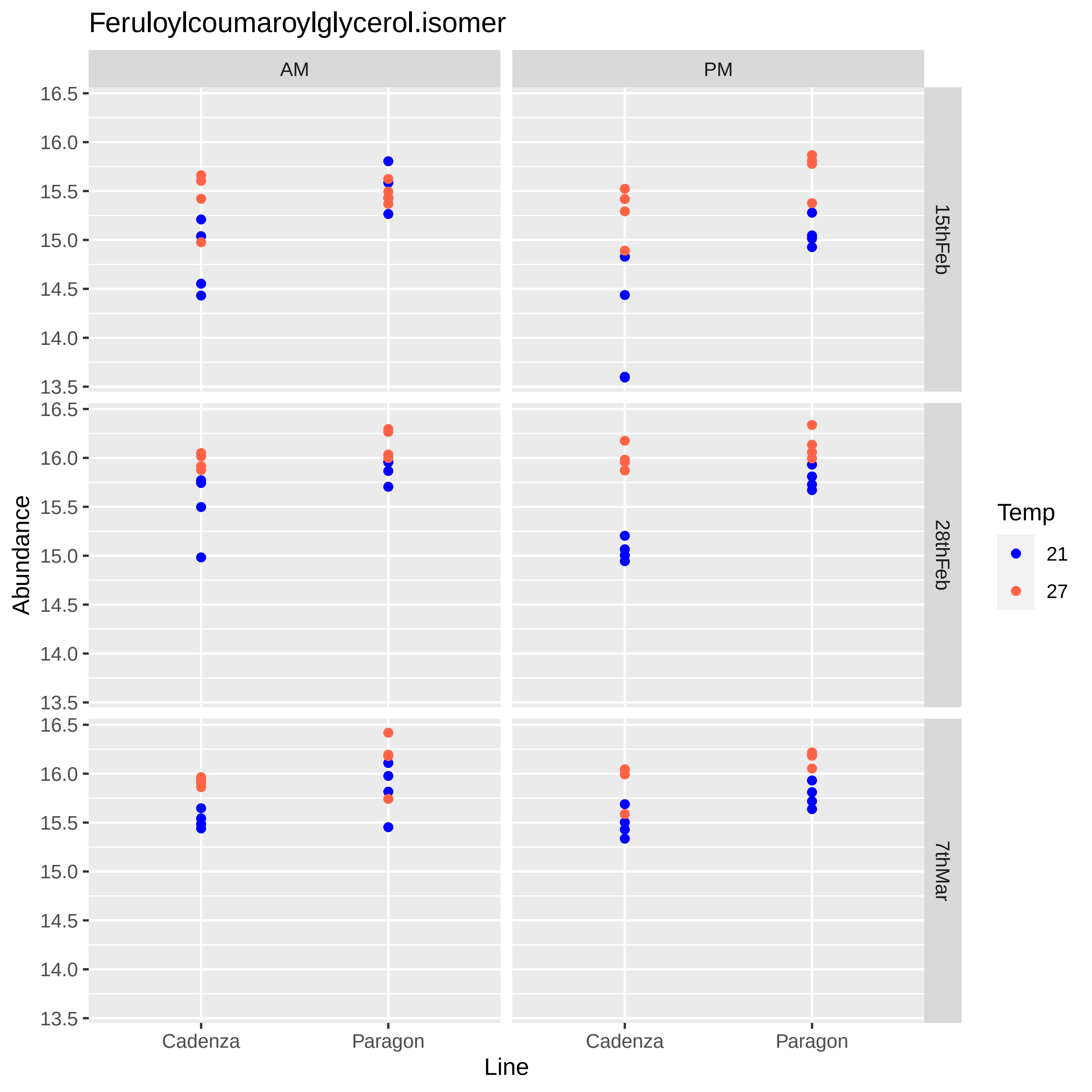
Feruloylcoumaroylglycerol.isomer
AM
PM
15thFeb
28thFeb
7thMar
Abundance
16.5
16.0
15.5
15.0
14.5
14.0
13.5
Line
Cadenza
Paragon
Temp
21
27

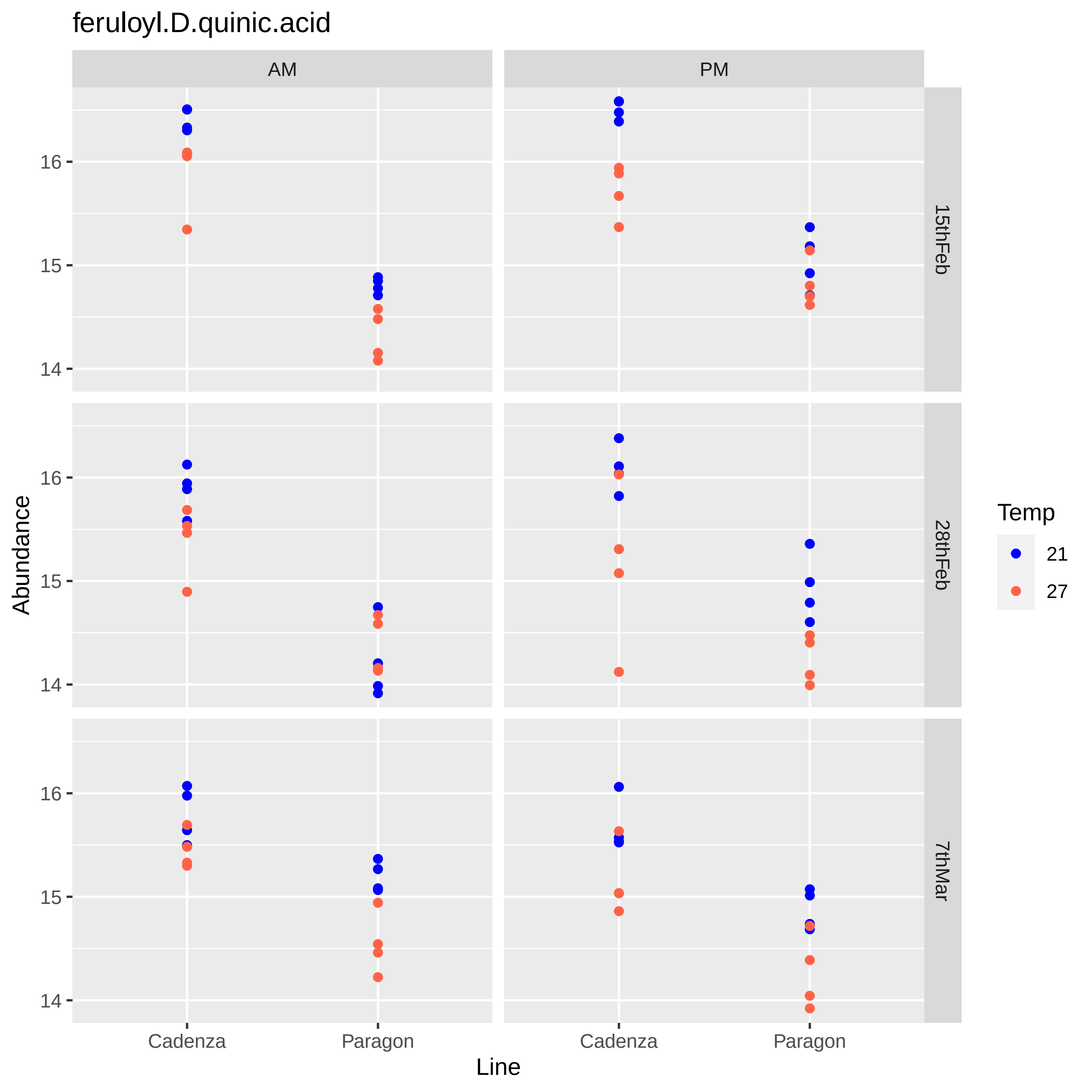

feruloyl.D.quinic.acid
AM
PM
15thFeb
28thFeb
7thMar
16
15
14
Abundance
Cadenza
Paragon
Line
Temp
21
27

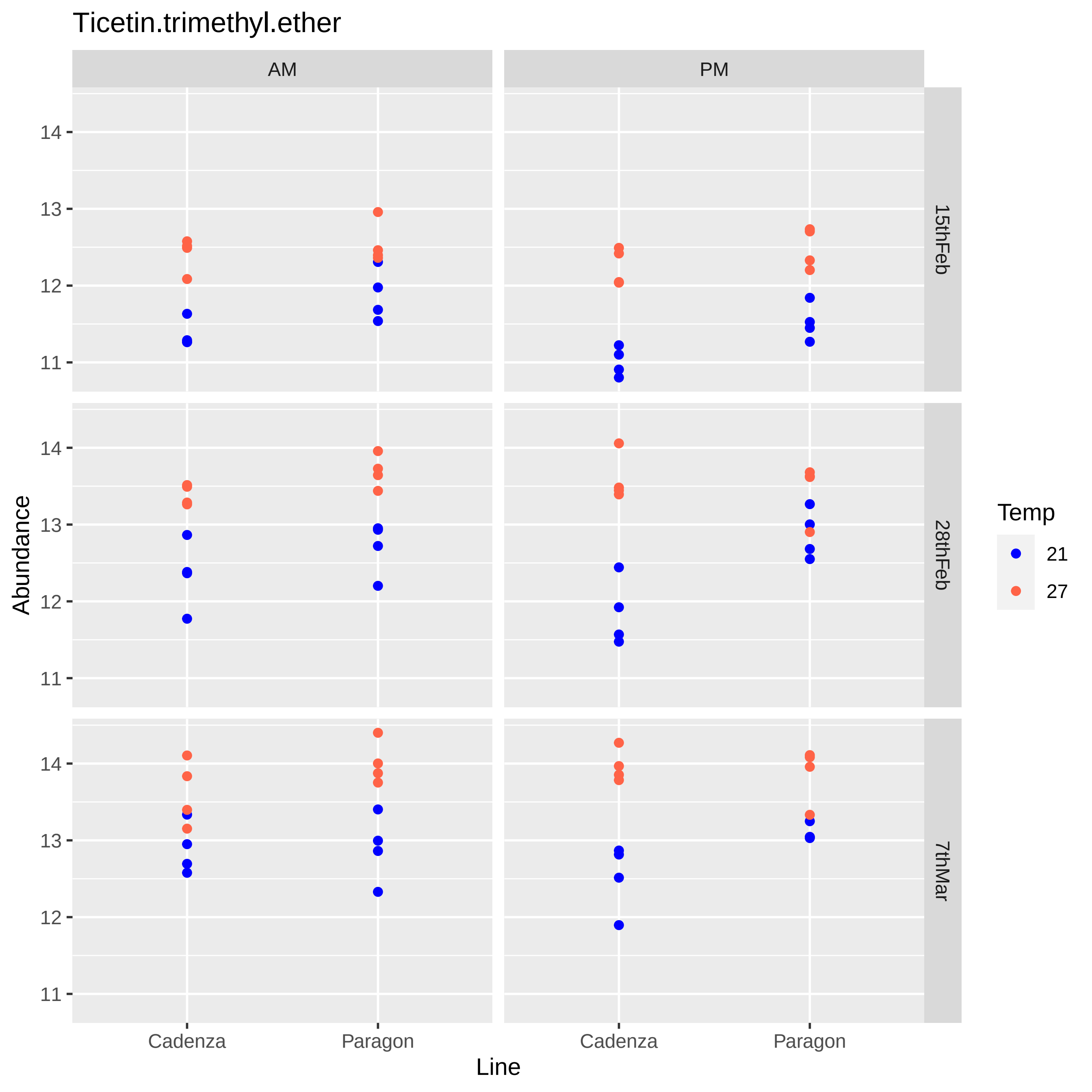
Ticetin.trimethyl.ether
AM
PM
15thFeb
28thFeb
7thMar
Abundance
11
12
13
14
Line
Cadenza
Paragon
Temp
21
27

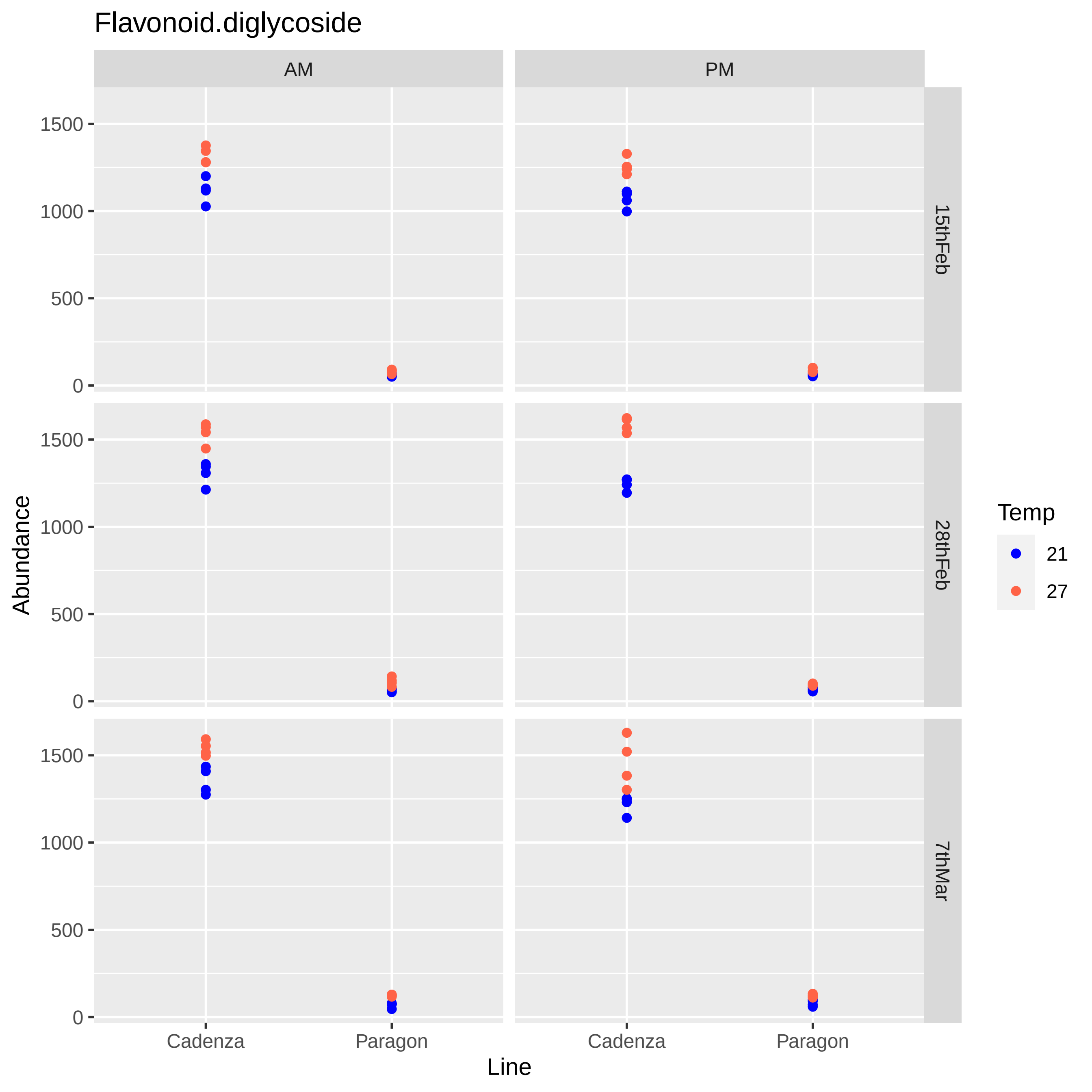

Flavonoid.diglycoside
AM
PM
15thFeb
28thFeb
7thMar
Abundance
0
500
1000
1500
Cadenza
Paragon
Line
Temp
21
27

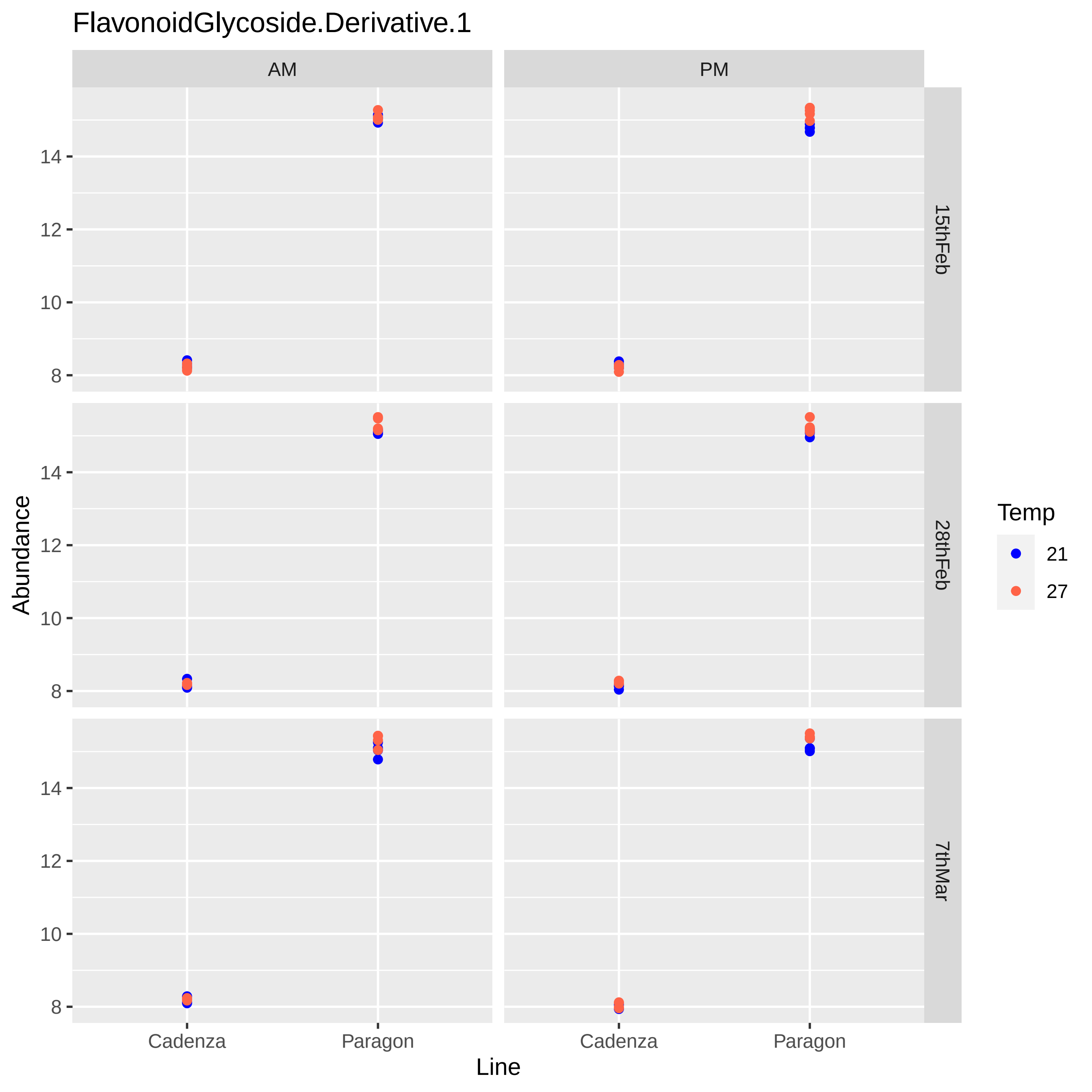
FlavonoidGlycoside.Derivative.1
AM
PM
15thFeb
28thFeb
7thMar
Abundance
8
10
12
14
Cadenza
Paragon
Line
Temp
21
27

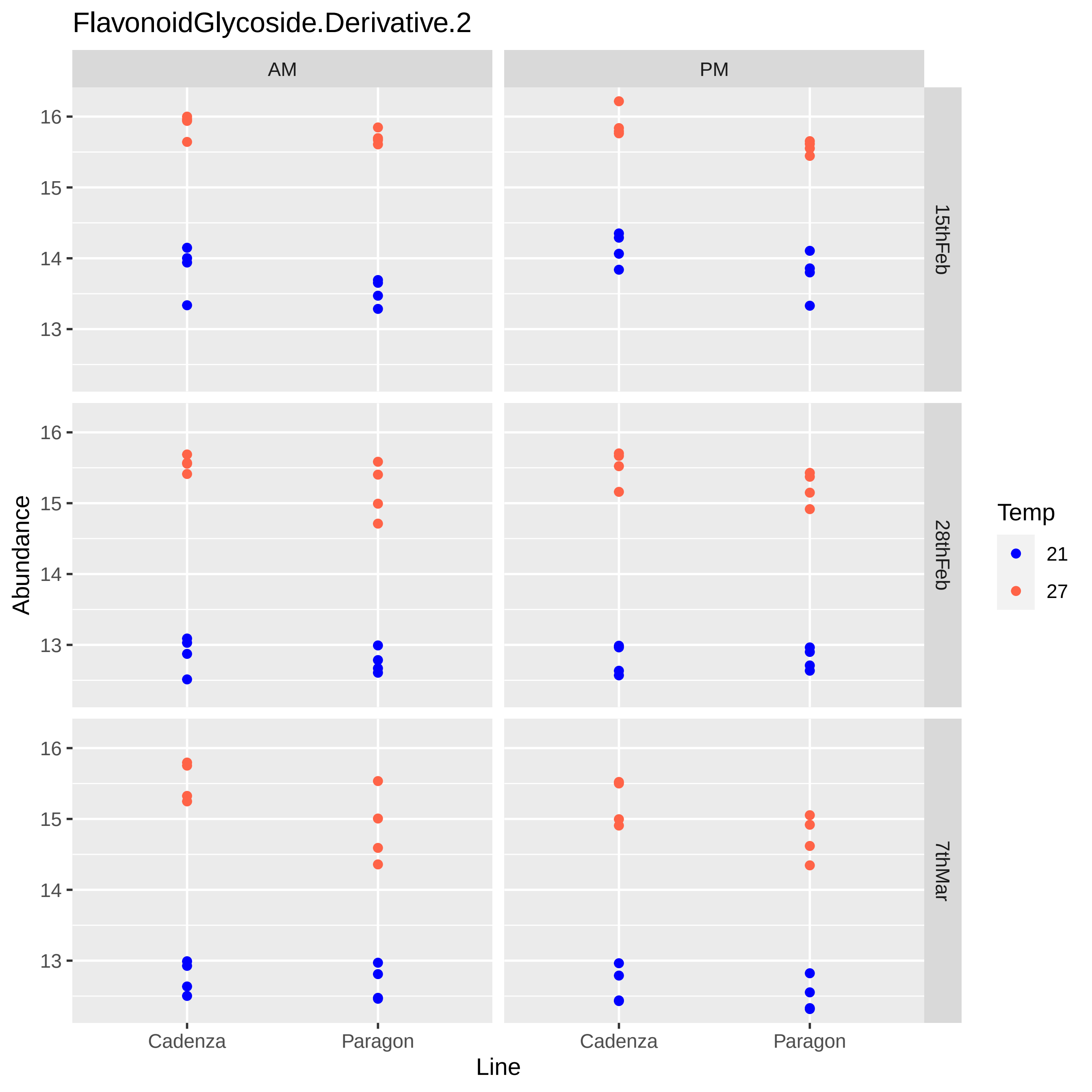

FlavonoidGlycoside.Derivative.2
AM
PM
15thFeb
28thFeb
7thMar
16
15
14
13
Abundance
Cadenza
Paragon
Line
Temp
21
27

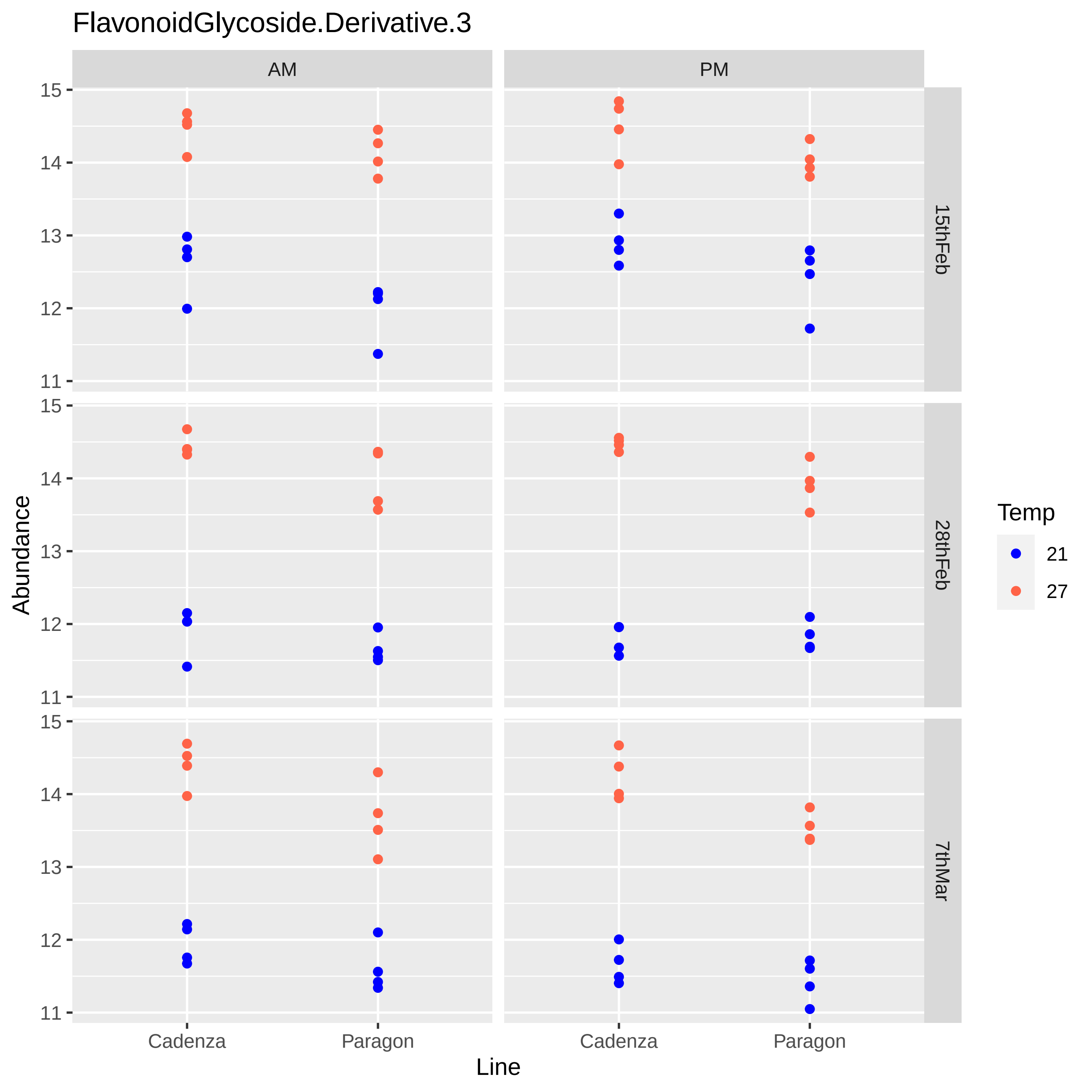
FlavonoidGlycoside.Derivative.3
AM
PM
15thFeb
28thFeb
7thMar
Abundance
Line
Cadenza
Paragon
11
12
13
14
15
Temp
21
27

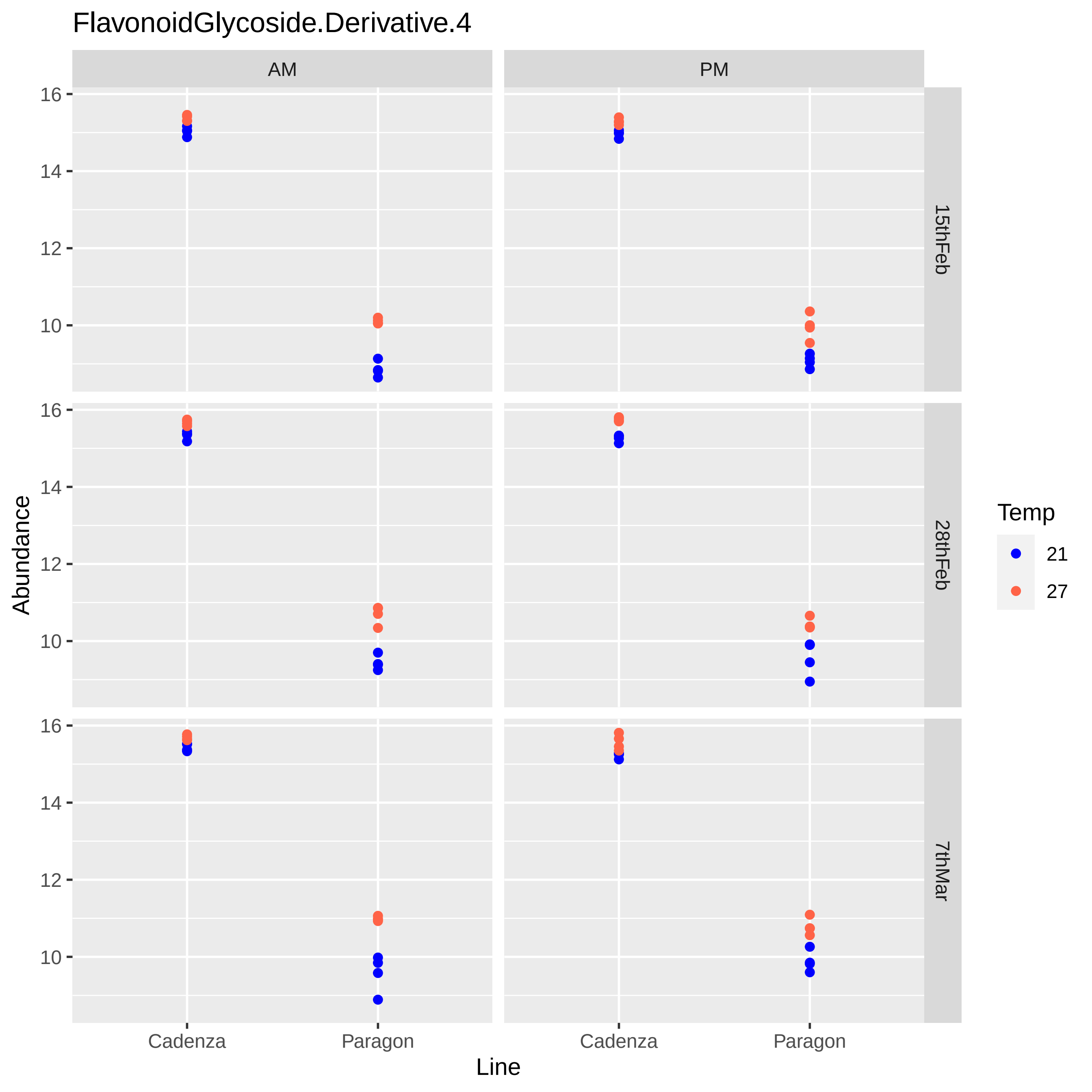

FlavonoidGlycoside.Derivative.4
AM
PM
15thFeb
28thFeb
7thMar
Abundance
16
14
12
10
Cadenza
Paragon
Line
Temp
21
27

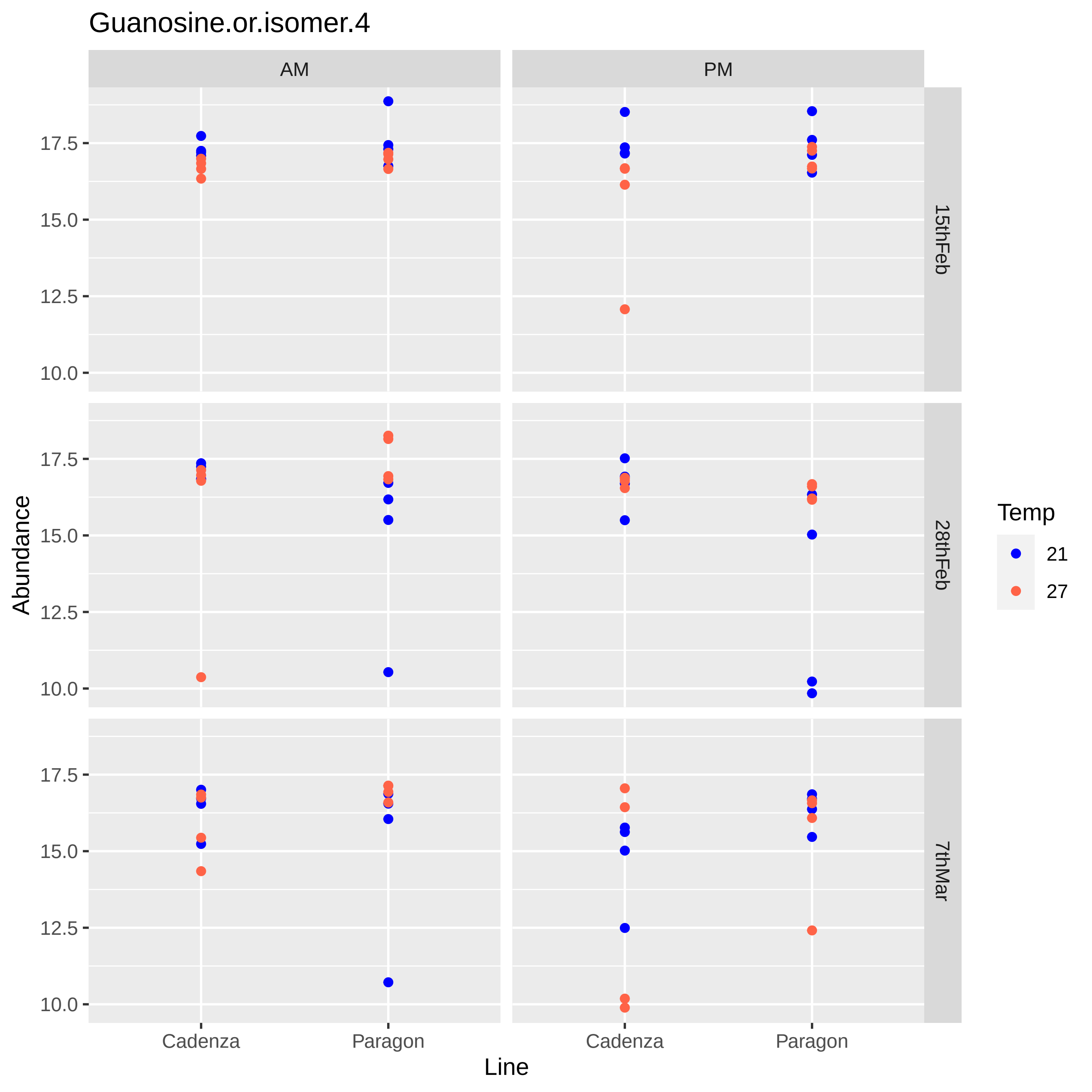

Guanosine.or.isomer.4
AM
PM
15thFeb
28thFeb
7thMar
Abundance
17.5
15.0
12.5
10.0
Cadenza
Paragon
Line
Temp
21
27

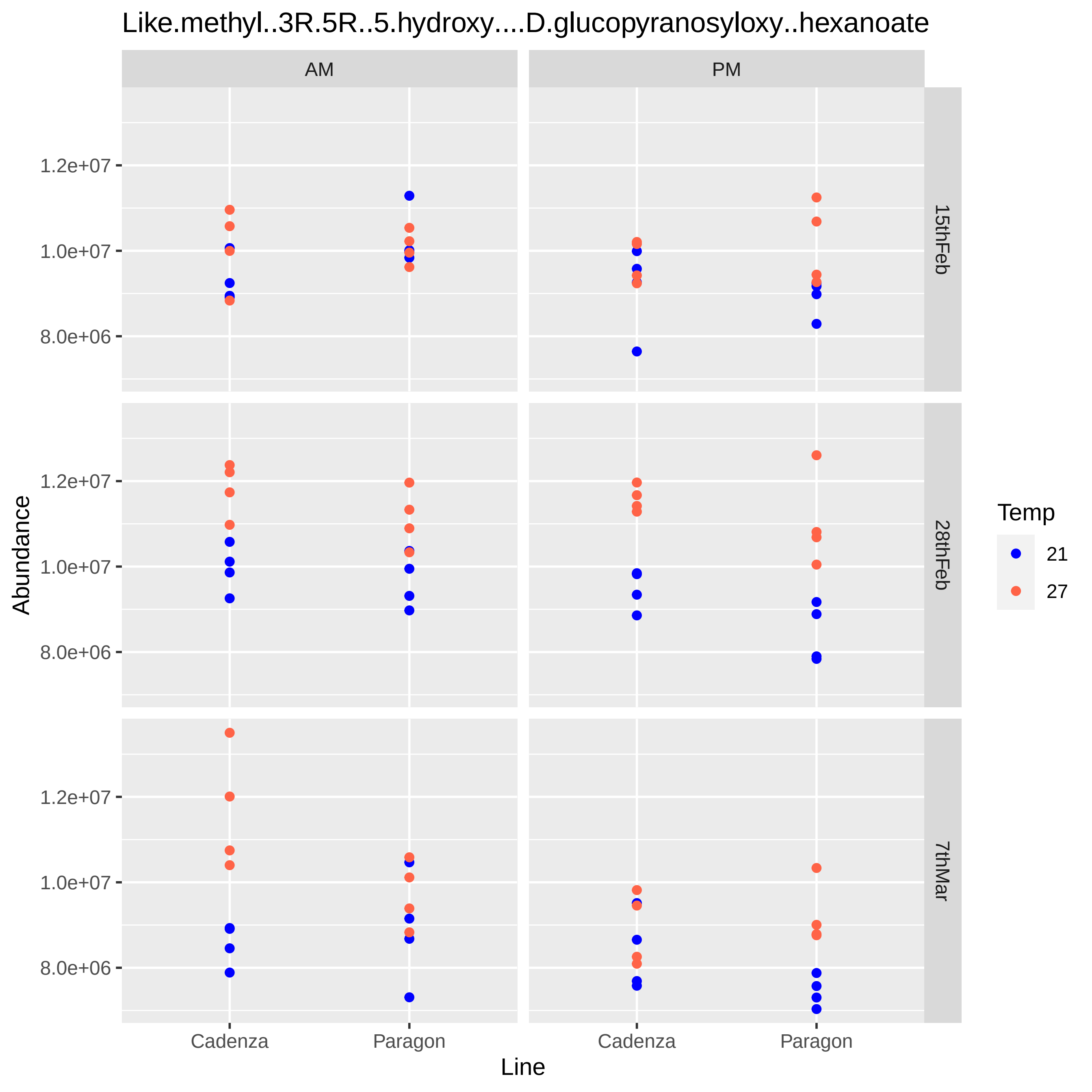
Like.methyl..3R.5R..5.hydroxy....D.glucopyranosyloxy..hexanoate
AM
PM
15thFeb
28thFeb
7thMar
1.2e+07
1.0e+07
8.0e+06
Abundance
Cadenza
Paragon
Line
Temp
21
27

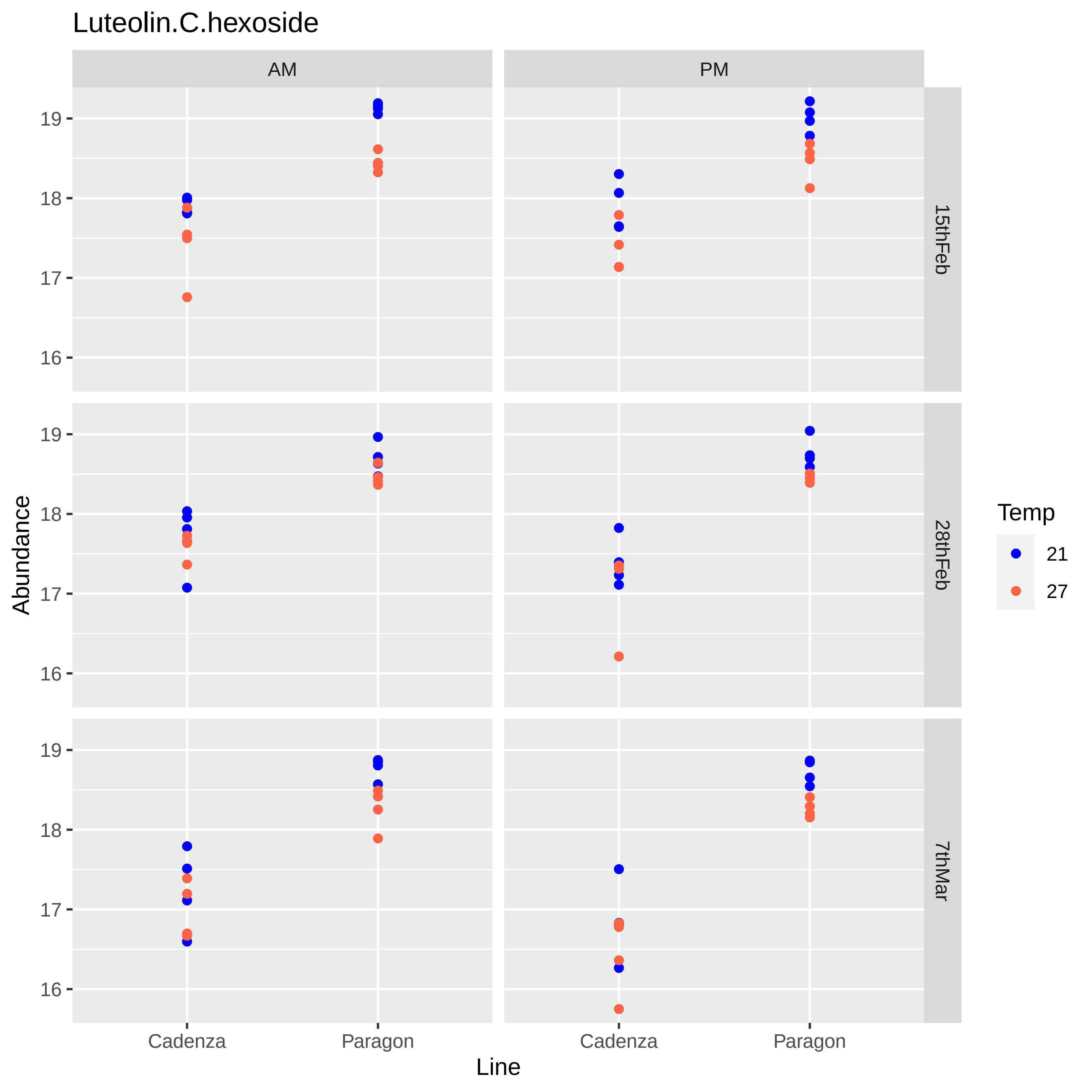

Luteolin.C.hexoside
AM
PM
15thFeb
28thFeb
7thMar
19
18
17
16
Abundance
Cadenza
Paragon
Line
Temp
21
27

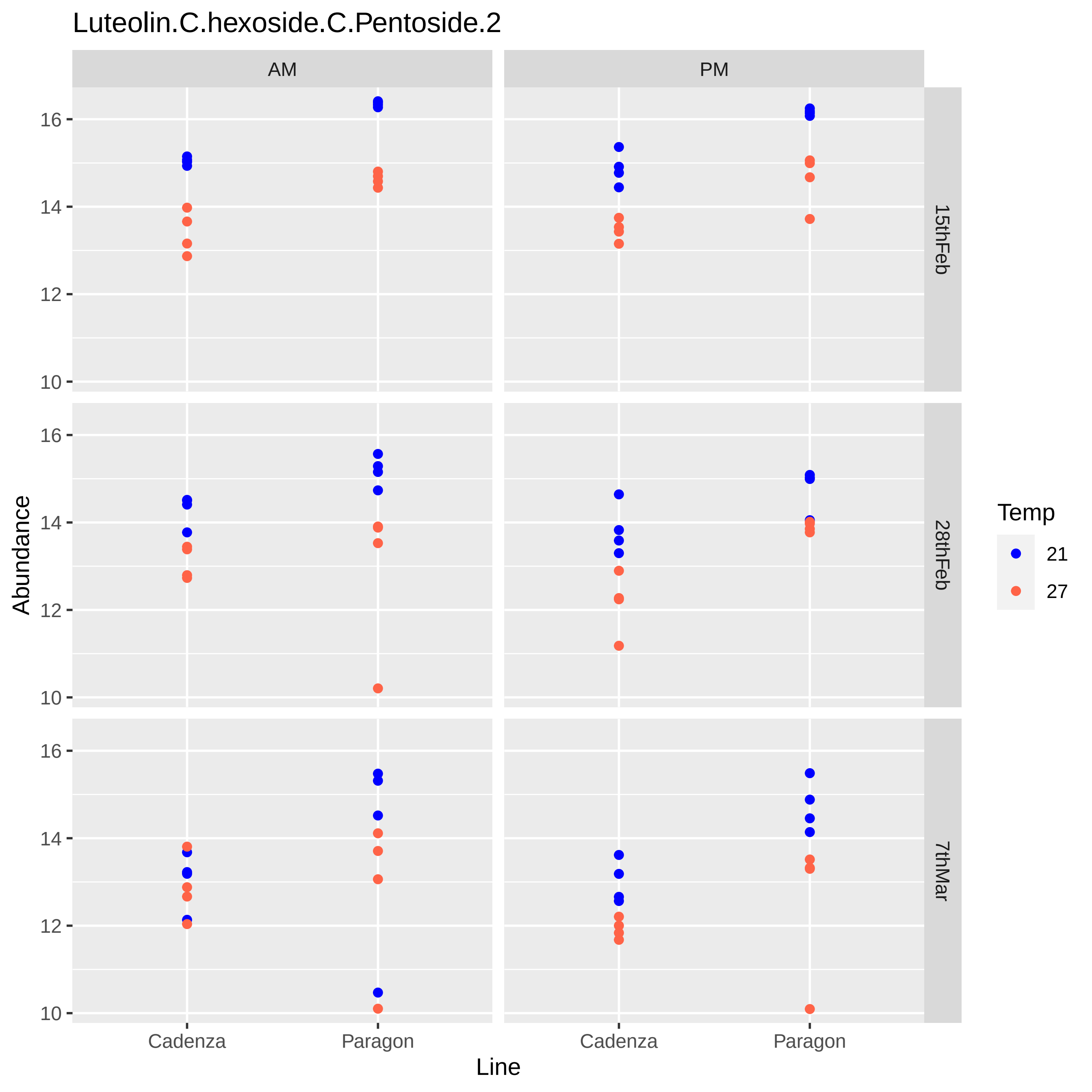

Luteolin.C.hexoside.C.Pentoside.2
AM
PM
15thFeb
28thFeb
7thMar
Abundance
16
14
12
10
Cadenza
Paragon
Line
Temp
21
27

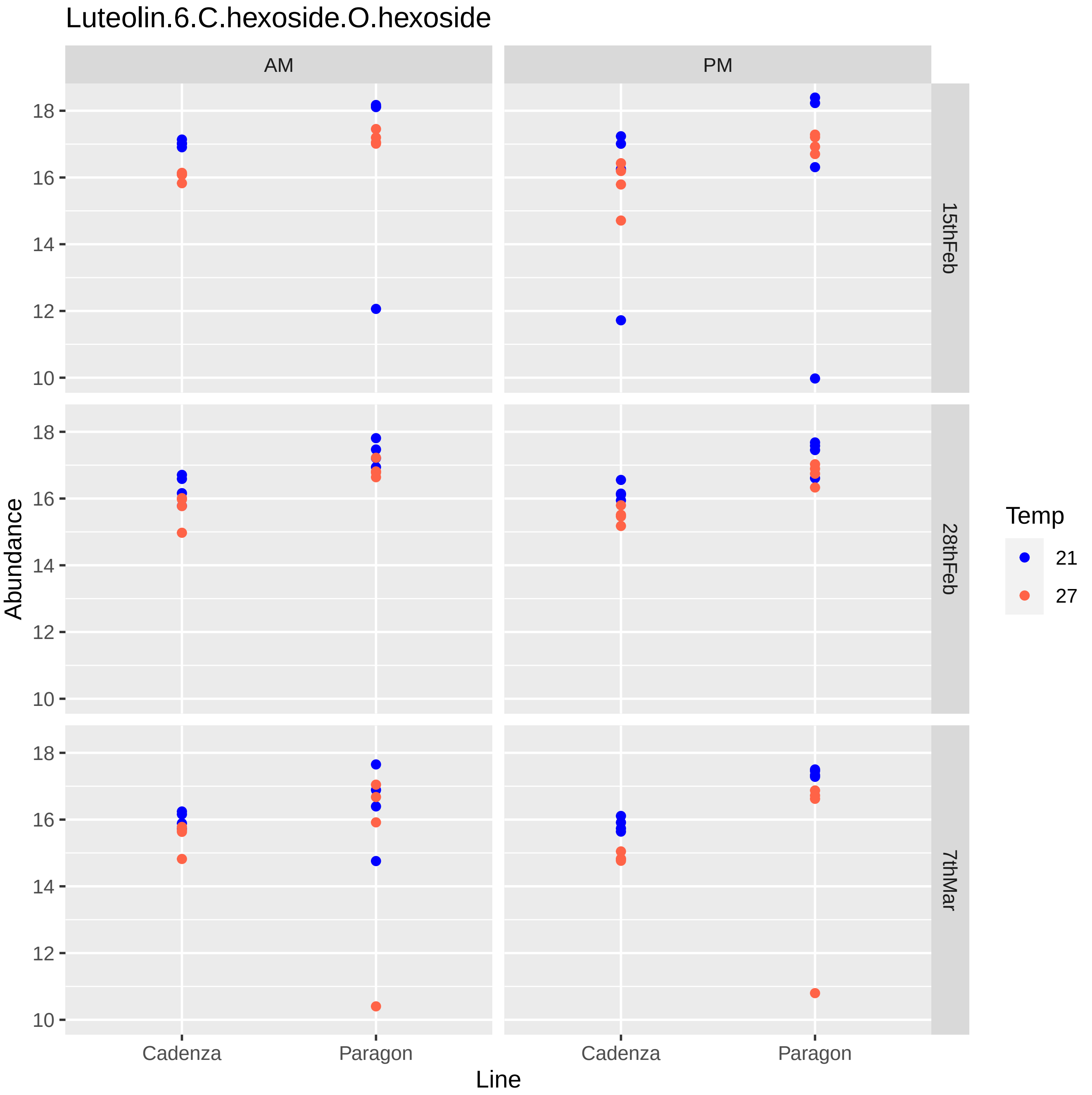

Luteolin.6.C.hexoside.O.hexoside
AM
PM
15thFeb
28thFeb
7thMar
Abundance
18
16
14
12
10
Cadenza
Paragon
Line
Temp
21
27

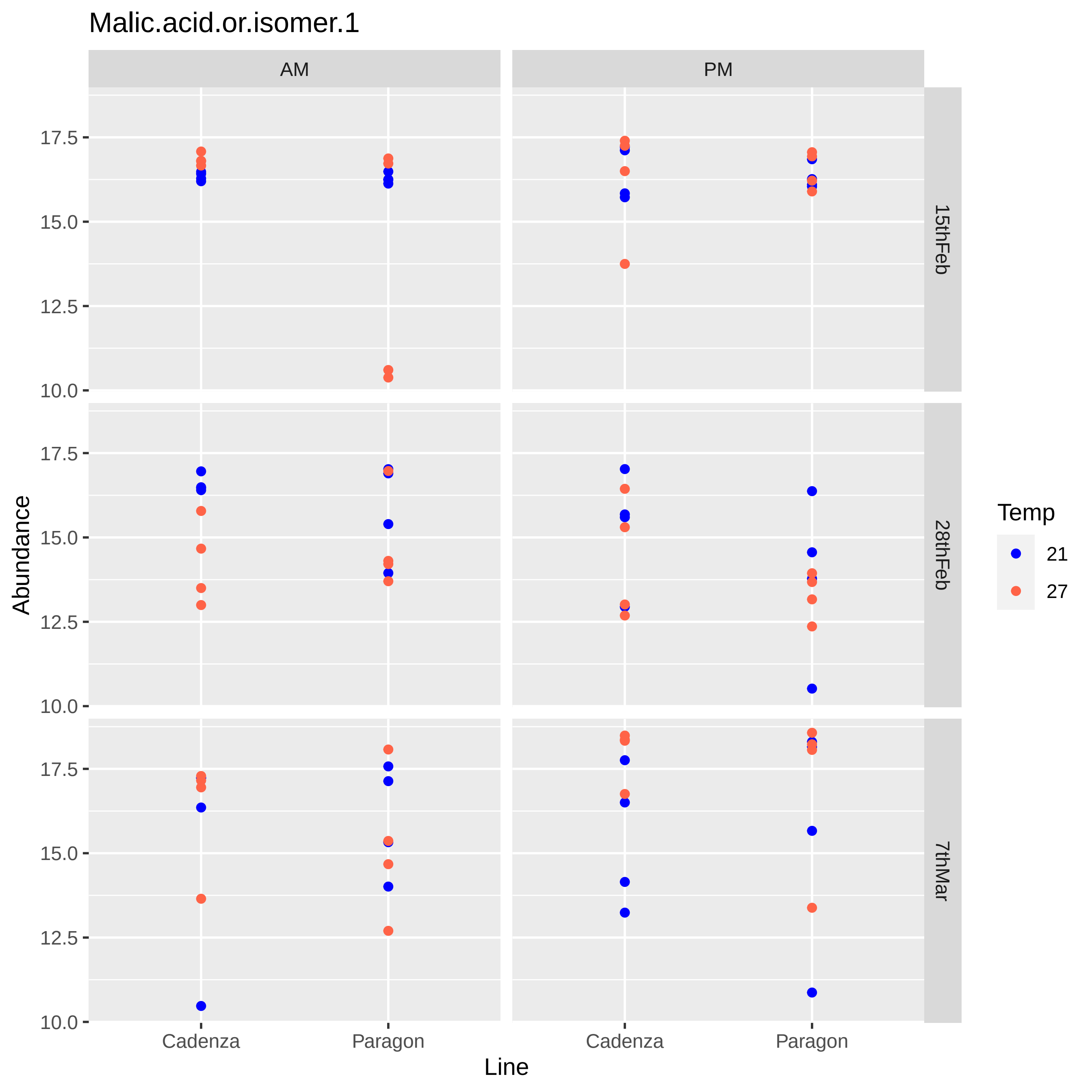

Malic.acid.or.isomer.1
AM
PM
15thFeb
28thFeb
7thMar
Abundance
17.5
15.0
12.5
10.0
Cadenza
Paragon
Line
Temp
21
27

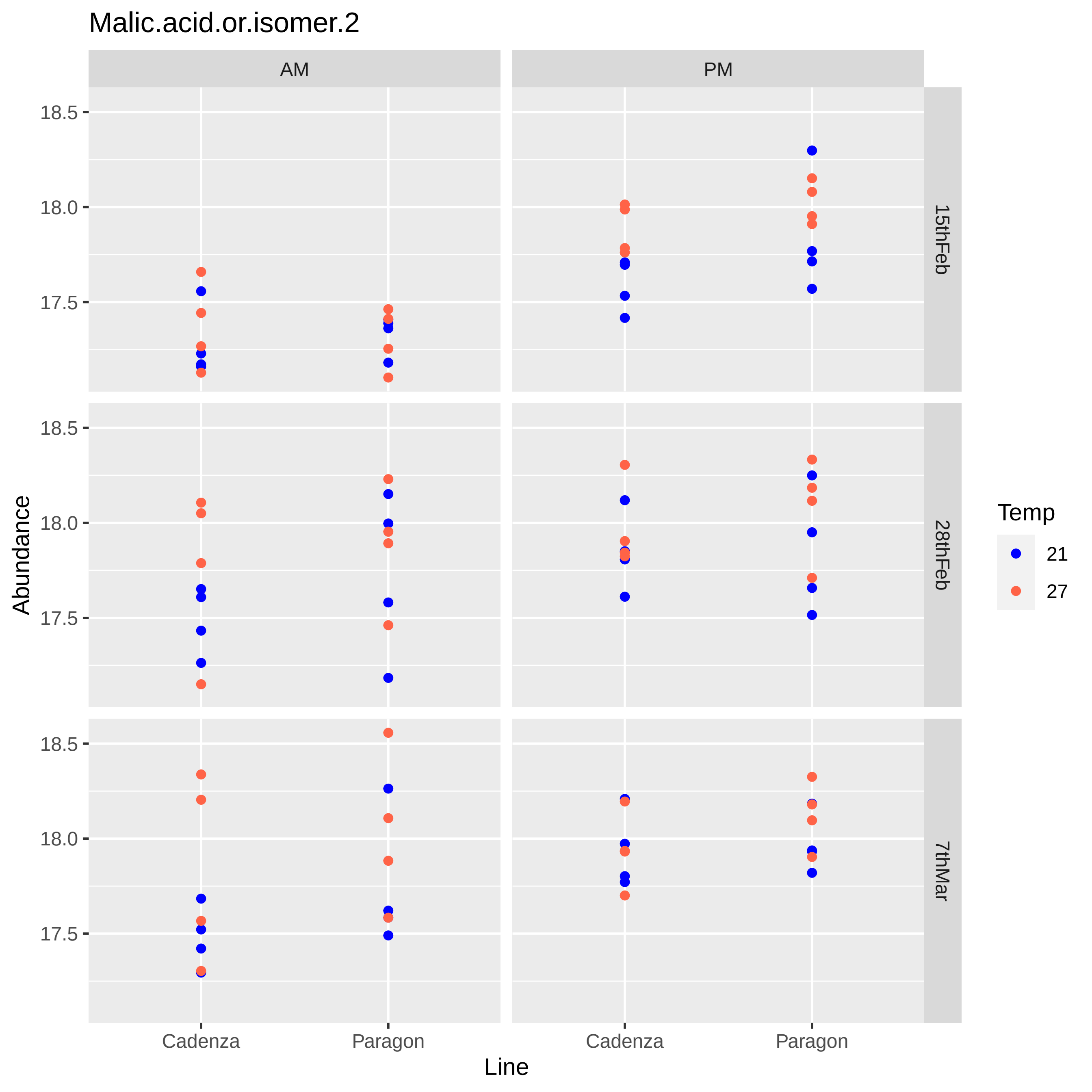

Malic.acid.or.isomer.2
AM
PM
15thFeb
28thFeb
7thMar
Abundance
18.5
18.0
17.5
Cadenza
Paragon
Line
Temp
21
27

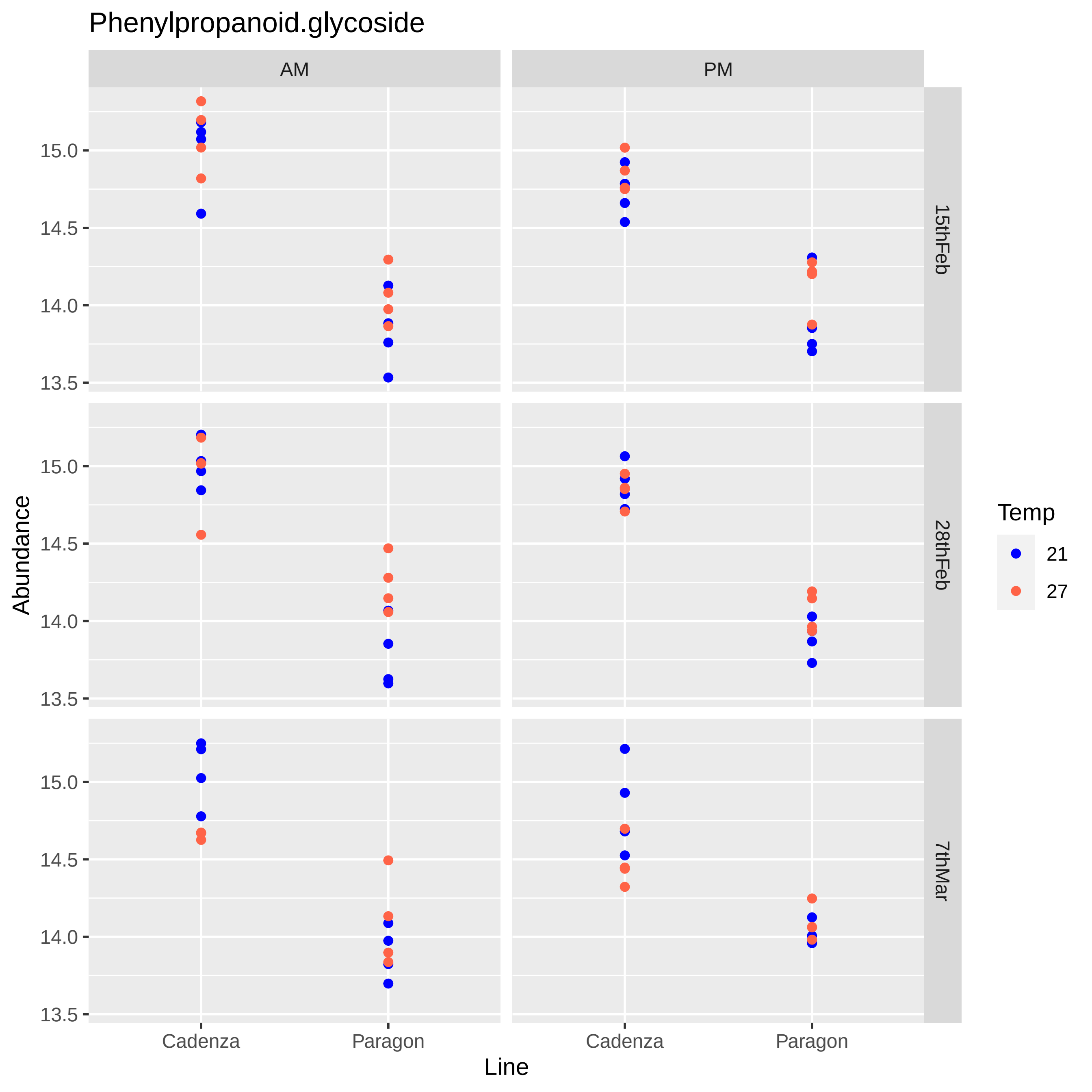

Phenylpropanoid.glycoside
AM
PM
15thFeb
28thFeb
7thMar
15.0
14.5
14.0
13.5
Abundance
Cadenza
Paragon
Line
Temp
21
27

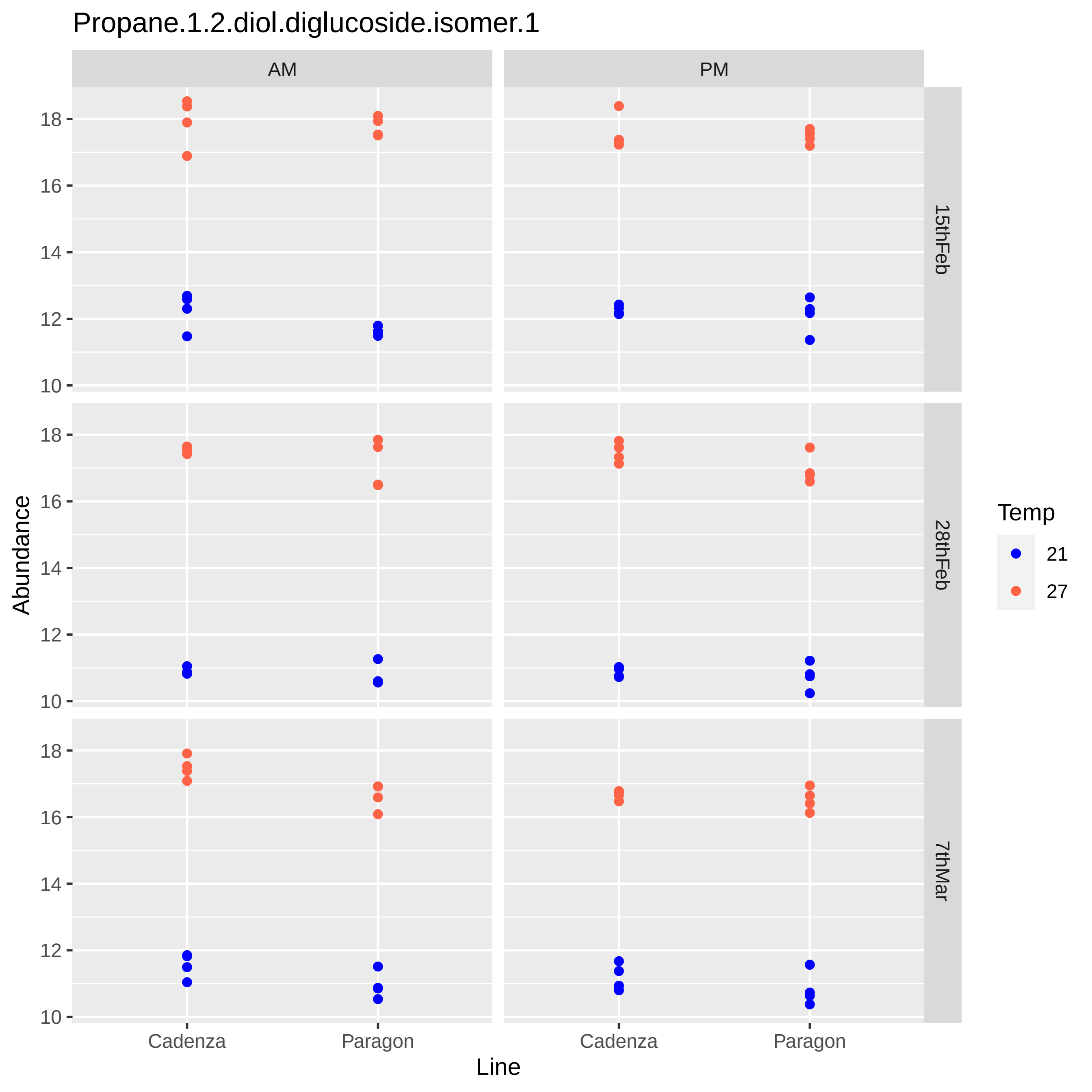

Propane.1.2.diol.diglucoside.isomer.1
AM
PM
15thFeb
28thFeb
7thMar
Abundance
18
16
14
12
10
Cadenza
Paragon
Line
Temp
21
27

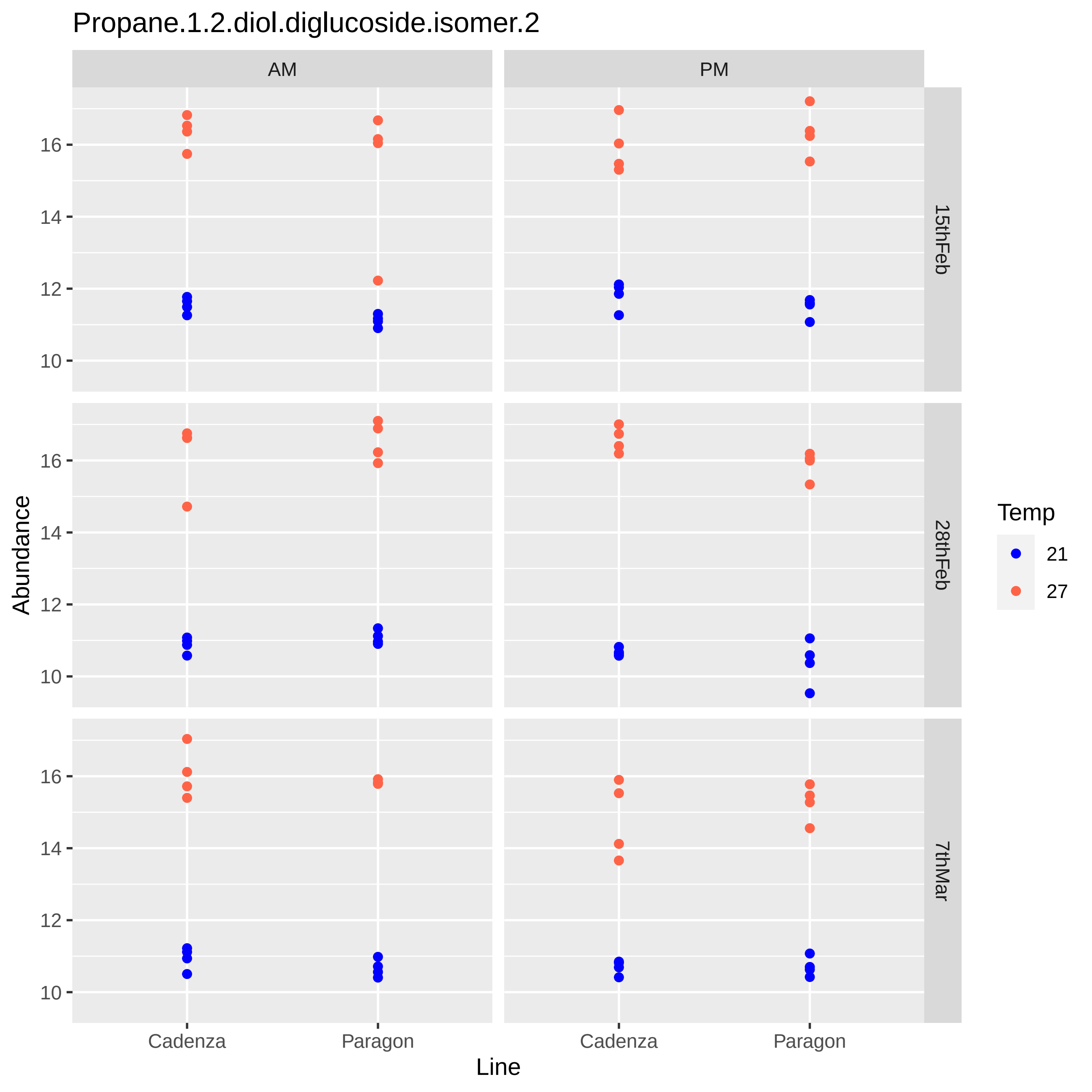

Propane.1.2.diol.diglucoside.isomer.2
AM
PM
15thFeb
28thFeb
7thMar
Abundance
16
14
12
10
Cadenza
Paragon
Line
Temp
21
27

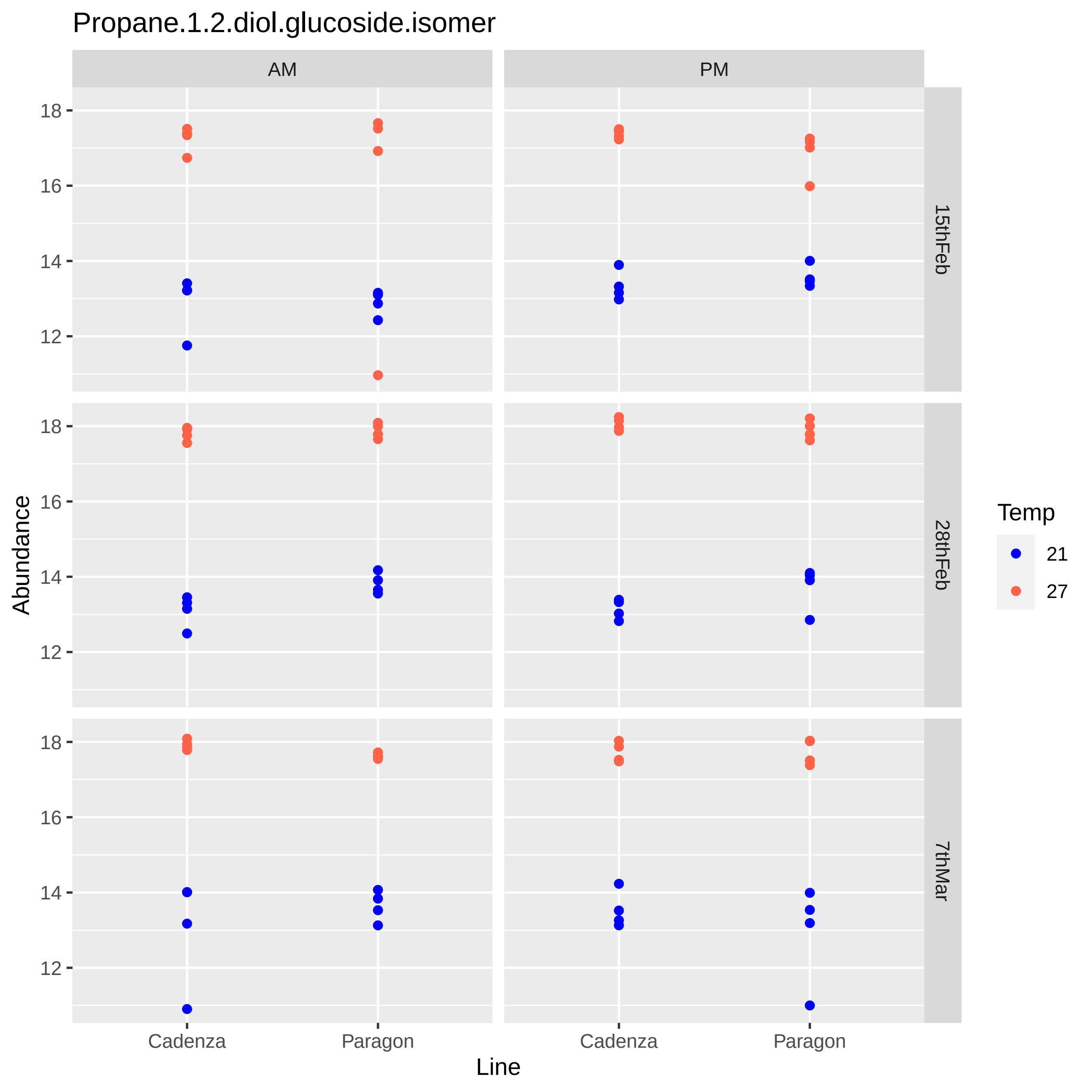

Propane.1.2.diol.glucoside.isomer
AM
PM
15thFeb
28thFeb
7thMar
Abundance
12
14
16
18
Cadenza
Paragon
Line
Temp
21
27

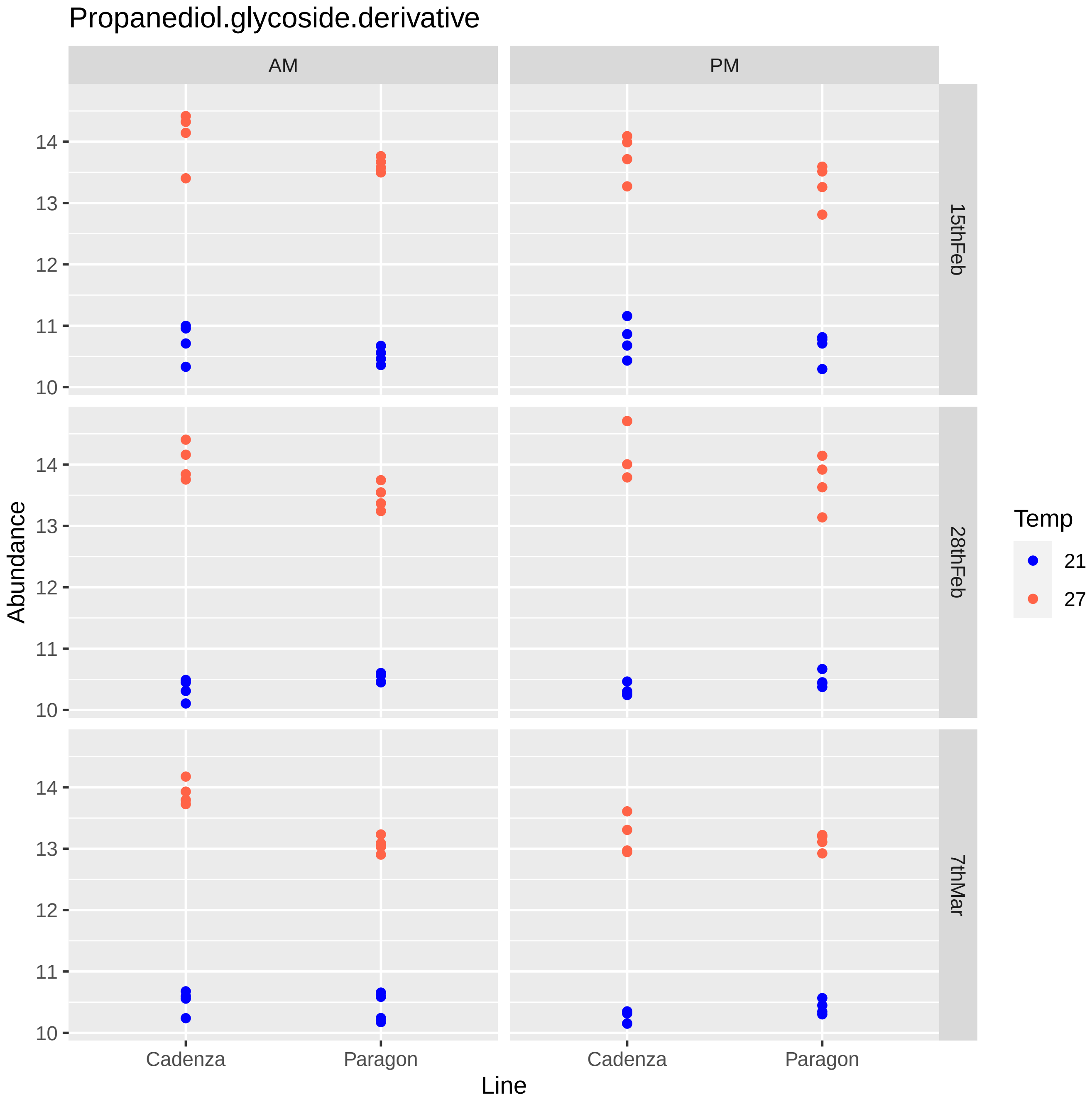
Propanediol.glycoside.derivative
AM
PM
15thFeb
28thFeb
7thMar
Abundance
10
11
12
13
14
Cadenza
Paragon
Line
Temp
21
27

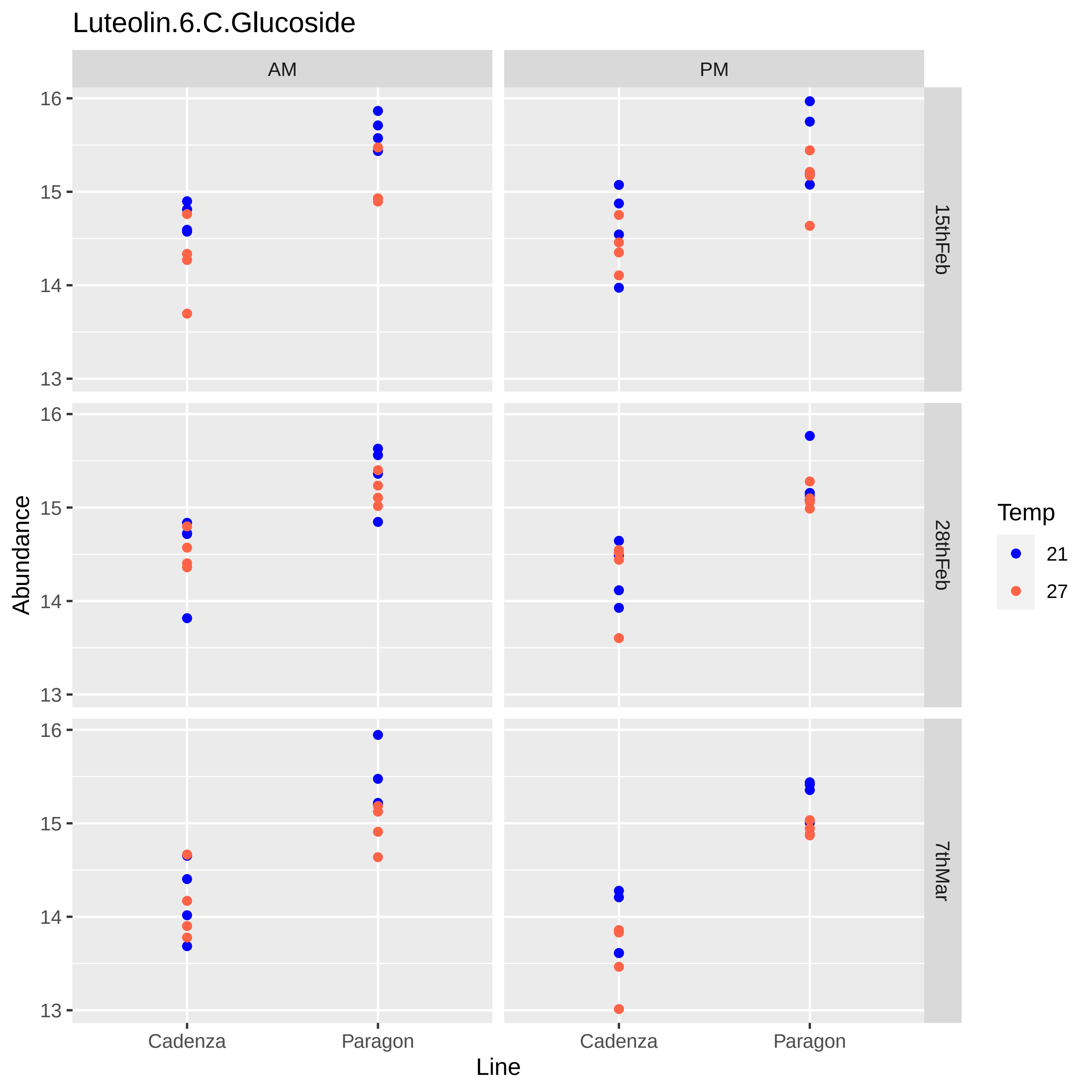
Luteolin.6.C.Glucoside
AM
PM
15thFeb
28thFeb
7thMar
Abundance
16
15
14
13
Cadenza
Paragon
Line
Temp
21
27

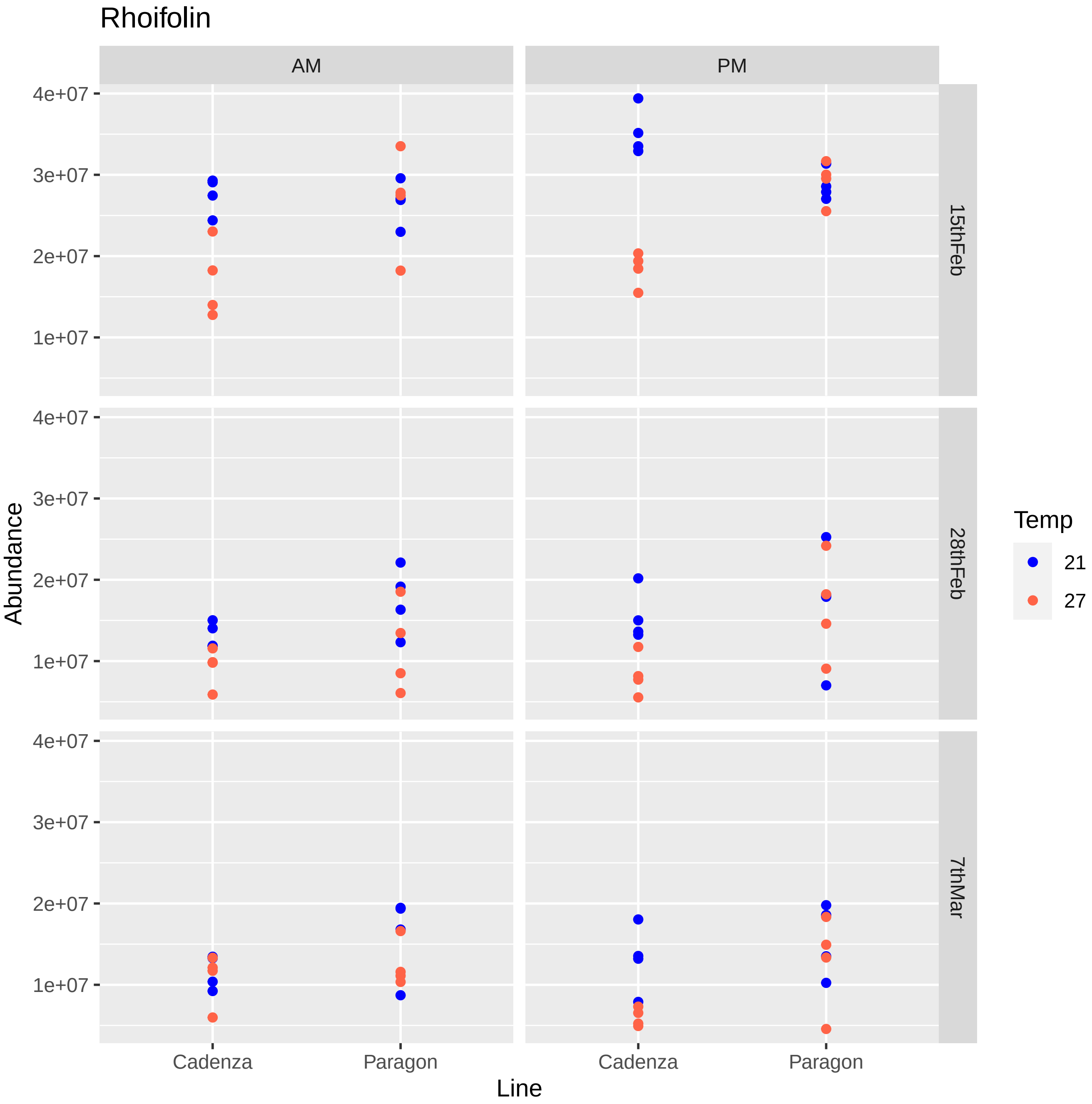
Rhoifolin
AM
PM
15thFeb
28thFeb
7thMar
4e+07
3e+07
2e+07
1e+07
Abundance
Cadenza
Paragon
Line
Temp
21
27

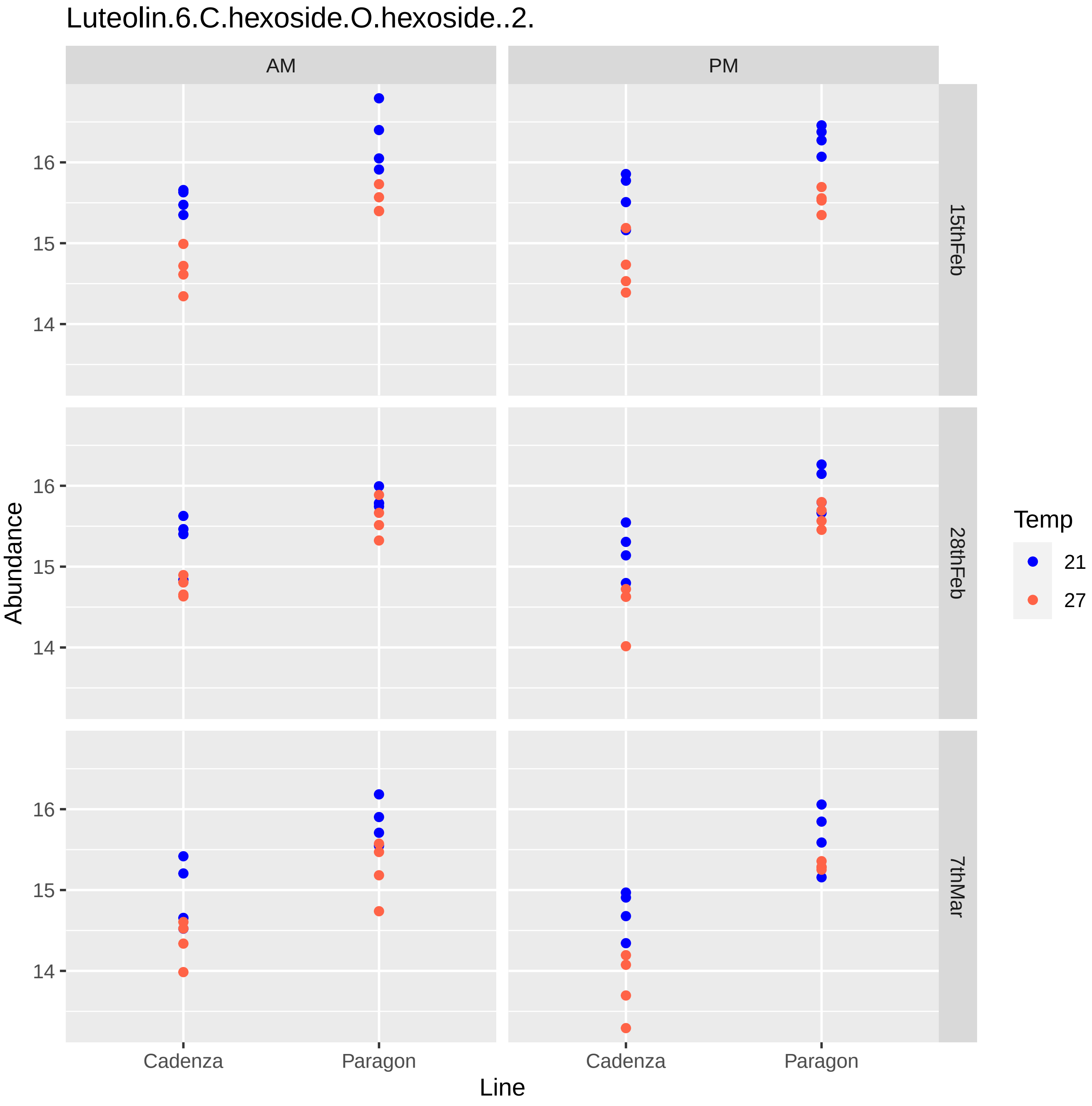
Luteolin.6.C.hexoside.O.hexoside..2.
AM
PM
15thFeb
28thFeb
7thMar
16
15
14
Abundance
Cadenza
Paragon
Line
Temp
21
27

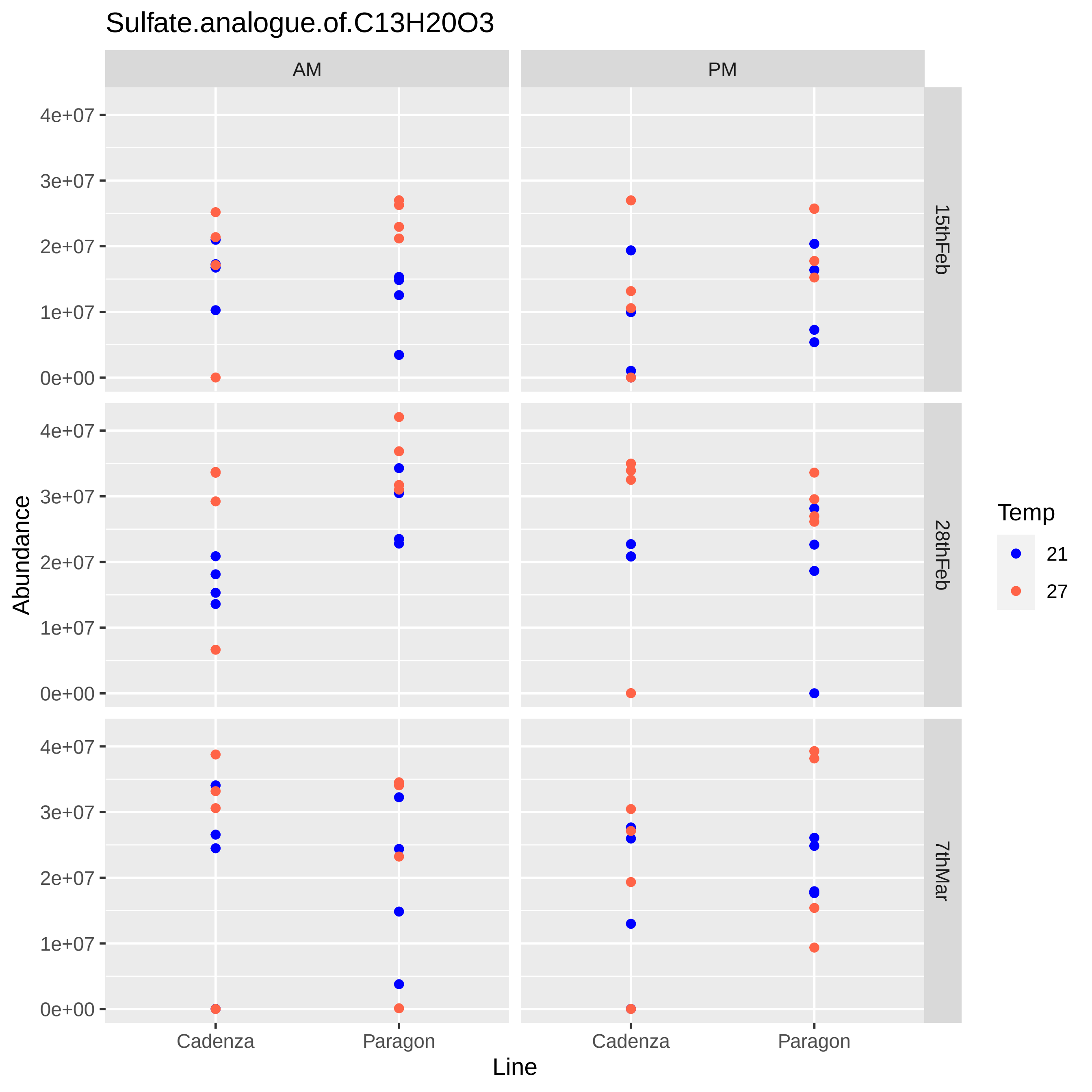

Sulfate.analogue.of.C13H20O3
AM
PM
15thFeb
28thFeb
7thMar
4e+07
3e+07
2e+07
1e+07
0e+00
Abundance
Cadenza
Paragon
Line
Temp
21
27

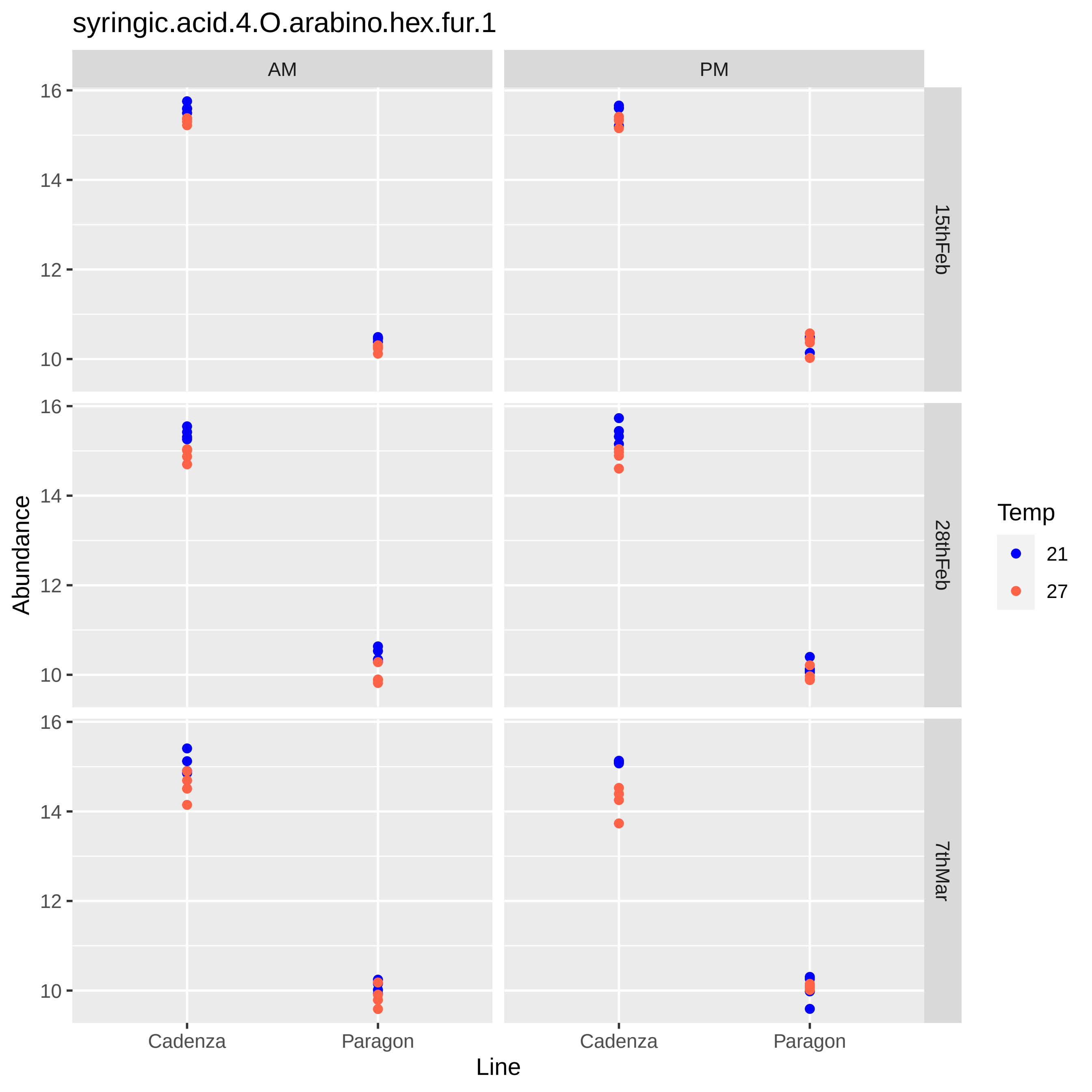

syringic.acid.4.O.arabino.hex.fur.1
AM
PM
15thFeb
28thFeb
7thMar
Abundance
16
14
12
10
Cadenza
Paragon
Line
Temp
21
27

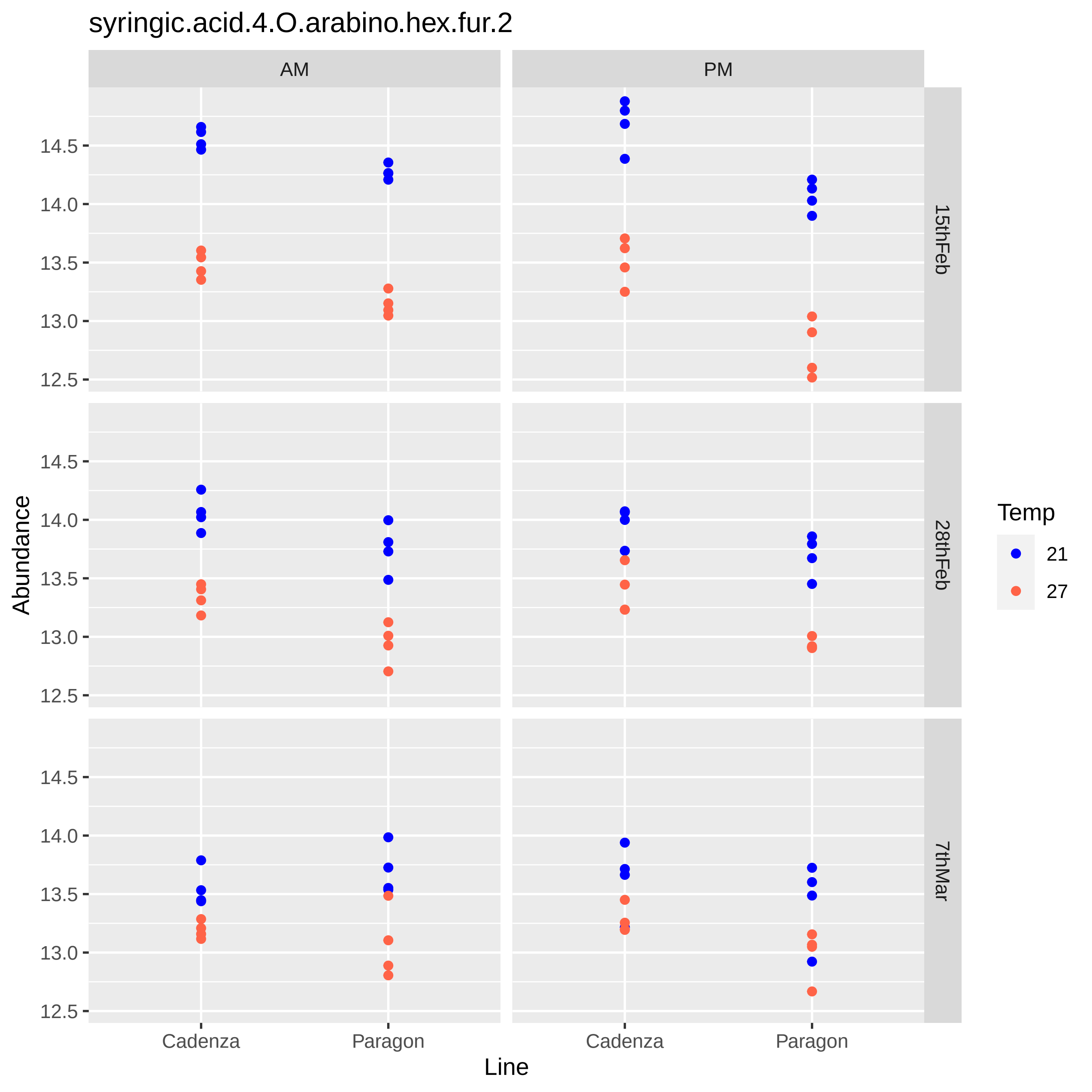

syringic.acid.4.O.arabino.hex.fur.2
AM
PM
15thFeb
28thFeb
7thMar
14.5
14.0
13.5
13.0
12.5
Abundance
Cadenza
Paragon
Line
Temp
21
27

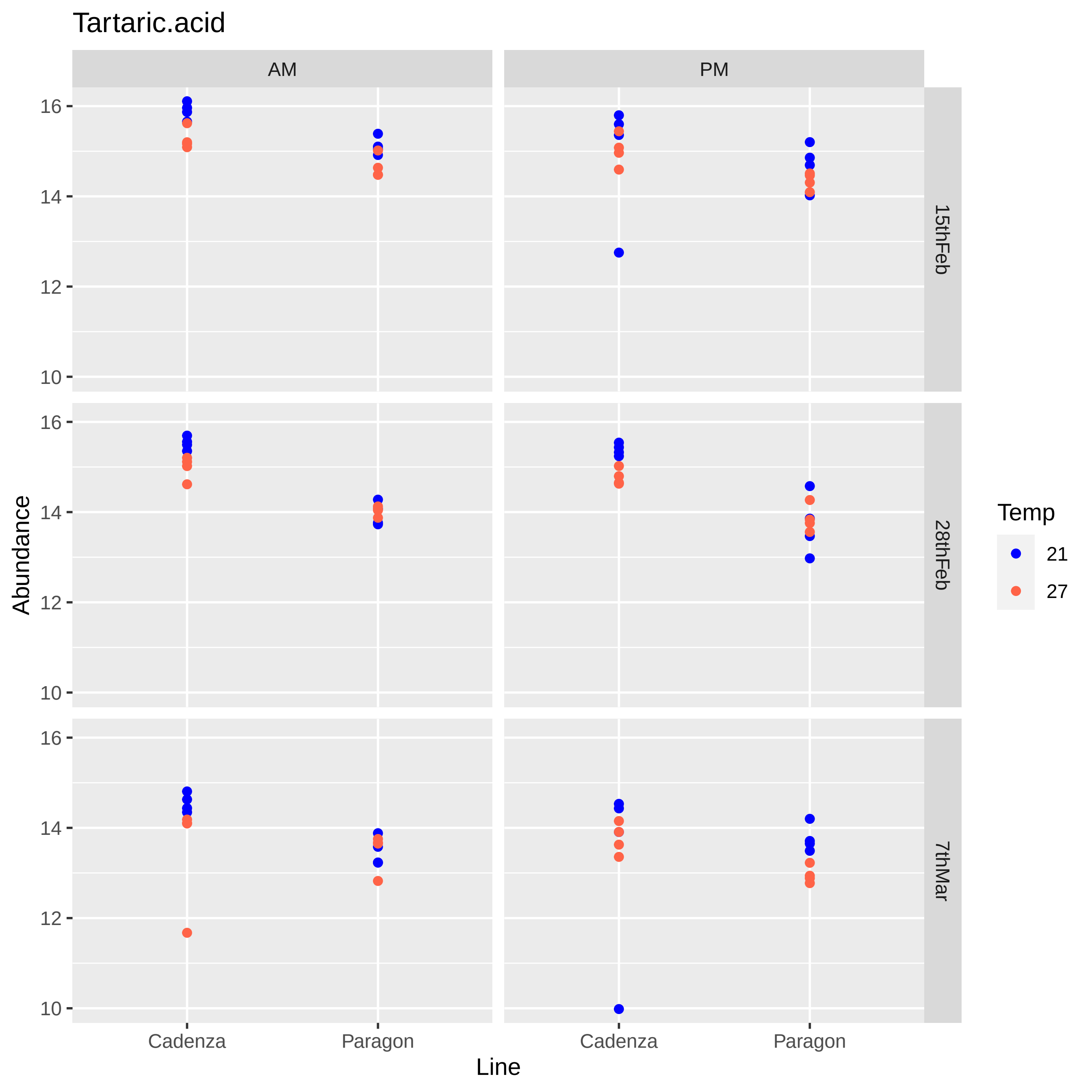

Tartaric.acid
AM
PM
15thFeb
28thFeb
7thMar
Abundance
16
14
12
10
Cadenza
Paragon
Line
Temp
21
27

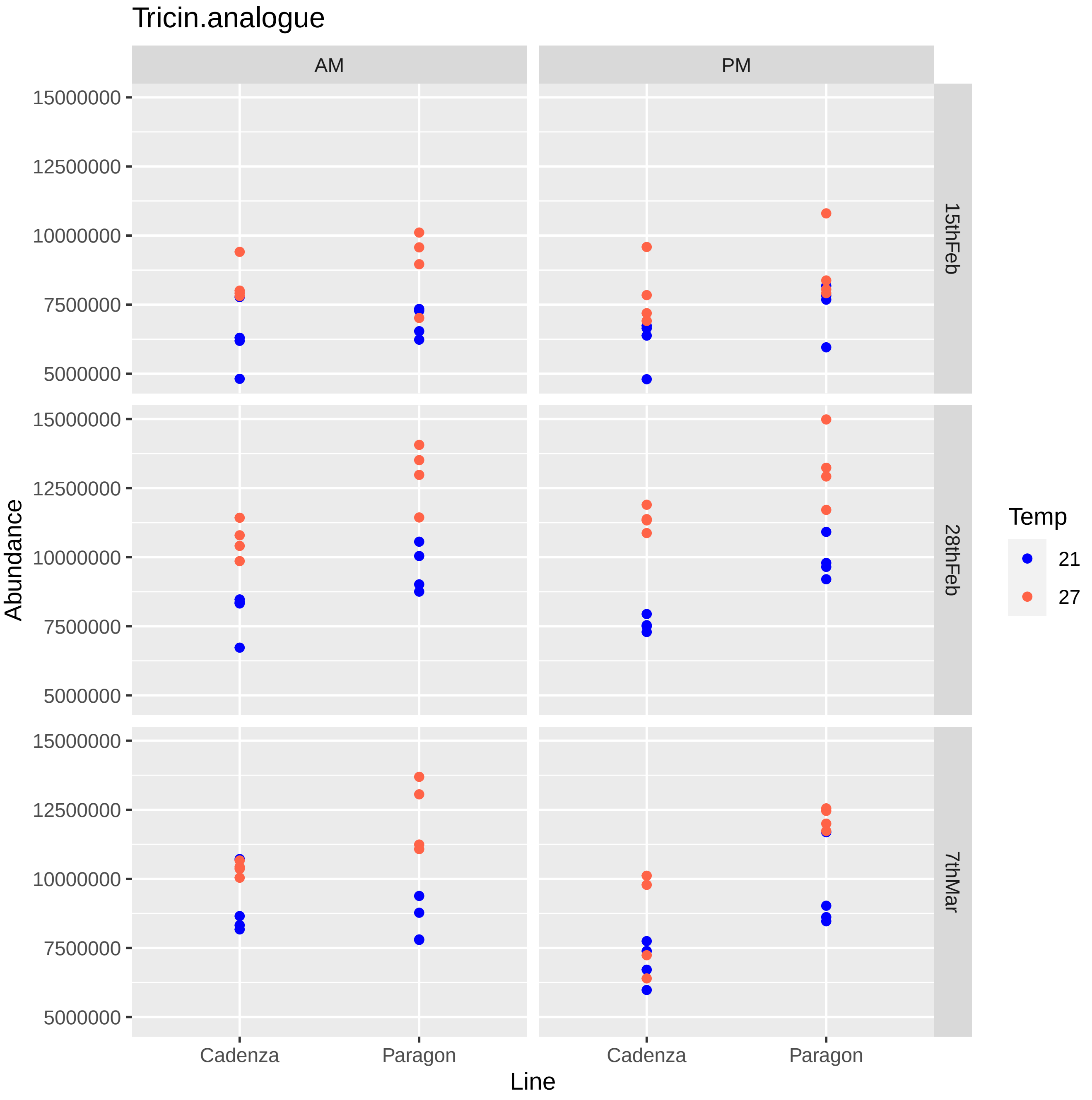
Tricin.analogue
AM
PM
15thFeb
28thFeb
7thMar
15000000
12500000
10000000
7500000
5000000
Abundance
Cadenza
Paragon
Line
Temp
21
27

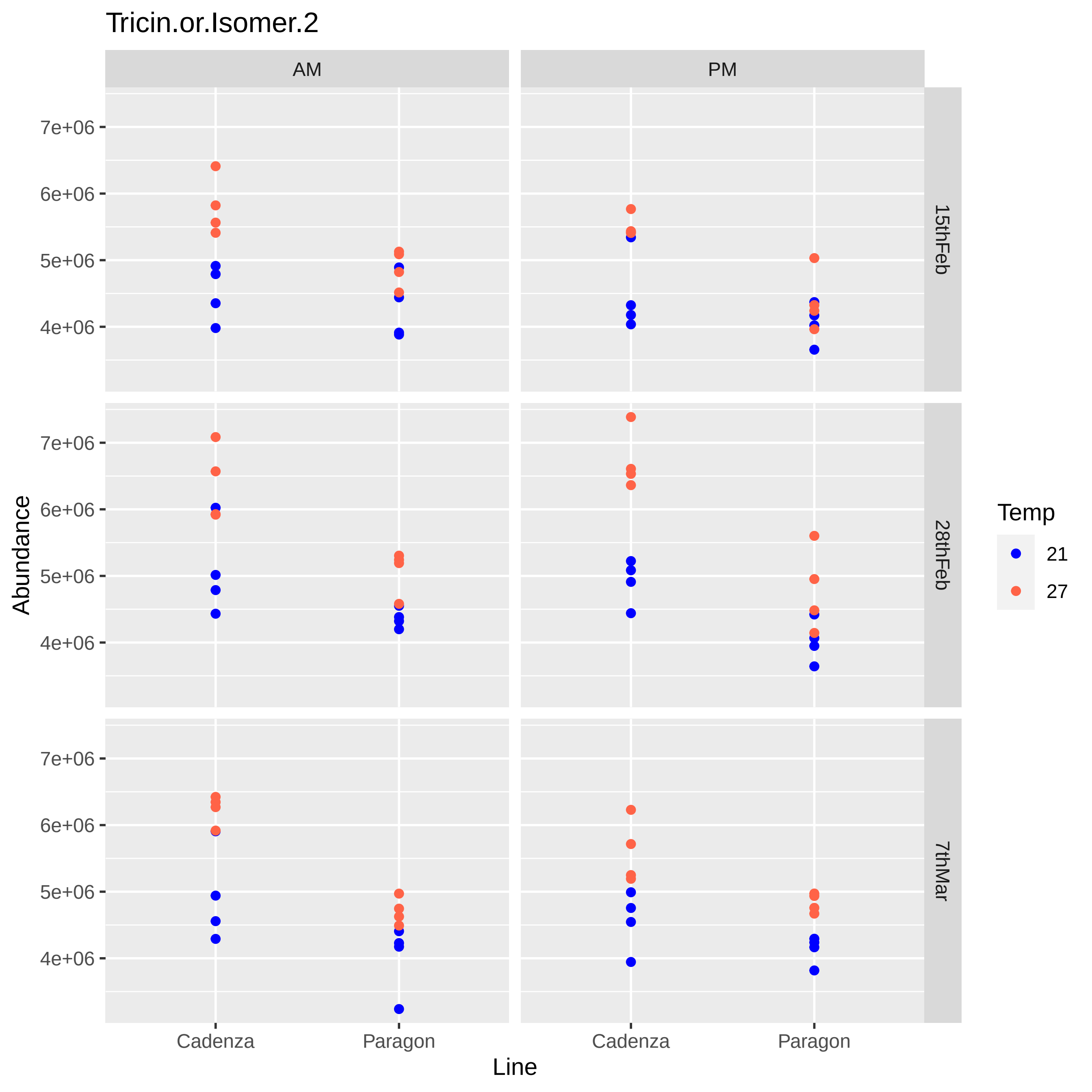

Tricin.or.Isomer.2
AM
PM
15thFeb
28thFeb
7thMar
Abundance
7e+06
6e+06
5e+06
4e+06
Cadenza
Paragon
Line
Temp
21
27

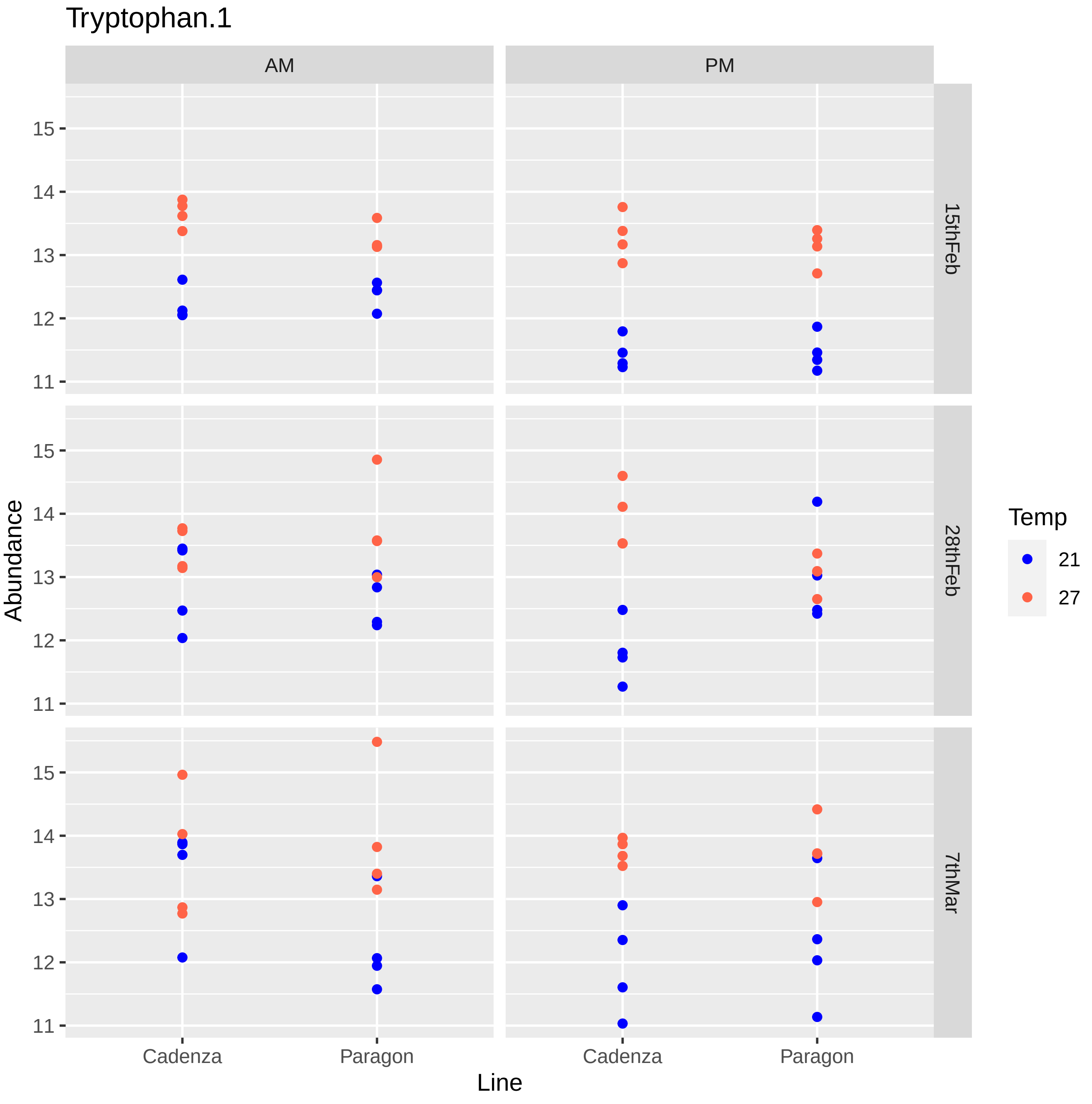

Tryptophan.1
AM
PM
15thFeb
28thFeb
7thMar
Abundance
15
14
13
12
11
Cadenza
Paragon
Line
Temp
21
27

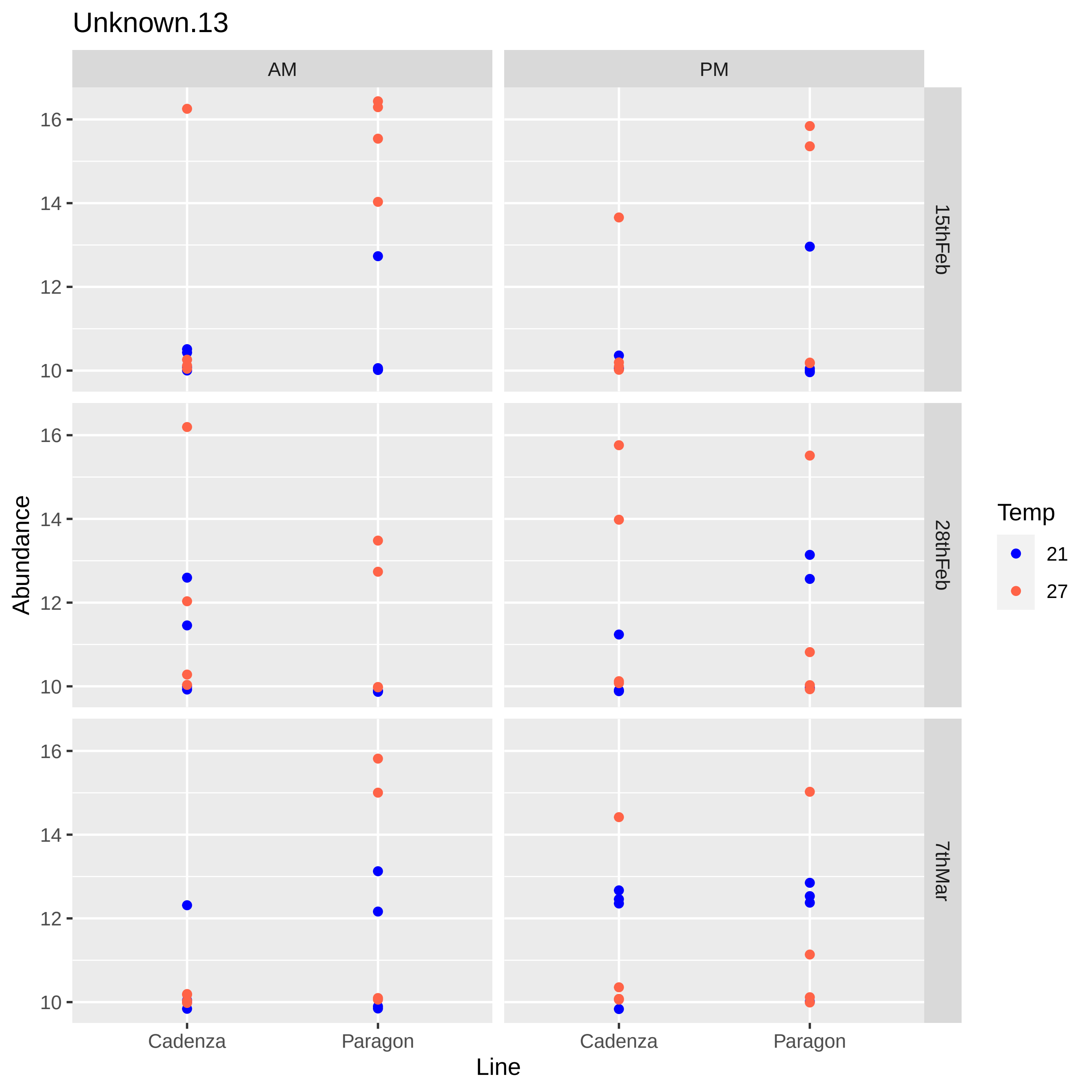

Unknown.13
AM
PM
15thFeb
28thFeb
7thMar
Abundance
16
14
12
10
Line
Cadenza
Paragon
Temp
21
27

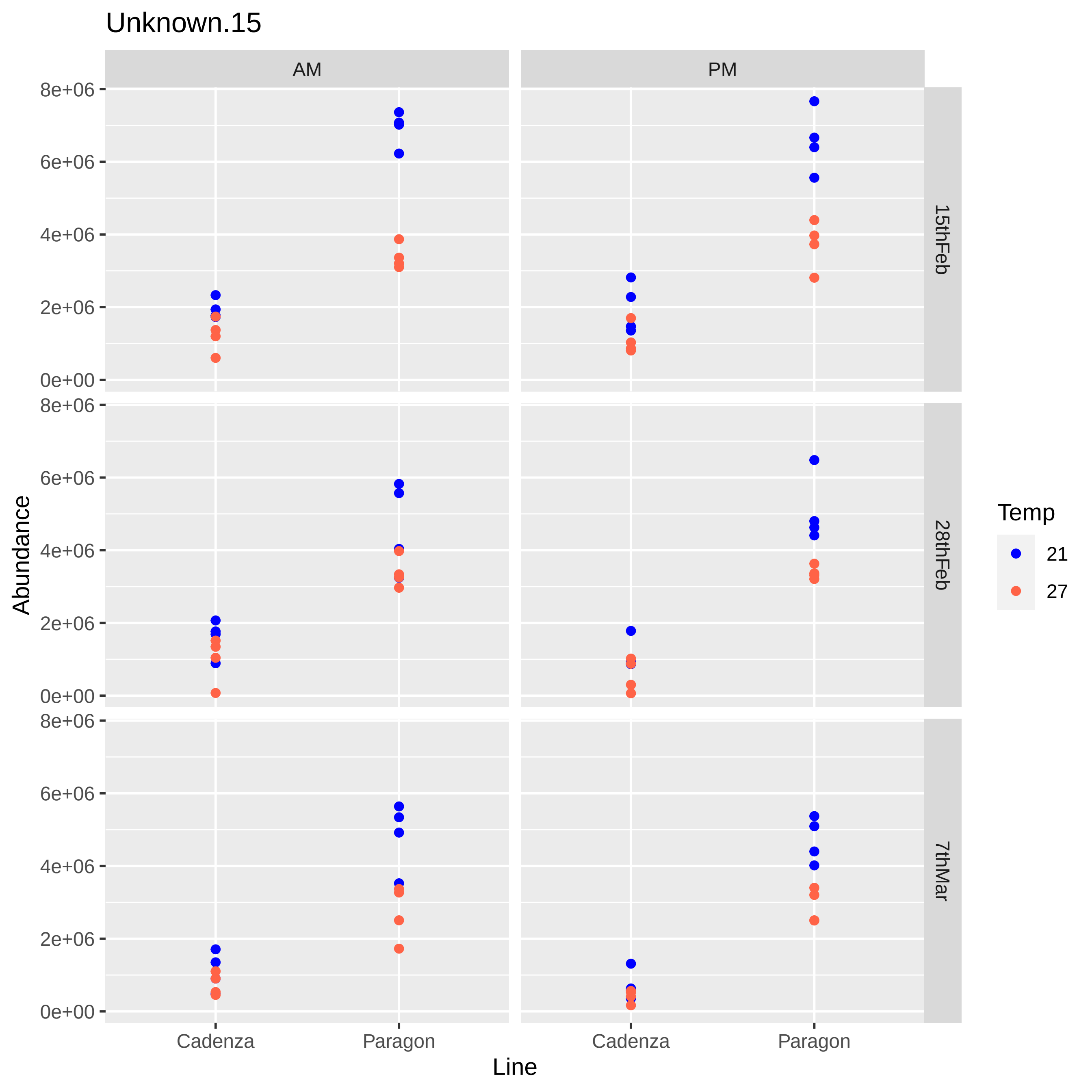

Unknown.15
AM
PM
15thFeb
28thFeb
7thMar
8e+06
6e+06
4e+06
2e+06
0e+00
Abundance
Cadenza
Paragon
Line
Temp
21
27

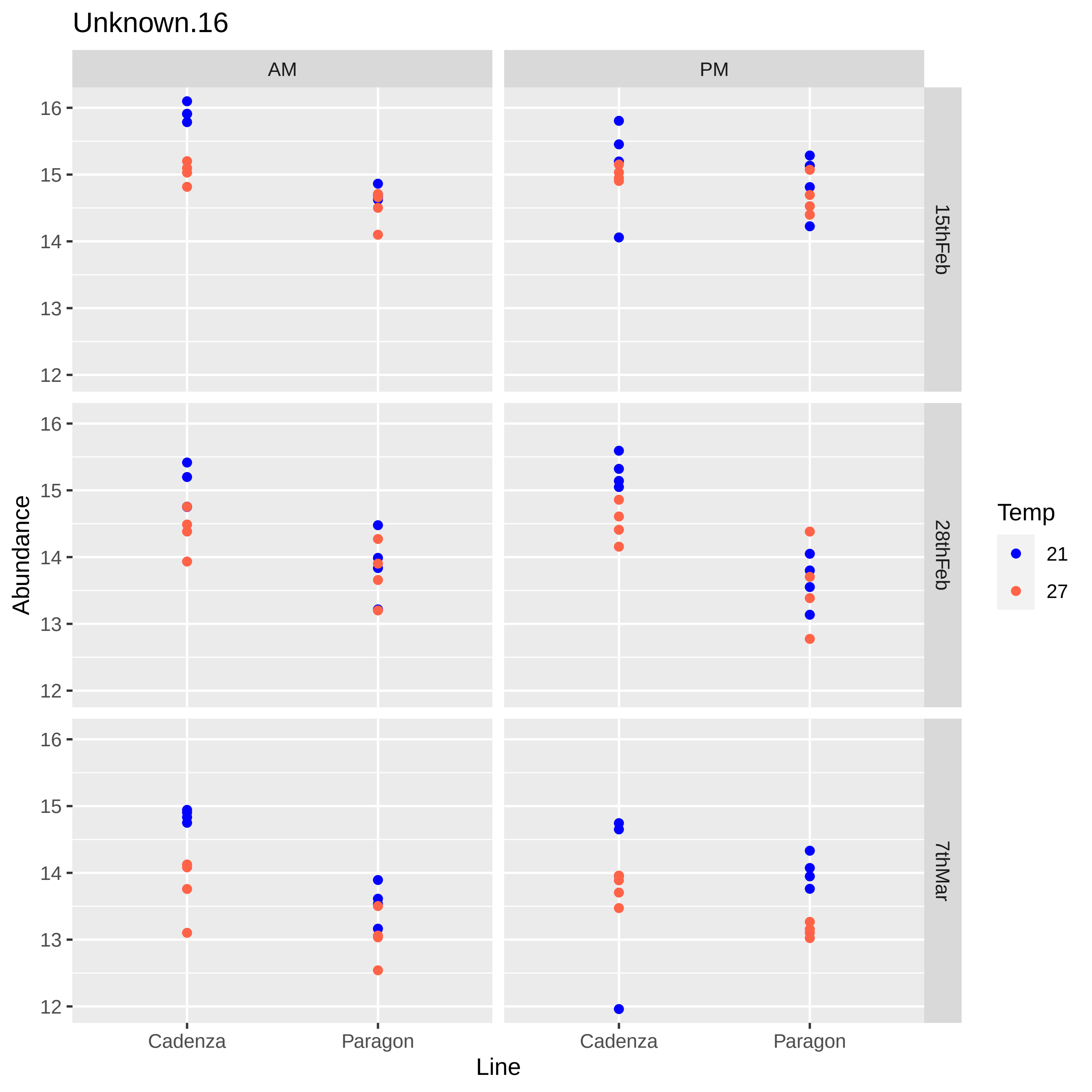

Unknown.16
AM
PM
15thFeb
28thFeb
7thMar
Abundance
16
15
14
13
12
Cadenza
Paragon
Line
Temp
21
27

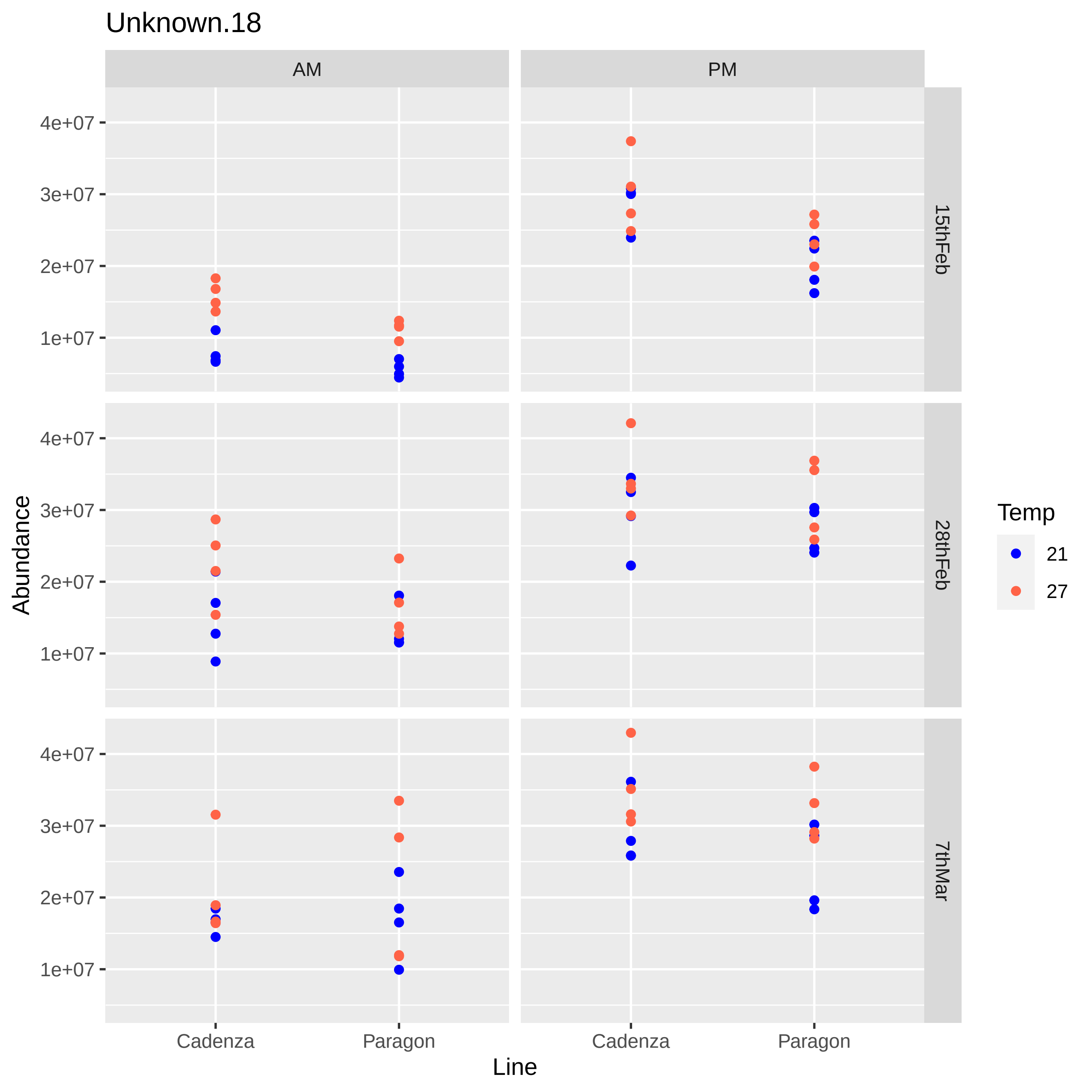

Unknown.18
AM
PM
15thFeb
28thFeb
7thMar
4e+07
3e+07
2e+07
1e+07
Abundance
Cadenza
Paragon
Line
Temp
21
27

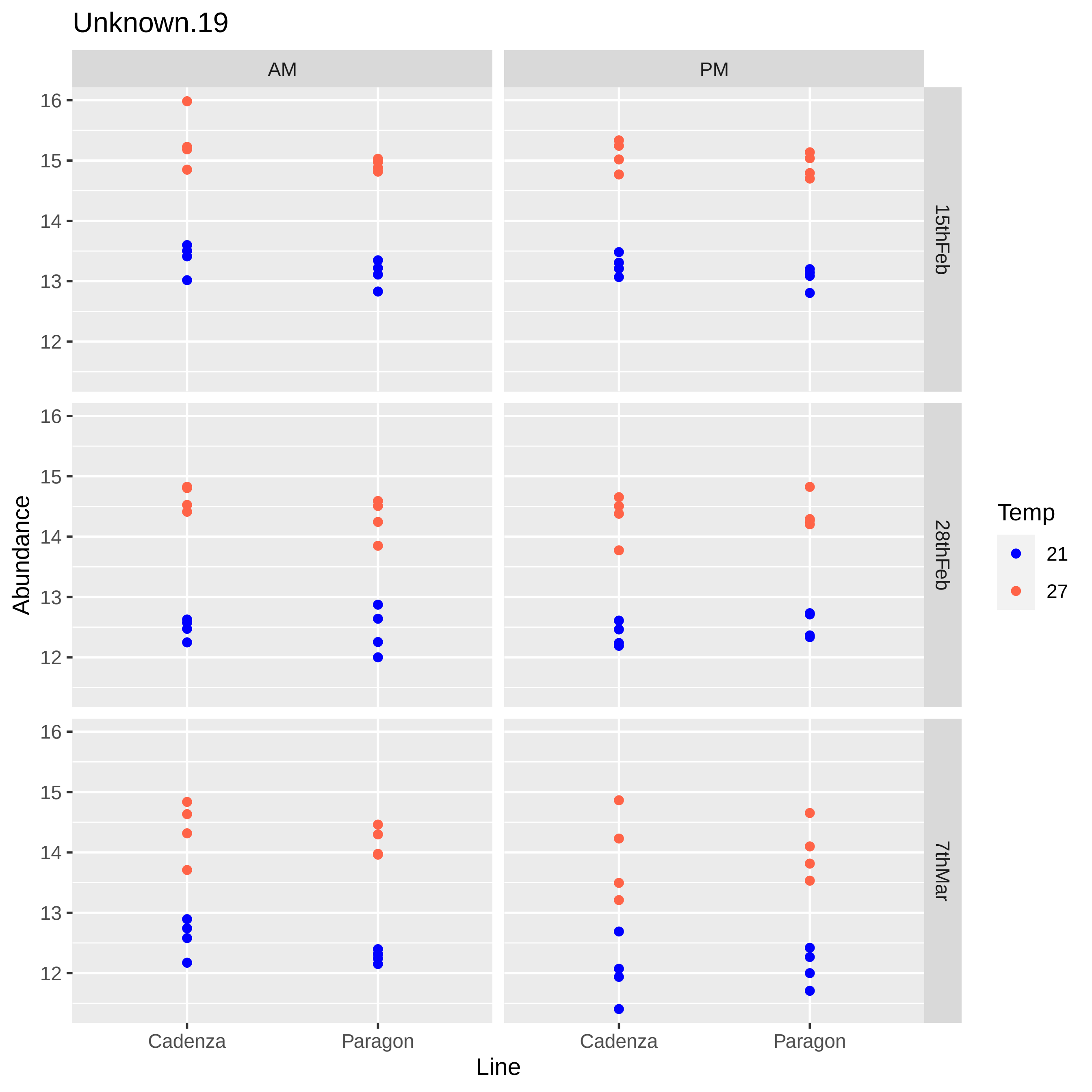
Unknown.19
AM
PM
15thFeb
28thFeb
7thMar
Abundance
16
15
14
13
12
Cadenza
Paragon
Line
Temp
21
27

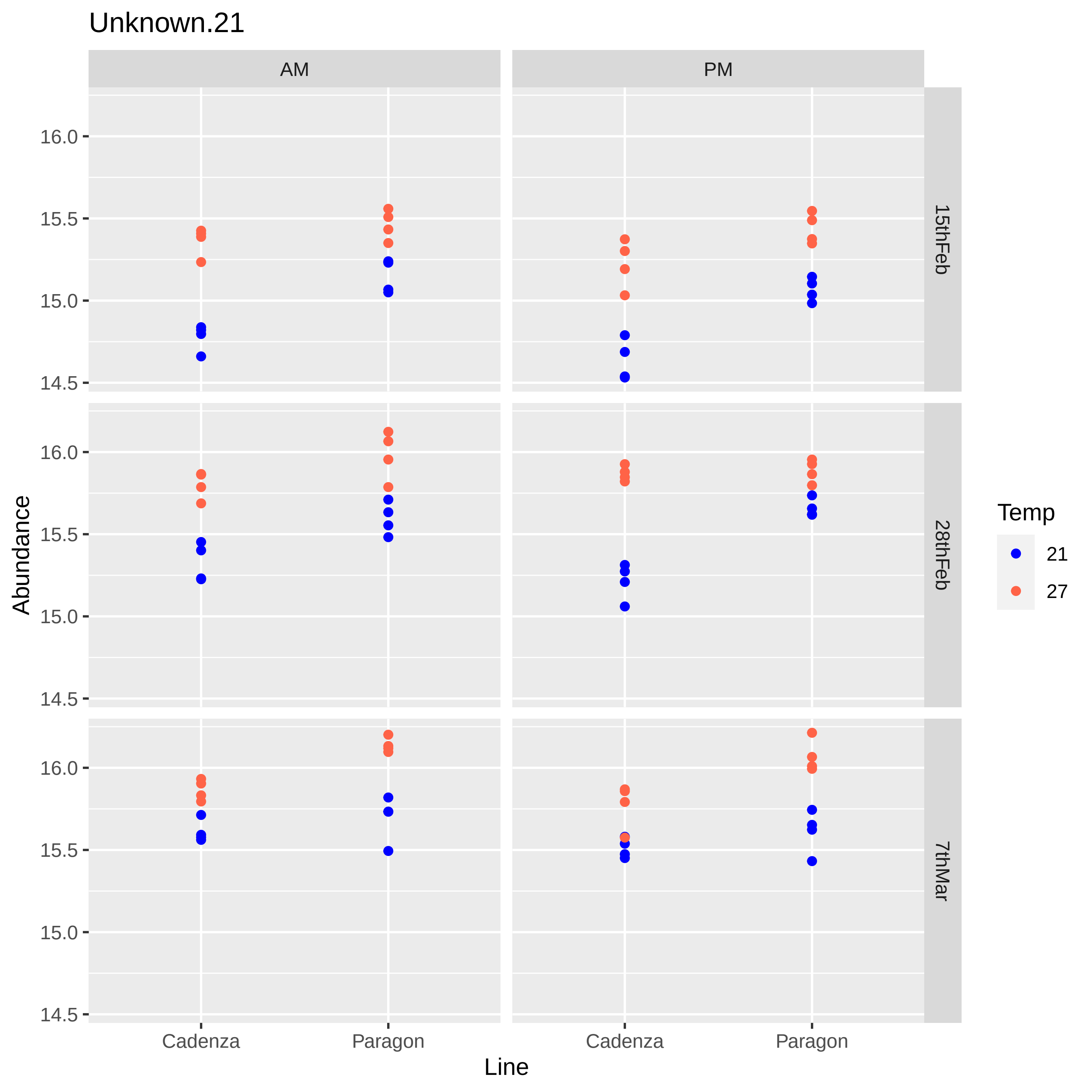

Unknown.21
AM
PM
15thFeb
28thFeb
7thMar
16.0
15.5
15.0
14.5
Abundance
Cadenza
Paragon
Line
Temp
21
27

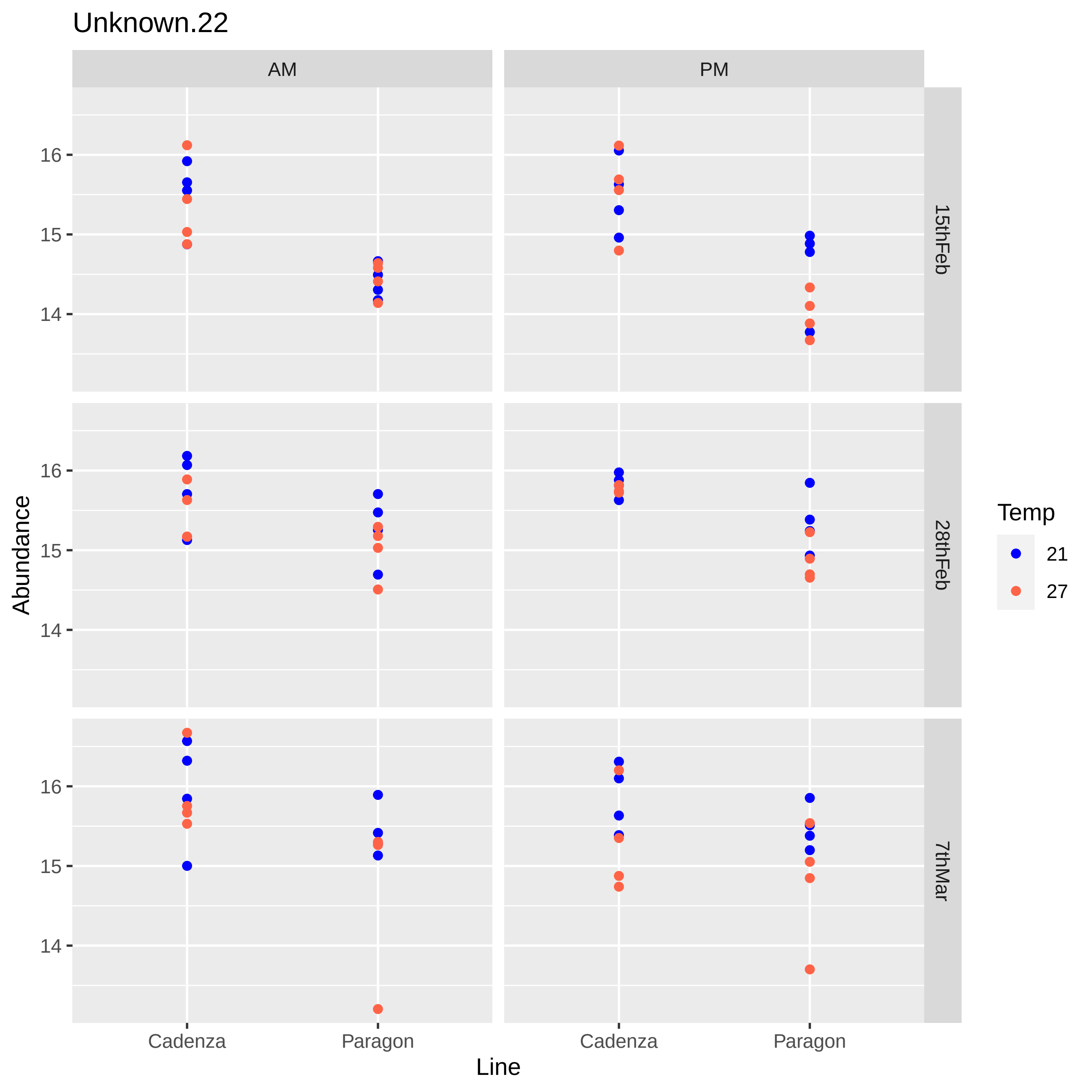

Unknown.22
AM
PM
15thFeb
28thFeb
7thMar
16
15
14
Abundance
Cadenza
Paragon
Line
Temp
21
27

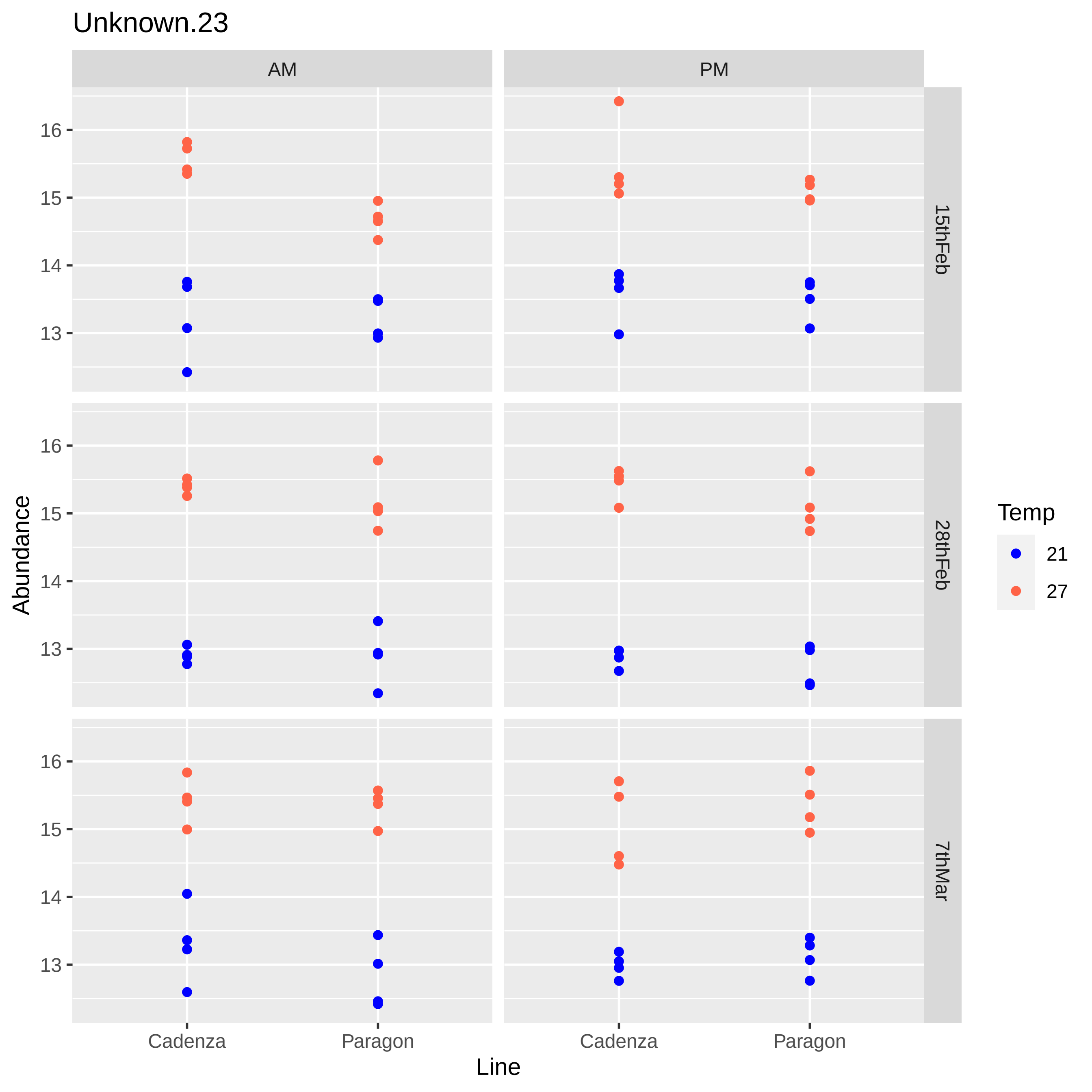
Unknown.23
AM
PM
15thFeb
28thFeb
7thMar
16
15
14
13
Abundance
Cadenza
Paragon
Line
Temp
21
27

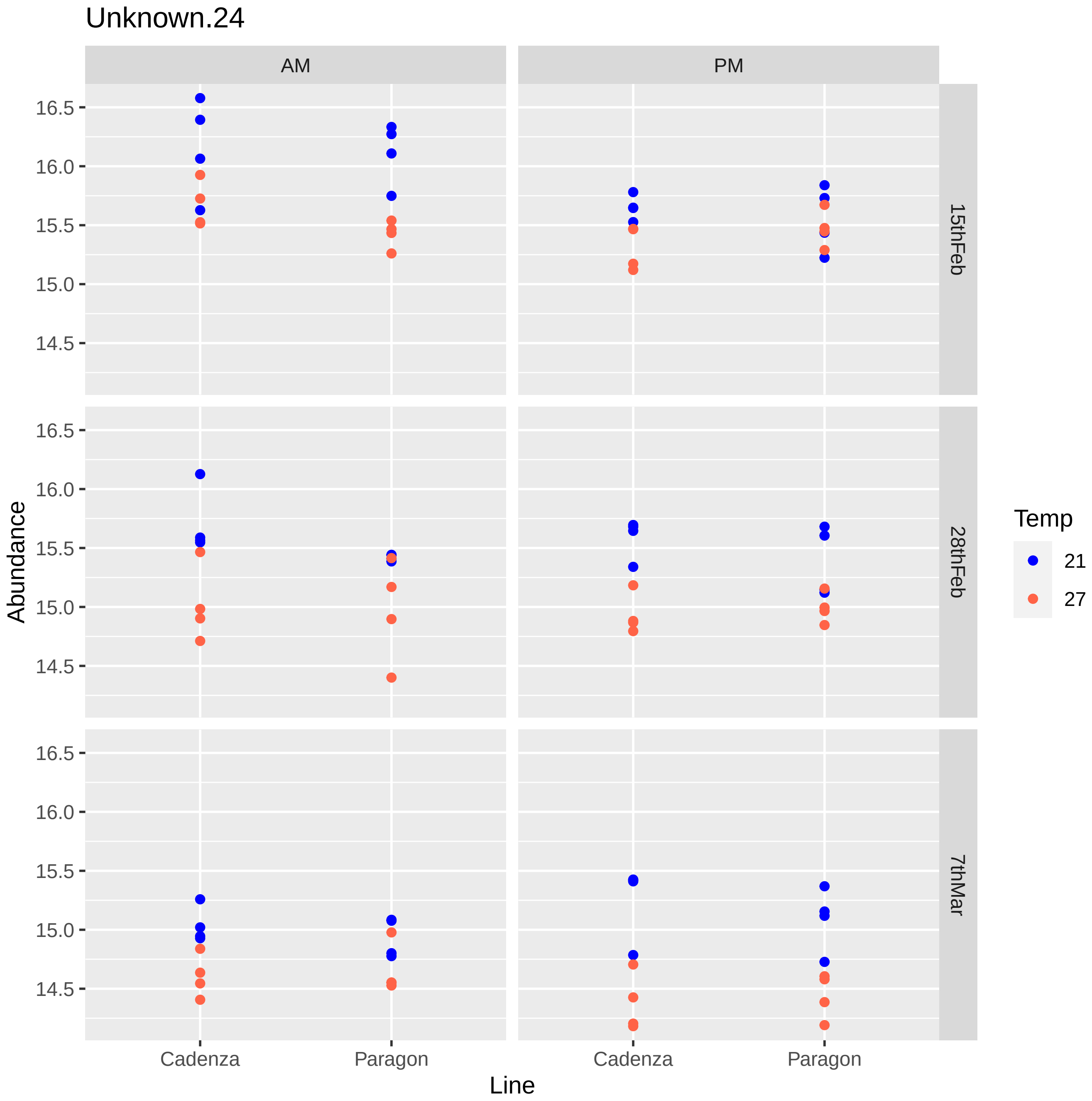
Unknown.24
AM
PM
15thFeb
28thFeb
7thMar
16.5
16.0
15.5
15.0
14.5
Abundance
Cadenza
Paragon
Line
Temp
21
27

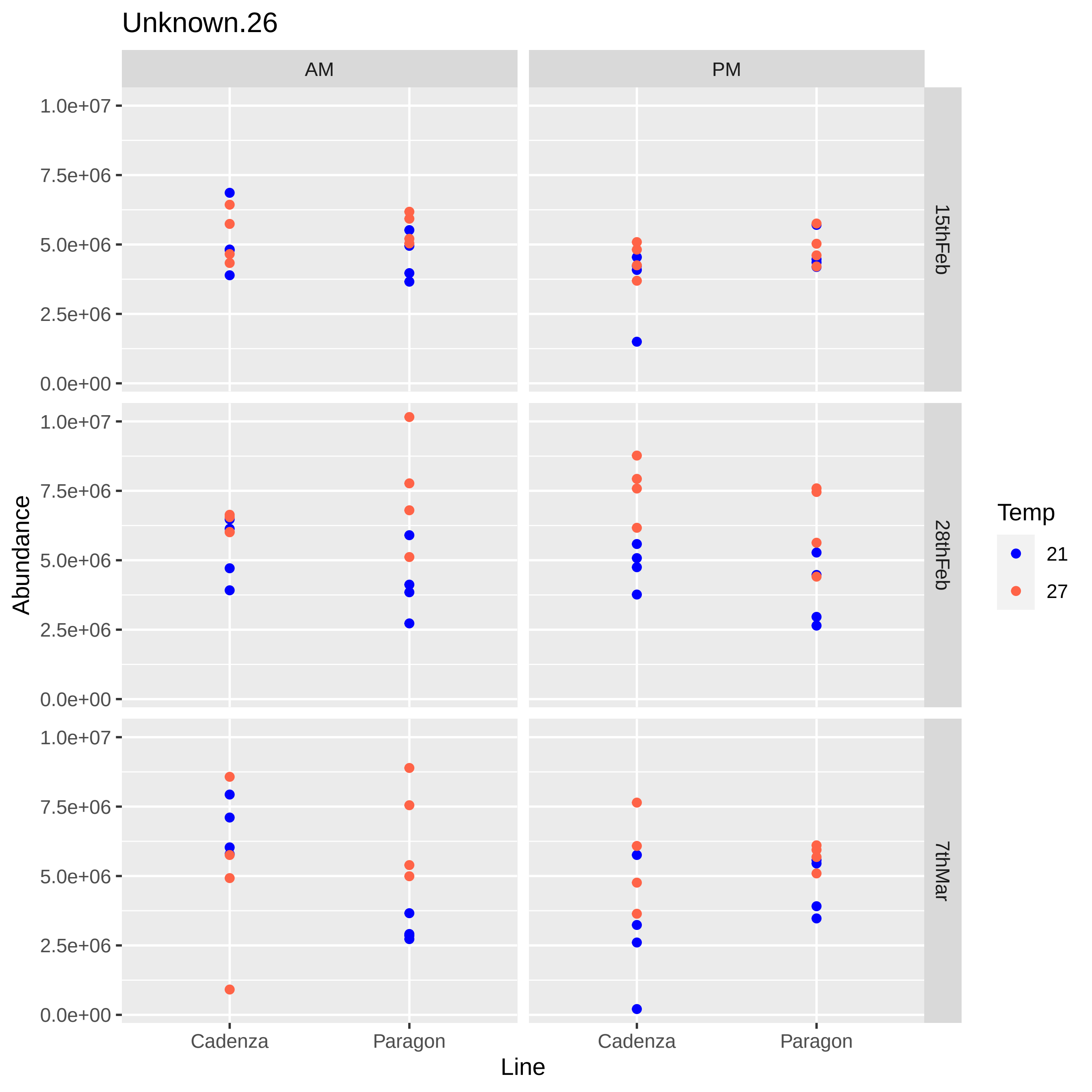

Unknown.26
AM
PM
15thFeb
28thFeb
7thMar
1.0e+07
7.5e+06
5.0e+06
2.5e+06
0.0e+00
Abundance
Cadenza
Paragon
Line
Temp
21
27

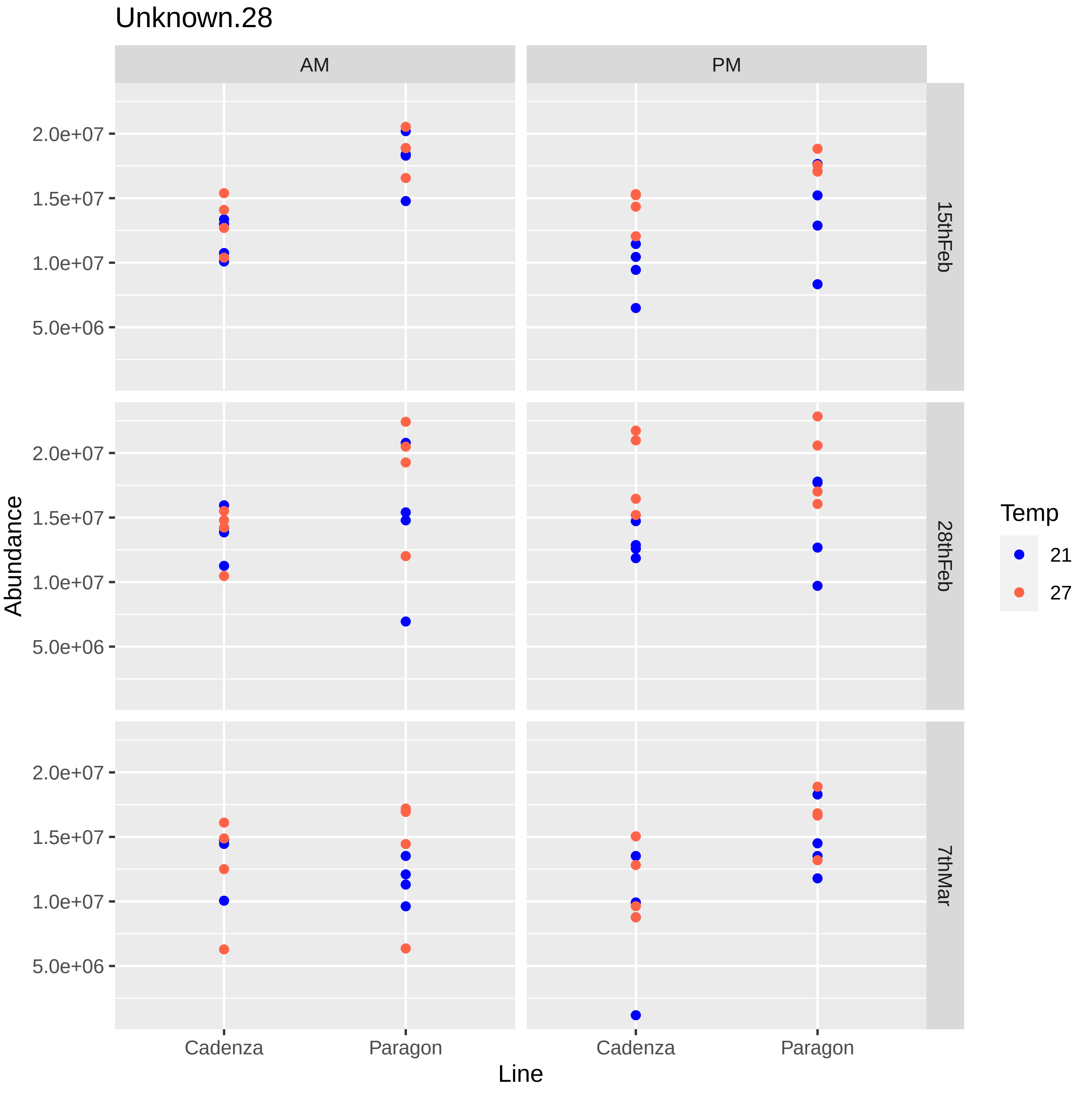
Unknown.28
AM
PM
15thFeb
28thFeb
7thMar
Abundance
2.0e+07
1.5e+07
1.0e+07
5.0e+06
Cadenza
Paragon
Line
Temp
21
27

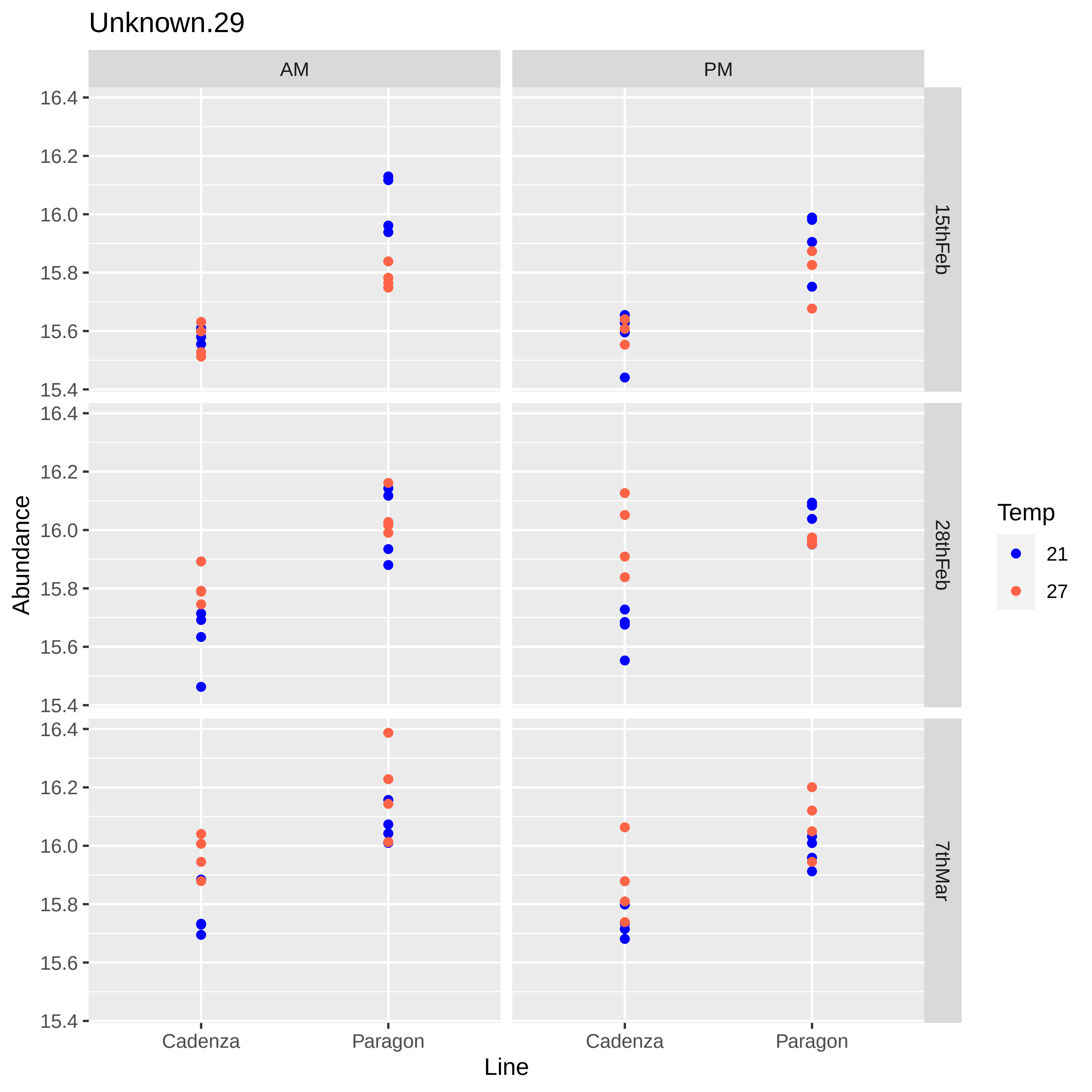

Unknown.29
AM
PM
15thFeb
28thFeb
7thMar
16.4
16.2
16.0
15.8
15.6
15.4
Abundance
Cadenza
Paragon
Line
Temp
21
27

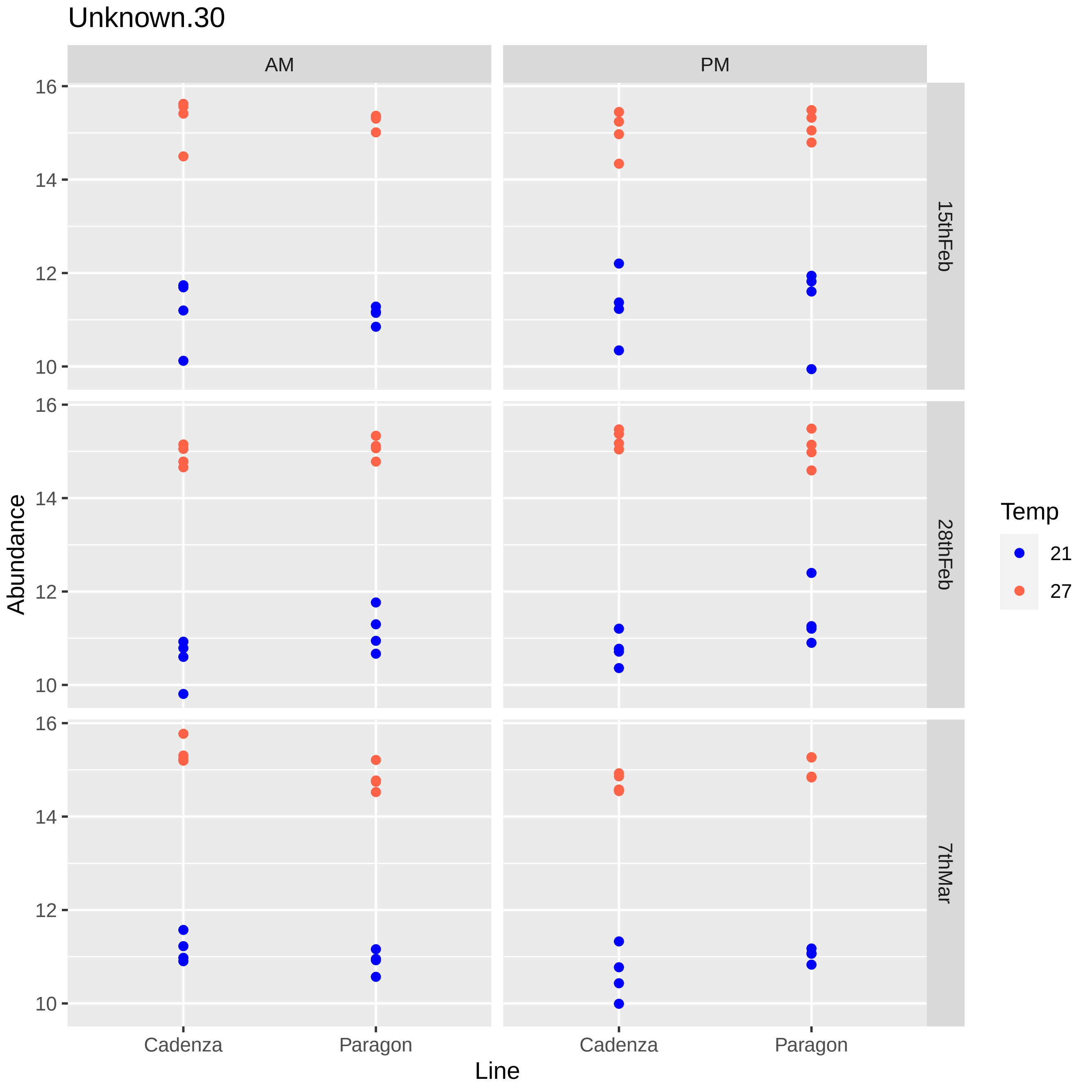

Unknown.30
AM
PM
15thFeb
28thFeb
7thMar
Abundance
16
14
12
10
Cadenza
Paragon
Line
Temp
21
27

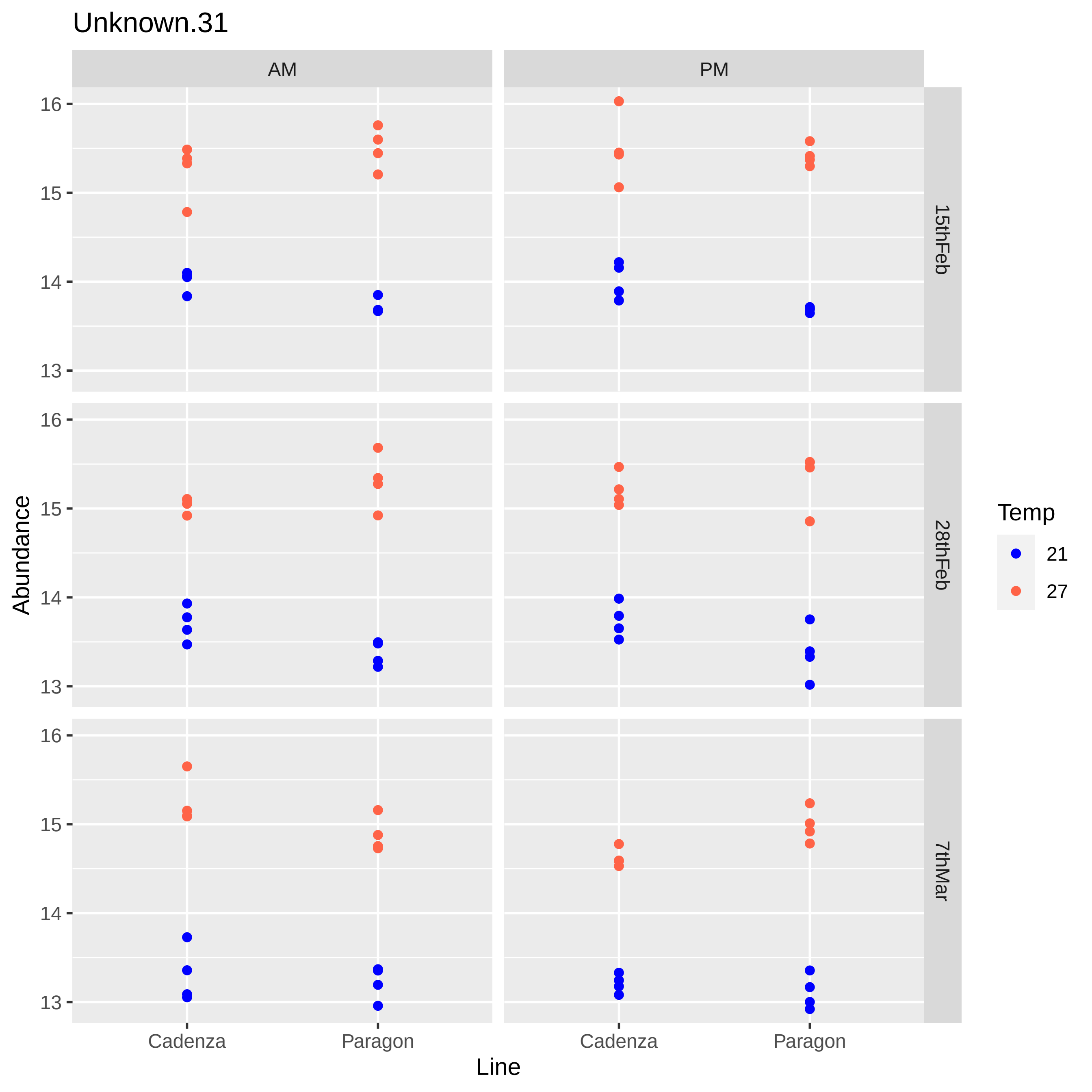
Unknown.31
AM
PM
15thFeb
28thFeb
7thMar
Abundance
16
15
14
13
Cadenza
Paragon
Line
Temp
21
27

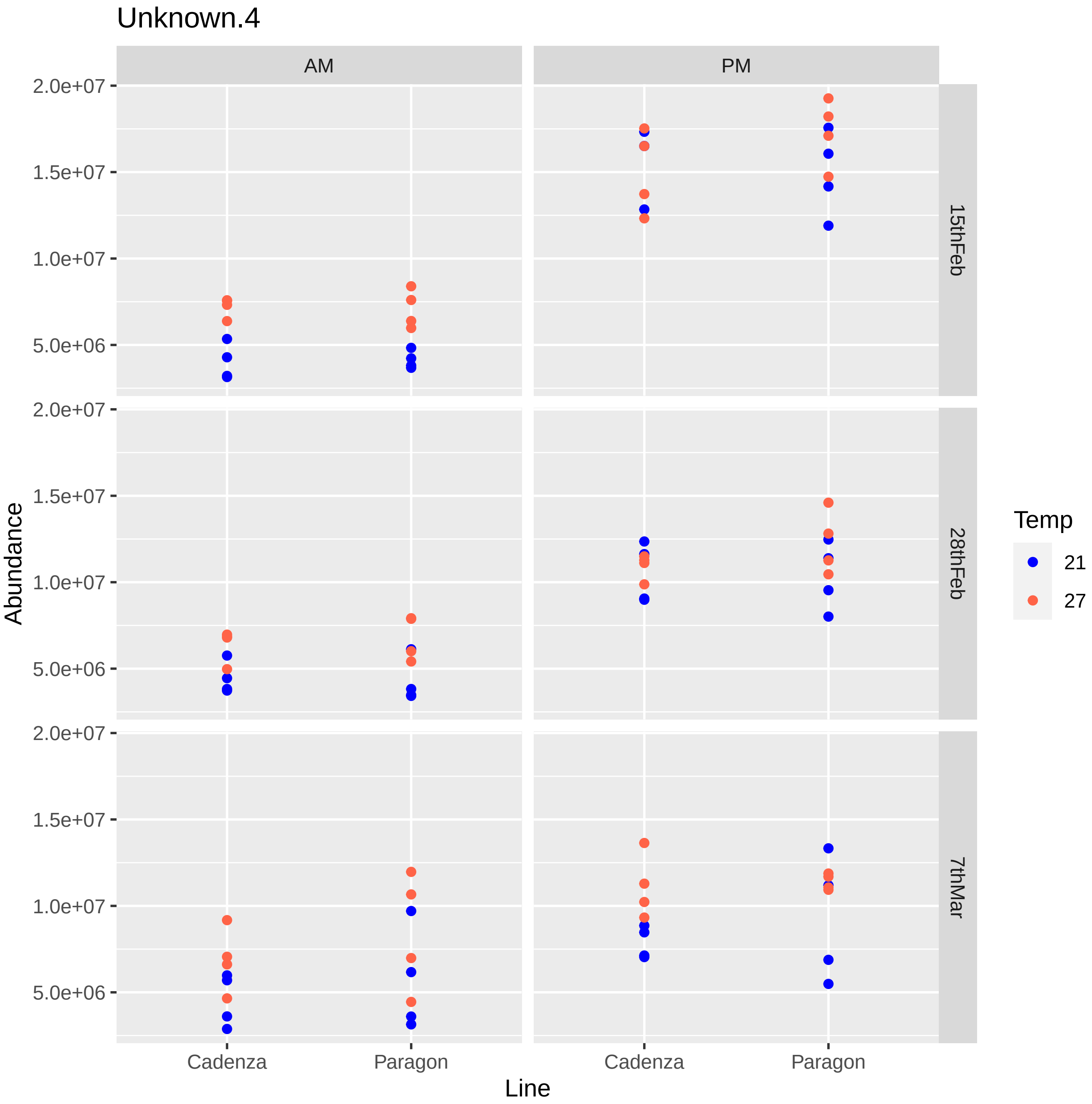

Unknown.4
AM
PM
15thFeb
28thFeb
7thMar
2.0e+07
1.5e+07
1.0e+07
5.0e+06
Abundance
Cadenza
Paragon
Line
Temp
21
27

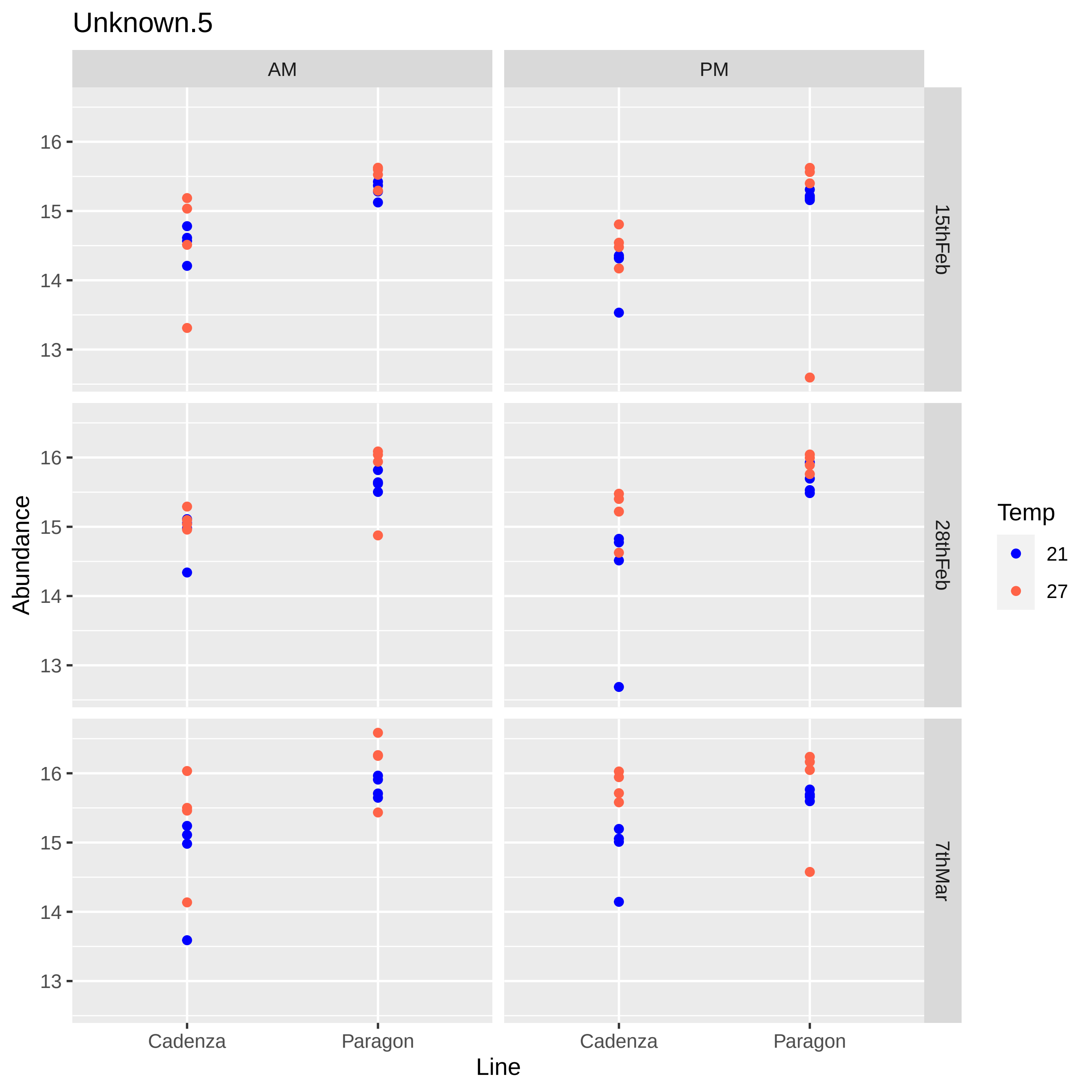

Unknown.5
AM
PM
15thFeb
28thFeb
7thMar
16
15
14
13
Abundance
Cadenza
Paragon
Line
Temp
21
27

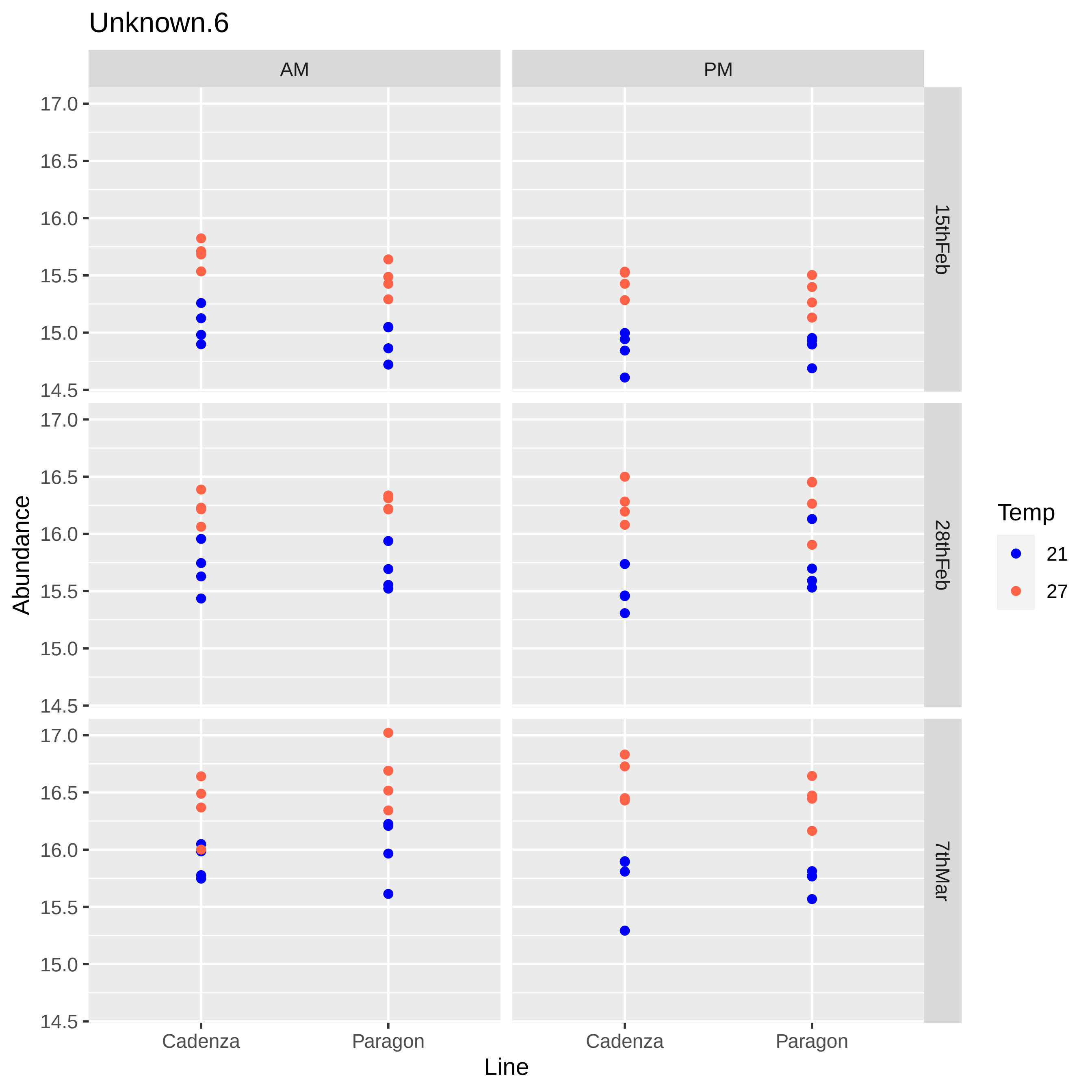

Unknown.6
AM
PM
15thFeb
28thFeb
7thMar
17.0
16.5
16.0
15.5
15.0
14.5
Abundance
Cadenza
Paragon
Line
Temp
21
27

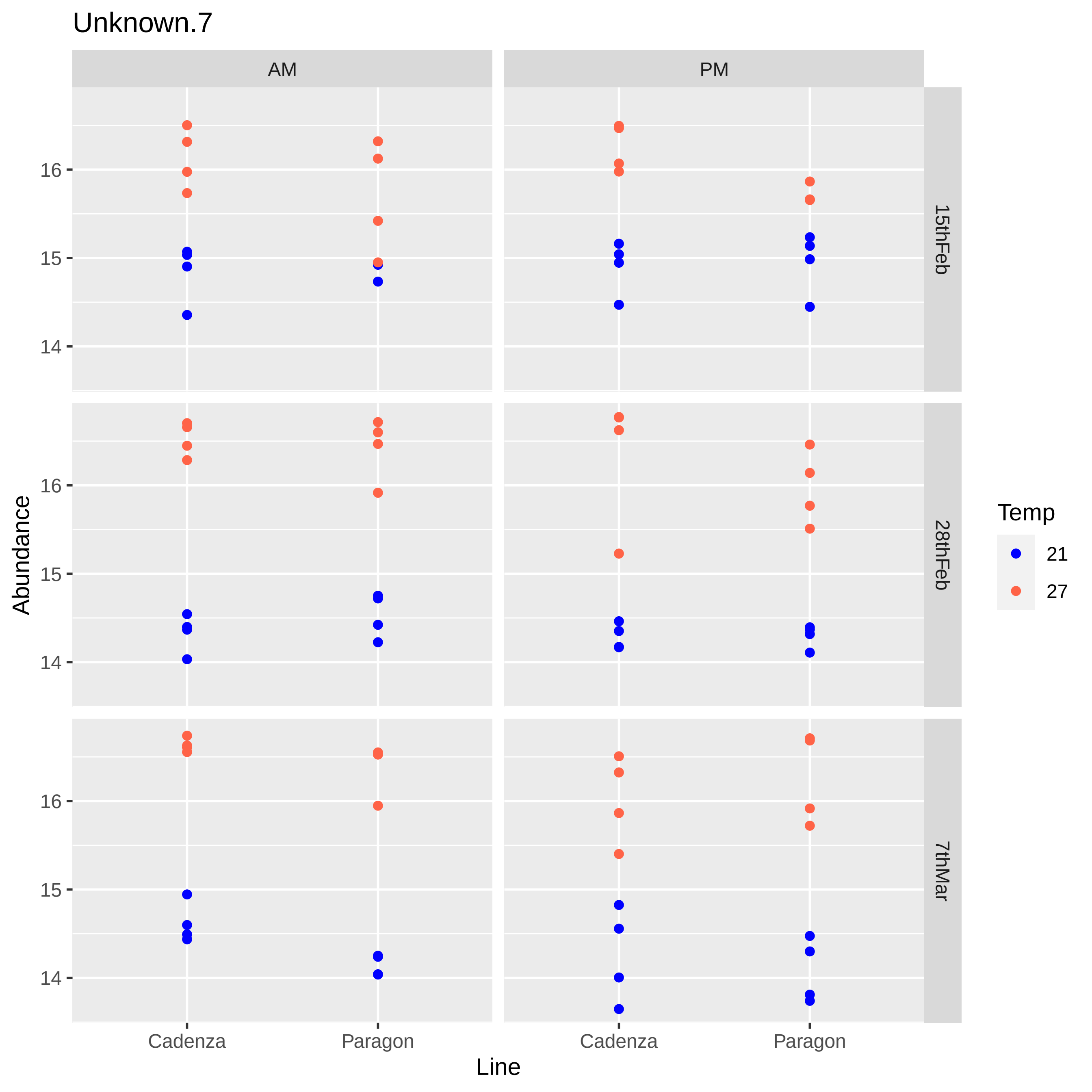

Unknown.7
AM
PM
15thFeb
28thFeb
7thMar
Abundance
14
15
16
Cadenza
Paragon
Line
Temp
21
27

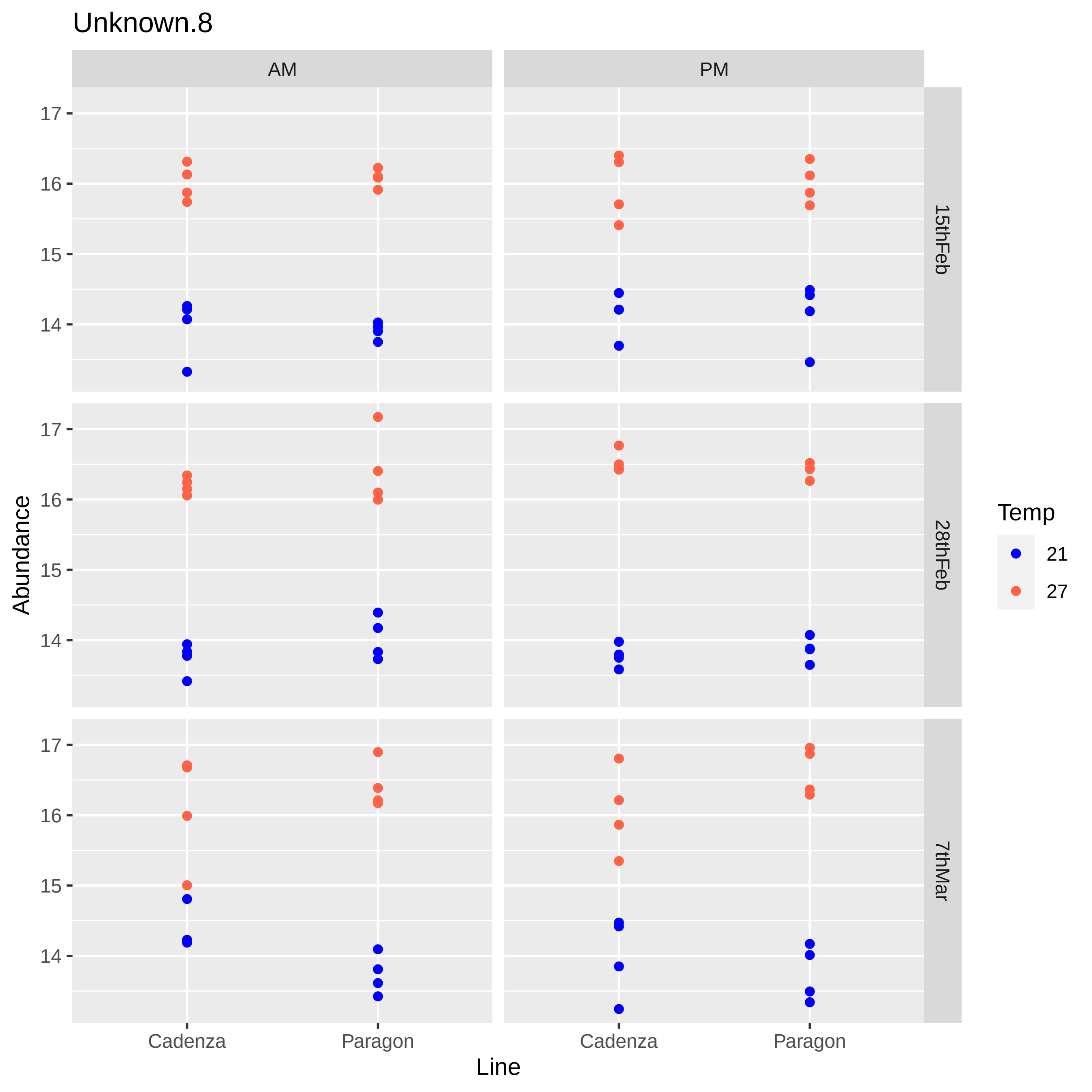

Unknown.8
AM
PM
15thFeb
28thFeb
7thMar
Abundance
Line
Cadenza
Paragon
Cadenza
Paragon
14
15
16
17
Temp
21
27

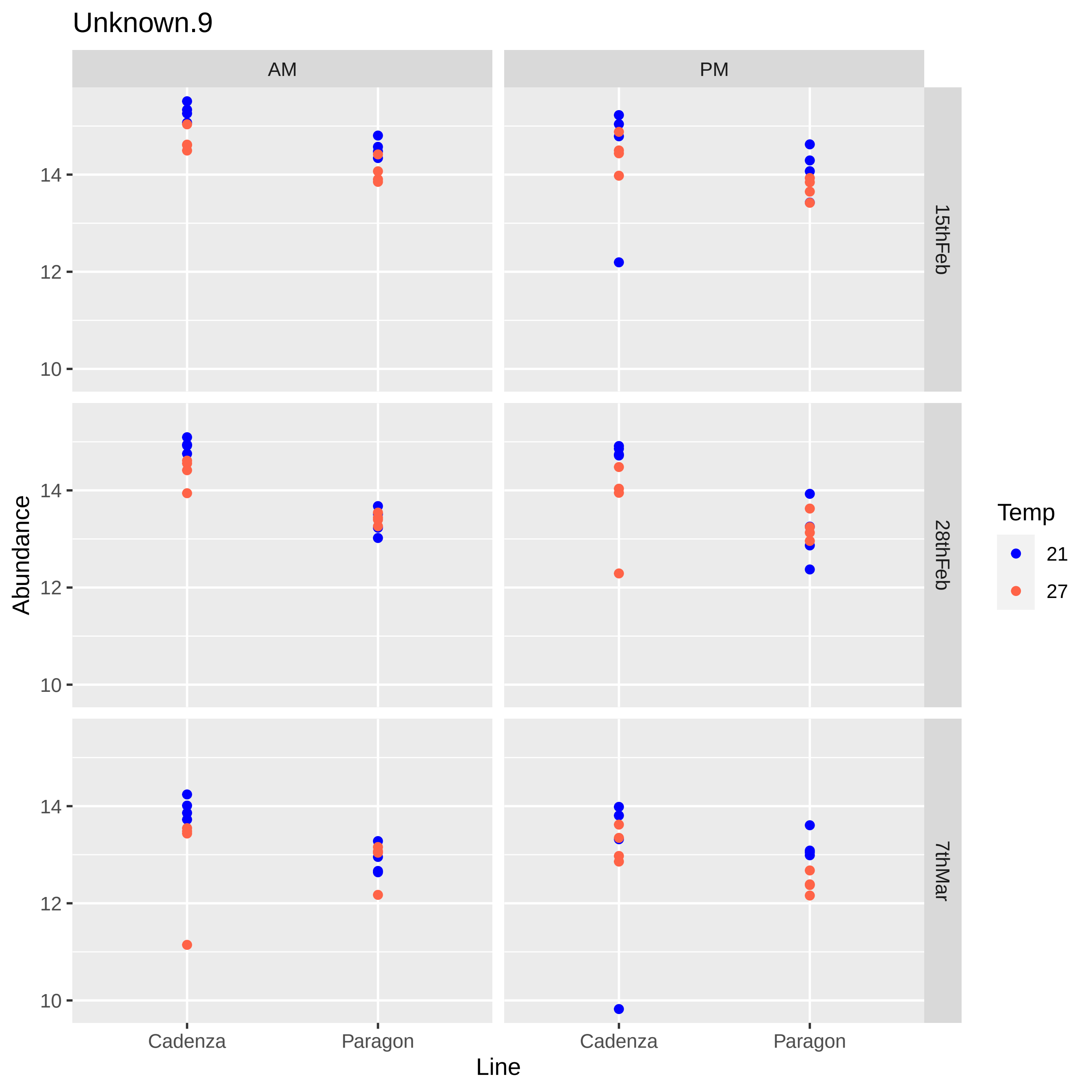
Unknown.9
AM
PM
15thFeb
28thFeb
7thMar
Abundance
10
12
14
Cadenza
Paragon
Line
Temp
21
27

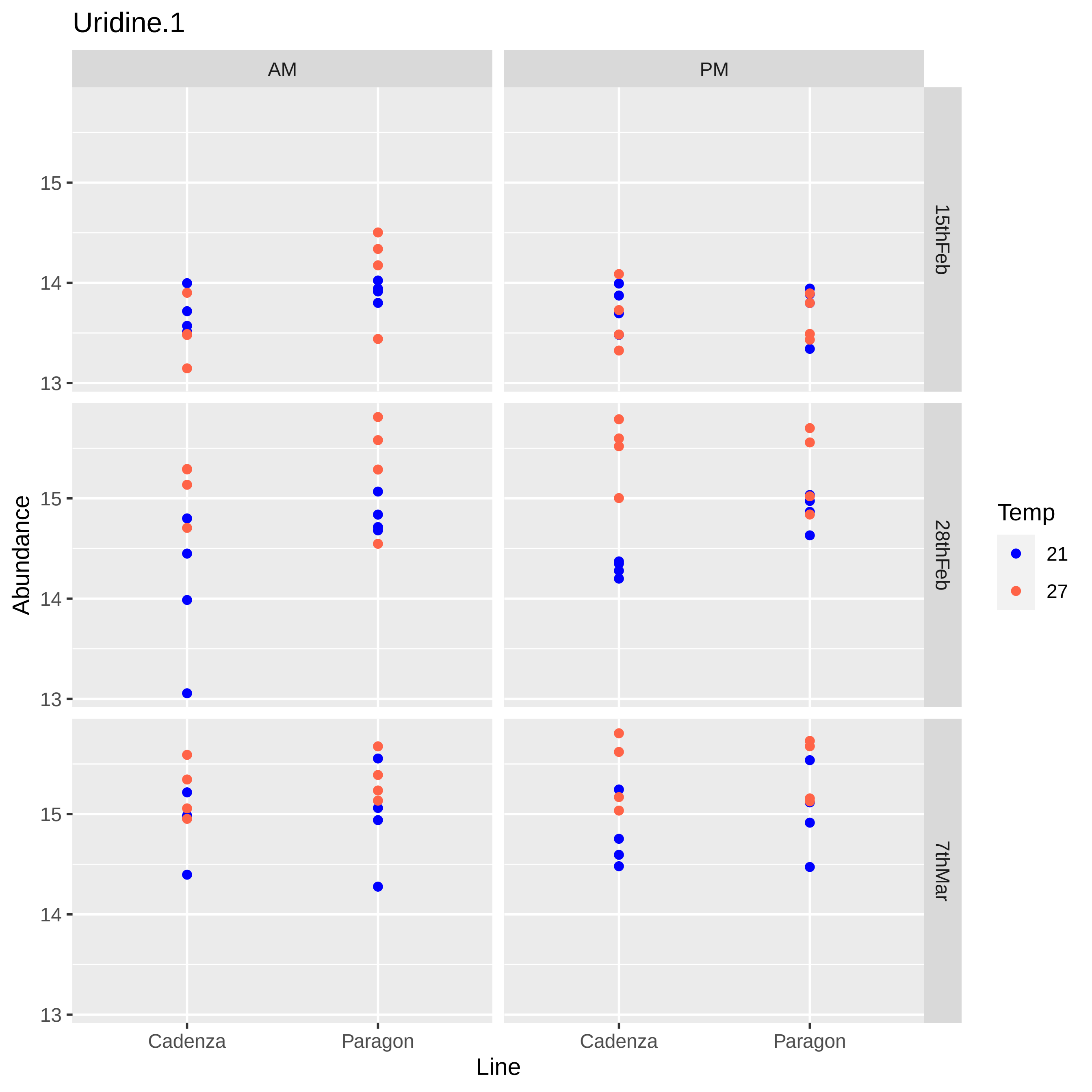
Uridine.1
AM
PM
15thFeb
28thFeb
7thMar
Abundance
13
14
15
Line
Cadenza
Paragon
Temp
21
27

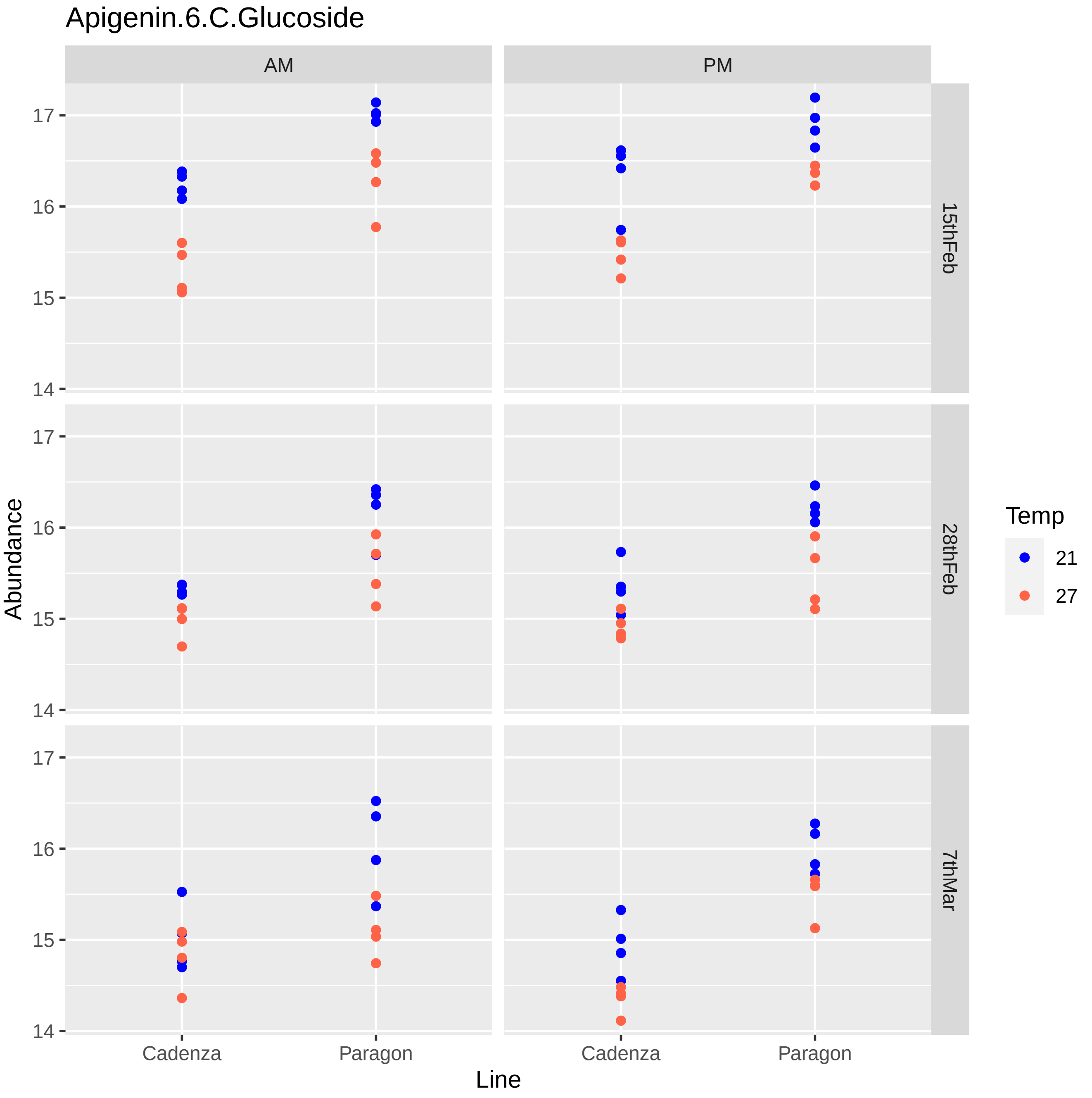

Apigenin.6.C.Glucoside
AM
PM
15thFeb
28thFeb
7thMar
Abundance
17
16
15
14
Cadenza
Paragon
Line
Temp
21
27

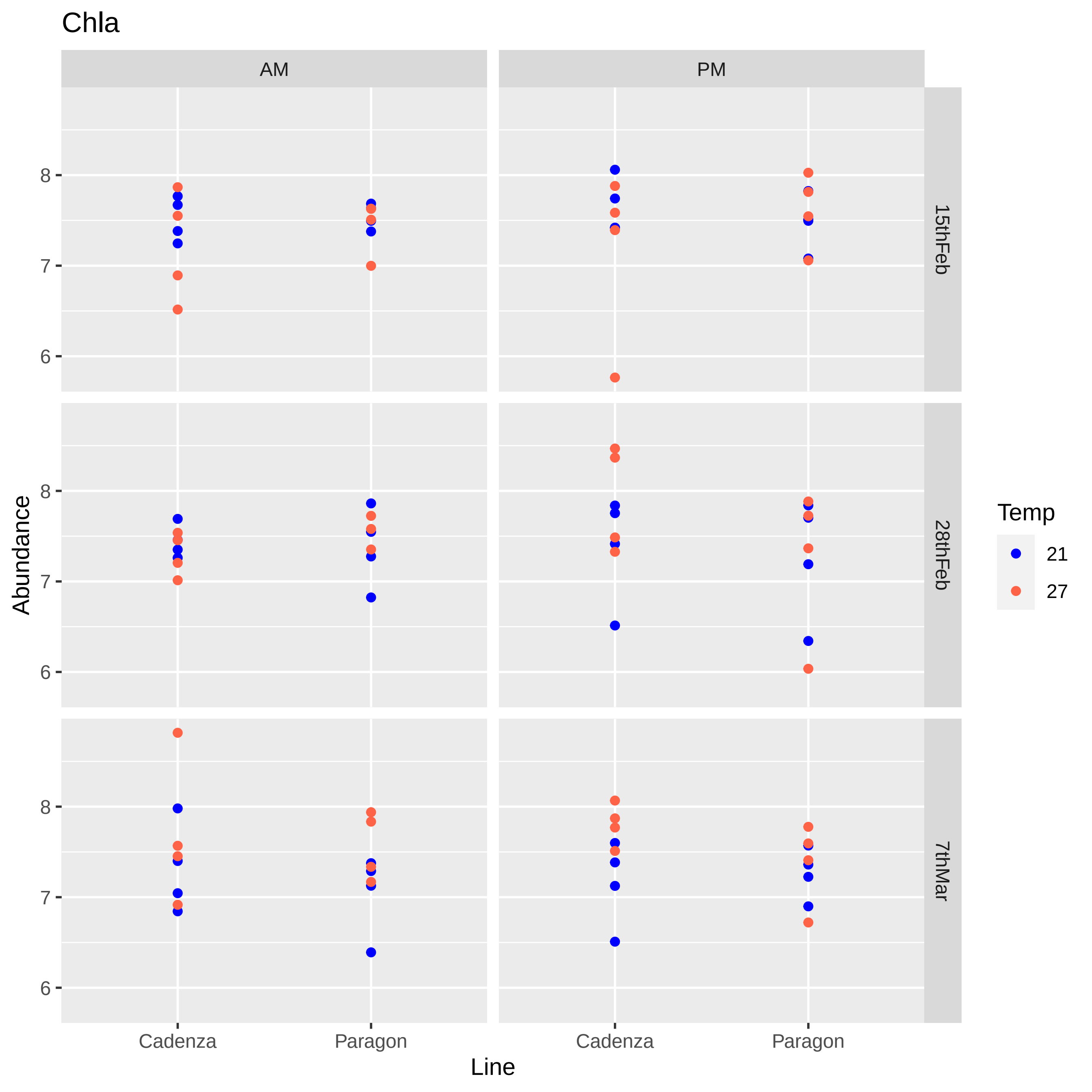

Chla
AM
PM
15thFeb
28thFeb
7thMar
Abundance
8
7
6
Cadenza
Paragon
Line
Temp
21
27

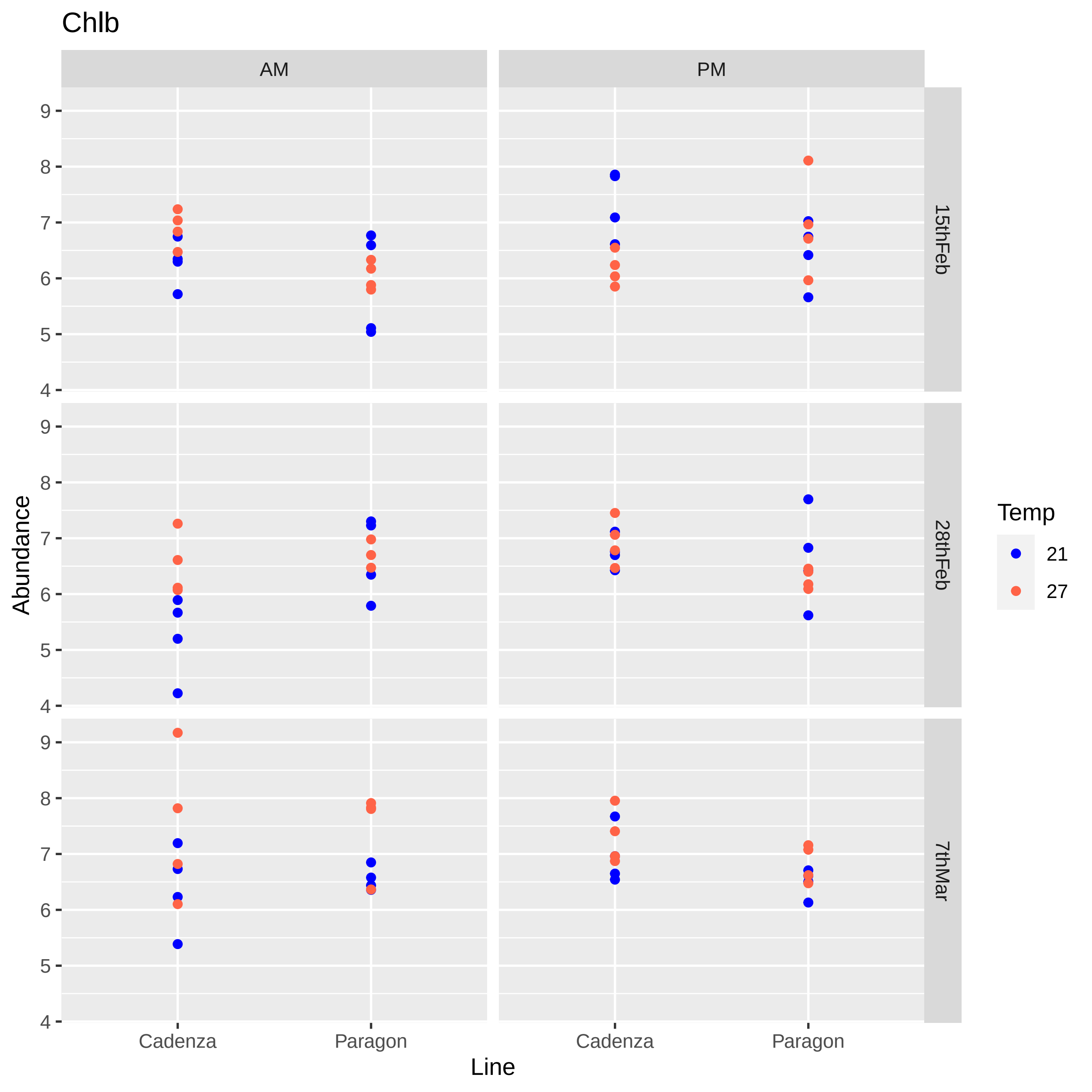

Chlb
AM
PM
15thFeb
28thFeb
7thMar
Abundance
Line
Cadenza
Paragon
Temp
21
27
9
8
7
6
5
4

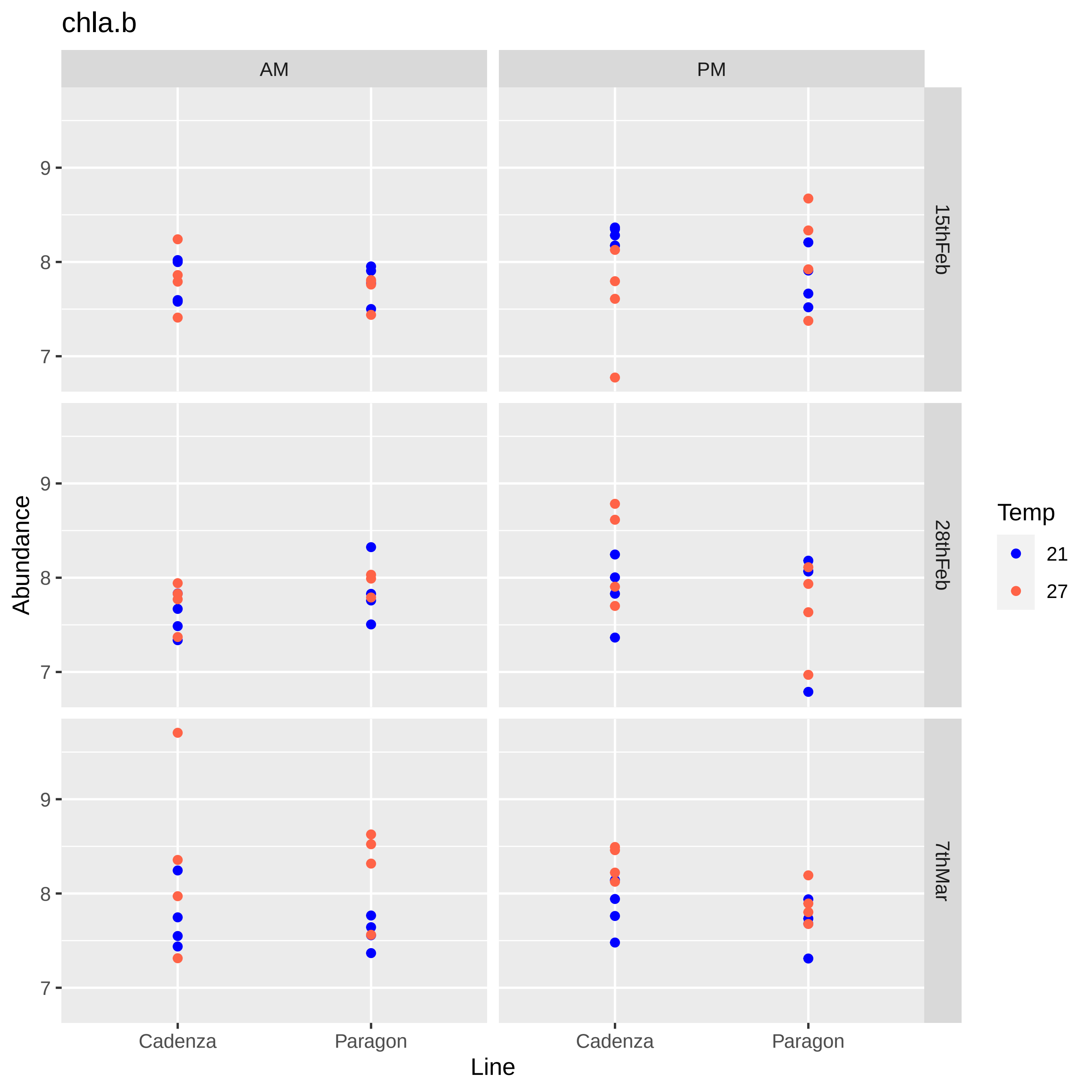
chla.b
AM
PM
15thFeb
28thFeb
7thMar
Abundance
9
8
7
Cadenza
Paragon
Line
Temp
21
27

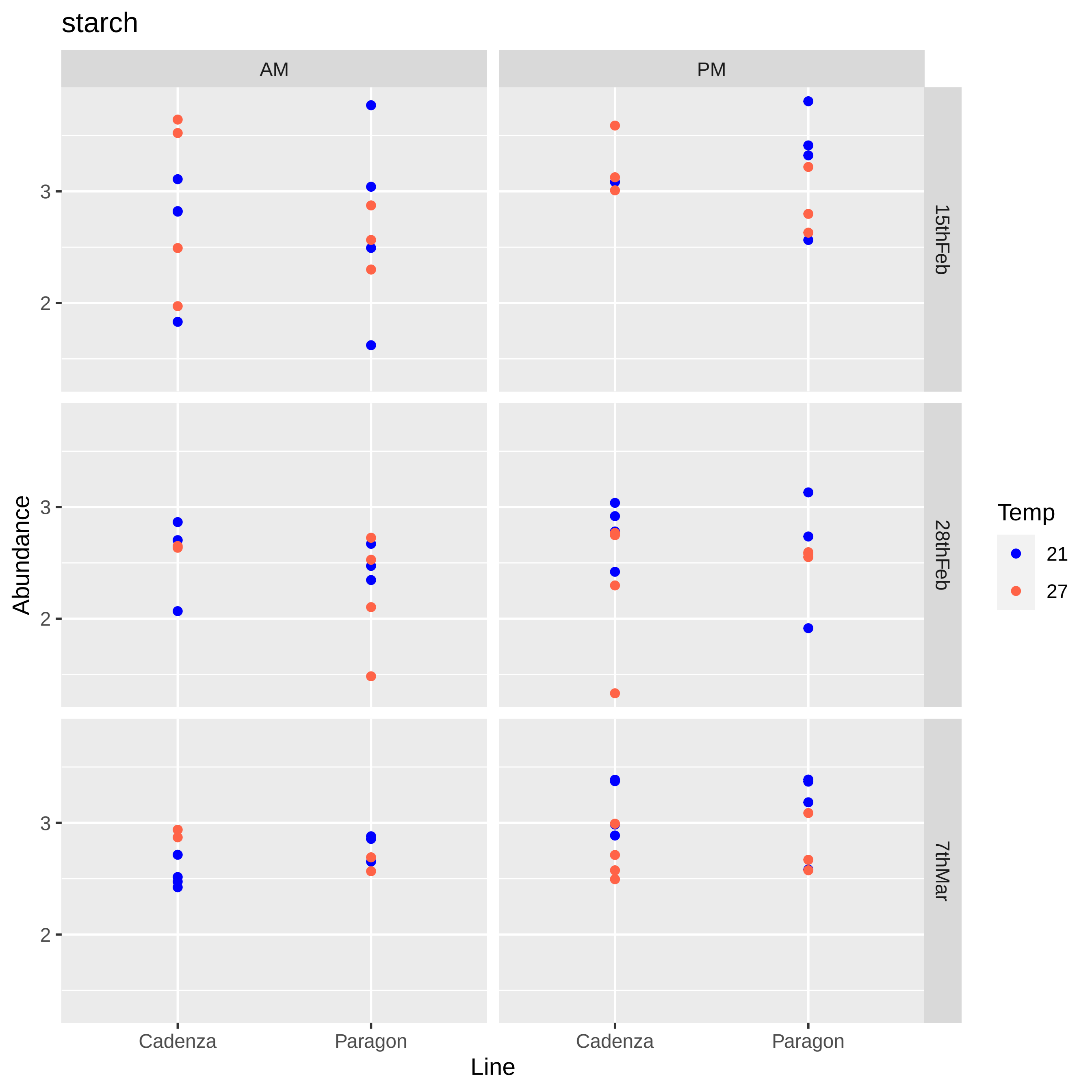
starch
AM
PM
15thFeb
28thFeb
7thMar
Abundance
2
3
Cadenza
Paragon
Line
Temp
21
27
