# Supplementary material for: Novel molecules and target genes for vegetative heat tolerance in wheat
Source: Plant Environ Interact. 2022 Dec 26;3(6):264–89. doi: 10.1002/pei3.10096 (PMC10168084; doi:10.1002/pei3.10096)

LICOR data

2022-11-16

Photosynthesis

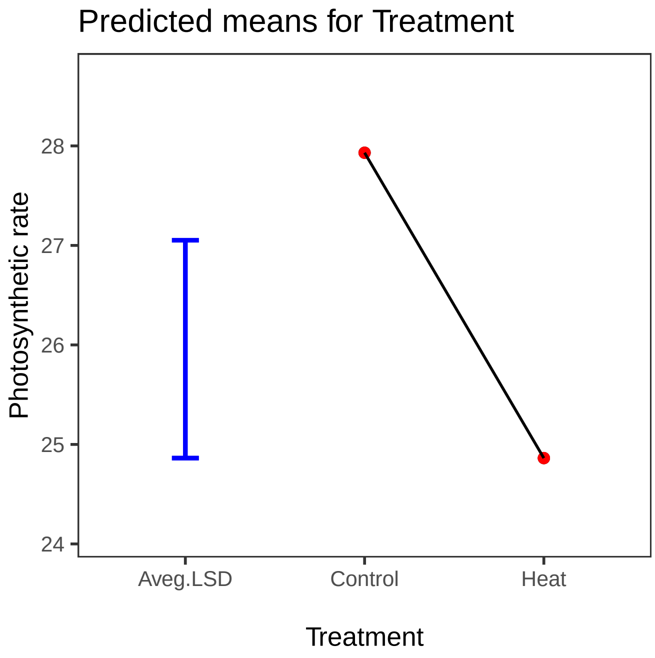

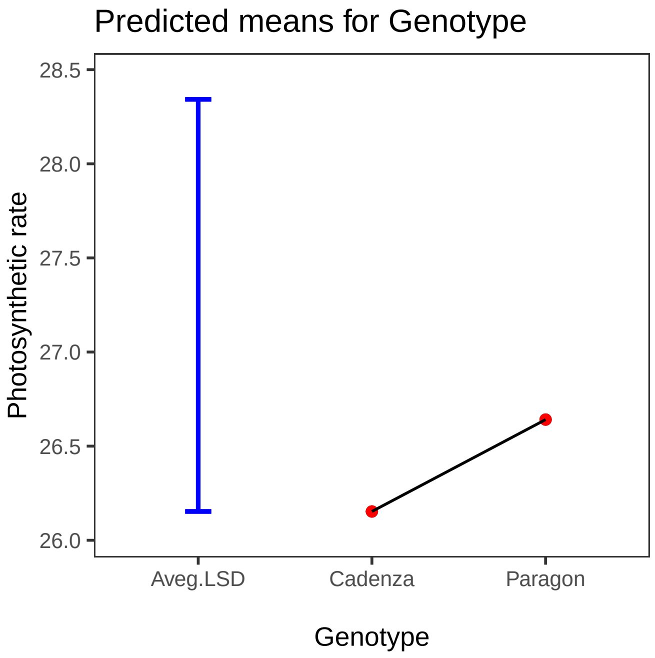

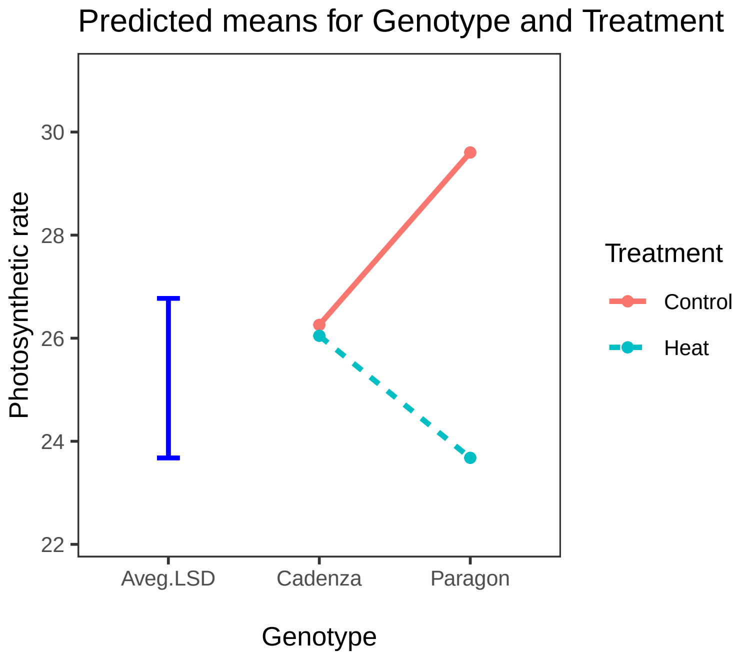

Respiration

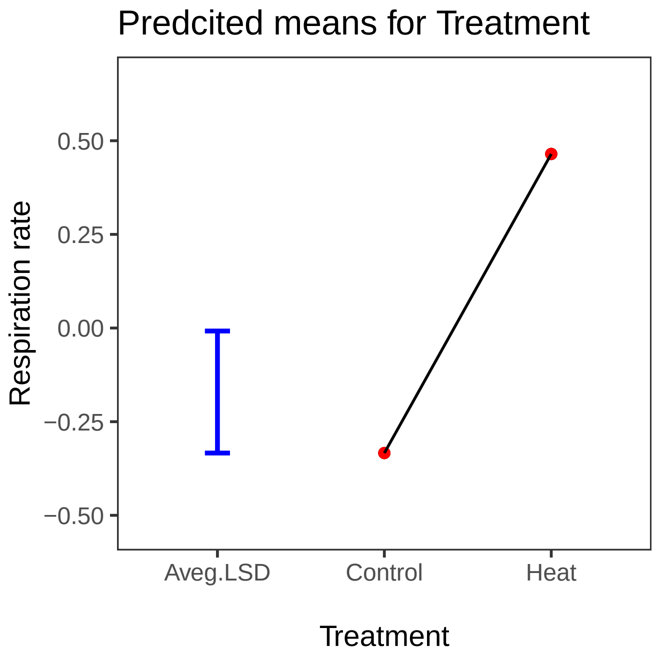

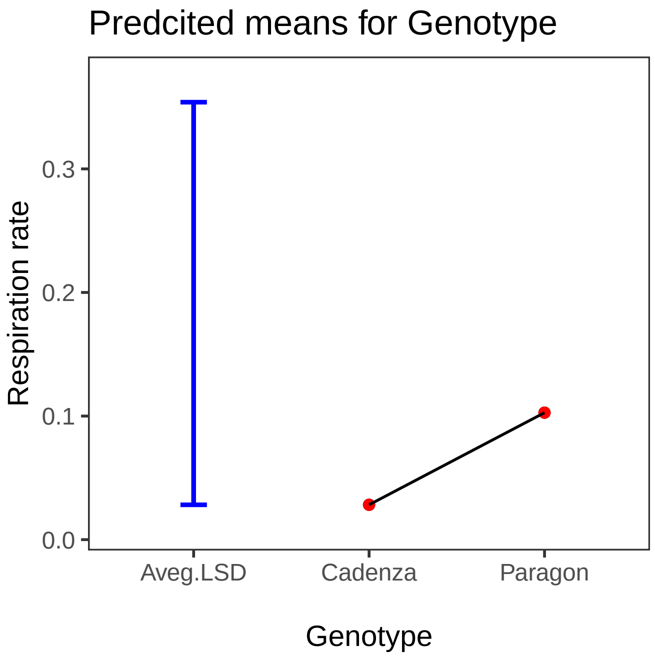

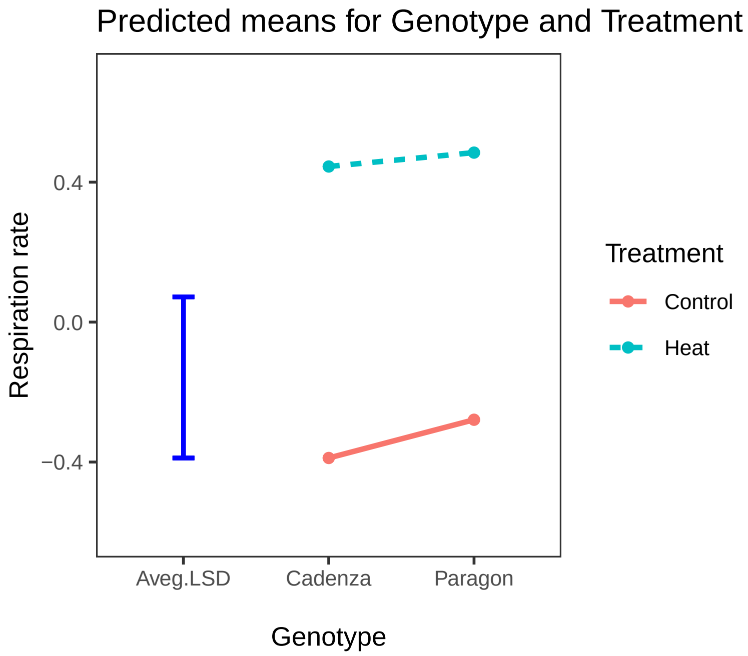

ΦPSII

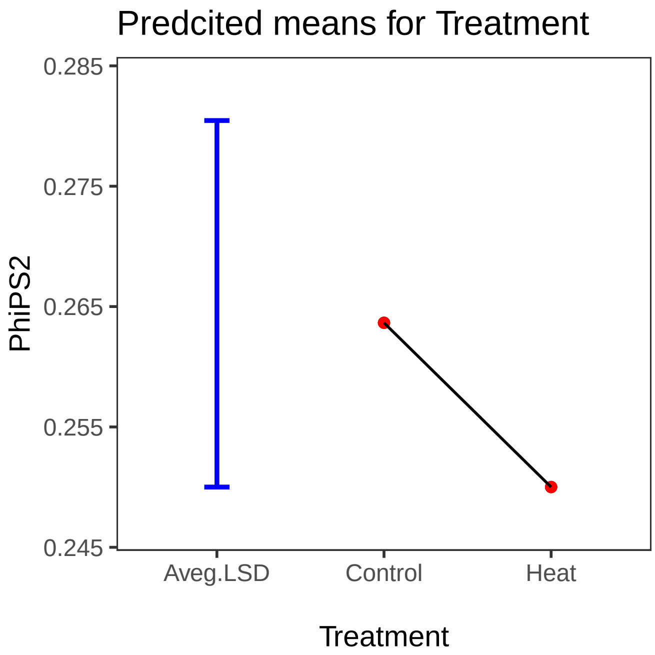
Predcited means for Treatment
0.285
0.275
0.265
0.255
0.245
PhiPS2
Aveg.LSD
Control
Heat
Treatment

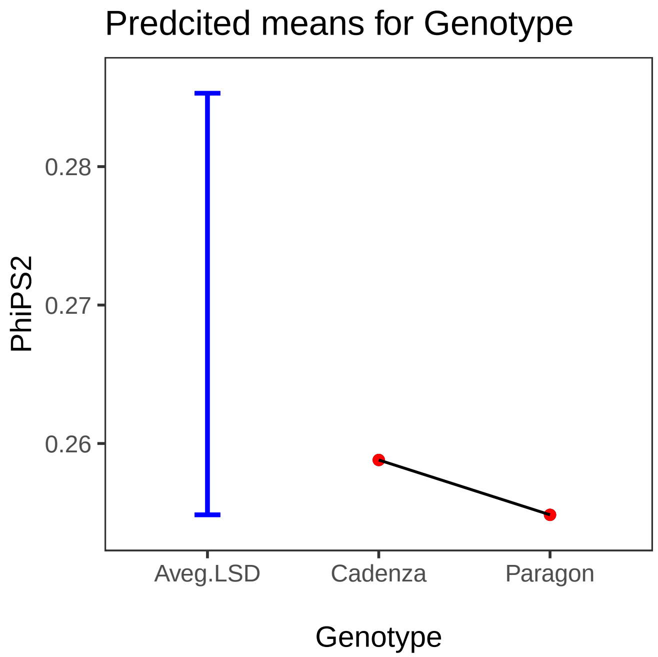
Predcited means for Genotype
0.28
0.27
0.26
PhiPS2
Aveg.LSD
Cadenza
Paragon
Genotype

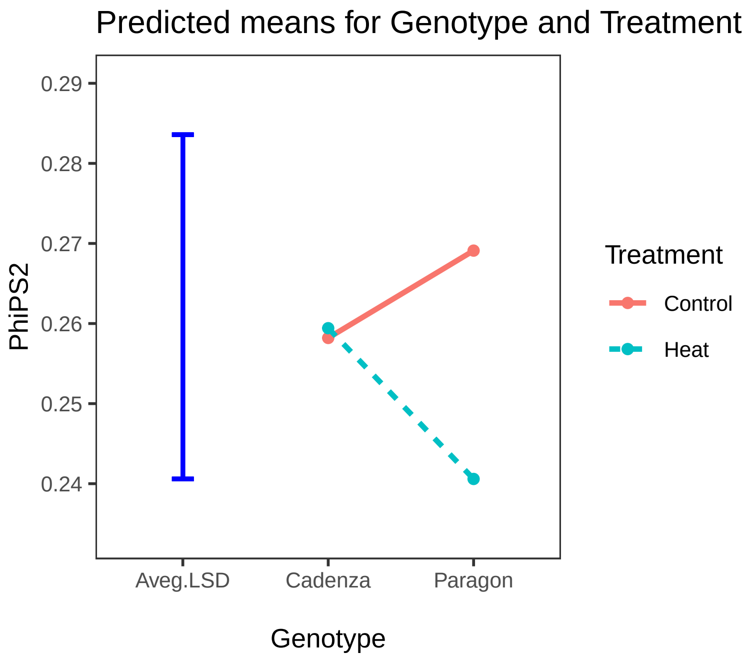
Predicted means for Genotype and Treatment
0.29
0.28
0.27
0.26
0.25
0.24
PhiPS2
Aveg.LSD
Cadenza
Paragon
Genotype
Treatment
Control
Heat

Fv'/Fm'

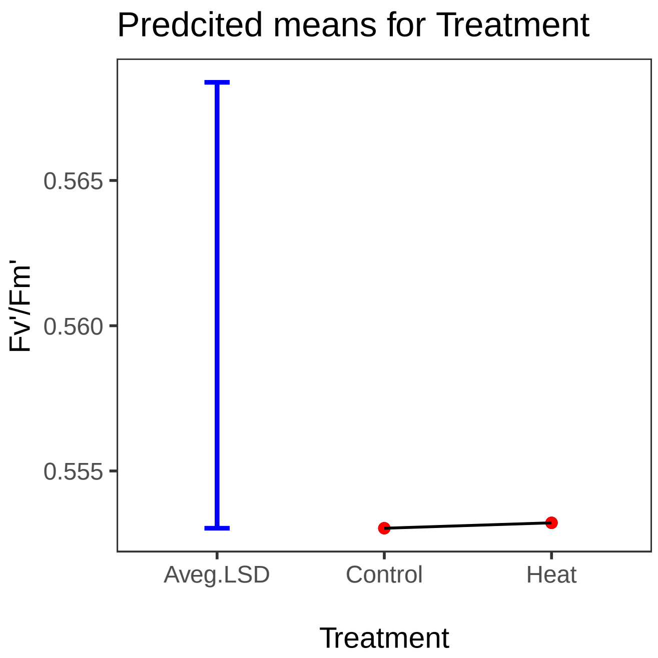
Predcited means for Treatment
Fv'/Fm'
0.565
0.560
0.555
Aveg.LSD
Control
Heat
Treatment

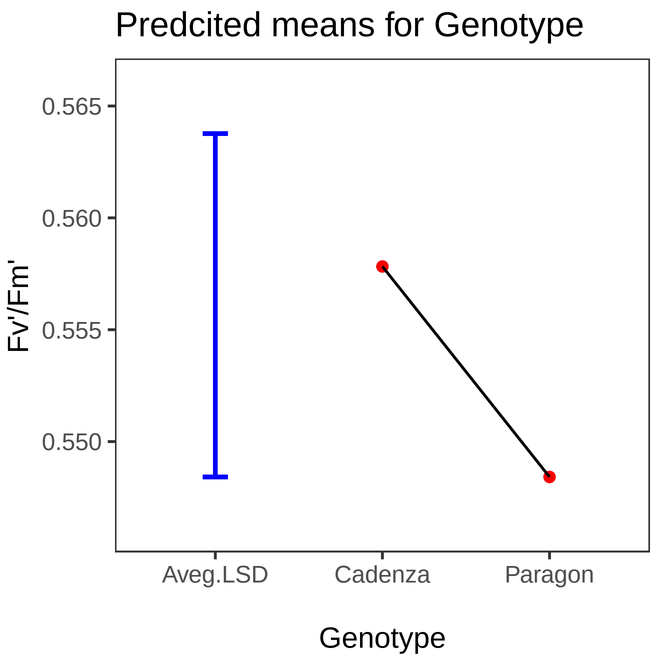
Predcited means for Genotype
Fv'/Fm'
0.565
0.560
0.555
0.550
Aveg.LSD
Cadenza
Paragon
Genotype

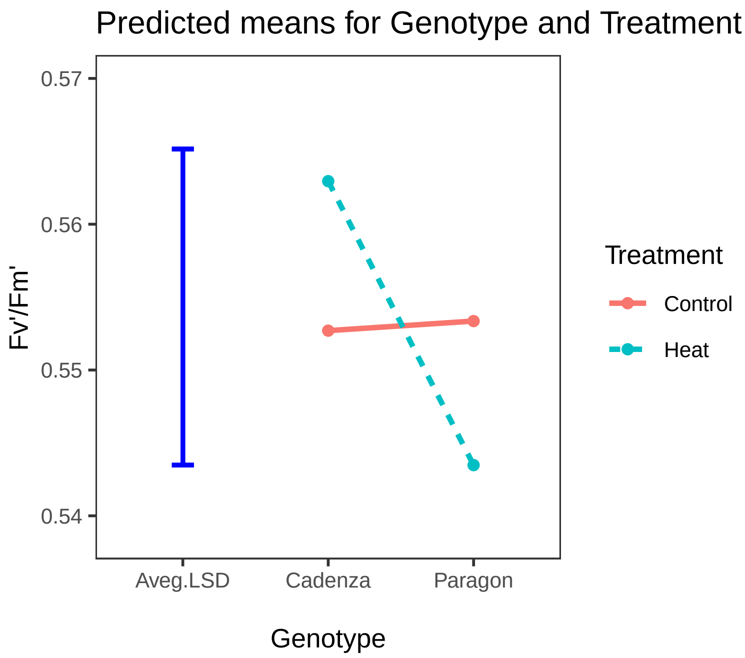
Predicted means for Genotype and Treatment
Fv'/Fm'
0.57
0.56
0.55
0.54
Aveg.LSD
Cadenza
Paragon
Genotype
Treatment
Control
Heat

Stomatal conductance (light)

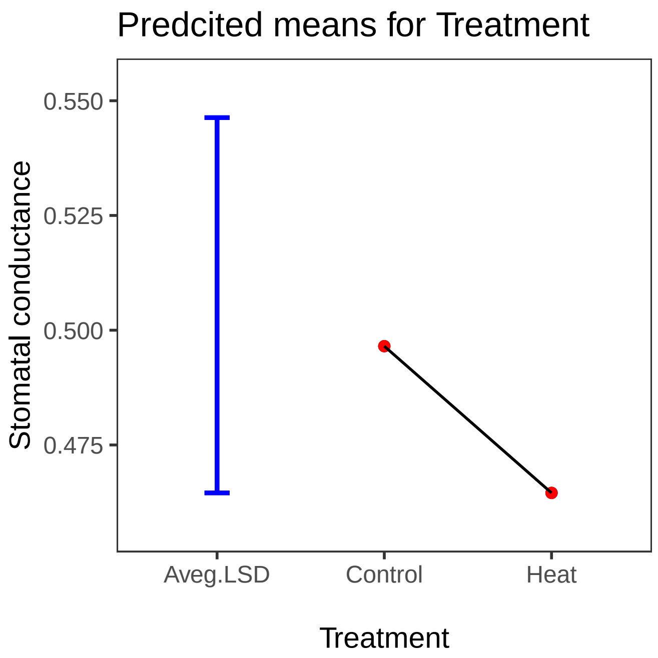

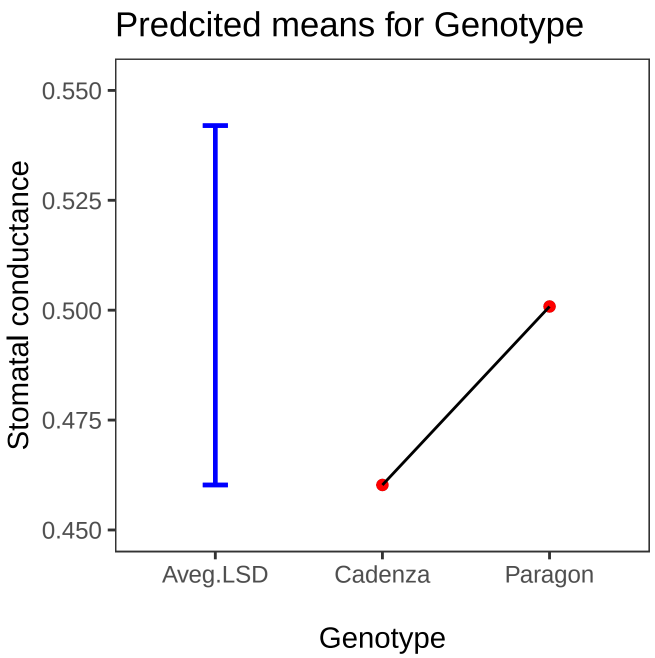

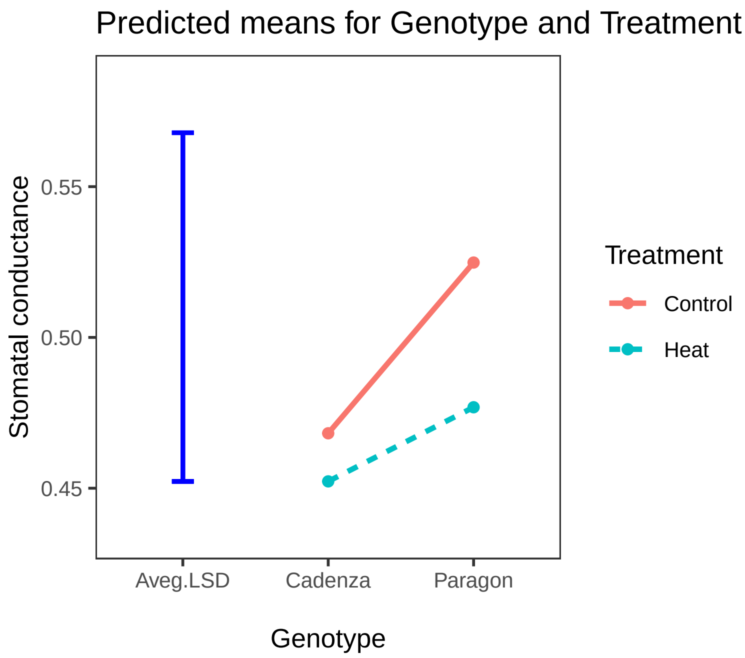

Stomatal conductance (dark)

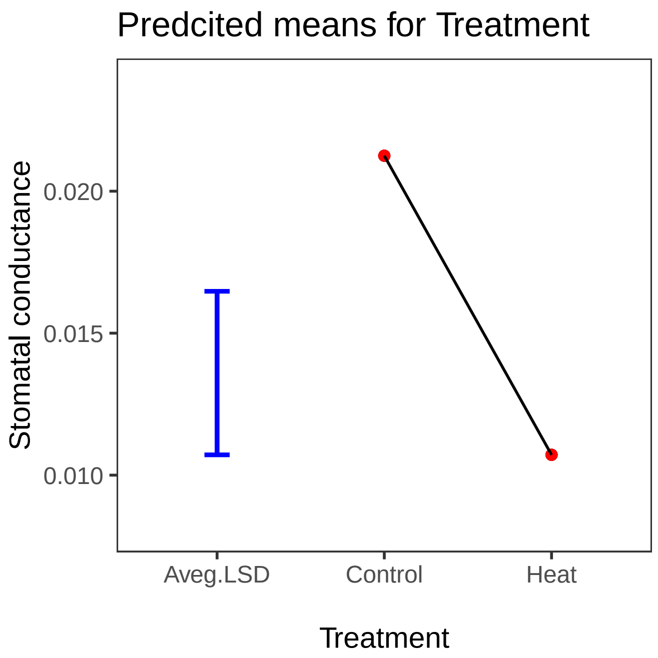

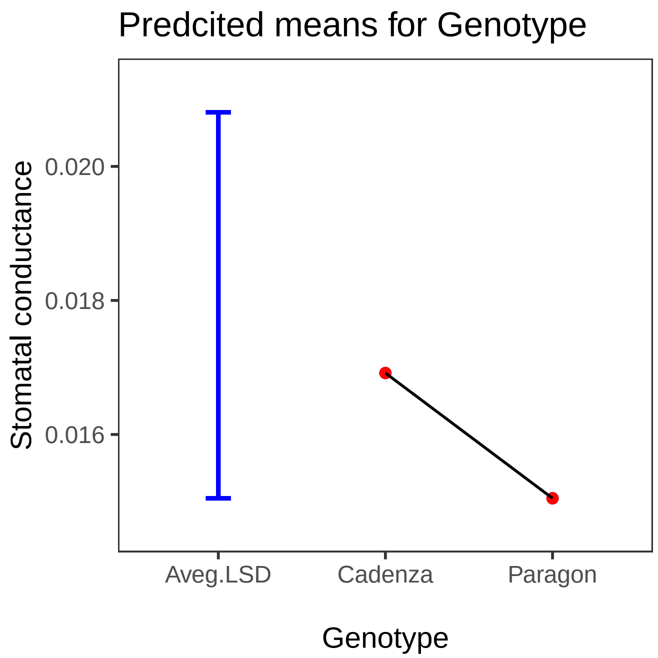

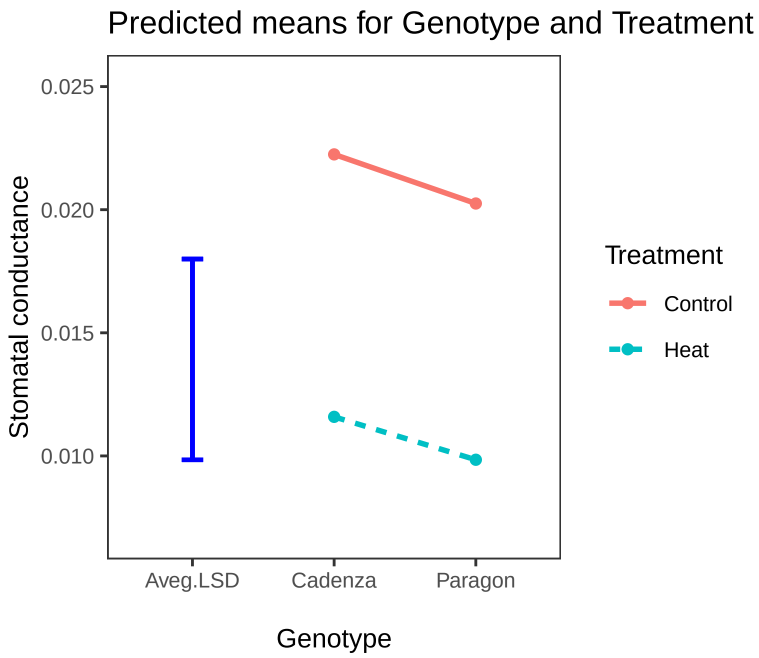

Vapour pressure deficit (air)

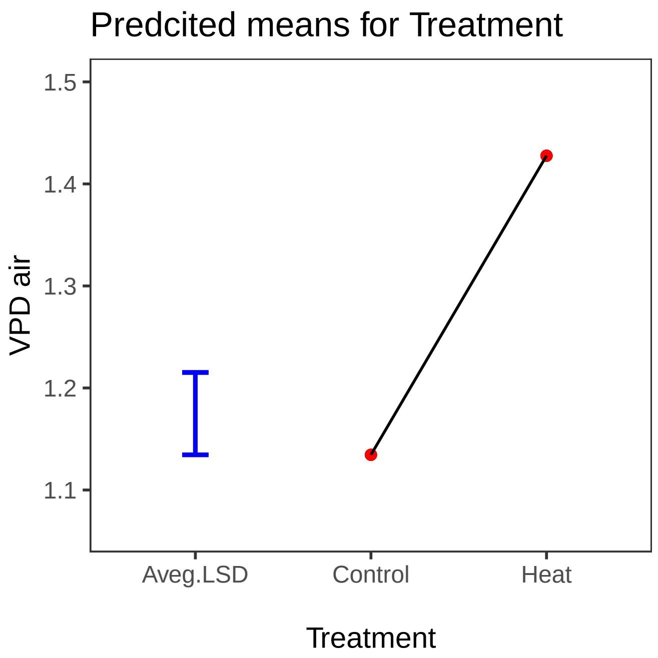

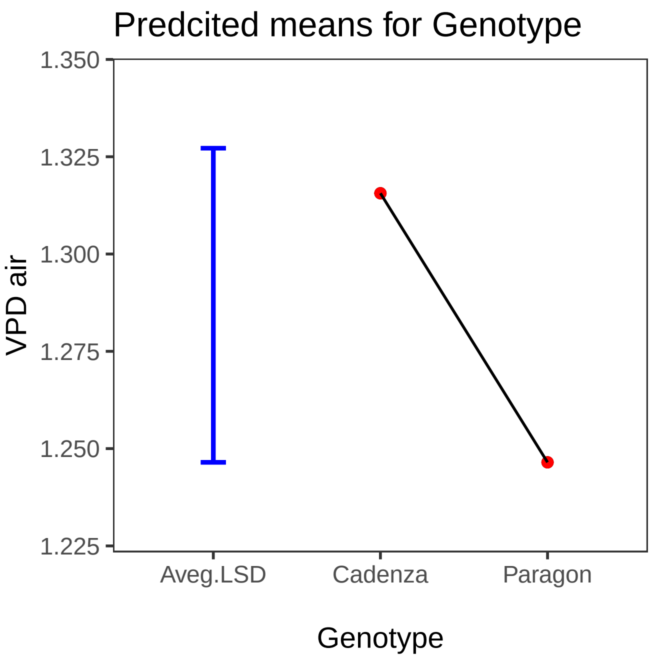

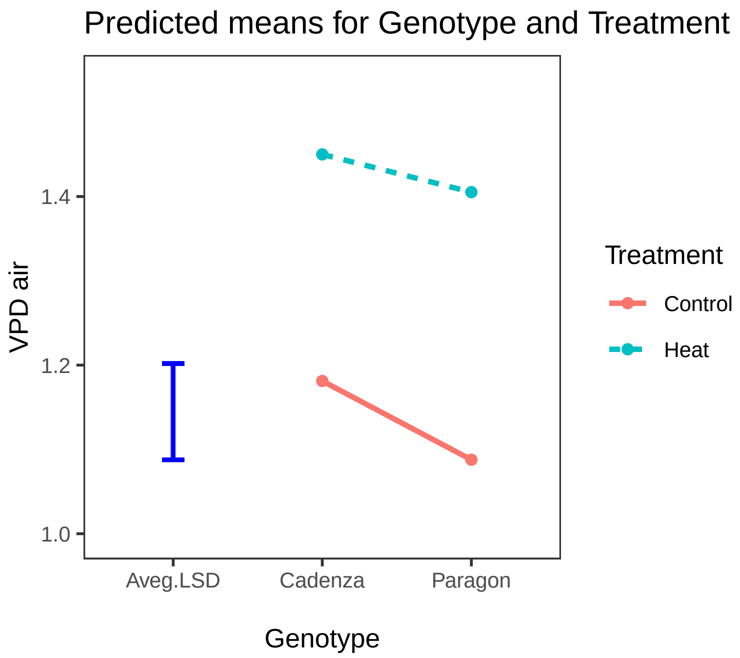

Vapour pressure deficit (leaf)

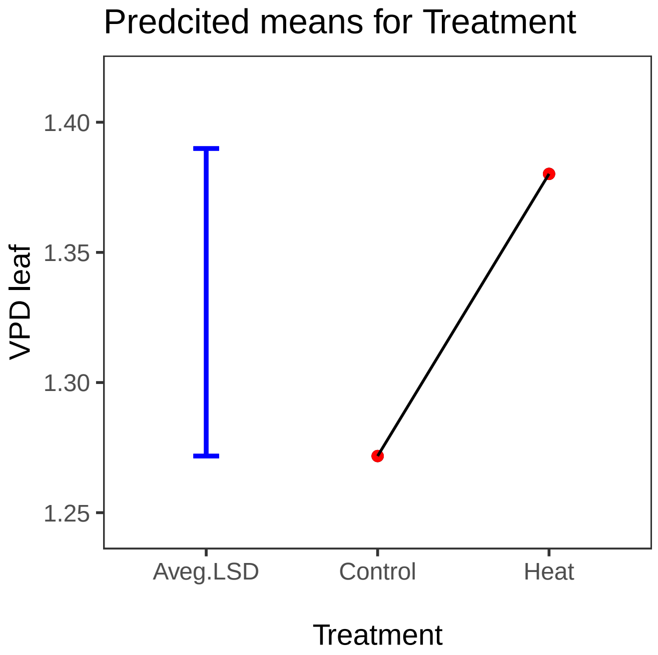

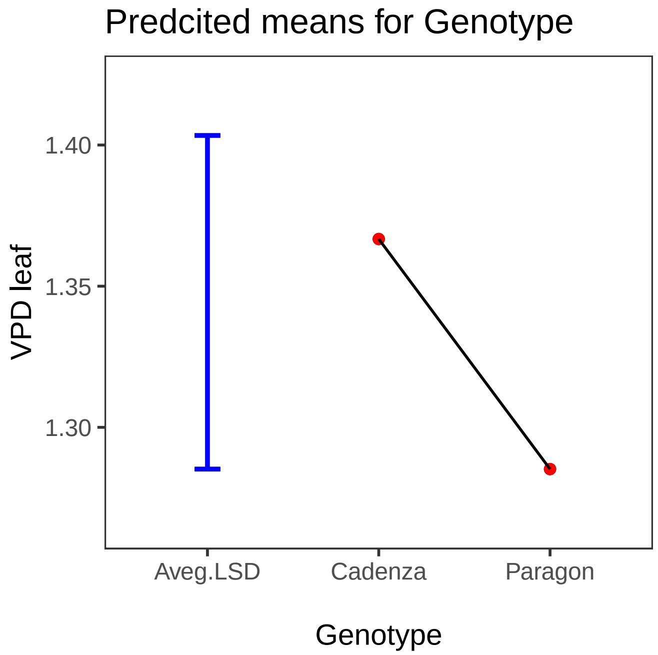

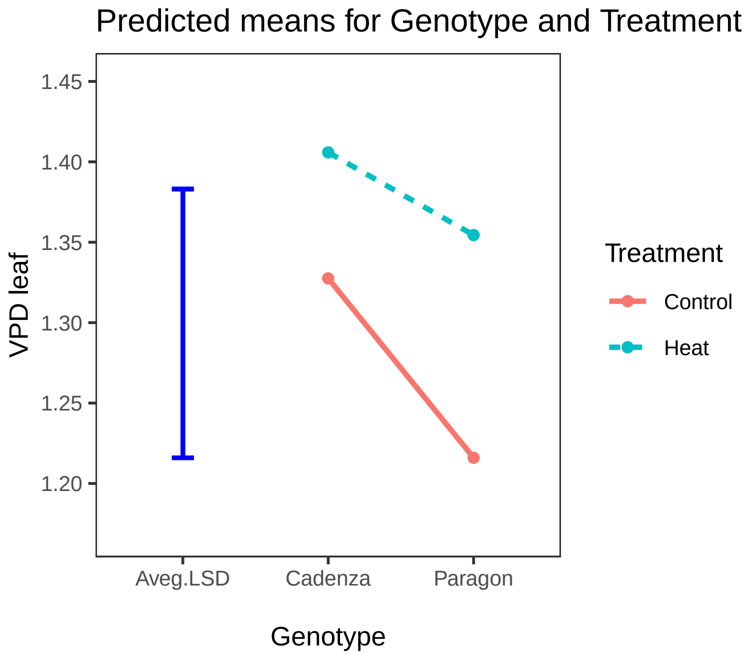

Supplement: Supplementary file 6 — Figure S3. [file PEI3-3-264-s005.pdf]
